# The Relevance of Thimet Oligopeptidase in the Regulation of Energy Metabolism and Diet-Induced Obesity

**DOI:** 10.3390/biom10020321

**Published:** 2020-02-17

**Authors:** Mayara C. F. Gewehr, Alexandre A. S. Teixeira, Bruna A. C. Santos, Luana A. Biondo, Fábio C. Gozzo, Amanda M. Cordibello, Rosangela A. S. Eichler, Patrícia Reckziegel, Renée N. O. Da Silva, Nilton B. Dos Santos, Niels O. S. Camara, Angela Castoldi, Maria L. M. Barreto-Chaves, Camila S. Dale, Nathalia Senger, Joanna D. C. C. Lima, Marilia C. L. Seelaender, Aline C. Inada, Eliana H. Akamine, Leandro M. Castro, Alice C. Rodrigues, José C. Rosa Neto, Emer S. Ferro

**Affiliations:** 1 Department of Pharmacology, Biomedical Sciences Institute, University of São Paulo, 05508-900 São Paulo, SP, Brazil; ferrari.mayaracalegaro@gmail.com (M.C.F.G.); bacsbruna@usp.br (B.A.C.S.); reichlerusp@gmail.com (R.A.S.E.); oliveirarenee@gmail.com (R.N.O.D.S.); niltonbsantos@gmail.com (N.B.D.S.); inadaaline@gmail.com (A.C.I.); eliakamine@usp.br (E.H.A.); alicecristinarodrigues@gmail.com (A.C.R.); 2 Department of Cell Biology and Development, Biomedical Sciences Institute, University of São Paulo, 05508-900 São Paulo, SP, Brazil; alexandreast@gmail.com (A.A.S.T.); luabiondo@gmail.com (L.A.B.); joannacarola@usp.br (J.D.C.C.L.); seelaend@icb.usp.br (M.C.L.S.); josecesar23@hotmail.com (J.C.R.N.); 3 Institute of Chemistry, State University of Campinas, 13083-862 Campinas, SP, Brazil; fgozzo@gmail.com (F.C.G.); amiquilini@id.uff.br (A.M.C.); 4 Department of Pharmacology, Federal University of São Paulo, 04023-062 São Paulo, SP, Brazil; reckziegel.patricia@gmail.com; 5 Department of Immunology, Biomedical Sciences Institute, University of São Paulo, 05508-900 São Paulo, SP, Brazil; niels.camara@gmail.com (N.O.S.C.); angela.castoldi@gmail.com (A.C.); 6 Department of Anatomy, Biomedical Sciences Institute, University of São Paulo, 05508-900 São Paulo, SP, Brazil; marialuizabarretochaves@gmail.com (M.L.M.B.-C.); camisdale@gmail.com (C.S.D.); nathaliasenger@usp.br (N.S.); 7 Biosciences Institute, São Paulo State University, 11330-900 São Vicente, SP, Brazil; leandromcbio@gmail.com

**Keywords:** obesity, insulin resistance, diet-induced obesity, proteasome, proteases, peptidases, mass spectrometry, peptidome

## Abstract

Thimet oligopeptidase (EC 3.4.24.15; EP24.15; THOP1) is a potential therapeutic target, as it plays key biological functions in processing biologically functional peptides. The structural conformation of THOP1 provides a unique restriction regarding substrate size, in that it only hydrolyzes peptides (optimally, those ranging from eight to 12 amino acids) and not proteins. The proteasome activity of hydrolyzing proteins releases a large number of intracellular peptides, providing THOP1 substrates within cells. The present study aimed to investigate the possible function of THOP1 in the development of diet-induced obesity (DIO) and insulin resistance by utilizing a murine model of hyperlipidic DIO with both C57BL6 wild-type (WT) and THOP1 null (THOP1^−/−^) mice. After 24 weeks of being fed a hyperlipidic diet (HD), THOP1^−/−^ and WT mice ingested similar chow and calories; however, the THOP1^−/−^ mice gained 75% less body weight and showed neither insulin resistance nor non-alcoholic fatty liver steatosis when compared to WT mice. THOP1^−/−^ mice had increased adrenergic-stimulated adipose tissue lipolysis as well as a balanced level of expression of genes and microRNAs associated with energy metabolism, adipogenesis, or inflammation. Altogether, these differences converge to a healthy phenotype of THOP1^−/−^ fed a HD. The molecular mechanism that links THOP1 to energy metabolism is suggested herein to involve intracellular peptides, of which the relative levels were identified to change in the adipose tissue of WT and THOP1^−/−^ mice. Intracellular peptides were observed by molecular modeling to interact with both pre-miR-143 and pre-miR-222, suggesting a possible novel regulatory mechanism for gene expression. Therefore, we successfully demonstrated the previously anticipated relevance of THOP1 in energy metabolism regulation. It was suggested that intracellular peptides were responsible for mediating the phenotypic differences that are described herein by a yet unknown mechanism of action.

## 1. Introduction

Mammalian proteasomes play essential functions in degrading proteins [1]. Proteasomes are also important in the processing of peptides for major histocompatibility class I (MHC-I) antigen presentation [2]. However, an evolutionary ancient function must exist for peptides processed by proteasomes, considering that active proteasomes are found in prokaryotes (some bacteria and archaea), while MHC-I is only found in cartilaginous fish [3,4,5,6] and not in earlier, more primitive species. Indeed, in the late 1950s, some intracellular peptides were described in *Pseudomonas hydrophila* [7] and *Torula utilis* [8]. Recently, our group developed a substrate-capture assay using a catalytically inactive form of thimet oligopeptidase (EC 3.4.24.15, EP24.15; THOP1) that allowed for the seminal identification of mammalian intracellular peptides, which are products of proteasome activity distinct from MHC-I antigens [9,10]. To date, multiple research groups have identified hundreds of novel intracellular peptides in human cell lines [11,12], human tissues [13,14], rodents [15,16], zebrafish [17], yeast [18], and plants [19,20]. THOP1 only hydrolyzes a restricted group of peptides in the optimal range of 8–12 amino acids in length [21,22,23], and has never been shown to degrade proteins, likely due to its catalytic site being deeply located in the bottom of a narrow channel [24,25]. Therefore, proteasome activity generates intracellular peptides that can eventually be processed by THOP1 [12,26,27]. THOP1 is ubiquitously present in the cytoplasm and nuclei of mammalian cells and tissues [28,29,30,31,32], and it is also extracellularly secreted [33,34,35,36,37,38,39] and membrane-associated [35,40,41,42,43]. THOP1 has been established as one of the highly expressed genes related to epigenetic interactions in lung adenocarcinoma of poor prognosis [44]; it is also associated with MHC-I antigen presentation [26,27,45,46,47] and the inactivation of several neuropeptides [41,42,48,49,50,51,52,53]. These data were recently corroborated by THOP1 C57BL6 null mice (THOP1^−/−^), which showed poor clinical scores compared to wild-type C57BL6 mice (WT) in an autoimmune encephalomyelitis neurodegeneration model for multiple sclerosis [54]. THOP1 was also shown to have roles in sepsis, peripheral bradykinin metabolism related to the inflammatory pain response, and in affecting animal behaviors such as depression, attention, and memory retention [54]. Previous studies have associated THOP1 with distinct human pathologies [44,55,56,57,58,59], including Alzheimer’s disease [59] and early diabetic retinopathy [55]. In a primate model of maternal obesity in which obesity was induced in baboons prior to pregnancy, THOP1 was identified as one of the five differentially expressed proteasome pathway genes targeted by four differentially expressed microRNAs [60]. Neurolysin, an oligopeptidase that is closely structurally related to THOP1 [23,61,62], was shown to be a key enzyme for energy metabolism, increasing glucose tolerance, insulin sensitivity, and gluconeogenesis [63]. Neurolysin was also shown to be a key enzyme for intracellular peptide metabolism [63,64]. Intracellular peptides were previously shown to have multiple functions, both inside and outside cells [65], such as facilitating glucose uptake [66] and activating fat metabolism [10,67].

Overweight and obesity are major risk factors for several chronic diseases, including diabetes, cardiovascular diseases, stroke, and cancer, according to the World Health Organization (WHO) 2019 report. There are currently over 1.9 billion people who are obese or overweight, leading to a rise in related health complications, including insulin resistance, type 2 diabetes, cardiovascular disease, liver disease, cancer, and neurodegeneration [68]. Therefore, the search for novel pharmacological targets for treating overweight, obesity, and obesity-related chronic diseases remains an important goal. In the present report, THOP1^−/−^ and WT animals were challenged by either a standard diet (SD, 3.8 kcal/g) or a hyperlipidic diet (HD, 5.4 kcal/g) for 24 weeks. Using this model, it was possible to investigate the potential functions of THOP1 on hyperlipidic diet-induced obesity (DIO) as well as some obesity-associated diseases such as insulin resistance. THOP1^−/−^ was considered a unique animal model to induce changes only in the intracellular peptide profile without disturbing protein degradation, due to THOP1’s well-characterized unconventional substrate-size restriction [21,22,23,24,62,69,70]. Moreover, THOP1^−/−^ mice were shown to be healthy and viable, in addition to having a normal external appearance, estrous cycle, and fertility [54]. Altogether, the results presented herein successfully suggest THOP1 is a novel target for controlling obesity and associated diseases. Intracellular peptides products of proteasome and THOP1 activities could be responsible for functionally mediating the phenotypic differences observed between THOP1^−/−^ and WT mice.

## 2. Materials and Methods

### 2.1. Generating and Genotyping THOP1^−/−^ Mice

A full detailed description of THOP1**^−/−^** mice generation and genotyping procedures were previously described [54]. Briefly, the THOP1 gene trap knockout mouse strain was generated by blastocyst microinjection of genetically modified embryonic stem cells (CSG163, 129ola) that were obtained from BayGenomics through the International Gene Trap Consortium. After transmission of the knock-out allele from chimera to F1 generation, the THOP1 mice were obtained from heterozygous breeding, and the line was further maintained on the mixed background by breeding THOP1^+/−^ with THOP1^+/−^ animals. After breeding these mice for several generations, the animals were genotyped while using a mouse 384 single nucleotide polymorphism (SNP) panel (SNP markers were spread across the genome at about 7 Mbp intervals; Charles River, NY, USA; data not shown). THOP1 was confirmed to be located on chromosome 10 at 81 Mb. The animal chosen for further breeding had no other 129 alleles unlinked to the THOP1 gene of interest, and was considered 99.35% congenic to C57BL/6N. The controls C57BL6/N WT animals used herein were not littermate, although all of the animals were indeed initially generated from C57BL/6N littermates. All of the animals lived and shared the same environment, and the same persons maintained them during the entire experimental process. The mice were maintained in individual ventilated cages (Ventilife, ALESCO, SP, Brazil) under standardized conditions with an artificial 12-h dark-light cycle, with free access to standard chow and drinking water *ad libitum*. The experiments were performed with mice four weeks old. All of the animals were maintained and used in accordance with the guidelines of the National Council for Control of Animal Experiments (CONCEA), following international norms of animal care and maintenance. Thus, we hereby state that the University of São Paulo Ethics Committee Councils from the Biomedical Science Institute previously approved all experimental protocols (approval number for mice experimentation ICB/USP No. 138/2015).

### 2.2. Diet-Induced Obesity (DIO) Murine Model and Food Restriction

The animals were fed either an SD with a caloric content of 3.8 kcal/g (70% carbohydrate, 20% protein, 10% fat; Nuvilab CR1, Nuvital Nutrientes S.A., Colombo, PR, Brazil), or with a HD with a caloric content of 5.4 kcal/g (25.9% carbohydrate, 14.9% protein, 59% fat; PragSoluções, Jaú, SP, Brazil). Four-week-old mice began being fed with either an SD or a HD for the next 24 weeks. Food restriction diet protocols were performed, as previously described [24]. Briefly, 12-week-old animals started being fed with only 1.5 g/day of an SD. This represented approximately 40% of the regular food ingestion of these animals per day. Some groups of animals were also administrated food orally by gavage, once a day, with the beta-adrenergic antagonist, propranolol (10 mg∙kg^−1^/day).

### 2.3. Adipose Tissue Image Analyses and Quantification

For the quantification of adipose tissue from WT or THOP1**^−/−^** animals, mice that were fed either with an SD or a HD were used. The in vivo imaging system MS-FX-PRO (Bruker BioSpin, Ettlingen, Germany; Research Facility CEFAP, ICB/USP) was used for in vivo image acquisition. The animals from each group were first anesthetized by intraperitoneal administration of a mixture containing ketamine (150 mg/kg) and xylazine (7.5 mg/kg). The generated images provided an accurate measurement of X-ray densities, and the analysis software that was integrated with the equipment allowed for the quantification of abdominal fat from the animals. The results are expressed as percentage of fat and the evaluated contents were compared among the groups.

### 2.4. Quantification of Lipid Content in Animal Feces

The determination of the lipid content in animal feces was performed by extraction while using an organic solvent, as previously described [71].

### 2.5. Food, Calories, and Water Consumption and Body and Tissue Weights

The body weight of the animals was determined weekly, as well as the consumption rates of food (g/day/animal), calories (kcal/day/animal), and water (mL/day/animal), over the 24 weeks for the experiments with different diets. The weight at the end of the 24 weeks was corrected by the nasoanal length to determine the Lee index (weight 1/3(g)/nasoanal length (cm)) × 100) [72]. The weights (g) of the liver, retroperitoneal, inguinal, and epididymal/ovarian adipose tissues, as well as the soleus and gastrocnemius muscles, were determined at the end of the 24 weeks.

### 2.6. Glucose and Insulin Tolerance Tests and Pyruvate Determinations

For the glucose tolerance test (GTT), the animals were first fasted for 10 h, and a tail blood drop was then used to determine the basal glucose levels while using a glucose meter (Accu-Check Performa, Roche, São Paulo, SP, Brazil). Subsequently, 2 g of glucose/kg of body weight was injected into the peritoneal cavity and tail blood glucose was measured at 15, 30, 60, 90, and 120 min after glucose injection. For the insulin tolerance test (ITT), similar procedures to those that are described above were followed. However, insulin was injected at a dose of 0.75 IU/kg of body weight and the blood glucose was measured at 15, 30, 60, 90, and 120 min after glucose injection. The results are expressed as the percentage of blood glucose reduction. Gluconeogenesis was assessed by injecting 2 g/kg sodium pyruvate after 16 h of fasting. In all three assays, glycemia was measured while using a glucose meter (Accu-Check Performa, Roche, São Paulo, SP, Brazil).

### 2.7. Blood Pressure and Heart Rate

Indirect systolic blood pressure (SBP) and heart rate (HR) were determined by tail-cuff plethysmography (Kent Scientific, Litchfield, CT, USA) in WT or THOP1**^−/−^** mice that were fed an SD or a HD. The mice were familiarized with the apparatus for a total of seven days before the measurements were taken. The measurements of SBP and HR were obtained in the 20th and 24th week of experimentation with different diets [73].

### 2.8. Western Blot Assays

THOP1 Western blot analyses were performed while using the Mini-Protean^®^ Tetra Cell apparatus (BioRad Laboratories, Inc., Hercules, CA, USA), as previously described [34]. Briefly, total proteins obtained from the 10,000× *g* supernatant of adipose tissue or liver homogenates were combined with an equal part of Laemmli buffer (BioRad Laboratories, Inc. USA), containing 5% of beta-mercaptoethanol and boiled at 95 °C for 5 min. Next, 80 µg of protein was submitted to electrophoresis on a 10% SDS-PAGE gel at constant voltage (90 V), and then blotted onto Immobilon® PVDF membranes (EMD Millipore Corporation, Temecula, CA, USA). The membranes were stained with Ponceau S to certify that equal protein amounts were loaded on the immunoblot membranes. The membranes were blocked with 3% bovine serum albumin (BSA) that was diluted in TBS-T buffer (50 mM Tris-HCL, 150 mM NaCl, 0.1% Tween 20, pH 7.5) for 1 h at room temperature, and subsequently incubated overnight at 4 °C with specific anti-THOP1 rabbit antiserum (1:1000; Proteimax Biotechnology LTDA, São Paulo, SP, Brazil), followed by incubation with specific anti-beta-actin mouse antiserum (1:5000; Sigma Aldrich, USA). After incubation with the primary antibodies, the membranes were developed while using the appropriate infra-red-conjugated secondary antibodies (dilution of 1:10,000; LI-COR, Inc., Lincoln, NE, USA) for 1 h at room temperature. The images were captured while using ChemiDoc MP Imaging Detection System and analyzed with Image Lab software (BioRad Laboratories, Inc., USA).

### 2.9. Blood Concentration of Glucose, Cholesterol, Triglycerides, and High-Density Lipoprotein (HDL) 

Glucose was determined by reactive tapes while using an Accu-Chek Performa glucose meter (Accu-Check Performa, Roche, São Paulo, SP, Brazil) in the blood drops that were obtained from the tail of animals that were awake and had previously fasted for 10 h. Additional biomarkers were determined in blood samples that were collected by cardiac puncture from animals fasted for 10 h and anesthetized with isoflurane. The serum that was obtained by centrifugation (3000 rpm for 15 min.) was stored at −80 °C until needed. Total cholesterol, HDL, and triglycerides were measured by colorimetric assays (kits Liquiform, Labtest, São Paulo, SP, Brazil).

### 2.10. Adiponectin Assays

The quantitative assessment of adiponectin was performed by enzyme-linked immunosorbent assay (ELISA) while using DuoSet ELISA^®^, according to the manufacturer’s instructions (R&D Systems, Minneapolis, MN, USA).

### 2.11. Quantification of Blood Peptide Hormones by Luminex^®^

The blood samples were collected by cardiac puncture and placed in tubes with protease inhibitors (Complete ™Protease Inhibitor Cocktail, Sigma-Aldrich, St. Louis, MO, USA) and a dipeptidyl peptidase IV inhibitor (Januvia^®^, Merck Sharp & Dohme Limited, Broxbourne, UK). Serum was stored at −80 °C until the day of the assays, as previously described [74]. The Milliplex^®^ MAP magnetic sphere panel metabolic hormone kit (MMHMAG-44K, Merck Millipore, São Paulo, SP, Brazil) was adopted to detect the markers: C-peptide, ghrelin, gastric inhibitor peptide (GIP), glucagon-like peptide (GLP-1), interleukin 6 (IL-6), insulin, leptin, monocyte chemotactic protein (MCP-1), polypeptide (PP), YY peptide (PYY), and resistin. For these assays, the serum samples were incubated for 2 h with a mixture of Magplex^®^ microspheres (Grand Island, NY, USA) that were coated with the respective antibodies. The detection of target antigens bound to the microspheres was conducted while using a mixture of biotinylated capture antibodies and incubation for 1 h followed by incubation with phycoerythrin-labeled streptavidin for 30 min. The microspheres were then identified by means of phycoerythrin while using the Magpix^®^ instrument (Life Technologies, Grand Island, NY, USA). The calculated values of each analyzed hormone were expressed relative to the total protein concentration based on standard curves.

### 2.12. Indirect Calorimetry

Indirect calorimetry allows for monitoring the energy metabolism of resting animals by measuring spontaneous activity, heat, oxygen consumption (VO_2_), and carbon dioxide production (VCO_2_). In addition, the respiratory exchange ratio (RER) can be calculated by the VCO_2_/VO_2_ ratio to evaluate energy use and energy expenditure, as previously described [75].

### 2.13. Corticosterone Levels

From the animal tail tip, 100 µL of blood was taken and plasma was prepared by centrifugation at 1900× *g* at 4 °C for 10 min. The levels of corticosterone were determined according to the kit manufacturer’s instructions (cat. no. ADI-900-097; Enzo Life Sciences, Inc., Farmingdale, NY, USA).

### 2.14. Histological Analyses

The liver tissue samples were withdrawn and then immersed in a fixative solution containing paraformaldehyde in 0.1 M PBS buffer for 24 h. The samples were then washed with 0.1 M PBS buffer and finally stored in 70% alcohol until histological preparation. The tissues were dehydrated through successive bathing in a series of solutions with increasing ethanol concentration (70%, 95%, and 100%). They were then diaphanized in xylol and impregnated in histological paraffin. The paraffin blocks were sliced while using a microtome (5–8 μm thick slices) to obtain slices from various regions of the tissues being analyzed. The sections were stretched in a water bath (40 °C) and placed on glass slides covered with Meyer’s albumin for fixation. Once mounted onto the glass slides, the tissue slices were placed in a 55 °C oven for approximately 4 h for better fixation and to remove the excess paraffin. The slides were then dewaxed while using xylol and hydrated using a series of solutions of decreasing ethanol concentration (100%, 95%, and 70%) and, finally, water, so that the dye could penetrate the tissue. The slices were then stained with either hematoxylin-eosin (HE) or periodic acid-Schiff (PAS) stains. PAS is commonly used to stain the main polysaccharide in human and animal tissue sections, that is, glycogen. After staining, the sections were dehydrated again, rinsed in xylol, and finally covered with a glass cover.

### 2.15. Quantitative Real-Time PCR (qRT-PCR)

Quantitative real-time polymerase chain reaction (qRT-PCR) was conducted to determine the expression of specific mRNAs, as previously described [76,77]. Table S1 shows the primer sequences. The relative quantification of dicer and microRNAs was conducted by stem–loop RT-qPCR [78]. The TaqMan assays required for each microRNA detection were purchased from Applied Biosystems (assays ID: 456 for mmu-miR-130b-3p; 2249 for mmu-miR-143-3p; 439 for mmu-miR-103-3p; 2276 for mmumiR-222-3p; mmu480910_mir for mmu-miR-127-3p; mmu480946_mir for mmu-miR-149-5p; mmu480960_mir for mmu-miR-182-5p; and, mmu478318_mir for mmu-miR-212-3p). For the expression of either the mRNAs or the microRNAs, the relative quantification method was applied, while using the average Ct of sno202 as a reference, according to the 2^−∆∆CT^ method. We analyzed the results from the qRT-PCR assays while using the Pfaffl equation [77]. We calculated Ct = (Ct of the target gene in the WT group − Ct of the target gene in the THOP**^−/−^** group)/(Ct of the housekeeping gene in the WT group − Ct of the housekeeping gene in the THOP**^−/−^** group). We transformed the Ct variation ratios into fold change using the 2^−Ct^ formula because PCR products are exponentially produced.

### 2.16. Glycerol (lipolysis) Dosage

The animals were anesthetized with isoflurane, and samples from inguinal adipose tissue were taken and weighed. First, the samples were separated into two pieces and both were placed in Krebs buffer (values in mM: 118 NaCl, 4.7 KCl, 1.2 MgSO_4_, 1.2 KH_2_PO_4_, 2.5 CaCl_2_, and 25 NaHCO_3_; pH 7.2) for 10 min. One adipose tissue piece was placed in Krebs buffer alone to determine the basal glycerol concentration (basal group). The other piece was placed in Krebs buffer containing 0.10 ng/g isoproterenol (ISO) to detect post-stimulus glycerol levels. Glycerol was quantified while using the Free Glycerol Reagent kit (Sigma, MO, USA). Tissue weight normalized glycerol levels.

### 2.17. Progressive Treadmill Test

The exercise capacity, as estimated by the total distance traveled, correlates with the skeletal muscle’s working capacity. Here, the exercise capacity of the animals was evaluated while using a graded treadmill, as previously described [63].

### 2.18. Peptide Isolation and Quantification Using Isotopic Labeling

Peptide isolation and concentration were conducted, as previously described [79]. The procedures for reductive methylation used in isotope labeling were performed, as previously described [80]. Five biological samples were prepared for each group (WT/SD, WT/HD, THOP1**^−/−^**/SD, THOP1**^−/−^**/HD). These samples were labeled twice (forward and reverse labeling) and pooled into 10 runs for LC/MS. The odd runs were different biological samples and the even runs were technical replicates of each biological sample. Table S2 and Figure S6 show a schematic view of the sample preparation.

### 2.19. Mass Spectrometry and Data Analyses

LC-MS and LC-MS/MS experiments were performed in an Orbitrap (OT) Fusion Tribrid mass spectrometer (Thermo Fisher Scientific, Waltham, MA, U.S.A) within the facilities at Harvard Medical School while using similar conditions to those previously described [81]. Briefly, OT top speed data-dependent precursor ion selection was used. The parent ions were scanned with an OT resolution of 60 K and an automatic gain control (AGC) target of 3,000,000. Dynamic exclusion was turned on with the following settings: exclusion time = 60 s; mass tolerance = ± 7 ppm; and, repeat count = 1. For OT detection, the parent ions were scanned in the range of 395–1800 m/z, and the fragment ions were scanned with the Ion Trap (IT) with a maximum injection time of 200 ms and an AGC target of 50,000. Precursor ions with +2 and +3 charges were considered. A normalized collision energy of 35% was used for CID fragmentation. The raw data files were converted into a peak list format (mgf) by Mascot Distiller version 2.7.1 (Matrix Science Ltd., London, UK) and analyzed while using the search engine MASCOT version 2.6.2 (Matrix Science Ltd., London, UK) to identify peptides. The databases used for searching included SwissProt (555,100 sequences; 198,754,198 residues) and the taxonomy *Mus musculus* (16,916 sequences). No cleavage site was specified and a mass tolerance of 0.5 Da was applied to the MS and MS/MS ions. The included variable modifications were N-terminal protein acetylation, methionine oxidation, and also the reductive Me labels: Dimethyl (K), Dimethyl (N-term), Dimethyl:2H(2) (N-term), Dimethyl:2H(2) (K), Dimethyl:2H(4) (K), Dimethyl:2H(4) (N-term), Dimethyl:2H(6)13C(2) (K), and Dimethyl:2H(6)13C(2) (N-term). All of the search results were manually interpreted to eliminate false positives. In brief, the criteria included (1) observed mass within 40 ppm of the theoretical mass; (2) the number of Me tags observed that matched the number of free amines available (i.e., Lys residue and N-terminus if not acetylated); (3) observed charge state(s) of the peptide was consistent with the expected number of positive charges; and, (4) ≥80% of the major fragments that were observed in MS/MS-matched b or y ions, with a minimum of five matches. Relative quantification of each peptide present in both WT and THOP1**^−/−^** mice fed either with an SD or a HD, labeled either “Light 1”, “Light 2”, “Intermediate”, or “Heavy” isotopes, were automatically performed while using the Mascot software (http://www.matrixscience.com), version 2.6.2 (Matrix Science Ltd., London, UK). The peptides that showed differences between treatments in automatic quantification were manually confirmed while using multiple scans of the MS spectra. Manual quantification was performed by determining the relative intensity of each isotopic peak, while considering both the monoisotopic peak and the peak containing one atom of ^13^C and then subtracting the baseline due to overlapping lower-mass peaks. The intensity of each peptide (labeled with one of the isotopic forms) was compared with the same peptide that was labeled with another isotopic form (Mass Spectrometry, Supporting Information). In these analyses, the peptides that increased more than 100% (two-fold) or decreased more than 50% (0.5-fold) as compared to the respective control WT mice, fed either an SD or a HD, were selected. Only peptides that were present in both duplicates (“forward” and “reverse”) from at least three different animals (from the initial five biological replicates) were considered. Peptides that showed differences between treatments in automatic quantification were manually checked (Mass Spectrometry, Supporting Information). Complete mass spectrometry proteomics data were deposited to ProteomeXchange Consortium via PRIDE [82] partner repository with the dataset identifier PXD016265 and 10.6019/PXD016265 (“Intracellular peptides are linked to obesity and obesity-associated diseases”).

### 2.20. Intracellular Peptide Modeling and Docking with microRNAs

Conformational modeling and docking of microRNAs and intracellular peptides were carried out, as previously described [83]. Molecular dynamic simulation was done while using GROMACS 4.6.1 to check for conformational stability [84]. All of the atomistic simulations were carried out while using the CHARMM27 all-atom force field (version 2.0) with the periodic boundary condition [85,86]. Electrostatic interactions were calculated while using the particle mesh Ewald (PME) summation. The microRNA was modeled using the mc-fold/mc-sym pipeline [87]. Docking between the modeled intracellular peptides and microRNAs was performed using ZDock 3.0.2. ZDock uses a fast Fourier transformation (FFT) algorithm to search the rotational space [88]. The models were ranked according to their ZDock score, which is based on a shape complementarity scoring function [88].

### 2.21. Statistics

Values are expressed as means ± standard error of the mean (SEM). Statistical analyses were conducted while using Prisma software. Statistical significance was based on Student’s unpaired *t*-test for independent samples or the ANOVA test to compare more than two groups. *p*-values < 0.05 were considered to be significantly different.

## 3. Results

### 3.1. THOP1^−/−^ Animals Gained Much Less fat and Weight than WT Animals, Despite having a Similar Consumption of Chow, Calories, and Water

**Figure 1:**
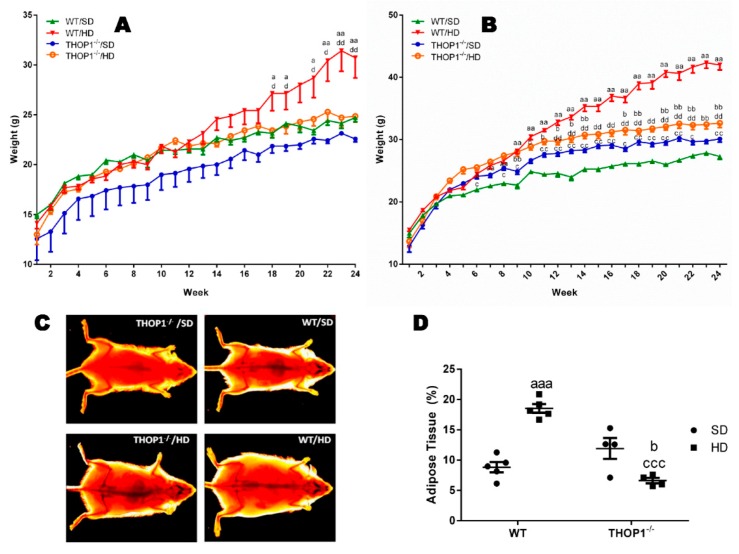
Weight and adipose tissue gain of animals during the 24 weeks of the diets. (**A**) females; (**B**–**D**) males. (**A**,**B**) show that wild-type (WT) animals fed a hyperlipidic diet (HD) began to weigh more than THOP1^−/−^ mice after 18 (females) or 12 (males) weeks, depending on gender. After 24 weeks, the adipose tissue (fat) content was observed by X-ray density images (**C**,**D**). Results are expressed as mean ± standard error of the mean (SEM). Statistical analyses were conducted using two-way ANOVA followed by Tukey’s test (**A**,**B**) or Student’s unpaired *t*-test (**D**). One letter, *p* ≤ 0.05; two letters, *p* ≤ 0.01; three letters *p* ≤ 0.001. a, WT standard diet (SD) vs. WT/HD; b, THOP1^−/−^/SD vs. THOP1^−/−^/HD; c, WT/SD vs. THOP1^−/−^/SD; d, WT/HD vs. THOP1^−/−^/HD (*n* = 5–9).

The animals were four weeks old and their weights were determined before they began to be fed either an SD or a HD; their weights were checked weekly for 24 weeks. After the 12th week, females and males that were fed HD began to show significant differences in their body weight when compared to animals fed an SD (Figure 1). The body weight gained by THOP1**^−/−^** animals fed a HD was significantly lower than of WT animals fed a HD (Figure 1). Similarly, WT females that were fed a HD gained more body weight than THOP1**^−/−^** females fed a HD (Figure 1A). THOP1**^−/−^** male animals receiving an SD gained slightly more body weight along the 24 weeks than WT males fed an SD (Figure 1B). In vivo X-ray imaging (Figure 1C,D) suggests that male WT and THOP1**^−/−^** mice that were fed an SD had similar fat contents (8.98% ± 0.85% vs. 6.58% ± 0.45%, respectively; *n* = 4–5). WT animals fed a HD had 18.2% ± 0.72% fat when compared to 12.64% ± 1.7% of THOP1**^−/−^** animals (*n* = 4–5). These data suggest that WT male mice gained 5.5% more body fat than THOP1**^−/−^** male mice when both were fed a HD (Figure 1C,D).

The differences in body weight of WT and THOP1**^−/−^** mice could be better visualized after subtracting the value for their body weights that were measured after the 24th week from their respective body weights determined before the dietary experiments started (Table 1). These data show that, after 24 weeks, WT females that were fed a HD gained an extra 6.9 g of body weight compared to WT females fed an SD (Table 1). In the same period, THOP1**^−/−^** females fed a HD gained only 3.9 g, which corresponds to 56% of the body weight gained by WT females fed a HD (Table 1). Male WT animals fed a HD gained an extra 14.2 g of body weight when compared to WT males fed an SD (Table 1). THOP1**^−/−^** animals fed a HD during the same period gained only 3.8 g, corresponding to 27% of the body weight gained by WT animals fed a HD (Table 1).

Despite the differences in body weight, the food, water, and caloric intakes among these different groups were similar (Table 2). The Lee index [72] of animals fed a HD was larger than those of animals fed an SD after 24 weeks (Table 2). The weight of the liver, adipose tissues (retroperitoneal, inguinal, epididymal/ovarian), and muscles (soleus and gastrocnemius) was determined and corrected by the body weight of the respective animal (Table 2). The livers of WT and THOP1**^−/−^** females as well as THOP1**^−/−^** males receiving a HD weighed less than their respective control animals fed an SD (Table 2). The adipose tissue weights were not different among WT and THOP1**^−/−^** animals that were fed an SD when comparing both female and male groups (Table 2). However, adipose tissues from THOP1**^−/−^** female and male animals fed a HD weighed significantly less than the respective tissues from WT animals that were fed a HD (Table 2). The soleus and gastrocnemius muscles weighed less in WT and THOP1**^−/−^** females fed a HD compared to their respective control females fed an SD (Table 2). The gastrocnemius muscle from THOP1**^−/−^** animals fed a HD weighed more compared to their respective WT control animals (Table 2). Taken altogether, the above results corroborate the X-ray analyses (Figure 1C,D), showing that the greatest differences in body weights from WT and THOP1**^−/−^** animals are related to their fat content. As shown above, the food, water, and caloric intakes among these different groups were similar. In addition, there were no observed differences in the total fecal lipid content of both WT and THOP1**^−/−^** mice that were fed either an SD or a HD during the 24-week experiment (Supplementary Materials, Figure S1).

### 3.2. WT but Not THOP1^−/−^ Animals fed a HD Developed Insulin Resistance without Signs of Metabolic Syndrome

**Figure 2:**
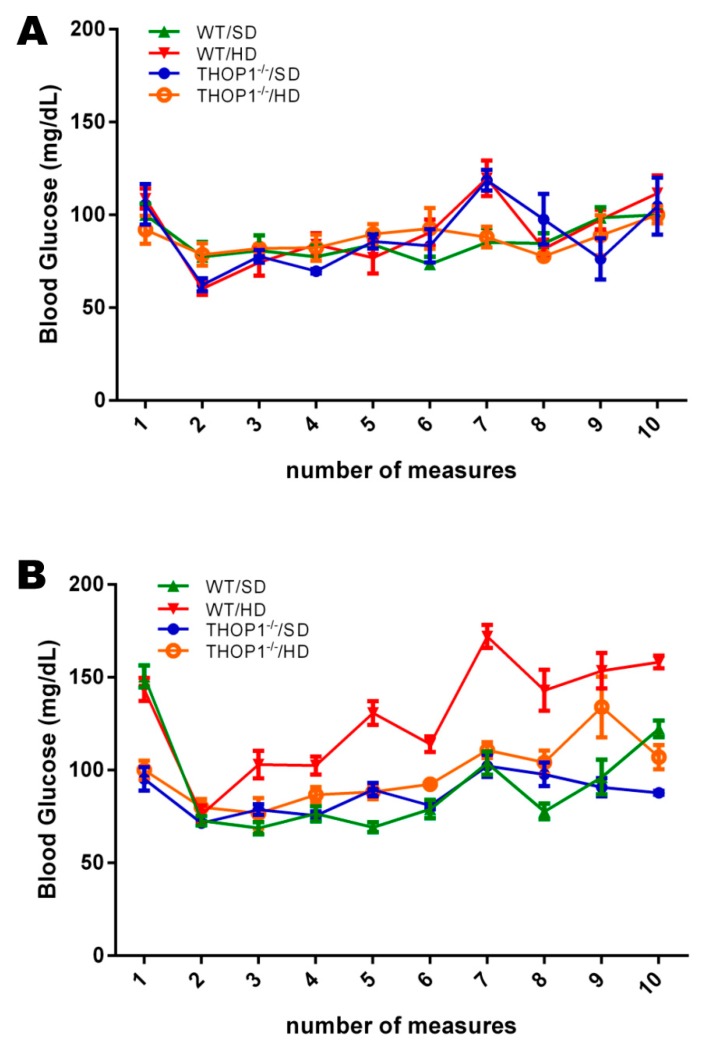
Blood glucose levels. Pre-prandial glucose levels of female (**A**) and male (**B**) mice, WT or THOP1^−/−^, fed either an SD or a HD, were evaluated every two weeks across the 24 weeks. Note that only WT male mice fed a HD showed an increase in the pre-prandial blood glucose levels across the 24 weeks. Results are expressed as mean ± SEM. Statistical analyses were conducted using Two-way ANOVA followed by Tukey’s test or Student’s unpaired *t*-test (*n* = 6–9).

Blood glucose monitoring was performed weekly throughout the 24-week experiment in animals that were fasted for 10 h prior to measurement (Figure 2). The group of WT male animals that were fed a HD (Figure 2B) was the only one to present a gradual and sustained increase in blood glucose levels during the 24 weeks of the experiment (Figure 2).

**Figure 3:**
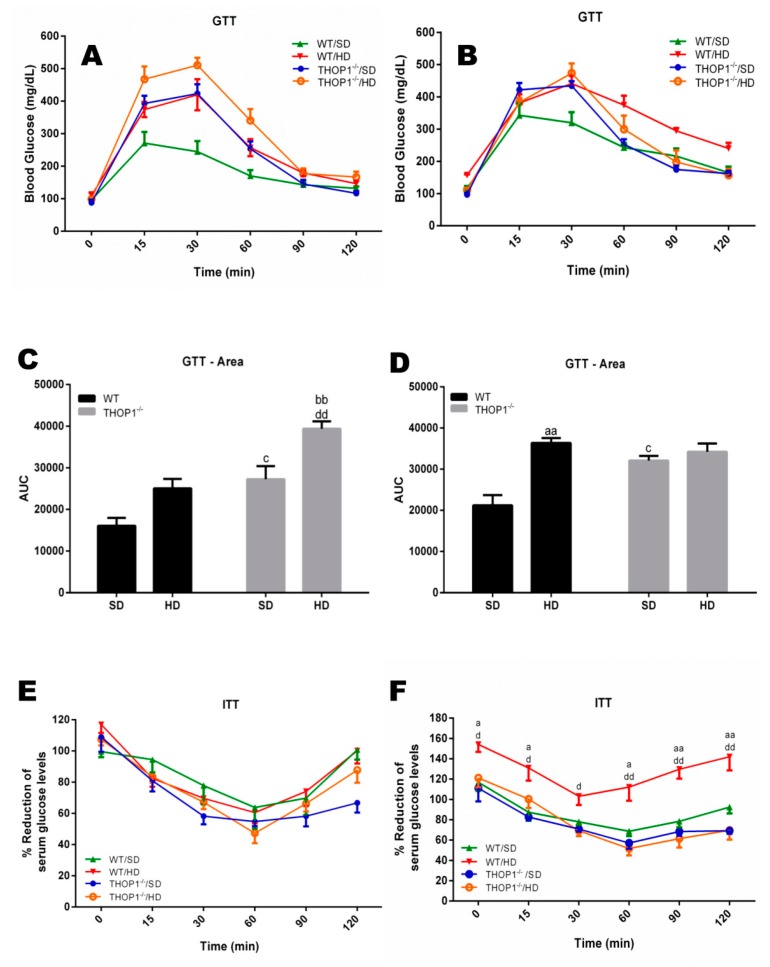
Glucose and insulin tolerance tests. (**A**,**C**,**E**) females and (**B**,**D**,**F**) males. (**A**–**D**) show the glucose tolerance test (GTT). (**E**,**F**) show the insulin tolerance test (ITT). (**C**,**D**) show the area under the curve (AUC) for groups A (**C**) or B (**D**). Note that only WT male animals fed a HD for 24 weeks were insulin-resistant (**F**). Data are presented as mean ± SEM. Statistical analyses were conducted using Two-way ANOVA followed by Tukey’s test (**A**,**B**,**E**,**F**) or Student’s unpaired *t*-test (**C**,**D**). One letter, *p* ≤ 0.05; two letters, *p* ≤ 0.01; three letters *p* ≤ 0.001. a, WT/SD vs. WT/HD; b, THOP1^−/−^/SD vs. THOP1^−/−^/HD; c, WT/SD vs. THOP1**^−/−^**/SD; d, WT/HD vs. THOP1**^−/−^**/HD (*n* = 6–9).

At the end of the 24 weeks, tests for glucose and insulin tolerance, respectively, GTT and ITT, were performed on both males and females that were fed either an SD or a HD (Figure 3). The GTT results suggest that THOP1**^−/−^** males and females that were fed either an SD or a HD had a slower decay in blood glucose levels than the WT animals that were fed an SD (Figure 3A–D). This phenotypic difference was more evident in THOP1**^−/−^** females (Figure 3A,C). After 120 min. of GTT experiments, the blood glucose levels of all groups, except for the WT fed a HD, returned to the initial basal levels (Figure 3A,B). Next, the ITT results suggest that male WT animals that were fed a HD developed insulin resistance, whereas all other groups remained sensitive to insulin (Figure 3E,F). The insulin blood levels of WT and THOP1**^−/−^** animals fed an SD, both females and males, fasted for either 4 or 10 h, were similar; these data suggest that pancreatic beta cells were regularly functioning in these animals (Figure S2). THOP1 protein immunoreactivity, either on liver or adipose tissue, was similar among WT animals that were fed either an SD or a HD, suggesting that THOP1 expression was not induced by DIO in these tissues (Figure S3). However, a higher expression of THOP1 could be observed in the adipose tissue of females as compared to males, fed either SD or HD (Figure S3A).

Hypertension and type 2 diabetes are two common obesity- and overweight-associated diseases. Cardiac functional parameters, such as systolic blood pressure (SBP) and heart rate (HR), were not altered in WT or THOP1**^−/−^** mice that were fed with either an SD or a HD (Table 3). Therefore, it can be concluded that the DIO model used herein causes insulin resistance and obesity without inducing metabolic syndrome.

### 3.3. Typical Biochemical Parameters and Endocrine Signaling Peptides Further Corroborate the Distinguishable DIO Phenotypes of WT and THOP1^−/−^

**Figure 4:**
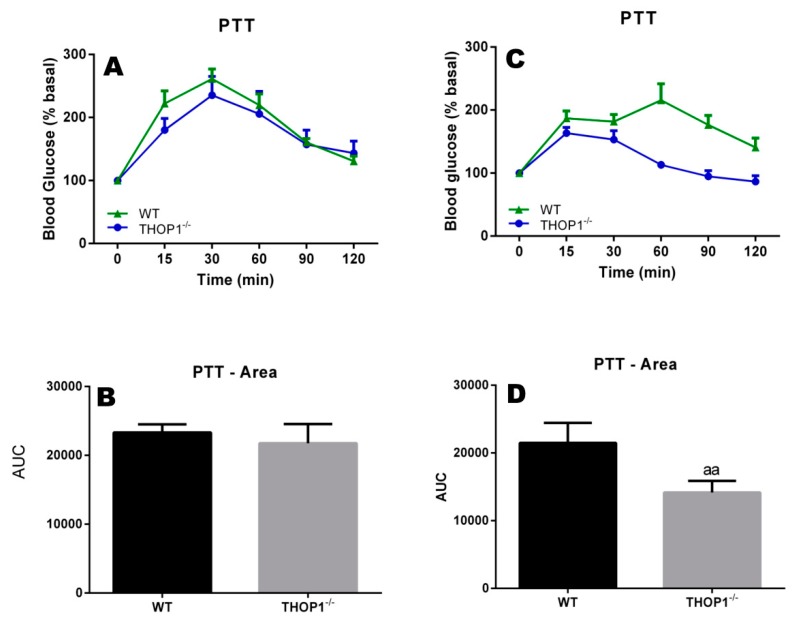
Pyruvate tolerance test of 24-week-old WT or THOP1**^−/−^** mice fed an SD to evaluate their liver gluconeogenesis. (**A**,**B**) females; (**C**,**D**) males; (**B**,**D**) respectively, A and C area under the curve (AUC) for blood glucose curves. Colored curves (**A**,**C**) show blood glucose levels after pyruvate injection (2 g/kg sodium pyruvate after 16 h of fasting) at the indicated time point (x-axis). Blood glucose was measured using a glucose meter as detailed in the Methods section. Statistical analyses were performed using Student’s unpaired *t*-test. aa, *p* ≤ 0.01 (*n* = 6).

Common blood biochemical parameters for evaluating energy metabolism were investigated herein. First, pyruvate tests were conducted. The results suggest that WT and THOP1**^−/−^** females (with a regular SD) had similar gluconeogenetic abilities (Figure 4A,B). Male THOP1**^−/−^** animals that were fed an SD also had normal gluconeogenetic capacities (Figure 4C,D). However, the total amount of glucose produced from the pyruvate by male THOP1**^−/−^** animals was lower compared to control male WT animals over the 120 min. period of analyses (Figure 4D). One possibility for explaining these results is that male THOP1**^−/−^** mice have reduced gluconeogenesis ability.

Blood cholesterol was higher in both female and male WT mice that were fed a HD as compared to WT fed an SD (Table 4). Among the THOP1**^−/−^** groups fed either an SD or a HD, no differences were observed in their cholesterol levels, whereas reduced levels of cholesterol were observed in THOP**^−/−^** mice fed a HD when compared to WT mice fed a HD (Table 4). The blood high-density lipoprotein (HDL) levels were reduced in THOP1**^−/−^** females fed a HD as compared to WT females fed a HD (Table 4). THOP1**^−/−^** mice were observed to have higher blood triglyceride levels when compared to WT mice fed either an SD or a HD (Table 4). These results corroborate previous studies associating alterations in THOP1 genetic features to alterations in human cholesterol metabolism [89,90].

Neuronal and endocrine signals are well known for acting on the hypothalamus to control hunger and satiety through orexigenic and anorexigenic peptides. The adiponectin levels were similar in WT and THOP1**^−/−^** animals that were fed either an SD or a HD (Figure S4). In WT animals, for both females and males, the blood levels of insulin and resistin increased, while ghrelin decreased in animals that were fed a HD as compared to those fed an SD (Table 5). On the other hand, the levels of these hormones remained similar in THOP1**^−/−^** animals (Table 5). These data corroborate the observations above that THOP1**^−/−^** animals do not develop obesity. The detection limit for several additional analytes evaluated herein (leptin, C-peptide, PYY, glucagon, TNF-α, MCP-1, GLP-1, GIP, IL-6, and PP) was out of the range of the standard curve (data not shown).

### 3.4. The Higher Response of Adipose Tissue to Beta-Adrenergic Stimulation Performs a Key Function in the Greater Resistance to DIO and Improved Cardiorespiratory Fitness of THOP1^−/−^ Mice

**Figure 5:**
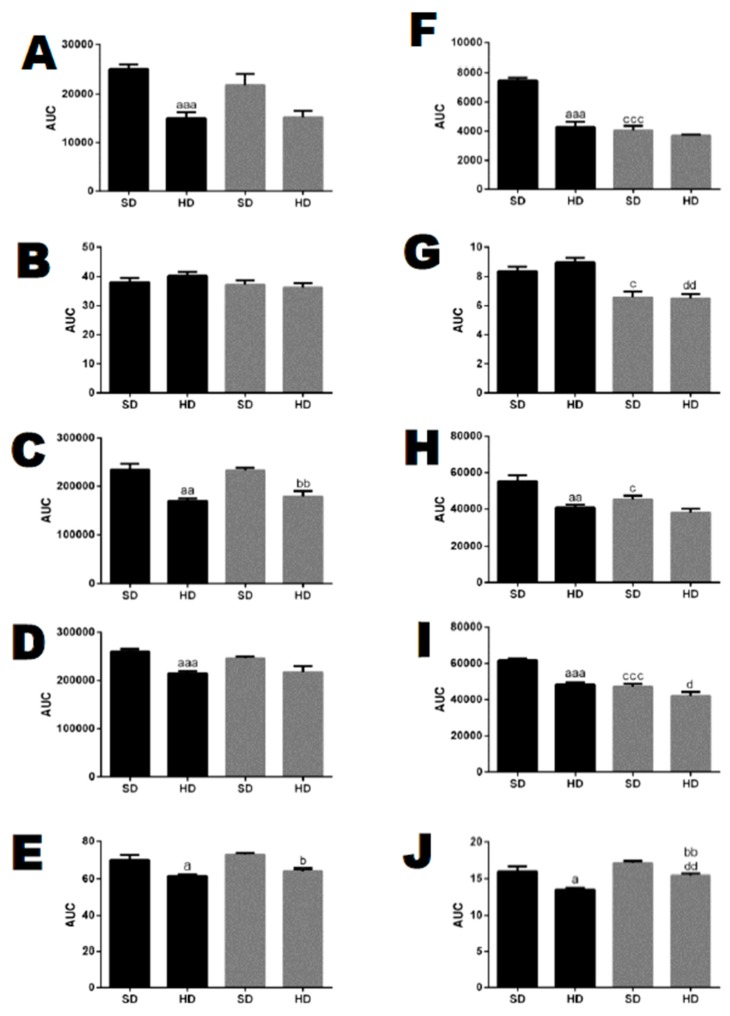
Resting energy metabolism of WT (black bars) or THOP1^−/−^ (green bars) male mice across 24 h. (**A**–**E**) 24 h period (6 a.m.–6 a.m.); (**F**–**J**) 5–10 p.m. period; (**A**,**F**) spontaneous locomotor activity; (**B**,**G**) heat production; (**C**,**H**) VCO_2_ (mL/kg/h) production; (D,I) VO_2_ (mL/kg/h) consumption; (**E**,**J**) respiratory exchange ratios (RERs). The results are shown for mice previously fed with either an SD or a HD for 24 weeks. Data are presented as mean ± SEM. Statistical analyses were performed using Student’s unpaired *t*-test. One letter, *p* ≤ 0.05; two letters, *p* ≤ 0.01; three letters *p* ≤ 0.001. a, WT/SD vs. WT/HD; b, THOP1**^−/−^**/SD vs. THOP1**^−/−^**/HD; c, WT/SD vs. THOP1**^−/−^**/SD; d, WT/HD vs. THOP1**^−/−^**/HD (*n* = 4). y-axis: bars represent the area under the curve (AUC).

The data above show that the restricted body weight gain of THOP1**^−/−^** mice could possibly be explained by higher energy expenditure (higher metabolic rate and/or physical activity). The resting energy metabolism of WT and THOP1**^−/−^** mice was evaluated through the measurement of spontaneous activity, heat production, oxygen consumption (VO_2_), and carbon dioxide production (VCO_2_) over 24 h (Figure 5A–E; Figure S5). Heat production remained similar between the WT and THOP1**^−/−^** male groups while considering the entire 24 h period (Figure 5B). However, only considering the period from 5to 10 p.m. (17:00–22:00), which includes the light/dark exchange period, the heat production of THOP1**^−/−^** mice that were fed either an SD or a HD was smaller compared to their respective WT control (Figure 5G; Figure S5). The spontaneous activity, VCO_2_, and VO_2_ levels of WT mice fed a HD were smaller than that of WT mice fed an SD, both during the entire 24 h period and from 5 to 10 p.m. (Figure 5A,C,D). The total spontaneous activity and VO_2_ of THOP1**^−/−^** mice that were fed either an SD or a HD was not different from WT mice during the 24 h period (Figure 5A,D), whereas the VCO_2_ of THOP1**^−/−^** mice fed a HD was smaller than that of THOP1**^−/−^** mice fed an SD (Figure 5C). During the 5–10 p.m. interval, the total spontaneous activity, VCO_2_, and VO_2_ levels of THOP1**^−/−^** mice fed an SD were smaller than that of WT mice that were fed an SD (Figure 5F,H,I; Figure S5). Among the different THOP1**^−/−^** groups, the spontaneous activity, VCO_2_, and VO_2_ levels were similar (Figure 5F,H,I). When the respiratory exchange ratio (RER; an RER of 0.7 indicates that fat is the predominant fuel source, a value of 1.0 is indicative of carbohydrates being the predominant fuel source, and a value between 0.7 and 1.0 suggests that a mix of both fats and carbohydrates) was observed during the 24 h period, both WT and THOP1**^−/−^** mice that were fed a HD had a smaller RER compared to their respective control groups fed an SD (Figure 5E). These data suggest that both WT and THOP1**^−/−^** mice fed a HD used more fat as a fuel source (Figure 5J; Figure S5). Altogether, these data indicate that the reduced body weight and adipose tissue of the THOP1**^−/−^** animals could be due to their distinctive fat metabolism, which did not induce higher heat production, nor was it related to higher locomotor activity. These latter parameters were even lower while considering the interval from 5–10 p.m.

**Figure 6:**
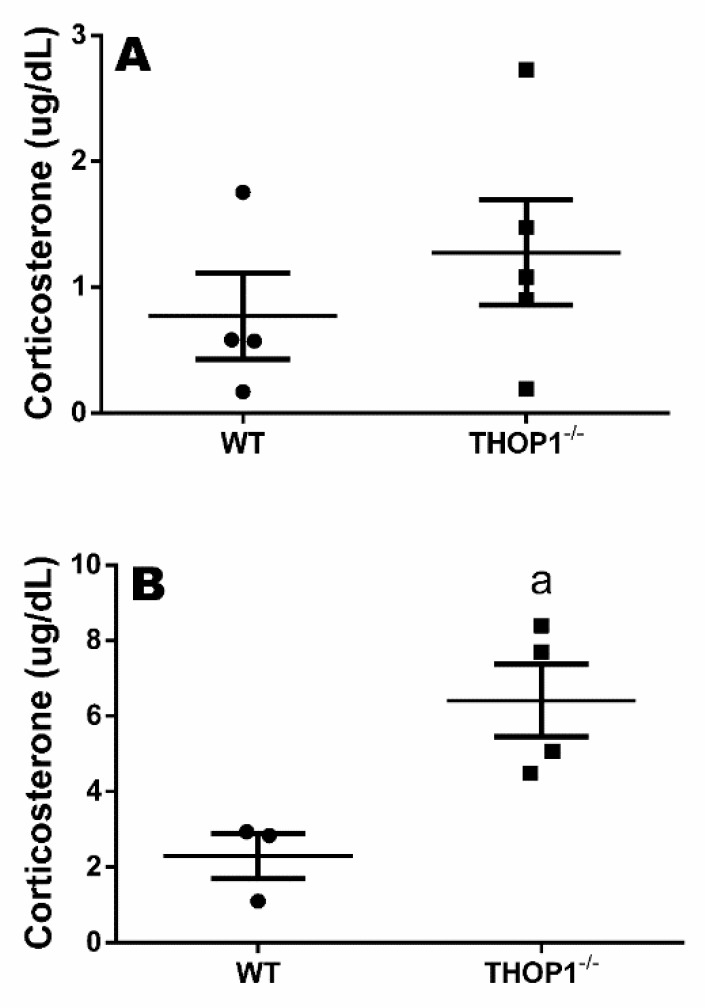
Plasma corticosterone levels of WT and THOP1^−/−^ mice. (**A**) plasma corticosterone levels (µg/dL) at 7 a.m.; (**B**) plasma corticosterone levels (µg/dL) at 7 p.m. Results are expressed as mean ± SEM. Statistical analyses were performed using Student’s unpaired *t-*test. a, *p* ≤ 0.05 between WT and THOP1**^−/−^** mice (*n* = 4–5).

Previous reports show that, in rodents, plasma corticosterone levels peak at the beginning of the dark phase, preceding the start of their activity period [91]. Indeed, WT and THOP1**^−/−^** animals both had significantly higher levels of corticosterone at 7 p.m. than at 7 a.m. (Figure 6). However, at 7 p.m., the corticosterone levels of THOP1**^−/−^** animals were even higher than those of WT animals (Figure 6). These transient high corticosterone levels of THOP1**^−/−^** mice could be associated with the differences observed in their heat production, spontaneous activity, VO_2_, VCO_2_, and RER levels during the interval from 5 to 10 p.m. Furthermore, these short and transient higher corticosterone levels of THOP1**^−/−^** mice could be beneficial in enabling a lower inflammatory response in these animals.

**Figure 7:**
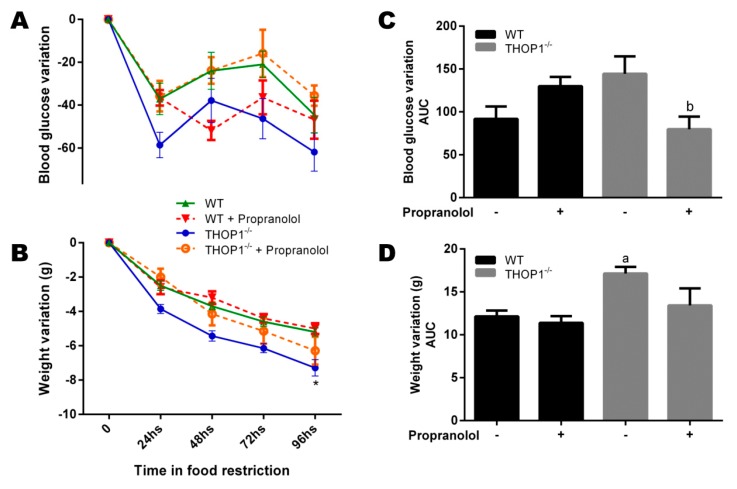
Food restriction experiments. Twelve-week-old WT and THOP1^−/−^ animals were fed with 1.5 g/day of standard diet (SD; corresponding to 40% of the regular fed for these animals). During four days, once a day, these animals received by gavage either saline or propranolol (10 mg∙kg^−1^/day). (**A**) blood glucose variation; (**B**) area under curve (AUC) of blood glucose variation; (**C**) animal weight variation; and, (**D**) AUC of animal weight variation. Results are expressed as mean ± SEM. Statistical analyses were performed using Student’s unpaired *t*-test. One letter *p* ≤ 0.05; between: a, WT vs. THOP1**^−/−^** WT/HD; b, THOP1**^−/−^** vs. THOP1**^−/−^** + propranolol (*n* = 7–10).

THOP1**^−/−^** and WT male mice were subjected to a caloric restriction diet for 96 h. THOP1**^−/−^** mice under caloric restriction lost more weight than WT animals (Figure 7). The body weight loss of THOP1**^−/−^** animals under caloric restriction was completely prevented by the administration of propranolol (Figure 7B,D). The blood glucose levels of THOP1**^−/−^** animals were lower than that of WT animals in the first 24 h of caloric restriction, and at 48 h of caloric restriction, their blood glucose levels returned to normal and remained similar to those of the WT animals (Figure 7). The treatment of WT animals with propranolol also prevented the reduction of blood glucose levels, mainly at 48 h (Figure 7).

**Figure 8:**
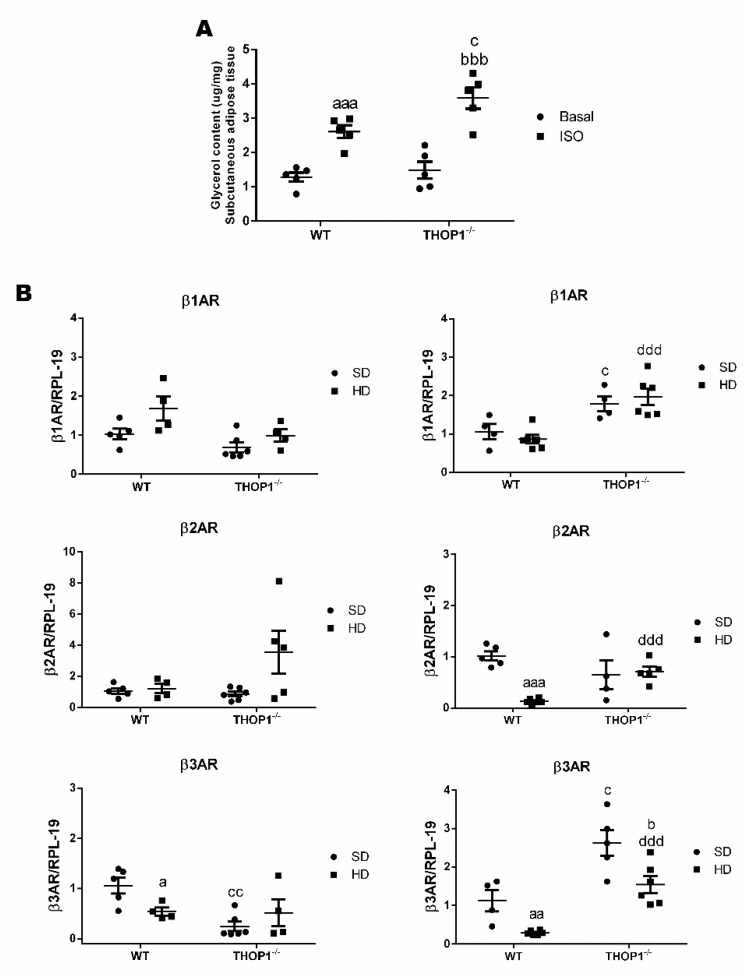
Isoproterenol-stimulated lipolytic activity and mRNA expression levels of β-adrenergic receptors 1, 2, or 3 (β1AR, β2AR, and β3AR, respectively). (**A**) Inguinal adipose tissue was removed, weighed, divided in two pieces, and placed in Krebs buffer, pH 7.2, at 37 °C for 10 min., in the absence (Basal) or presence of isoproterenol (0.10 ng/g; ISO). Lipolytic activity was measured as the amount of glycerol produced, using the “free glycerol reagent” kit (Sigma, MO, USA). Glycerol content (µg/mg) was normalized by tissue weight. Results are expressed as mean ± SEM. (**B**) mRNA expression levels of adrenergic receptors β1AR, β2AR, and β3AR. Analyses of gene expression were performed by Quantitative Real-Time PCR (qRT-PCR) in female or male retroperitoneal adipose tissue. Data are presented as mean ± standard deviation. Statistical analyses were performed using Student’s unpaired *t*-test. One letter, *p* ≤ 0.05; two letters, *p* ≤ 0.01; three letters *p* ≤ 0.001. a, WT/SD vs. WT/HD; b, THOP1**^−/−^**/SD vs. THOP1**^−/−^**/HD; c, WT/SD vs. THOP1**^−/−^**/SD; d, WT/HD vs. THOP1**^−/−^**/HD (*n* = 4–6).

The lipolysis of adipose tissue is a catabolic process stimulated by the adrenergic system that leads to the breakdown of triglycerides stored in adipose cells releasing fatty acids and glycerol. The dysregulation of the processes that are involved in lipolysis has already been observed in obesity [92,93]. To evaluate whether the lipolytic pathway of THOP1**^−/−^** animals was altered, a glycerol dosage test was performed. The basal- and isoproterenol-stimulated lipolytic activities of inguinal adipose tissue were both investigated. The basal lipolytic activities of inguinal adipose tissue in both WT and THOP1**^−/−^** mice were similar (Figure 8). After isoproterenol stimulation, an increase in the lipolytic activity was observed in the inguinal adipose tissues of both WT and THOP1**^−/−^** animals (Figure 8). However, under the same isoproterenol stimulus, the increase in the lipolytic activity of THOP1**^−/−^** animals was significantly higher than that of WT animals (Figure 8A). The mRNA expression levels of adrenergic receptors beta1 (B1AR), beta2 (B2AR), and beta3 (B3AR) were investigated herein. In male animals that were fed a HD, the expression of B1AR, B2AR, and B3AR were all higher in THOP1**^−/−^** when compared to WT mice (Figure 8B). Together, the caloric restriction, lipolytic activity, and adrenergic receptor expression analyses suggest that, at least in part, THOP1**^−/−^** mice may not gain weight/higher fat content due to a more efficient response to adrenergic stimulus.

**Figure 9:**
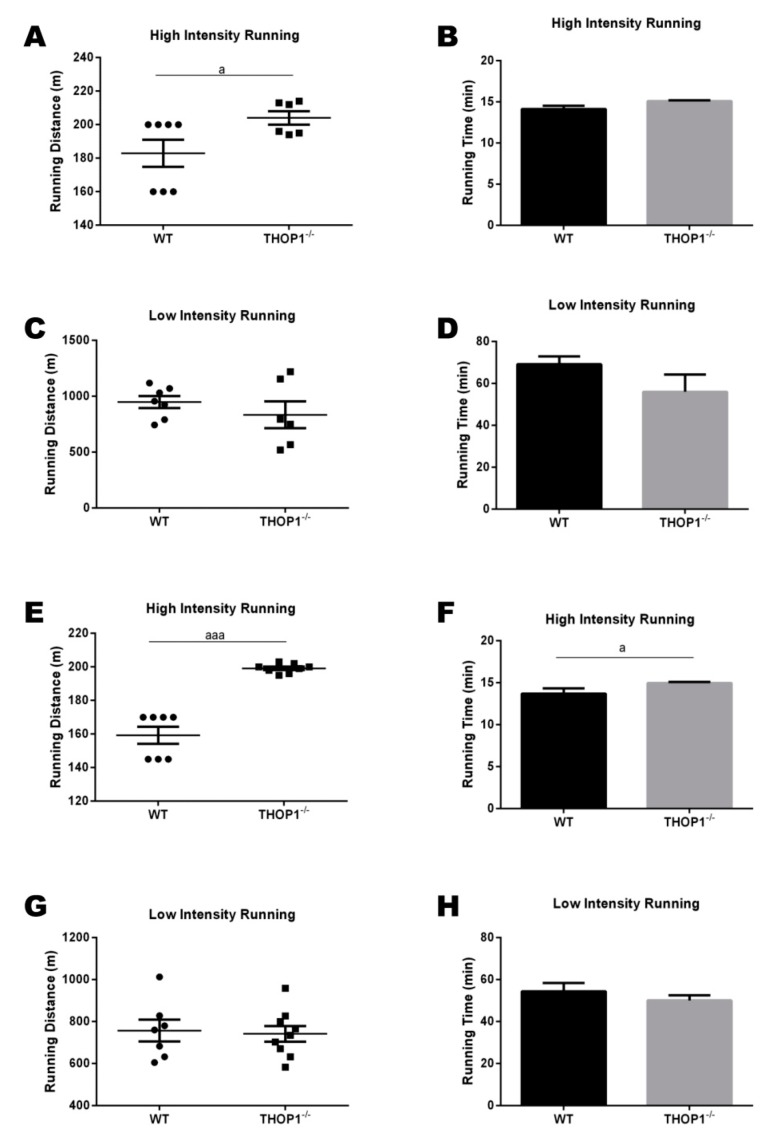
Progressive treadmill exercise test. The cardiorespiratory fitness of the animals fed with a standard diet (SD) was evaluated using a graded treadmill. (**A**,**C**,**E**,**G**) running distance (m); (**B**,**D**,**F**,**H**) running time (min.). Both running distance and time were evaluated in the high- and low-intensity tests. (**A**–**D**) females; (**E**–**F**) males. Data are presented as mean ± SEM. Statistical analyses were performed while using Student’s unpaired *t*-test. One letter, *p* ≤ 0.05; three letters *p* ≤ 0.001 (*n* = 7–9).

The cardiorespiratory fitness of the animals fed a standard diet (SD) was evaluated while using a graded treadmill. At the highest intensity of the treadmill test, for both females (Figure 9A,B) and males (Figure 9E,F), the THOP1**^−/−^** animals ran longer than the WT animals before exhaustion (Figure 9). At a low intensity in the treadmill tests (approximately 21 m/min. corresponding to 60% of the mean value of the maximum speed traveled by these animals during the previous high intensity treadmill test), no differences were seen when comparing THOP1**^−/−^** and WT mice. The higher lipolytic ability of THOP1**^−/−^** mice could have contributed to a better performance on the high-intensity treadmill test, thus increasing its oxidative metabolism.

### 3.5. Liver Morphological and Molecular Analyses Suggest that THOP1^−/−^ Mice Have Increased Glycogen Stores and Diminished Sensitivity to Non-Alcoholic Fatty Liver Steatosis (NAFLS)

**Figure 10:**
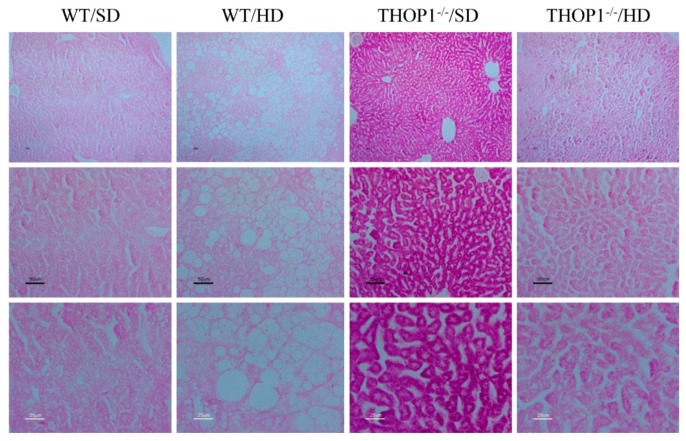
Liver slices from WT and THOP1**^−/−^** mice stained with periodic acid–Schiff (PAS). WT or THOP1**^−/−^** male mice were fed for 24 weeks with either an SD (WT/SD, THOP1**^−/−^**/SD) or a HD (WT/HD, THOP1**^−/−^**/HD). Note the higher intensity of PAS reaction in THOP1**^−/−^**/SD when compared to the other groups; this is an indicative of higher glycogen content in liver of THOP1**^−/−^** mice fed an SD. Panels from top to bottom present increased magnifications of 40- (top), 100- (middle), or 200-fold (bottom).

Staining male liver slices with periodic acid–Schiff (PAS) suggested that THOP1**^−/−^** mice that were fed an SD had increased glycogen accumulation when compared to WT animals fed an SD (Figure 10). Higher glycogen content was also present—although to a smaller extent—in the livers of THOP1**^−/−^** mice fed a HD as compared to their respective WT controls fed a HD (Figure 10).

**Figure 11:**
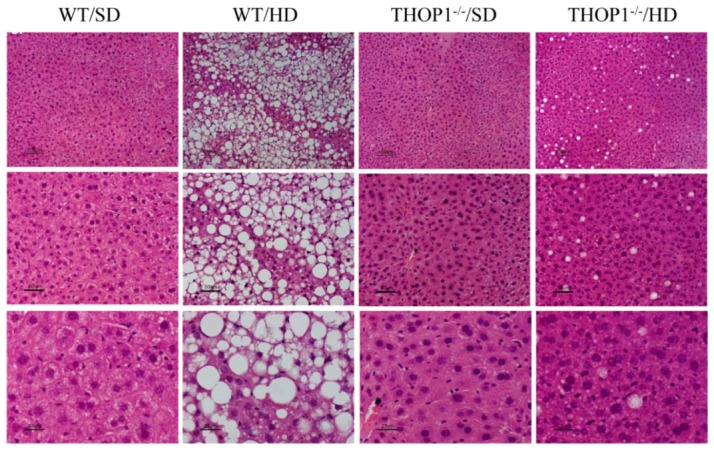
Liver slices from WT and THOP1**^−/−^** mice stained with hematoxylin and eosin (HE). WT or THOP1**^−/−^** male mice were fed for 24 weeks with either an SD (WT/SD, Table 1. or a HD (WT/HD, THOP1**^−/−^**/HD). Note the large number of lipid droplets (white spots) in the liver slices from WT mice fed a HD (WT/HD panels), suggesting the presence of NAFLS. Panels present increased magnifications from 40- (top), 100- (middle) or 200-fold (bottom). Bars, 100 μm.

Histological analyses were conducted to compare hematoxylin-eosin (HE)-stained liver slices that were obtained from both WT and THOP1**^−/−^** male mice fed either an SD or a HD. The HE-stained liver tissue slices suggested that WT male animals that were fed a HD, distinctly from the other groups analyzed, had large areas of lipid accumulation in their livers, resembling those with NAFLS (Figure 11).

### 3.6. Specific Genes and microRNAs were Differentially Expressed in the Liver and Adipose Tissue of WT and THOP1^−/−^ Mice, and are Unlikely to be Regulated by HD

The expression levels of specific genes were analyzed in these liver tissues. THOP1**^−/−^** mice that were fed an SD or a HD had reduced CD36/FAT, fatty acid translocase, also known as cluster of differentiation 36 (CD36) mRNA expression levels when compared to WT mice fed either an SD or a HD (Table 6). CD36 is present in humans and mice with NAFLS, also contributing to dyslipidemia that is associated with HD-induced obesity in C57BL6 mice [94]. Lower liver CD36 expression, together with the regular insulin sensitivity of THOP1**^−/−^** mice, could favor a reduction of NAFLS of THOP1**^−/−^** mice. THOP1**^−/−^** mice that were fed a HD also had reduced mRNA expression levels of peroxisome proliferator-activated receptor gamma (PPAR-γ) and fatty-acid synthase (FAS) when compared to WT mice fed a HD (Table 6). Increased hepatic expression of PPAR-γ and FAS also increases fat accumulation in the liver [95,96].

**Figure 12:**
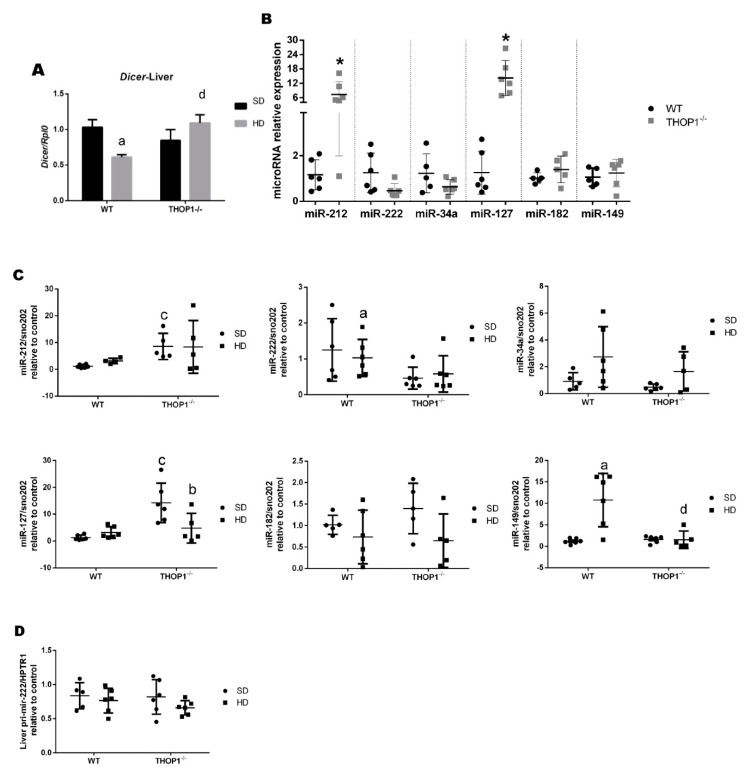
Dicer mRNA and microRNA expression levels in male liver tissue. (**A**) dicer mRNA expression levels. (**B**) Effect of phenotype on the expression levels of the indicated mature microRNAs; * *p* ≤ 0.05 comparing WT and THOP1^−/−^ mice. (**C**) Effect of diet and phenotype on the expression levels of the indicated mature microRNAs. (**D**) Effect of diet and phenotype on the expression level of pri-miR-222. Results are expressed as mean ± SEM. Statistical analyses were performed using Student’s unpaired *t*-test. One letter, *p* ≤ 0.05; a, WT/SD vs. WT/HD; b, THOP1**^−/−^**/SD vs. THOP1**^−/−^**/HD; c, WT/SD vs. THOP1**^−/−^**/SD; d, WT/HD vs. THOP1**^−/−^**/HD (*n* = 4–6).

Next, the expression levels of dicer and specific microRNAs were evaluated (Figure 12A). Dicer mRNA expression was reduced in WT mice that were fed with a HD as compared to an SD, whereas no differences were observed in dicer mRNA levels in THOP1**^−/−^** mice fed either an SD or a HD (Figure 12A). Dicer expression in THOP1**^−/−^** mice fed a HD was higher than in WT mice fed a HD (Figure 12A). THOP1**^−/−^** animals that were fed an SD had higher expression levels of miR-212 and miR-127 than WT mice fed an SD, while the expression levels of miR-222, miR-34a, miR-182, and miR-149 were similar (Figure 12B). A HD increased the expression levels of miR-222 and miR-149 in WT, but not THOP1**^−/−^** mice (Figure 12C). The expression levels of pri-miR-222 were not altered in these groups (Figure 12D). Therefore, the altered expression of specific genes and microRNAs corroborates the histological analyses, suggesting that THOP1**^−/−^** animals fed a HD do not develop NAFLS, as observed for WT mice fed a HD (Figure 11).

The expression levels of specific genes were analyzed in the inguinal adipose tissue of WT and THOP1**^−/−^** animals; the inguinal and retroperitoneal adipose tissues were chosen for gene and microRNA expression analyses due to large differences in their weight when comparing both WT and THOP1**^−/−^** animals (Table 2). WT males that were fed an SD had higher adipose tissue expression levels of lipoprotein lipase (LPL), CD36/SR-B2 (also known as cluster of differentiation 36, platelet glycoprotein 4, or fatty acid translocase/FAT), CD206 (also known as cluster of differentiation 206, C-type lectin mannose receptor), CD11C (also known as cluster of differentiation 11c or integrin alpha X), and murine macrophage F4/80 glycoprotein (F4/80) compared to THOP1**^−/−^** males that were fed an SD (Table 7). WT males fed a HD showed reduced expression levels of PPAR-γ, FAS, and LPL and increased expression levels of CD206, CD11C, and F4-80 as compared to WT males fed an SD (Table 7). THOP1**^−/−^** males fed a HD when compared to those fed an SD showed increases in the levels of LPL, CD36, CD206, and F4/80 expression (Table 7). Distinctly from that of WT males, THOP1**^−/−^** males showed increased levels of PPAR-γ, FAS, and CD36 expression when fed a HD as compared to an SD (Table 7). Similarly to WT, when THOP1**^−/−^** males were fed a HD, the levels of CD206 expression increased, although to lower levels (Table 7). Additionally, THOP1**^−/−^** mice fed a HD had increased levels of PPAR-γ, FAS, LPL, and F4/80 when compared to WT mice fed a HD (Table 7). The increased PPAR-γ expression in THOP1**^−/−^** as compared with WT mice could be associated with their higher levels of corticosterone at the beginning of the activity period, in addition to maintaining regular insulin sensitivity, also when compared to WT mice. Higher PPAR-γ expression could increase the adipogenesis in THOP1**^−/−^** mice.

**Figure 13:**
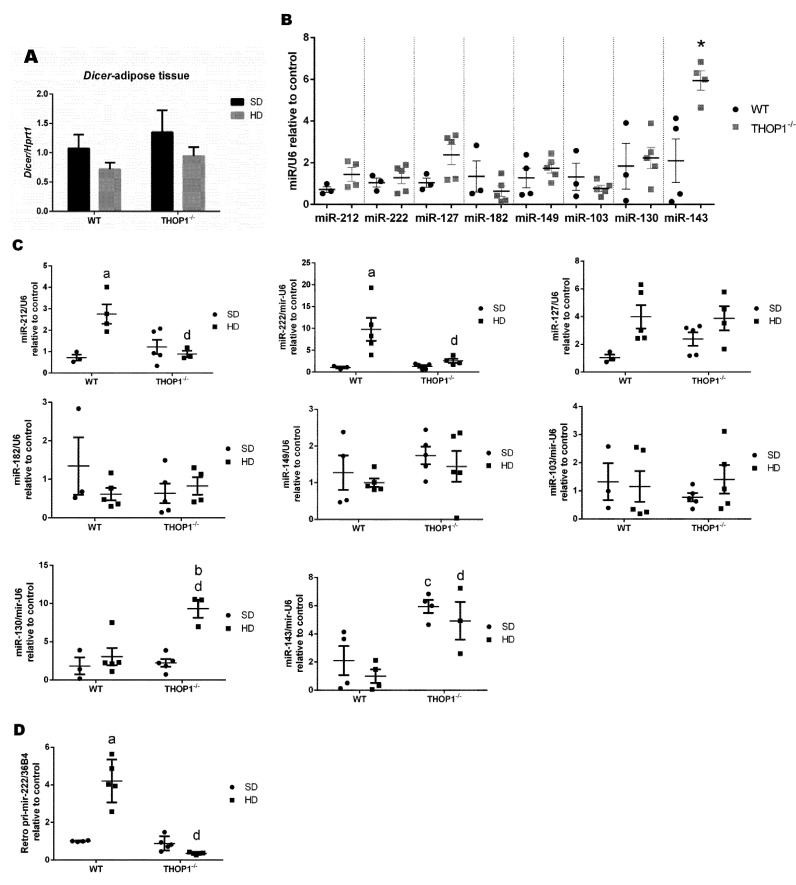
Dicer mRNA and microRNA expression levels in male retroperitoneal adipose tissue. (**A**) Dicer mRNA expression levels. (**B**) Effect of phenotype on the expression levels of the indicated microRNAs; * *p* ≤ 0.05 when comparing WT and THOP1^−/−^ mice. (**C**) Effect of diet and phenotype on the expression levels of the indicated microRNAs. (**D**) The effect of diet and phenotype on the expression level of pri-miR-222. Results are expressed as mean ± SEM. Statistical analyses were performed using Student’s unpaired *t*-test. One letter, *p* ≤ 0.05; a, WT/SD vs. WT/HD; b, THOP1**^−/−^**/SD vs. THOP1**^−/−^**/HD; c, WT/SD vs. THOP1**^−/−^**/SD; d, WT/HD vs. THOP1**^−/−^**/HD (*n* = 4–6).

Next, the dicer and specific microRNA expression levels were evaluated in male retroperitoneal adipose tissue (Figure 13). Dicer expression was similar between WT and THOP1**^−/−^** mice that were fed either an SD or a HD (Figure 13A). In THOP1**^−/−^** mice fed an SD, miR143 expression levels were higher than in WT mice fed an SD; all other analyzed microRNAs were similarly expressed for the SD (Figure 13B). The expression levels of miR-212 and miR-222 only increased in WT mice fed a HD (Figure 13C). In THOP1**^−/−^** mice fed a HD, only the expression levels of miR-130 were increased by the HD (Figure 13C). The expression levels of pri-miR-222 were drastically reduced in the retroperitoneal adipose tissue of THOP1**^−/−^** mice (Figure 13D). Altogether, the data above suggest that THOP1**^−/−^** mice have—at least to some degree—an altered C57BL6 gene and altered microRNA expression patterns in their liver and adipose tissues. 

### 3.7. DIO Further Modified the Relative Levels of Particular Intracellular Peptides in the Adipose Tissue of WT and THOP1^−/−^ Mice

**Figure 14:**
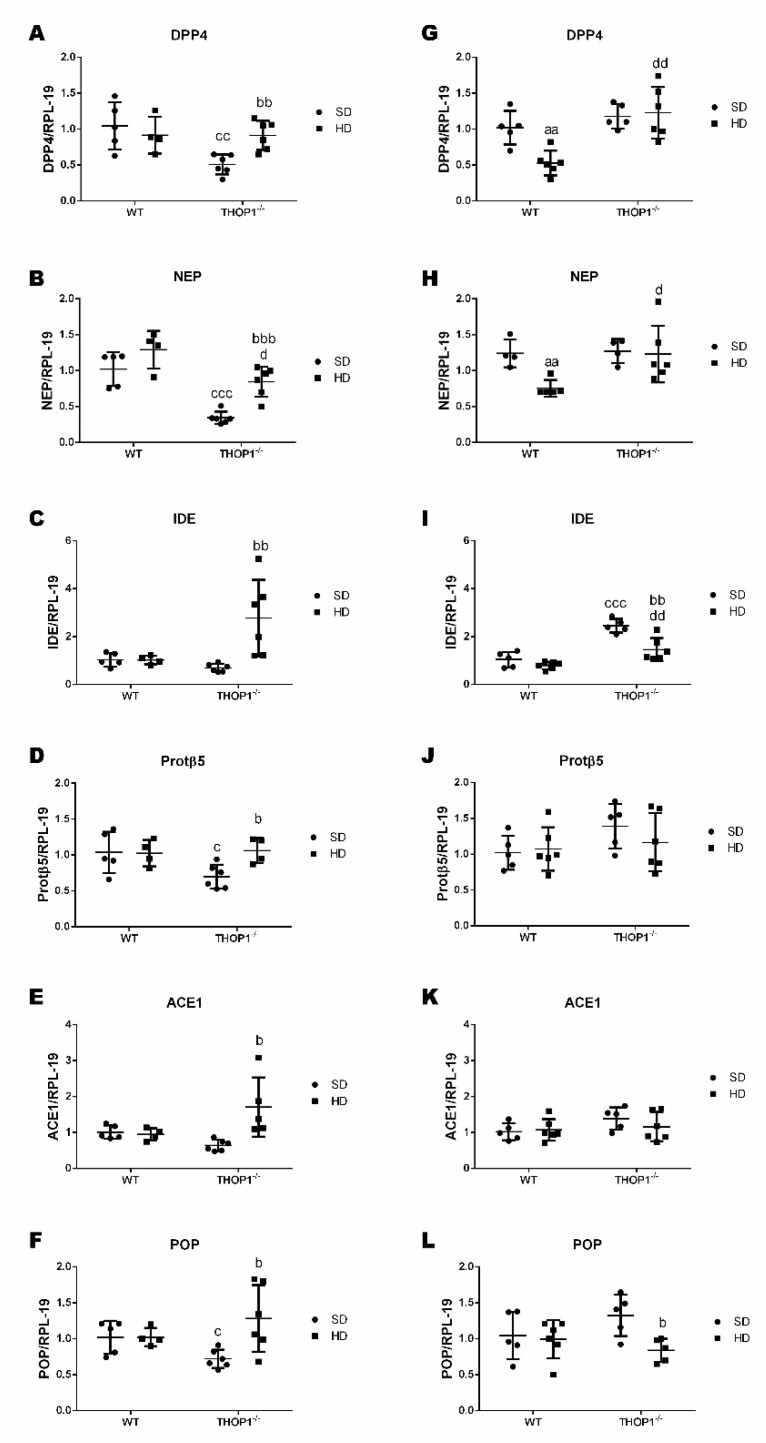
Gene expression of peptidases and proteasome beta5-subunit (Protβ5) in adipose tissue from WT and THOP1**^−/−^** mice. **A**–**F**, female; **G**–**L**, male. Analyses of gene expression were conducted by qRT-PCR in female and male retroperitoneal adipose tissue for specific peptidases, dipeptidyl peptidase 4 (DPP4), neprilysin (NEP), insulin-degrading enzyme (IDE), angiotensin converting enzyme 1 (ACE1), prolyl-oligopeptidase (POP), and Protβ5. Data are presented as mean ± standard deviation. Statistical analyses were performed using Student’s unpaired *t-*test. One letter, *p* ≤ 0.05; two letters, *p* ≤ 0.01; three letters *p* ≤ 0.001. a, WT/SD vs. WT/HD; b, THOP1**^−/−^**/SD vs. THOP1**^−/−^**/HD; c, WT/SD vs. THOP1**^−/−^**/SD; d, WT/HD vs. THOP1**^−/−^**/HD (*n* = 4–6).

Electron spray mass spectrometry and isotope labeling were combined [79,80,97] in order to identify and semi-quantify intracellular peptides in the adipose tissue of WT and THOP1**^−/−^** mice fed either an SD or a HD (Table 8). Inguinal adipose tissue was chosen because of its physiological relevance for DIO and also because of its higher weight and fat contents when comparing WT and THOP1**^−/−^** animals that were fed a HD (Table 2). Table 8 shows the amino acid sequences of the 17 identified peptides, their precursor proteins with respective predominant subcellular localization, and the average THOP1**^−/−^**/WT ratios of peptides identified in duplicates from at least three out of the five experimental replicates. These procedures were relevant for increasing data robustness, because the relative concentrations of intracellular peptides varied greatly between the different samples, as previously observed [98]. Most of the peptides identified herein (14 of 17) had altered relative ratios in THOP1**^−/−^** animals compared to WT animals fed an SD (Table 8, colored lanes). In animals that were fed a regular SD, the relative THOP1**^−/−^**/WT ratio of 13 peptides increased (possibly representing THOP1 substrates), whereas one peptide decreased (possibly representing a THOP1 product). These data suggest that THOP1**^−/−^** by itself affected the levels of intracellular peptides in the adipose tissue of C57BL6 mice. From the 13 peptides in which the relative levels were elevated in THOP1**^−/−^** mice as compared to WT mice, seven peptides remained increased, whereas six peptides decreased after these animals were fed a HD (Table 8). There were three peptides, in which their relative ratios were not altered for THOP1**^−/−^**/WT when fed an SD, but they decreased in THOP1**^−/−^** animals that were fed a HD (Table 8). Therefore, the HD reduced the levels of 10 out of the 17 intracellular peptides identified herein in the inguinal adipose tissue of both THOP1**^−/−^** and WT animals (Table 8). At least some of the intracellular peptide precursor proteins identified herein have well-known functions in cellular metabolism and/or obesity. Therefore, it is possible that changes in the expression levels of these precursor proteins have, at least in part, influenced the changes in intracellular peptides levels that are reported above. However, the variation in the profile of intracellular peptides could also be related to differential expression of proteasome and/or other intracellular peptidases. Indeed, dipeptidyl peptidase 4 (DPP4), neprilysin (NEP), and insulin degrading enzyme (IDE) expression levels were higher in THOP1**^−/−^** mice than in WT mice that were fed a HD for both males and females; in animals fed an SD, only the IDE mRNA expression levels were higher in THOP1**^−/−^** mice as compared to WT mice (Figure 14A–C,G–I). These latter results suggest a function for additional peptidases in adipose tissue intracellular peptide metabolism. No alterations in mRNA expression levels were observed for proteasome beta5-subunit (Protβ5) or for angiotensin converting enzyme 1 (ACE1), whereas prolyl-oligopeptidase (POP) was only slightly decreased under some conditions (Figure 14).

### 3.8. Molecular Modeling Suggests a Direct Interaction between Intracellular Peptides and Murine pre-miR-143 or pre-miR-222 

**Figure 15:**
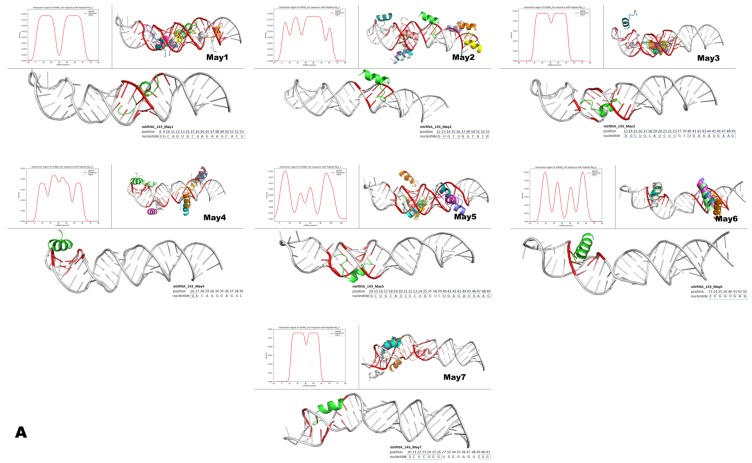

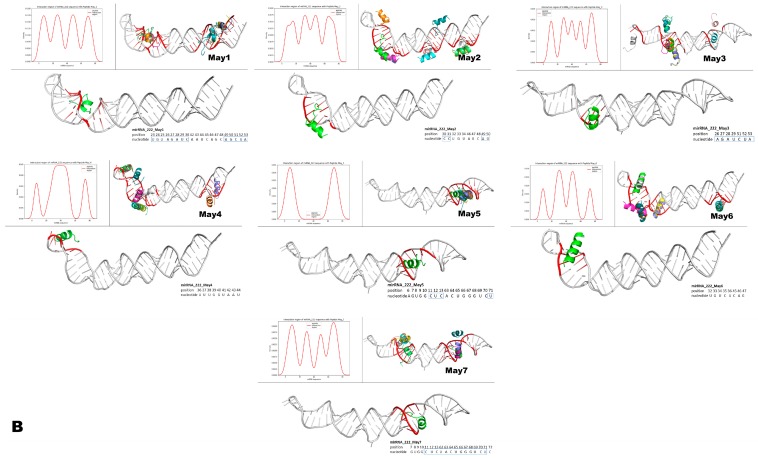
Structural modeling of murine May peptides interacting with either miR-143 or miR-222. Each panel contains seven small Figures that represent, from left to right, pre-miR-143 (A) or pre-miR-222 (B) interacting with the indicated intracellular peptide (May1–May7). The top left histogram panel from each small Figure represents the different densities/theoretical affinities (shown on the y-axis) of the top 10 structures predicted for each indicated May peptide. Shown on the x-axis of the histograms are the nucleotide positions from each microRNA that interacted with the predicted structures of May peptides. Note that intracellular peptides were predicted to interact with different regions and with different affinities along the nucleotide sequences of the respective pre-microRNAs. The top right panel on each small Figure shows all of the top ten predicted peptide structures interacting with their respective pre-microRNAs. The lower larger panel shows only one predicted structure of the corresponding May peptide, which was predicted to interact with the highest affinity to the indicated region of the respective pre-microRNA. In these large panels, inside the small blue boxes, are the nucleotides from the mature microRNAs predicted to interact with the peptide of the highest affinity. Note that intracellular peptides May1–May7 frequently interacted with a large portion (both at 5p and 3p) of the mature region of these miRNAs.

Specific genes and microRNAs involved in obesity and adipogenesis were differentially modulated in the liver and adipose tissue of THOP1**^−/−^** mice. In parallel, the profile of intracellular peptides was also modified in the adipose tissue of THOP1**^−/−^** mice. Thus, it was hypothesized that intracellular peptides could regulate gene expression by directly binding specific microRNAs. Intracellular peptides could affect the expression levels of microRNAs through binding and, consequently, the expression of specific genes that are related to obesity and energetic metabolism. One intracellular peptide (AQGGVLPNIQAVLLPK, coined “May1”), derived from histone H2A type 1-B, increased even more when the animals were fed a HD (Table 8). The relative ratio of six peptides (May2/May7) increased in THOP1**^−/−^**/WT mice that were fed an SD, whereas largely decreased after these animals were fed a HD (Table 8). The May1/May7 peptides were selected for molecular modeling to analyze their possible interaction with pre-miR-143 and pre-miR-222. The 10 most probable structures of the selected intracellular peptides (namely, May1 through May7) and their interaction with microRNAs were modeled because short peptides have dynamic structures in solution; all of these structures could, indeed, co-exist endogenously (Figure 15). These 10 structures were individually simulated interacting with either pre-miR-143 or pre-miR-222; each of these pre-microRNAs have only one predicted structure. All of the 10 most probable structures of the May1/May7 intracellular peptides interacted with murine pre-miR-143 and pre-miR-222, although within different regions and with different densities/theoretical affinities (Figure 15). The regions on the pre-miR-143 and pre-miR-222 found to interact with the May peptides were frequently identified on the corresponding mature microRNA region; five of the seven May peptides interacted within the mature region of pre-miR-143, and three of the seven peptides interacted within the mature region of pre-miR-222 (Figure 15). Thus, this demonstrates that the intracellular peptides likely interact with microRNAs, but the possible biological significance of this interaction still needs further investigations.

## 4. Discussion

Here, THOP1 was shown to play a key role in energy metabolism; THOP1**^−/−^** mice, distinctive from WT mice, were resistant to DIO and showed no insulin resistance after being fed a HD for 24 weeks. In THOP1**^−/−^** mice, the higher adrenergic-stimulated lipolytic activity seems seminal to the characterized phenotypes of these animals with reduced weight gain, no insulin resistance, and reduced liver and adipose tissue fat stores. Overall, we have, for the first time, successfully provided evidence that THOP1 could be a therapeutic target for controlling obesity and associated diseases, such as insulin resistance and NAFLS.

The predominant intracellular localization of THOP1 suggests that its main physiological significance should take place inside the cells. Corroborating this suggestion, THOP1 has been shown to play a key intracellular function in processing MHC-I antigens [2,26,27,45]. THOP1 has additional intracellular functions, as shown by siRNA inhibition of its expression, which was sufficient to potentiate intracellular beta-adrenergic signal transduction; conversely, when THOP1 was overexpressed, isoproterenol and angiotensin signal transduction was inhibited [99,100]. Herein, a broader characterization of THOP1 null mice uncovered its physiological relevance for the regulation of energy metabolism. Altogether, the data presented herein suggest that increased adipose tissue adrenergic-stimulated lipolysis could be seminal for the lack of DIO, NAFLS, and insulin resistance presented by THOP1**^−/−^** mice that were fed a HD. The proteasome is suggested to produce most of its substrates, which were coined intracellular peptides, because THOP1 is mainly located within cells. Intracellular peptides were differentially expressed in the adipose tissue of THOP1**^−/−^** mice and were suggested to mediate the phenotypic differences characterized herein, as follows: THOP1 controls the intracellular peptide profile, which then regulates protein interactions and the expression levels of microRNAs and, consequently, controls the expression levels of genes that regulate energy balance within adipose tissue. This possible mechanism is based on both the predominant intracellular location and the structural substrate-size restriction of THOP1. The ubiquitous existence of intracellular peptides and microRNAs in different cells of different species suggests a broader biological significance of the present results, whereby proteasome activity is connected to protein synthesis through a new route. Further investigations are still necessary to demonstrate the feasibility of these yet hypothetical mechanisms.

The molecular mechanism supporting the distinctive phenotypes of THOP1**^−/−^** mice possibly involves intracellular peptides [54]. Anorexigenic and orexigenic neuropeptides have not previously been characterized as THOP1 substrates [49] and, here, the levels of hormonal peptides related to obesity were shown to be unaffected in THOP1**^−/−^** animals. THOP1 is a predominantly intracellular protein [29,36], in support of it having a major function in intracellular peptide metabolism. Therefore, the extracellular activity of secreted or membrane-associated THOP1 should not play a major role in the phenotypic differences that are characterized in THOP1**^−/−^** mice. Previously, specific brain regions of THOP1**^−/−^** mice were shown to exhibit altered mRNA expression of proteasome beta5 subunit, serotonin 5HT2a receptor, and dopamine D2 receptor. Peptidomic analysis of specific brain regions identified differences mainly in the intracellular peptide ratios between THOP1**^−/−^** and WT, which were suggested to affect normal cellular functioning. In the present study, the observed changes in additional peptidases, such as DPP4, NEP, and IDE, could also contribute to the phenotypic differences described herein, as well as modulate intracellular peptides in the adipose tissue of THOP1**^−/−^** mice that were fed a HD. The differences in the profiles of intracellular peptides observed when THOP1**^−/−^** mice were a fed HD could also be related to alterations in the levels of intracellular peptide precursor proteins. Most of the intracellular peptide precursor proteins identified herein were previously shown to be functional in energy metabolism [101,102,103,104,105,106,107,108,109,110]. Several histone marks were identified in a prediabetic mouse model, providing a resource for studying the epigenetic functions of histone modifications in obesity and type 2 diabetes [109].The histone-derived intracellular peptides identified herein have putative post-translational lysine modification sites in their structure (indicated in bold underlined: AQGGVLPNIQAVLLP**K** and **K**QVHPDTGISSKAMGIMNS), suggesting a possible role of these peptides in regulating histone modifications in adipose tissue. Intracellular peptides containing putative post-translational modification sites were previously shown to modulate protein kinase and peptidase activities [10,70]. The histone-derived intracellular peptides that were identified to be differentially expressed in the brains of schizophrenic patients were also shown to be neuroprotective [14]. The expression of non-specific lipid-transfer protein, also known as sterol carrier protein (2SCP-2), significantly altered the association of several proteins (important to lipid droplet metabolism) with purified lipid droplets both in vitro as well as in intact cells [101]. Therefore, if intracellular peptides from 2SCP-2 could interfere with lipid composition and/or the association of proteins to adipose tissue lipid vesicles, energy metabolism, and fat deposition could be affected. Similarly, intracellular peptides from apolipoprotein A could play anti-obesity functions [110]. Acyl-CoA-binding protein (ACBP; also known as diazepam-binding inhibitor, DBI) is a lipogenic factor that triggers food intake and obesity [111]. In mice, systemic injection of ACBP protein inhibited autophagy, induced lipogenesis, reduced glycemia, and stimulated both appetite and weight gain. Thus, the neutralization of ACBP might constitute a strategy for treating obesity and its co-morbidities [111]. Indeed, altered levels of intracellular peptides that were derived from ACBP have been observed in the adipose tissue of Wistar rats fed a hypercaloric Western diet, and this was shown to improve glucose uptake in 3T3L1 adipocytes [66].Therefore, by regulating protein-protein interactions [112], intracellular peptides substrates and/or products of THOP1 could affect the DIO phenotype.

The intracellular peptides May1–May7, identified herein, were suggested to directly interact with microRNAs miR-143 and miR-222 while using molecular modeling. Therefore, these intracellular peptides could plausibly regulate microRNA functions by, at the very least, interfering with their processing and/or their endogenous stability [76,112] Differences in the expression levels of microRNAs could contribute to balancing the expression of genes shown herein to be differentially modulated in THOP1**^−/−^** mice that were fed a HD. MicroRNAs typically repress gene expression by binding to the 3’ UTR of mRNA, leading to its degradation [113]. The intracellular peptides identified in the present research were predicted to interact with different regions of the microRNAs miR-143 and miR-222, including the eight-base seed region [113]. By binding to the seed region of miR-143 and miR-222, intracellular peptides could prevent/favor microRNAs binding to the 3’ UTR of their target mRNAs to regulate gene expression. If these are possible mechanisms through which intracellular peptides operate, they could modulate gene expression to influence the phenotypic differences that are characterized between WT and THOP1**^−/−^** mice (i.e., great resistance to DIO, lack of insulin resistance, and absence of large areas of NAFLS in the liver tissue of THOP1**^−/−^** animals). However, further investigations should be conducted to elucidate the mechanisms through which THOP1 and intracellular peptides operate in regulating energy metabolism.

The differential expression of specific genes in the liver and/or in adipose tissue, including CD11c, PPAR-γ, LPL, F4/80, and FAS, corroborate the greater resistance of THOP1**^−/−^** mice to DIO and insulin resistance. The enhanced expression of adrenergic receptors should also contribute to isoproterenol-stimulated enhancement of lipolytic activity of adipose tissue in THOP1**^−/−^** animals, particularly in males. Previous studies have shown that intracellular peptides reintroduced into HEK293 and CHO cells potentiate isoproterenol-stimulated beta adrenergic signaling [99]. The inhibition of THOP1 in HEK293 cells while using siRNA was also shown to increase isoproterenol response through protein kinase A [100]. THOP1**^−/−^** males showed decreased blood glucose levels under food restriction, which was prevented by the beta-adrenergic antagonist propranolol. Altogether, these data corroborate previous assertions [99,100] that THOP1 plays a previously anticipated role in the beta-adrenergic signaling pathway. The blood pressure and heart rate of WT and THOP1**^−/−^** mice were similar, which suggested that THOP1 has tissue-specific adrenergic-enhanced properties. The apparent contradictory parameters of THOP1**^−/−^** animals that exhibit higher lipolysis and lower inflammation in adipose tissue could be compensated by the higher PPAR-γ expression observed herein, which could inhibit the expression of nuclear factor kappa-light-chain-enhancer of activated B cells [114]. In addition, higher transient levels of corticosterone at the beginning of the night period could benefit THOP1**^−/−^** animals by reducing the likelihood of adipose tissue inflammation. 

The resting energy metabolism of WT and THOP1**^−/−^** mice was similar across the 24 h period, while some differences were observed after the beginning of the mice’s activity period (~5–10 p.m.). Therefore, changes in resting energy metabolism were probably not important for explaining the differences in weight gain of THOP1**^−/−^** mice. The higher levels of corticosterone that were observed in male THOP1**^−/−^** mice correlate with the shift observed in the resting energy metabolism during the interval from 5 to 10 p.m. The circadian rhythm has previously been shown to influence THOP1 enzyme activity (formerly endopeptidase 22.19; EC 3.4.22.19) in the whole brain and in individual areas, such as cerebellum, striatum, hypothalamus, and periaqueductal gray matter [115]. These data could suggest that the expression levels of the genes that regulate circadian rhythms were also affected in these animals, because it is well known that circadian rhythm genes are closely related to energy metabolism [116,117,118]. A higher expression of THOP1 was observed in the adipose tissue of WT females when compared to WT males, fed either SD or HD. Previous studies have shown that, after exposure to a high-fat diet for 12 weeks, females gained less weight than males, and that ovarian hormones were partially responsible for these differences [119]. Similarly, at least some of the differences in the weight gain and metabolic parameters observed herein were more evident in THOP**^−/−^** males than females. Future experiments will be still necessary to evaluate the importance of THOP1 to DIO sexual dimorphism.

## 5. Conclusions

We demonstrate, for the first time, that THOP1 plays various key functions in energy metabolism. Intracellular peptides were suggested to mediate the phenotypic differences that are characterized herein between WT and THOP1**^−/−^** mice. These data also provide novel alternative targets for the pharmacological treatment of obesity and obesity-associated diseases.

## Figures and Tables

**Table 1:** Delta (Δ) of body weight gained from the 4th–24th week of mice fed a standard diet (SD) or a hyperlipidic diet (HD) diet.

Gender/phenotype	Δ(g) SD	Δ(g) HD	Δ(g) HD − Δ(g) SD
WT females	9.7	16.6	6.9 (100%)
THOP1^−/−^ females	8.0	11.9	3.9 (56%)
WT males	12.3	26.5	14.2 (100%)
THOP1^−/−^ males	15.0	18.8	3.8 (27%)

In parentheses are the percentages of the weight gained by wild-type (WT) and THOP**^−/−^** mice, on average, across the 24 weeks of the HD compared to the SD. Note that both female and male THOP1**^−/−^** mice gained less body weight than the WT mice during 24 weeks of the HD (*n* = 6–9).

**Table 2:** Biological parameters measured during the 24 weeks of the HD and standard diet (SD) diets.

	**Female**			
**Parameters**	**WT/SD**	**WT/HD**	**THOP1^−/−^/SD**	**THOP1^−/−^/HD**
Body weight (g)	24.7 ± 0.42	29.3 ± 1.72 ^a^	22.3 ± 0.2 ^ccc^	24.8 ± 0.59 ^bb;d^
Food intake (g/day/animal)	3.55 ± 0.15	2.25 ± 0.05 ^a^	3.2 ± 0.1	1.9 ± 0.1 ^bb^
Water intake (mL/day/animal)	4.05 ± 0.05	2.35 ± 0.3 ^a^	3.5 ± 0.1^c^	2.55 ± 0.2 ^b^
Caloric intake (kcal/day/animal)	13.5 ± 0,6	12.05 ± 0.3	11.75 ± 0.05	10.35 ± 0.05
Lee Index	39.0 ± 0.43	43.3 ± 1.70 ^a^	37.1 ± 0.26 ^cc^	39.3 ± 0.52 ^bb;d^
Liver (g)	3.53 ± 0.11	2.56 ± 0.06 ^aaa^	3.46 ± 0.09	3.01 ± 0.07 ^bb;ddd^
Retroperitoneal adipose tissue (g)	0.73 ± 0.10	4.22 ± 0.25 ^aaa^	0.82 ± 0.05	2.00 ± 0.33 ^bb;ddd^
Inguinal adipose tissue (g)	1.12 ± 0.11	4.63 ± 0.28 ^aaa^	1.49 ± 0.09	2.42 ± 0.26 ^bb;ddd^
Ovarian adipose tissue (g)	1.87 ± 0.12	6.28 ± 0.35 ^aaa^	1.99 ± 0.20	3.59 ± 0.42 ^bb;ddd^
Soleus (g)	0.06 ± 0.003	0.05 ± 0.003 ^a^	0.07 ± 0.002	0.06 ± 0.001 ^bb;ddd^
Gastrocnemius (g)	1.09 ± 0.02	0.75 ± 0.02 ^aaa^	1.00 ± 0.03	0.83 ± 0.12
	**Male**			
**Parameters**	**WT/SD**	**WT/HD**	**THOP1^−/−^/SD**	**THOP1^−/−^/HD**
Body weight (g)	27.7 ± 0.28	42.0 ± 0.8 ^aaa^	30.1 ± 0.5 ^cc^	32.6 ± 0.74 ^b;ddd^
Food intake (g/day/animal)	3.5 ± 0.2	2.5 ± 0.2	3.4 ± 0.1	2.5 ± 0.2
Water intake (mL/day/animal)	3.65 ± 0.05	2.55 ± 0.6 ^bb^	3.45 ± 0.05	2.65 ± 0.5
Caloric intake (kcal/day/animal)	13.25 ± 0.7	13.45 ± 1.0	12.8 ± 0.3	13.8 ± 0.1
Lee index	42.2 ± 0.24	55.8 ± 0.82 ^aaa^	44.2 ± 0.48 ^cc^	46.5 ± 0.70 ^b;ddd^
Liver (g)	3.77 ± 0.07	4.60 ± 0.25 ^aa^	3.87 ± 0.10	3.09 ± 0.08 ^bbb;ddd^
Retroperitoneal adipose tissue (g)	0.94 ± 0.13	3.11 ± 0.19 ^aaa^	0.44 ± 0.05 ^cc^	1.03 ± 0.18 ^bb;ddd^
Inguinal adipose tissue (g)	1.35 ± 0.06	4.83 ± 0.19 ^aaa^	0.92 ± 0.04 ^ccc^	1.40 ± 0.17 ^b;ddd^
Epididymal adipose tissue (g)	1.87 ± 0.11	3.07 ± 0.23 ^aaa^	1.41 ± 0.13 ^c^	2.58 ± 0.36 ^bb^
Soleus (g)	0.06 ± 0.001	0.05 ± 0.002 ^aaa^	0.07 ± 0.003	0.05 ± 0.001 ^bb;d^
Gastrocnemius (g)	1.07 ± 0.02	0.72 ± 0.01 ^aaa^	1.06 ± 0.01	0.99 ± 0.02 ^b;ddd^

Results are expressed as mean ± SEM. Student’s unpaired *t*-test. One letter, *p* ≤ 0.05; two letters, *p* ≤ 0.01; three letters, *p* ≤ 0.001. a, WT/SD vs. WT/HD; b, THOP1**^−/−^**/SD vs. THOP1**^−/−^**/HD; c, WT/SD vs. THOP1**^−/−^**/SD; d, WT/HD vs. THOP1**^−/−^**/HD (*n* = 6–9).

**Table 3:** Systolic blood pressure (mmHg) and heart rate (bpm).

	Systolic Blood Pressure (mmHg)		Heart Rate (bpm)	
	20th Week	24th Week	20th Week	24th Week
**WT/SD**	101.1 ± 2.91	95.99 ± 0.86	748.1 ± 12.50	766.5 ± 8.88
**WT/HD**	105.1 ± 4.42	97.19 ± 2.32	751.3 ± 7.58	761.7 ± 9.85
**THOP1^−/−^/SD**	111.3 ± 1.82	107.5 ± 3.15	724.8 ± 6.67	708.5 ± 37.28
**THOP1^−/−^/HD**	99.87 ± 6.55	102.0 ± 7.79	723.9 ± 13.82	695.6 ± 64.86

Results are expressed as mean ± SEM. Statistical analyses were conducted using. Student’s unpaired t-test, suggesting no differences between groups (n = 6–9).

**Table 4:** Biochemical parameters of WT and THOP1**^−/−^** mice fed an SD or a HD.

	**Female**			
**Parameters**	**WT/SD**	**WT/HD**	**THOP1^−/−^/SD**	**THOP1^−/−^/HD**
Total cholesterol	118.5 ± 6.9	151.4 ± 9.9 ^a^	115.9 ± 6.8	143.9 ± 16.4
Triglycerides	111.5 ±13.0	134.4 ± 17.4	132.1 ± 12.5	101.5 ± 8.6
HDL	58.5 ± 18.9	95.9 ± 18.0	30.6 ± 3.0	46.4 ± 11.0 ^d^
	**Male**			
**Parameters**	**WT/SD**	**WT/HD**	**THOP1^−/−^/SD**	**THOP1^−/−^/HD**
Total cholesterol	109.0 ± 5.7	235.8 ± 8.7 ^aaa^	132.8 ± 11.85	139.4 ± 7.2 ^ddd^
Triglycerides	115.7 ± 11.7	100.4 ± 1.7	151.5 ± 8.2 ^c^	126.4 ± 5.8 ^b,d^
HDL	68.3 ± 3.8	61.3 ± 0.2	63.9 ± 3.8	68.9 ± 2.8

Results are expressed as mean ± SEM. Statistical analyses were conducted using Student’s unpaired *t*-test. One letter, *p* ≤ 0.05; two letters, *p* ≤ 0.01; three letters *p* ≤ 0.001. a, WT/SD vs. WT/HD; b, THOP1**^−/−^**/SD vs. THOP1**^−/−^**/HD; c, WT/SD vs. THOP1**^−/−^**/SD; d, WT/HD vs. THOP1**^−/−^**/HD (*n* = 4–9).

**Table 5:** Multiplex immunoassay in serum samples from WT and THOP1**^−/−^** mice fed an SD or a HD.

	**Female**			
**Hormones**	**WT/SD**	**WT/HD**	**THOP1^−/−^/SD**	**THOP1^−/−^/HD**
Insulin (pg/mL)	120.8 ± 7.5	459.9 ± 75.8 ^aaa^	95.10 ± 16.5	106.5 ± 17.8 ^dd^
Resistin (pg/mL)	3756 ± 536.2	10458 ± 1648 ^aa^	3957 ± 300.4	4845 ± 624.4 ^dd^
Ghrelin (pg/mL)	905.6 ± 105.3	485.9 ± 55.0^a^	710.8 ± 221.6	713.8 ± 211.8
	**Male**			
**Hormones**	**WT/SD**	**WT/HD**	**THOP1^−/−^/SD**	**THOP1^−/−^/HD**
Insulin (pg/mL)	223.0 ± 39.0	1901 ± 255.0 ^aaa^	148.0 ± 40.7	235.9 ±103.2 ^ddd^
Resistin (pg/mL)	2437 ± 140.1	8588 ± 506.0 ^aaa^	2837 ± 223.3	3417 ± 223.3 ^ddd^
Ghrelin (pg/mL)	552.2 ± 36.24	197.8 ± 29.4 ^aaa^	604.4 ± 88.9	701.7 ± 54.4 ^ddd^

Animals were fasted for 10 h prior to blood collection. Results are expressed as mean ± SEM. Statistical analyses were conducted using Student’s unpaired *t*-test. One letter, *p* ≤ 0.05; two letters, *p* ≤ 0.01; three letters *p* ≤ 0.001. a, WT/SD vs. WT/HD; b, THOP1**^−/−^**/SD vs. THOP1**^−/−^**/HD; c, WT/SD vs. THOP1**^−/−^**/SD; d, WT/HD vs. THOP1**^−/−^**/HD (*n* = 5–9).

**Table 6:** Analyses of gene expression by qRT-PCR in male mice livers.

	Male			
Gene	WT/SD	WT/HD	THOP1^−/−^/SD	THOP1^−/−^/HD
PPAR-α	1.13 ± 0.24	1.15 ± 0.27	1.11 ± 0.10	0.99 ± 0.18
PPAR-γ	1.11 ± 0.24	1.58 ± 0.18	0.85 ± 0.06	0.70 ± 0.04 ^dd^
PGC-1α	1.10 ± 0.23	0.70 ± 0.09	1.01 ± 0.10	1.53 ± 0.37
FAS	1.19 ± 0.33	1.55 ± 0.28	1.00 ± 0.05	0.74 ± 0.10 ^d^
LPL	1.08 ± 0.17	1.28 ± 0.15	1.31 ± 0.16	1.52 ± 0.20
CD36	1.18 ± 0.31	1.87 ± 0.15	0.43 ± 0.05 ^c^	0.27 ± 0.01 ^b;ddd^

Results are expressed as mean ± SEM. Statistical analyses were conducted using Student’s unpaired *t*-test. One letter, *p* ≤ 0.05; two letters, *p* ≤ 0.01; three letters *p* ≤ 0.001 between: a, WT/SD vs. WT/HD; b, THOP1**^−/−^**/SD vs. THOP1**^−/−^**/HD; c, WT/SD vs. THOP1**^−/−^**/SD; d, WT/HD vs. THOP1**^−/−^**/HD (*n* = 5–6).

**Table 7:** Analyses of gene expression by qRT-PCR in inguinal adipose tissue.

	**FEMALE**			
**Gene**	**WT/SD**	**WT/HD**	**THOP1^−/−^/SD**	**THOP1^−/−^/HD**
PPAR-α	1.22 ± 0.46	4.02 ± 1.02^a^	1.03 ± 0.14	4.45 ± 1.17^b^
PPAR-γ	1.01 ± 0.06	0.68 ± 0.05^aa^	0.37 ± 0.13^cc^	0.57 ± 0.17
FAS	1.23 ± 0.47	0.78 ± 0.28	0.56 ± 0.12	1.01 ± 0.58
LPL	1.01 ± 0.08	0.68 ± 0.13	0.21 ± 0.07^ccc^	0.59 ± 0.18
CD36	1.01 ± 0.08	0.85 ± 0.10	0.23 ± 0.07^ccc^	0.67 ± 0.13^b^
CD206	1.03 ± 0.12	1.26 ± 0.17	0.40 ± 0.08^cc^	0.82 ± 0.21
CD11C	1.01 ± 0.08	8.36 ± 2.10^aa^	2.48 ± 0.53^c^	1.45 ± 0.45^d^
F4/80	1.02 ± 0.10	2.86 ± 0.54^a^	0.59 ± 0.09^c^	1.35 ± 0.43
	**MALE**			
**Gene**	**WT/SD**	**WT/HD**	**THOP1^−/−^/SD**	**THOP1^−/−^/HD**
PPAR-α	1.00 ± 0.04	1.51 ± 0.24	0.99 ± 0.27	1.67 ± 0.65
PPAR-γ	1.03 ± 0.10	0.51 ± 0.02^aa^	0.96 ± 0.08	1.10 ± 0.07^ddd^
FAS	1.05 ± 0.14	0.27 ± 0.03^aaa^	0.78 ± 0.15	1.06 ± 0.16^ddd^
LPL	1.08 ± 0.16	0.58 ± 0.09^a^	0.47 ± 0.07^cc^	0.90 ± 0.10^bb; d^
CD36	1.05 ± 0.12	0.77 ± 0.06	0.59 ± 0.04^cc^	0.96 ± 0.04^bbb; d^
CD206	1.01 ± 0.07	1.28 ± 0.06^a^	0.36 ± 0.06^cc^	0.85 ± 0.08^bb; d^
CD11C	1.02 ± 0.08	6.48 ± 0.47^aaa^	0.34 ± 0.08^ccc^	0.27 ± 0.03^ddd^
F4/80	1.14 ± 0.31	2.16 ± 0.14^a^	0.30 ± 0.04^c^	0.47 ± 0.03^b; ddd^

Results are expressed as mean ± SEM. Statistical analyses were conducted using Student’s unpaired *t*-test. One letter, *p* ≤ 0.05; two letters, *p* ≤ 0.01; three letters *p* ≤ 0.001 between: a, WT/SD vs. WT/HD; b, THOP1**^−/−^**/SD vs. THOP1**^−/−^**/HD; c, WT/SD vs. THOP1**^−/−^**/SD; d, WT/HD vs. THOP1**^−/−^**/HD (*n* = 5–6).

**Table 8:** Intracellular peptides semi-quantitatively identified in inguinal adipose tissue of WT or THOP1^−/−^ mice.

Precursor Protein Name	Peptide Sequence	Subcellular Localization	Ratio THOP1^−/−^/SD to WT/SD ± SEM (*n*)	Ratio THOP1^−/−^/HD to WT/HD ± SEM (*n*)
Macrophage migration inhibitory factor	LSELTQQLAQATGKPAQ	C	13.35 ± 3.5 (3)	12.16 ± 3.9 (3)
Histone H2A type 1-B	AQGGVLPNIQAVLLPK (*May1) ^IP^* *^pH 10^*	N	9.75 ± 4.7 (5)	19.58 ± 8.14 (5)
Histone H2B type 2-B	KQVHPDTGISSKAMGIMNS	N	20.59 ± 4.3 (4)	16.65 ± 2.6 (4)
Elongation factor 2	ASVLTAQPRLMEPI	C	4.35 ± 0.8 (3)	7.74 ± 0.8 (3)
Creatine kinase M-type	DISNADRLGSSEVEQ	C	8.17 ± 2.1 (3)	15.66 ± 7.9 (3)
Creatine kinase M-type	DISNADRLGSSEVEQV	C	11.21 ± 3.8 (3)	12.32 ± 3.5 (3)
Creatine kinase M-type	IDDHFLFDKPVSPLL	C	14.31 ± 5.0 (4)	6.60 ± 1.7 (4)
Apolipoprotein A-I	LETLKTQVQSVIDKA (*May6*) *^IP pH 6.9^*	SP	4.25 ± 1.1 (4)	0.42 ± 0.07 (4)
Apolipoprotein A-II	FSSLMNLEEKPAPAA (*May3*) *^IP pH 4.2^*	SP	10.59 ± 6.3 (5)	0.27 ± 0.04 (5)
Apolipoprotein A-II	HEQLTPLVRSAGTSLVN (*May5*) *^IP pH 7.6^*	SP	5.07 ± 1.3 (4)	0.20 ± 0.03 (4)
Serum albumin	SQTFPNADFAEITKL (*May7*) *^IP pH 3.9^*	SP	12.01 ± 1.6 (4)	0.38 ± 0.04 (4)
Non-specific lipid-transfer protein	ADSDLLALMTGKMNPQSA (*May2*) *^IP pH 3.7^*	P	6.7 ± 1.8 (3)	0.49 ± 0.08 (3)
Hemoglobin subunit alpha	GAEALERMFASFPTTK (*May4*) *^IP pH 7.0^*	C	6.33 ± 4.4 (3)	0.18 ± 0.05 (3)
Hemoglobin subunit alpha	FDVSHGSAQVK	C	0.43 ± 0.06 (3)	0.29 ± 0.06 (3)
Hemoglobin subunit alpha	IGGHGAEYGAEALER	C	0.58 ± 0.1 (4)	0.23 ± 0.05 (4)
Hemoglobin subunit alpha	SVSTVLTSK	C	0.66 ± 0.1 (3)	0.25 ± 0.03 (3)
Acyl-CoA-binding protein	QATVGDVNTDRPGLLDLKGK	C	0.71 ± 0.1 (3)	0.19 ± 0.04 (3)

Numbers in the colored columns indicate the mean value ± standard error of the mean (SEM; no statistical analyses were performed) of each peptide semi-quantitatively identified by *Mascot* automatically after LC–MS/MS data analyses. Five biological replicates were (*n* = 5) in duplicates (technical replicates, forward and reverse labeling) with each sample labeled with a different isotope. Note that only peptides founded in 3 or more biological replicates (shown in parenthesis) and in both technical replicates (forward and reverse labeling) are listed. Isoelectric points (*IP*) of May peptides are shown; note that IP of May peptides varied from acidic (May2, May3, and May7), through neutral (May6 and May4), to basic (May1 and May5), indicating different net charges in physiological ~pH 7.4 conditions. C, cytosol; N, nucleus; SP, secretory pathway; P, peroxisome. Red labels are peptides that increased >2.0; green labels are peptides that decreased <0.5. White unlabeled peptides had relative concentrations between 0.5 and 2.0. Additional details are shown in Experimental procedures. Data are available via ProteomeXchange with identifier PXD016265.

## Data Availability

The mass spectrometry proteomics data were deposited to the ProteomeXchange Consortium via the PRIDE [82] partner repository with the dataset identifier PXD016265 and 10.6019/PXD016265 (“Intracellular peptides are linked to obesity and obesity-associated diseases”).

## Supplementary Materials

The following are available online at https://www.mdpi.com/2218-273X/10/2/321/s1, Figure S1: Quantification of lipid content in animal feces; Figure S2. Pre-prandial insulin levels of female; Figure S3. Western blots show immunoreactivity for THOP1 and beta-actin on adipose (A) and liver (B) tissue homogenates from WT animals; Figure S4. Adiponectin levels of female (A) and male (B) mice were evaluated after 10 h of food restriction (pre-prandial); Figure S5. Resting energy metabolism of WT (black lanes) or THOP1^−/−^ (red lanes) male mice across 24 h; Figure S6. Schematic representation to illustrate the semi-quantitative peptidomic analyses. Tables S1: Primers sequences; Table S2. Schematic distribution of “May” peptidomic samples; Table S3. Complete data analyses for the identified intracellular peptides. Additional Spectrometry Supporting Information.
