# Supplementary material for: The Relevance of Thimet Oligopeptidase in the Regulation of Energy Metabolism and Diet-Induced Obesity
Source: Biomolecules. 2020 Feb 17;10(2):321. doi: 10.3390/biom10020321 (PMC7072564; doi:10.3390/biom10020321)

Table 1. Intracellular peptides identified on inguinal adipose tissue of WT or THOP1$^{-/-}$

Precursor Protein name	Peptide sequence	RT	L1_m/z	L2_m/z	I_m/z	H_m/z	z	T	Avg. mass	SD	Obs. mass	Theor. mass	ppm
Macrophage migration inhib. factor	LSELTQQLAQATGKPAQ	64,7	920,5638	922,558	924,5377	928,5586	2	2	1783,0083	0,045	1783,008	1782,9549	29,9
Histone H2A type 1-B	AQGGVLPNIQAVLLPK	96,9	837,5191	839,5303	841,5442	845,5635	2	2	1616,9778	0,024	1616,978	1616,9687	5,6
Histone H2B type 2-B	KQVHPDTGISSKAMGImNS	37,6	701,0305	703,054	705,0552	709,0762	3	3	2016,0107	0,041	2016,011	2015,9792	15,6
Elongation factor 2	ASVLTAQPRLMEPI	80,1	777,4376	778,4376	779,4517	781,4618	2	1	1524,836	0,014	1524,836	1524,8407	-3,12
Creatine kinase M-type	DISNADRLGSSEVEQ	49,1	824,4038	825,4003	826,4043	828,4151	2	1	1618,7534	0,011	1618,753	1618,66	57,7
Creatine kinase M-type	DISNADRLGSSEVEQV	59,5	873,929	874,9346	875,9405	877,9507	2	1	1717,819	0,011	1717,819	1717,8191	-0,06
Creatine kinase M-type	IDDHFLFDKPVSPLL	99,3	906,5032	908,5107	910,5258	914,5468	2	2	1754,9425	0,022	1754,942	1754,9316	6,18
Apolipoprotein A-I	LETLKTQVQSVIDKA	73,2	586,3524	588,364	590,3767	594,3958	3	3	1671,9655	0,036	1671,965	1671,948	10,5
Apolipoprotein A-II	FSSLMNLEEKPAPAA	71,5	830,9321	832,9461	834,9584	838,9783	2	2	1603,8067	0,024	1603,807	1603,7989	4,83
Apolipoprotein A-II	HEQLTPLVRSAGTSLVN	60	925,5099	926,5137	927,5212	929,5321	2	1	1820,9801	0,011	1820,98	1820,9817	-0,91
Serum albumin	SQTFPNADFAEITKL	93,2	869,46	871,4683	873,4811	877,4998	2	2	1680,8538	0,023	1680,854	1680,8432	6,31
Non-specific lipid-transfer protein	ADSDLLALMTGKMNPQSA	99	959,9837	961,9929	964,0899	968,0289	2	2	1861,9469	0,103	1861,947	1861,8987	25,9
Hemoglobin subunit alpha	GAEALERMFASFPTTK	87,8	906,5109	908,4832	910,4923	914,5168	2	2	1754,9008	0,033	1754,901	1754,834	38,1
Hemoglobin subunit alpha	FDVSHGSAQVK	24,6	615,8264	617,8394	619,8522	623,8708	2	2	1173,5936	0,025	1173,594	1173,5851	7,24
Hemoglobin subunit alpha	IGGHGAEYGAEALER	39,5	779,3855	780,3916	781,3956	783,4088	2	1	1528,7324	0,009	1528,732	1528,7343	-1,28
Hemoglobin subunit alpha	SVSTVLTSK	35,3	489,298	491,3106	493,3228	497,342	2	2	920,5359	0,024	920,5359	920,5251	11,7
Acyl-CoA-binding protein	QATVGDVNTDRPGLLDLKGK	58,5	727,7425	729,7576	731,7711	735,7884	3	3	2096,1435	0,040	2096,144	2096,1299	6,49

RT= retention time; m= metionine oxidized; L1 or2 =Light; I=Intermediary; H=Heavy; z=charge; T= number of tags; SD= standard deviation; Avg=Average mass; Obs=Observed; theor.=theoric

Mascot Search Results

Peptide View

MS/MS Fragmentation of **LSELTQQLAQATGKPAQ**
Found in **MIF_MOUSE** in **SwissProt**, Macrophage migration inhibitory factor OS=Mus musculus GN=Mif PE=1 SV=2

Match to Query 14599: 1847.062548 from(924.538550,2+) intensity(2914775.6000) scans(14738-14751) rawscans(sn14738:sn14751) rtinseconds(3887.7387-3890.5887) index(9352)
Title: 9353: Sum of 2 scans in range 14738 (rt=3887.74) to 14751 (rt=3890.59) [D:\Users\Mayara - HMS Fusion data\170711PESSAM02977_May1.raw]
Data file 170711PESSAM02977_May1.temp.mgf

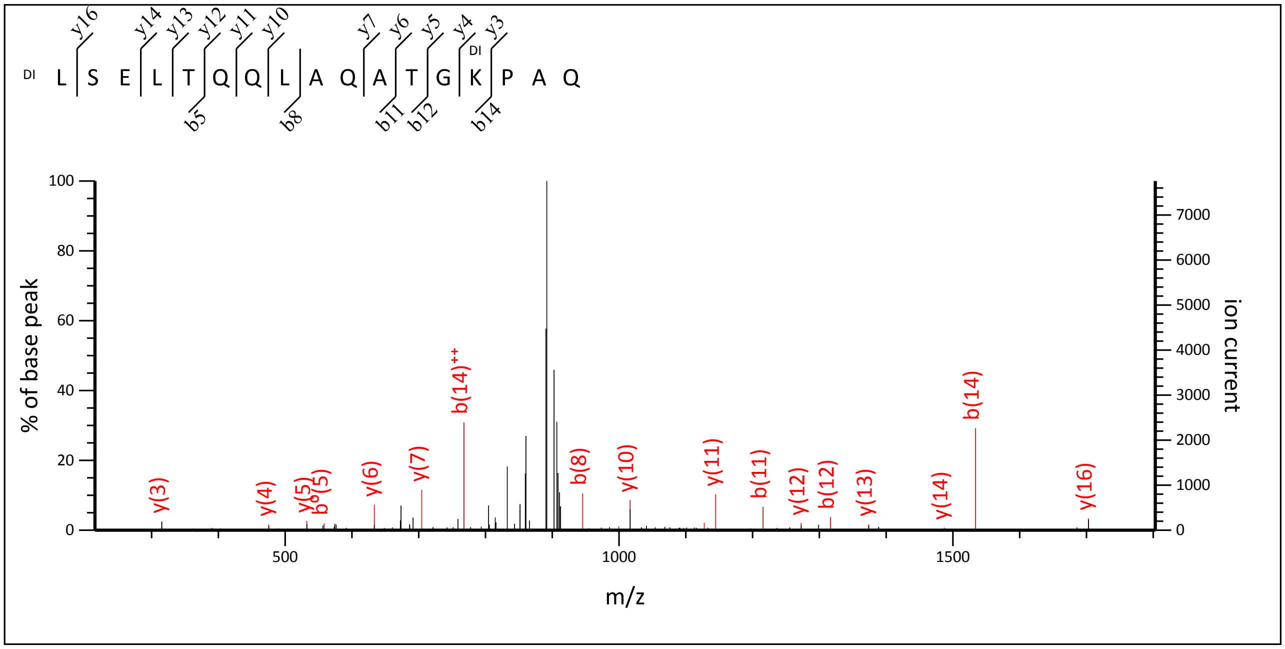

215.18 to 1803.06

Label all possible matches ○ Label matches used for scoring ◉

Monoisotopic mass of neutral peptide Mr(calc): 1847.0604
Variable modifications:
N-term : Dimethyl:2H(4) (N-term)
K14 : Dimethyl:2H(4) (K)
Ions Score: 103 **Expect:** 1.3e-008
Matches : 26/170 fragment ions using 26 most intense peaks (help)

#	b	b++	b*	b*++	b0	b0++	Seq.	y	y++	y*	y*++	y0	y0++	#
1	146.1477	73.5775					L							17
2	233.1798	117.0935			215.1692	108.0882	S	***1702.9272***	851.9672	1685.9007	843.4540	1684.9166	842.9620	16
3	362.2224	181.6148			344.2118	172.6095	E	1615.8952	808.4512	1598.8686	799.9379	1597.8846	799.4459	15
4	***475.3064***	238.1569			457.2959	229.1516	L	***1486.8526***	743.9299	1469.8260	735.4167	1468.8420	734.9246	14
5	576.3541	288.6807			558.3435	279.6754	T	***1373.7685***	687.3879	1356.7420	678.8746	1355.7579	678.3826	13
6	***704.4127***	352.7100	687.3861	344.1967	686.4021	343.7047	Q	***1272.7208***	636.8641	1255.6943	628.3508	1254.7103	627.8588	12
7	832.4713	416.7393	815.4447	408.2260	814.4607	407.7340	Q	***1144.6623***	572.8348	1127.6357	564.3215	1126.6517	563.8295	11
8	***945.5553***	473.2813	928.5288	464.7680	927.5448	464.2760	L	***1016.6037***	508.8055	999.5771	500.2922	998.5931	499.8002	10
9	***1016.5924***	508.7999	999.5659	500.2866	998.5819	499.7946	A	903.5196	452.2634	886.4931	443.7502	885.5091	443.2582	9
10	***1144.6510***	572.8291	1127.6245	564.3159	1126.6405	563.8239	Q	832.4825	416.7449	815.4560	408.2316	814.4719	407.7396	8
11	***1215.6881***	608.3477	1198.6616	599.8344	1197.6776	599.3424	A	***704.4239***	352.7156	687.3974	344.2023	686.4134	343.7103	7
12	***1316.7358***	658.8715	1299.7093	650.3583	1298.7253	649.8663	T	***633.3868***	317.1970	616.3603	308.6838	615.3762	308.1918	6
13	***1373.7573***	687.3823	1356.7307	678.8690	1355.7467	678.3770	G	***532.3391***	266.6732	515.3126	258.1599			5

**14**	***1533.9087***	767.4580	1516.8821	758.9447	1515.8981	758.4527	**K**	***475.3177***	238.1625	458.2911	229.6492			**4**
**15**	1630.9614	815.9843	1613.9349	807.4711	1612.9508	806.9791	**P**	***315.1663***	158.0868	298.1397	149.5735			**3**
**16**	1701.9985	851.5029	1684.9720	842.9896	1683.9880	842.4976	**A**	218.1135	109.5604	201.0870	101.0471			**2**
**17**							**Q**	147.0764	74.0418	130.0499	65.5286			**1**

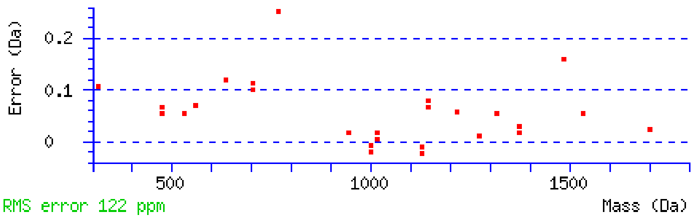

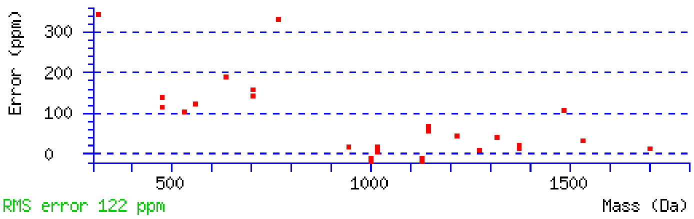

NCBI **BLAST** search of LSELTQQLAQATGKPAQ
(Parameters: blastp, nr protein database, expect=20000, no filter, PAM30)
Other BLAST web gateways

All matches to this query

Score	Mr(calc)	Delta	Sequence
103.3	1847.0604	0.0022	LSELTQQLAQATGKPAQ
18.4	1846.9858	0.0768	SSSFSIEEIQAVVPYR
15.2	1846.9497	0.1128	DSAVSVPTTQANATRRGS
14.9	1846.9930	0.0696	GVGAVPAAAAGSAGAAASLGNAT
14.0	1846.9975	0.0650	VFSLTQQPVRMEAAKA
13.8	1846.8692	0.1933	EDLEMRPGDMITLLE
13.3	1846.9887	0.0738	DSSITGELEKQLLQAN
13.1	1847.0780	-0.0154	ALCLQTQLLPYLALLT
12.6	1846.9207	0.1419	ELLRMGEVQTAQDNR
12.3	1847.0040	0.0585	LEWAASSVEVTAVAAKN

Mascot: http://www.matrixscience.com/

LSELTQQLAQATGKPAQ

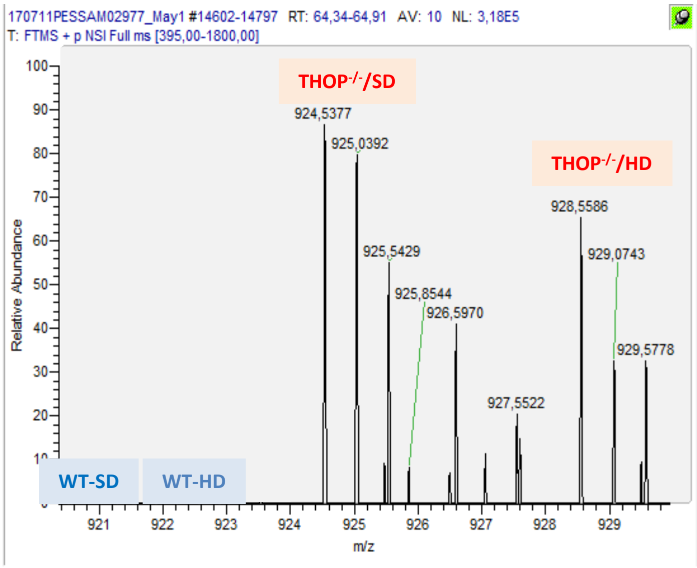

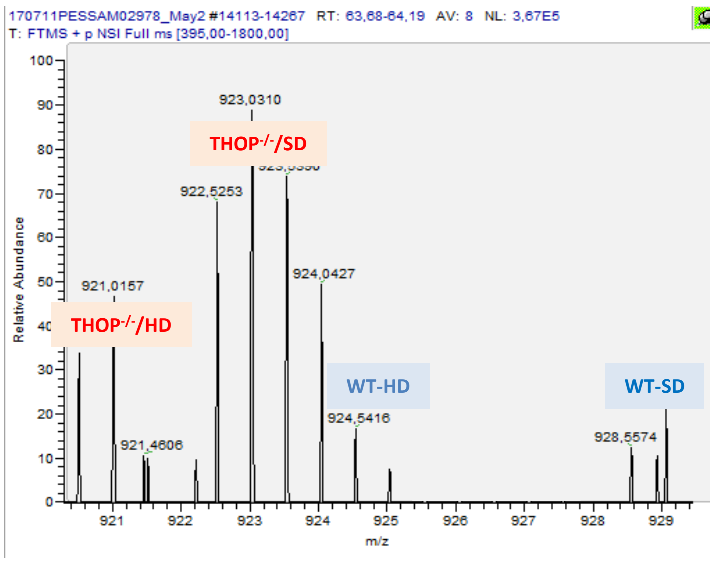

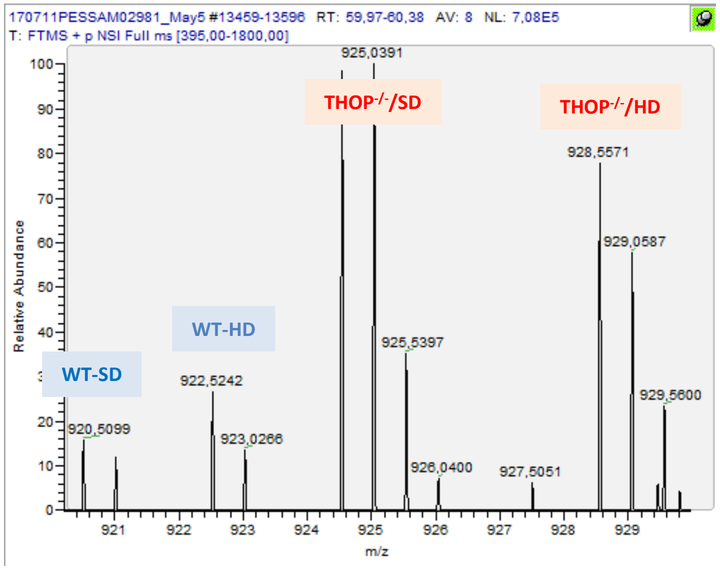

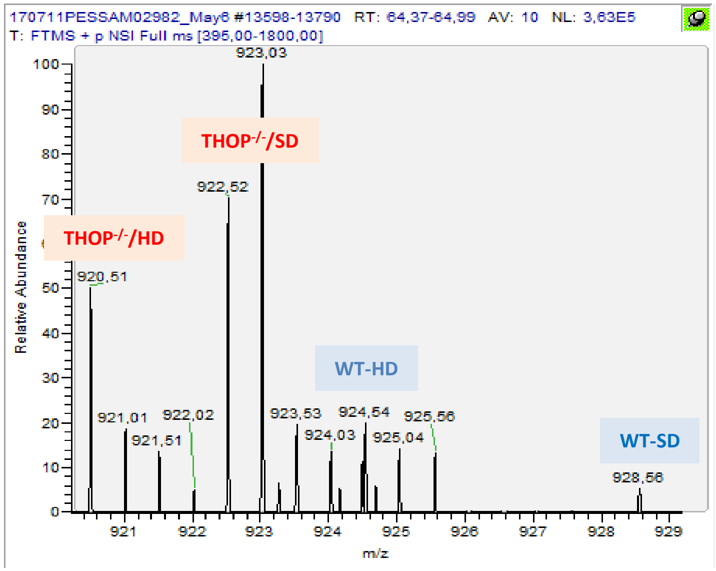

LSELTQQLAQATGKPAQ

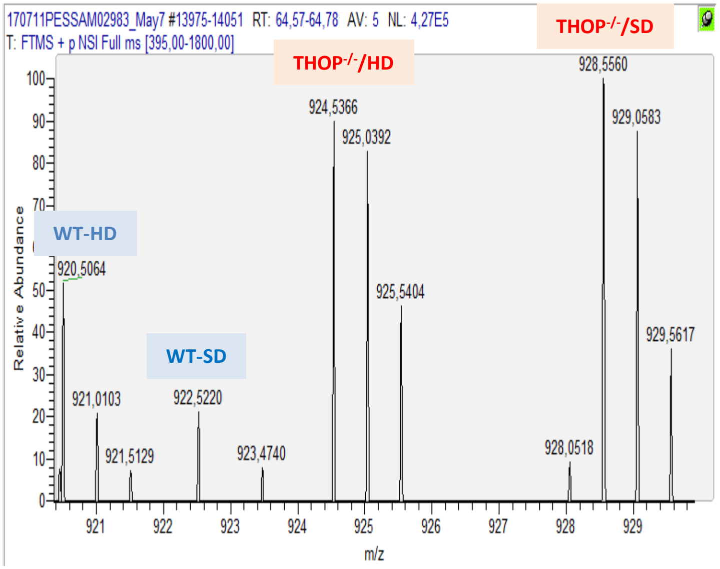

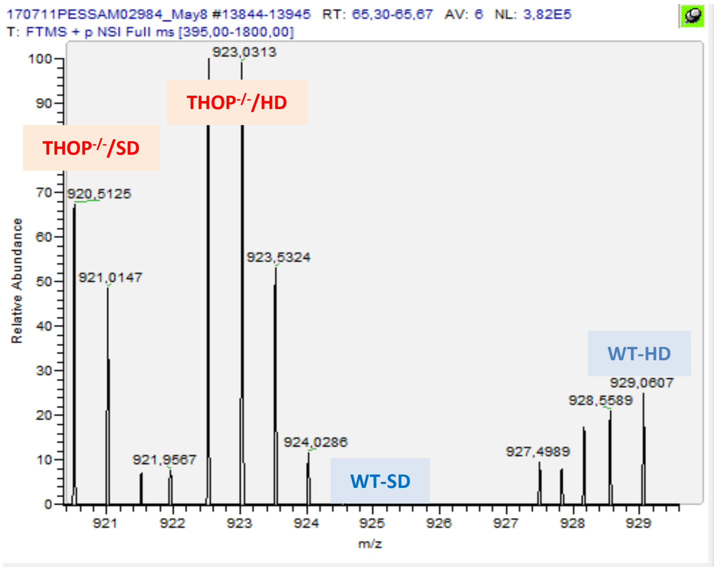

Mascot Search Results

Peptide View

MS/MS Fragmentation of **AQGGVLPNIQAVLLPK**
Found in **H2A2B_MOUSE** in **SwissProt**, Histone H2A type 2-B OS=Mus musculus GN=Hist2h2ab PE=1 SV=3

Match to Query 12540: 1681.073268 from(841.543910,2+) intensity(2031343.0000) scans(22590-22787) rawscans(sn22590:sn22787) rtinseconds(5814.52-5872.8208) index(14871)
Title: 14872: Sum of 2 scans in range 22590 (rt=5814.52) to 22787 (rt=5872.82) [D:\Users\Mayara - HMS Fusion data\170711PESSAM02977_May1.raw]
Data file 170711PESSAM02977_May1.temp.mgf

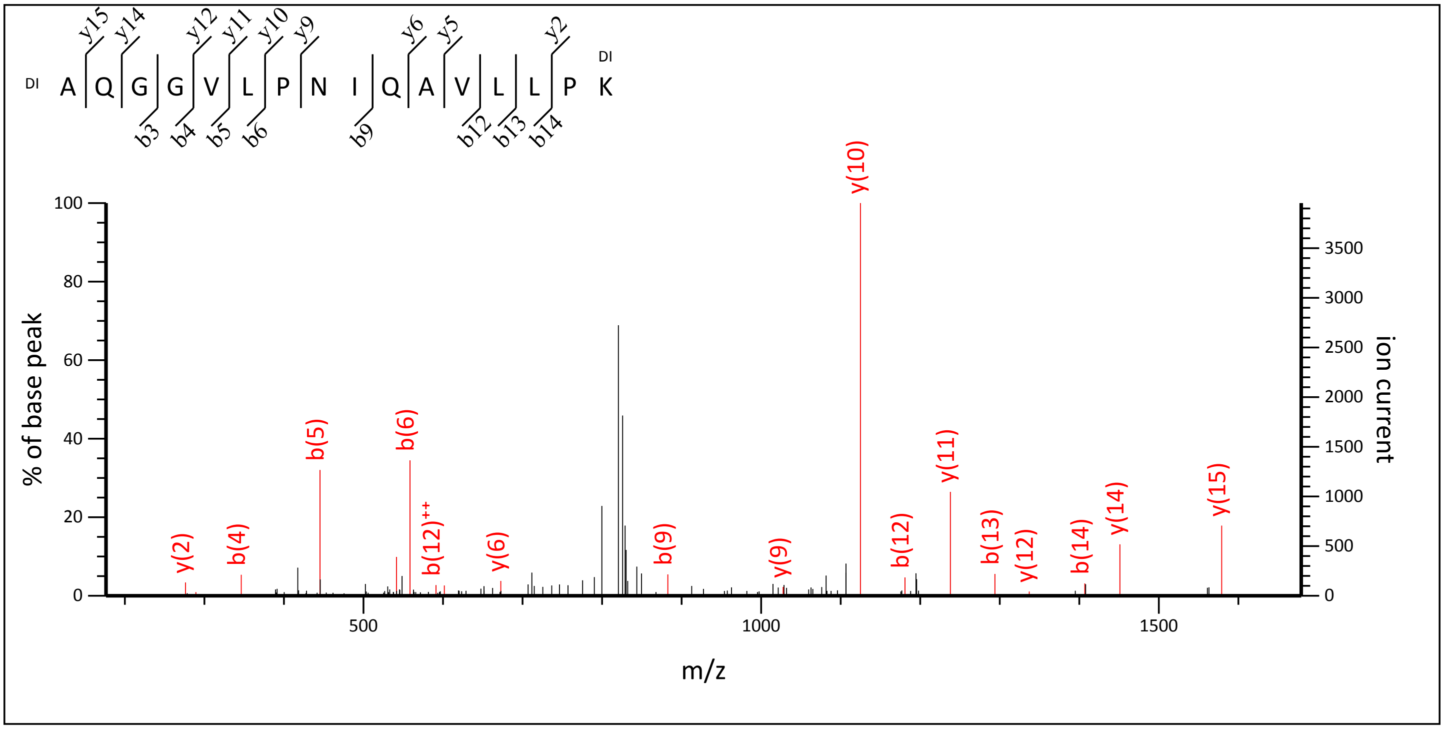

176.3 to 1679.05

Label all possible matches ○ Label matches used for scoring ◉

Monoisotopic mass of neutral peptide Mr(calc): 1681.0742
Variable modifications:
N-term : Dimethyl:2H(4) (N-term)
K16 : Dimethyl:2H(4) (K)
Ions Score: 66 **Expect:** 5.2e-005
Matches : 20/118 fragment ions using 38 most intense peaks (help)

#	b	b^{++}	b*	b^{*++}	Seq.	y	y^{++}	y*	y^{*++}	#
1	104.1008	52.5540			A					16
2	232.1594	116.5833	215.1328	108.0701	Q	***1578.9879***	789.9976	1561.9614	781.4843	15
3	***289.1808***	145.0941	272.1543	136.5808	G	***1450.9294***	725.9683	1433.9028	717.4550	14
4	***346.2023***	173.6048	329.1758	165.0915	G	1393.9079	697.4576	1376.8814	688.9443	13
5	***445.2707***	223.1390	428.2442	214.6257	V	***1336.8864***	668.9469	1319.8599	660.4336	12
6	***558.3548***	279.6810	541.3282	271.1678	L	***1237.8180***	619.4127	1220.7915	610.8994	11
7	655.4075	328.2074	638.3810	319.6941	P	***1124.7340***	562.8706	1107.7074	554.3573	10

8	769.4505	385.2289	752.4239	376.7156	N	*1027.6812*	514.3442	1010.6547	505.8310	9
9	*882.5345*	441.7709	865.5080	433.2576	I	913.6383	457.3228	896.6117	448.8095	8
10	1010.5931	505.8002	993.5666	497.2869	Q	800.5542	400.7807	783.5277	392.2675	7
11	1081.6302	**541.3188**	1064.6037	532.8055	A	*672.4956*	336.7515	655.4691	328.2382	6
12	*1180.6986*	**590.8530**	1163.6721	582.3397	V	*601.4585*	301.2329	584.4320	292.7196	5
13	*1293.7827*	647.3950	1276.7562	638.8817	L	502.3901	251.6987	485.3636	243.1854	4
14	*1406.8668*	703.9370	1389.8402	695.4237	L	389.3060	195.1567	372.2795	186.6434	3
15	1503.9195	752.4634	1486.8930	743.9501	P	*276.2220*	138.6146	259.1954	130.1014	2
16					K	179.1692	90.0882	162.1427	81.5750	1

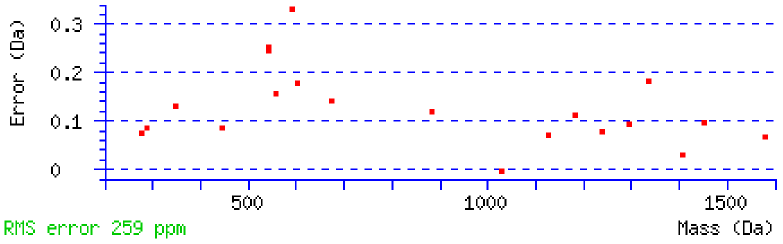

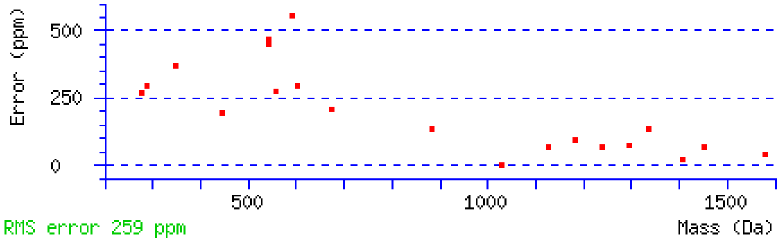

NCBI **BLAST** search of AQGGVLPNIQAVLLPK
(Parameters: blastp, nr protein database, expect=20000, no filter, PAM30)
Other BLAST web gateways

All matches to this query

Score	Mr(calc)	Delta	Sequence
65.7	1681.0742	-0.0009	AQGGVLPNIQAVLLPK
19.6	1680.8467	0.2266	MDAVLLVIAQTFMDA
19.4	1680.9826	0.0906	AWAAMALTFLLVLLT
19.4	1680.8215	0.2518	MVMPNLYGNIVNNV
18.5	1680.8458	0.2275	TDTGSAEQFDLVILT
18.1	1680.8252	0.2481	AGLEIMEQMALLQE
18.0	1680.7885	0.2848	DSAVPCAMMLELARA
17.8	1680.9484	0.1248	GDDSLKMHVVKILT
17.5	1681.0906	-0.0173	IFKLELPLKQKGVG
17.4	1681.0207	0.0526	LRPLELPVTPVIEQ

Mascot: http://www.matrixscience.com/

AQGGVLPNIQAVLLPK

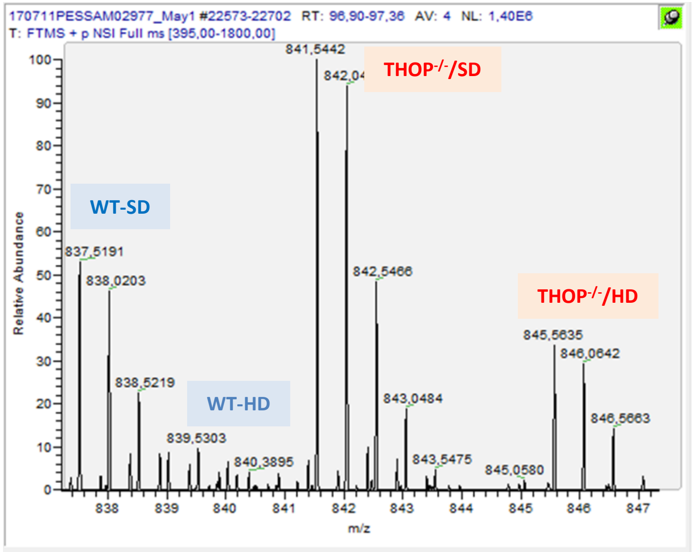

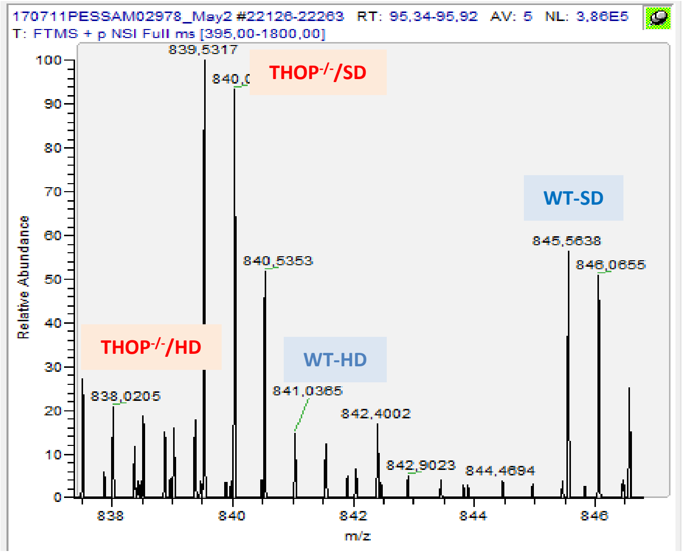

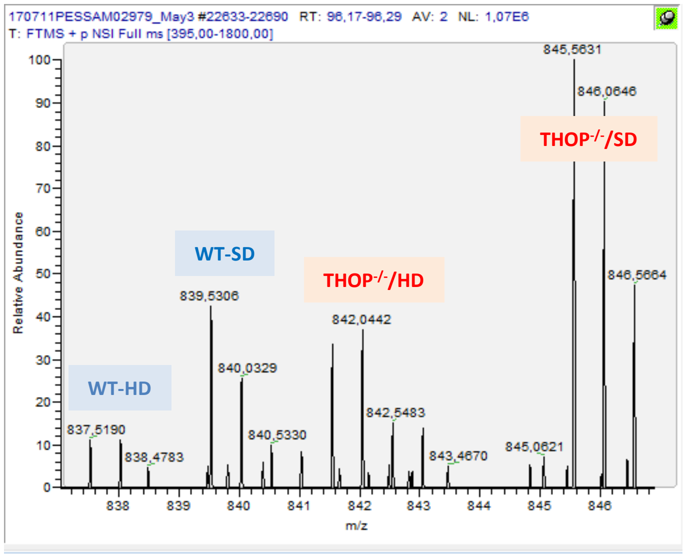

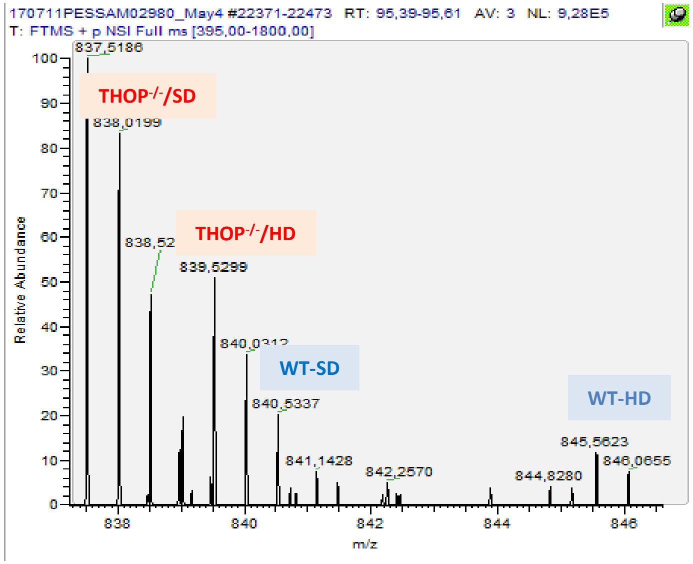

AQGGVLPNIQAVLLPK

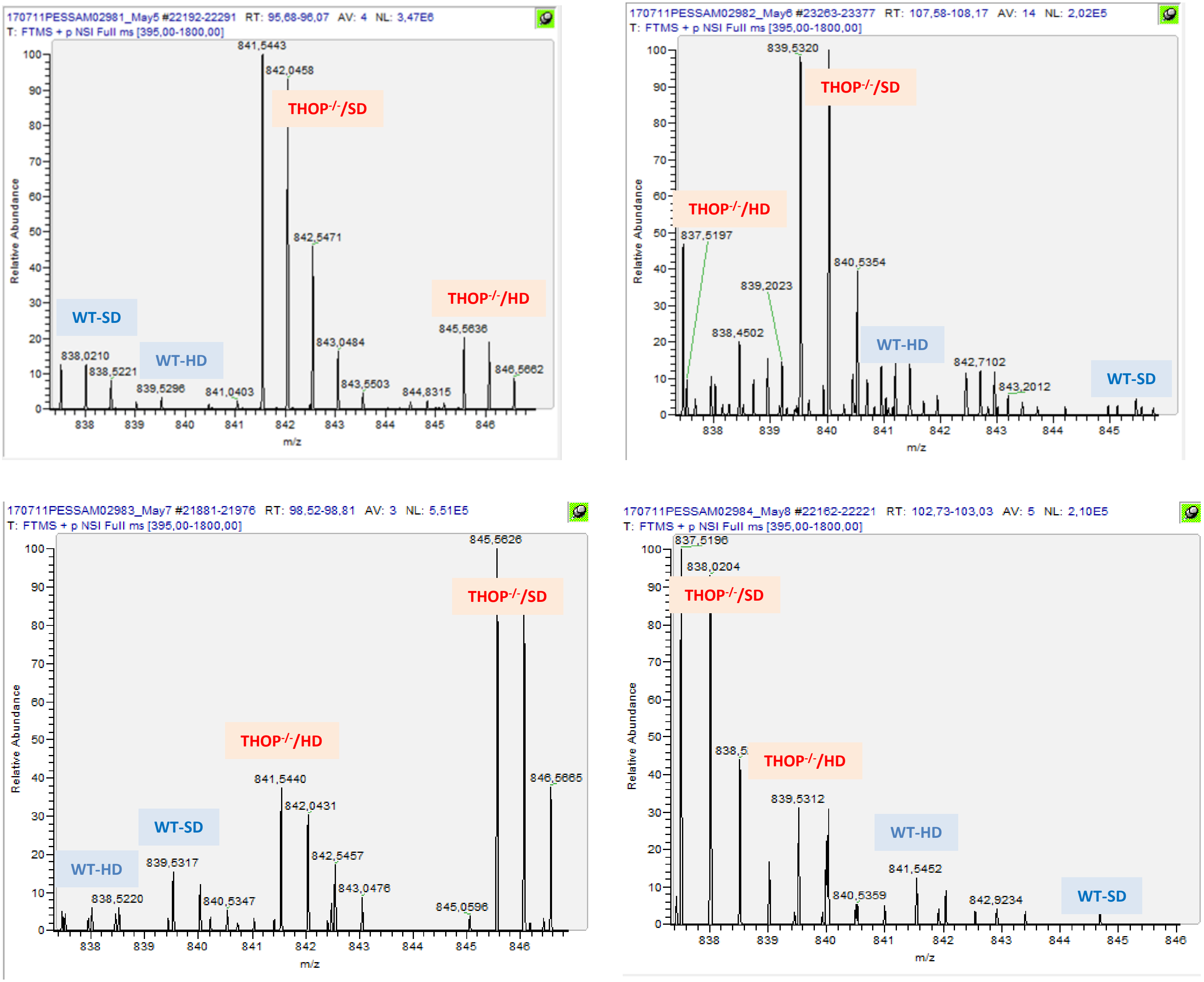

AQGGVLPNIQAVLLPK

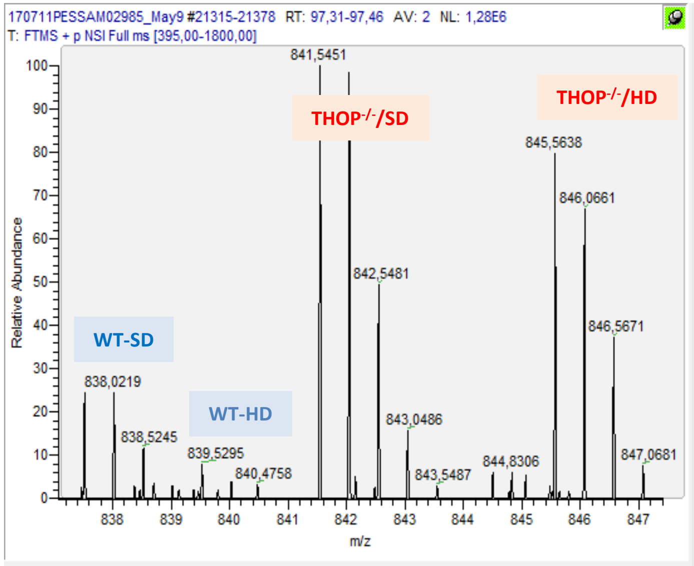

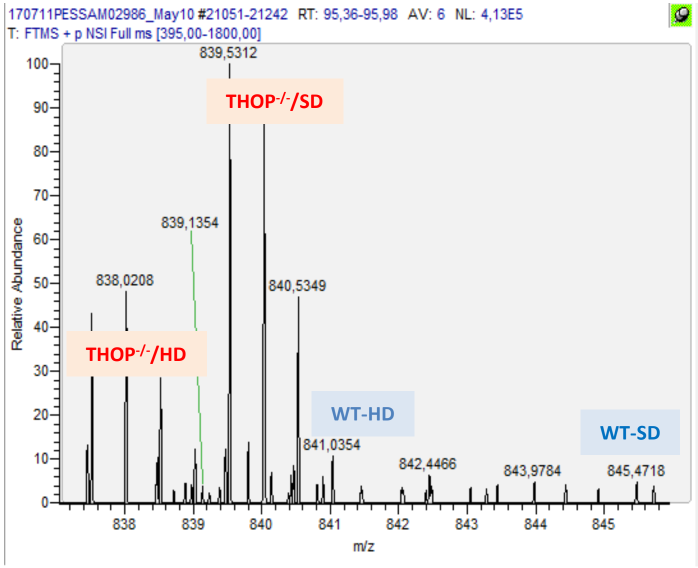

Mascot Search Results

Peptide View

MS/MS Fragmentation of **KQVHPDTGISSKAMGIMNS**
Found in **H2B1M_MOUSE** in **SwissProt**, Histone H2B type 1-M OS=Mus musculus GN=Hist1h2bm PE=1 SV=2

Match to Query 16712: 2124.205152 from(709.075660,3+) intensity(7826568.5000) scans(6099) rawscans(sn6099) rtinseconds(2250.2798) index(3917)
Title: 3918: Scan 6099 (rt=2250.28) [D:\Users\Mayara - HMS Fusion data\170711PESSAM02985_May9.raw]
Data file 170711PESSAM02985_May9.temp.mgf

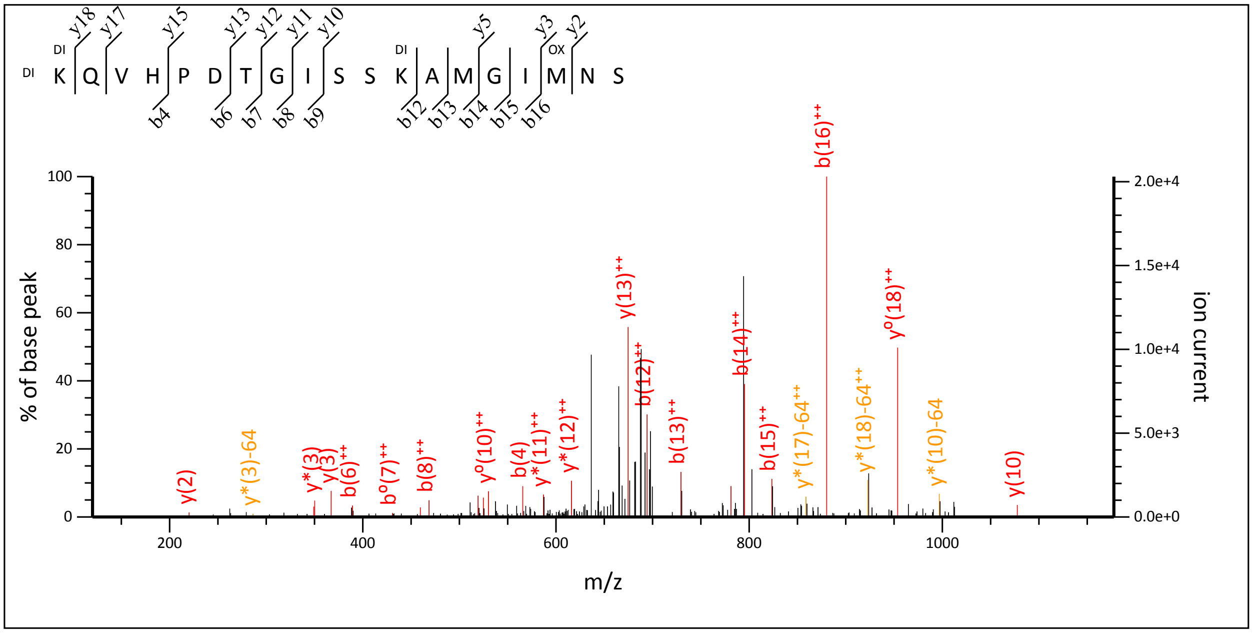

120.18 to 1177.58

Label all possible matches ○ Label matches used for scoring ◉

Monoisotopic mass of neutral peptide Mr(calc): 2124.2039
Variable modifications:
N-term : Dimethyl:2H(6)13C(2) (N-term)
K1 : Dimethyl:2H(6)13C(2) (K)
K12 : Dimethyl:2H(6)13C(2) (K)
M17 : Oxidation (M), with neutral losses 0.0000(shown in table), 63.9983
Ions Score: 33 **Expect:** 0.12
Matches : 31/312 fragment ions using 49 most intense peaks (help)

#	b	b^{++}	b*	b^{*++}	b^0	b^{0++}	Seq.	y	y^{++}	y*	y^{*++}	y^0	y^{0++}	#
1	201.2536	101.1304	184.2270	92.6172			K							19
2	329.3122	165.1597	312.2856	156.6464			Q	1924.9648	962.9861	1907.9383	954.4728	1906.9543	953.9808	18
3	428.3806	214.6939	411.3540	206.1806			V	1796.9063	898.9568	1779.8797	890.4435	1778.8957	889.9515	17
4	565.4395	283.2234	548.4129	274.7101			H	1697.8378	849.4226	1680.8113	840.9093	1679.8273	840.4173	16
5	662.4922	331.7498	645.4657	323.2365			P	1560.7789	780.8931	1543.7524	772.3798	1542.7684	771.8878	15
6	777.5192	***389.2632***	760.4926	380.7500	759.5086	380.2580	D	1463.7262	732.3667	1446.6996	723.8534	1445.7156	723.3614	14
7	878.5669	439.7871	861.5403	431.2738	860.5563	430.7818	T	1348.6992	674.8532	1331.6727	666.3400	1330.6887	665.8480	13
8	935.5883	***468.2978***	918.5618	459.7845	917.5778	459.2925	G	1247.6515	624.3294	1230.6250	615.8161	1229.6410	615.3241	12
9	1048.6724	***524.8398***	1031.6458	516.3266	1030.6618	515.8346	I	1190.6301	595.8187	1173.6035	587.3054	1172.6195	586.8134	11
10	1135.7044	568.3559	1118.6779	559.8426	1117.6939	559.3506	S	**1077.5460**	539.2766	1060.5195	530.7634	1059.5354	530.2714	10
11	1222.7365	611.8719	1205.7099	603.3586	1204.7259	602.8666	S	990.5140	495.7606	973.4874	487.2474	972.5034	486.7553	9
12	1386.9071	***693.9572***	1369.8805	685.4439	1368.8965	684.9519	K	903.4820	452.2446	886.4554	443.7313	885.4714	443.2393	8
13	1457.9442	***729.4757***	1440.9177	720.9625	1439.9336	720.4705	A	739.3113	370.1593	722.2848	361.6460	721.3008	361.1540	7
14	1588.9847	***794.9960***	1571.9581	786.4827	1570.9741	785.9907	M	668.2742	334.6407	651.2477	326.1275	650.2636	325.6355	6

**15**	1646.0061	***823.5067***	1628.9796	814.9934	1627.9956	814.5014	**G**	537.2337	269.1205	520.2072	260.6072	519.2232	260.1152	**5**
**16**	1759.0902	***880.0487***	1742.0637	871.5355	1741.0796	871.0435	**I**	480.2123	240.6098	463.1857	232.0965	462.2017	231.6045	**4**
**17**	1906.1256	***953.5664***	1889.0991	945.0532	1888.1150	944.5612	**M**	**367.1282**	184.0677	350.1016	175.5545	349.1176	175.0625	**3**
**18**	2020.1685	1010.5879	2003.1420	1002.0746	2002.1580	1001.5826	**N**	**220.0928**	110.5500	203.0662	102.0368	202.0822	101.5448	**2**
**19**							**S**	106.0499	53.5286			88.0393	44.5233	**1**

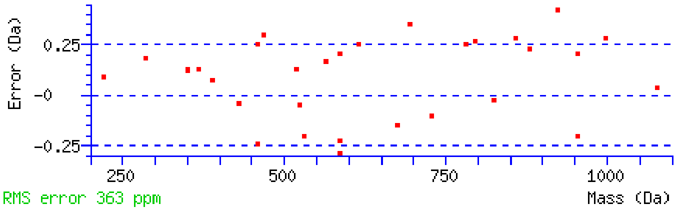

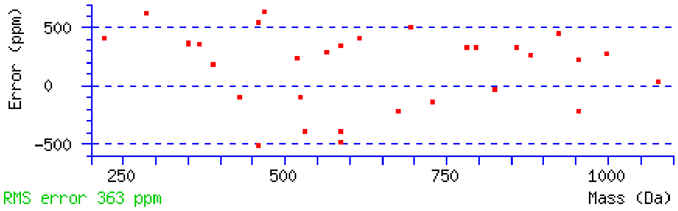

NCBI **BLAST** search of KQVHPDTGISSKAMGIMNS
(Parameters: blastp, nr protein database, expect=20000, no filter, PAM30)
Other BLAST web gateways

All matches to this query

Score	Mr(calc)	Delta	Sequence
33.4	2124.2039	0.0013	KQVHPDTGISSKAMGIMNS
23.1	2123.9537	0.2514	TGLETIGRDTCPGEMGAEMI
22.1	2124.0193	0.1858	TLTDEDFSPFGSRGGLFSN
21.4	2123.9948	0.2103	FMLGTIPFPEGMGGSVYFS
21.0	2124.1352	0.0700	APVVNESGAILGMVTLGNMLS
20.8	2124.0752	0.1299	YFHRDRLNTGSKDLFAE
20.7	2124.0751	0.1300	YNIVLFLTTFQESLGMSN
20.6	2124.1430	0.0621	LSSLGQIDGWSSMQLRAVV
19.8	2124.0918	0.1133	RERMDDSSYTSKLLSCK
19.4	2123.9892	0.2159	SVSLLSALATSDPGQMDSSQS

Mascot: http://www.matrixscience.com/

KQVHPDTGISSKAMGImNS

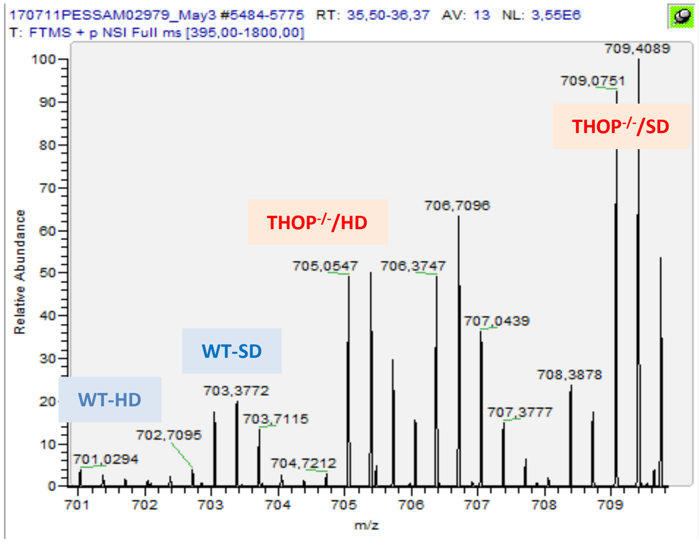

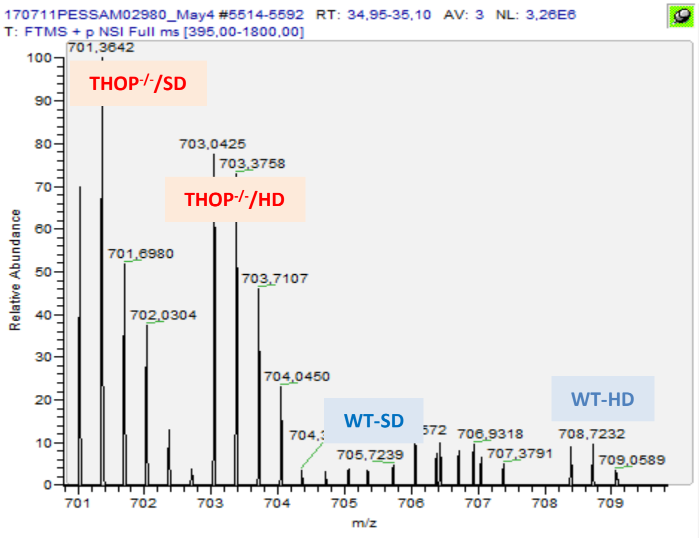

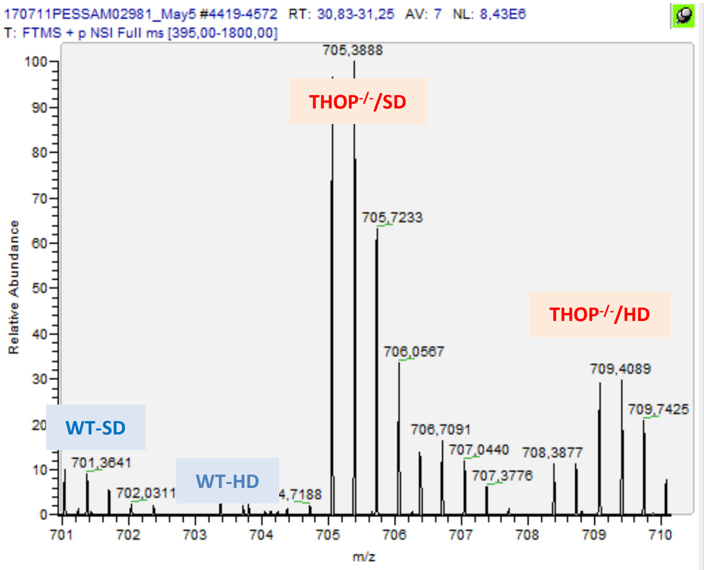

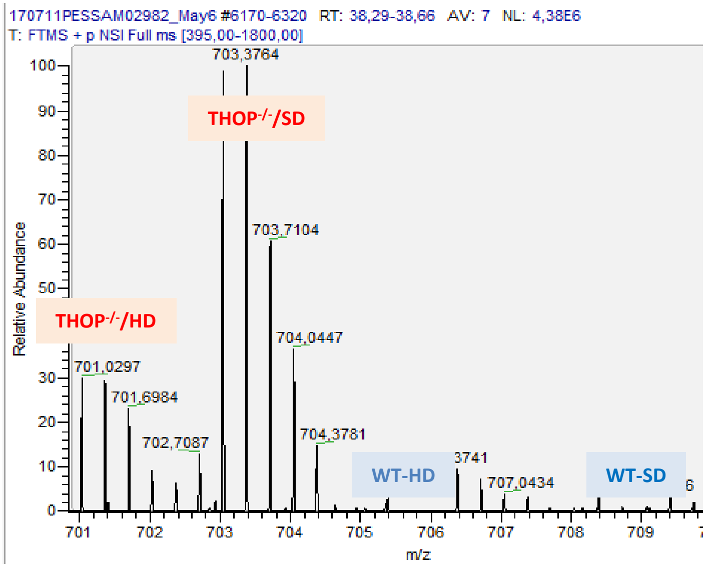

KQVHPDTGISSKAMGImNS

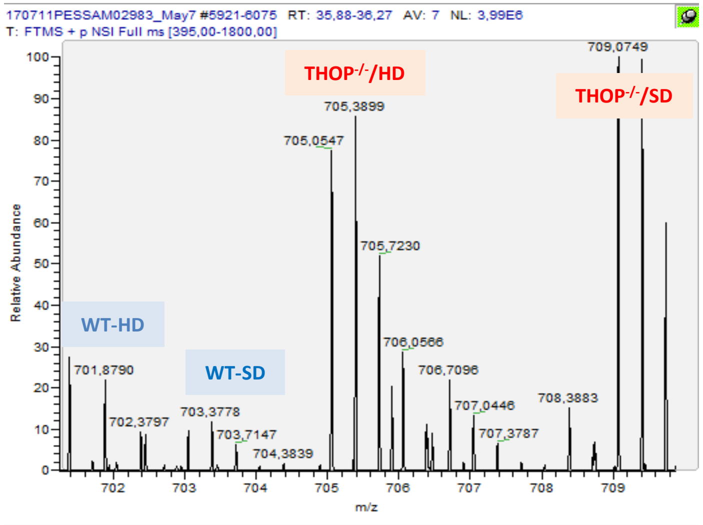

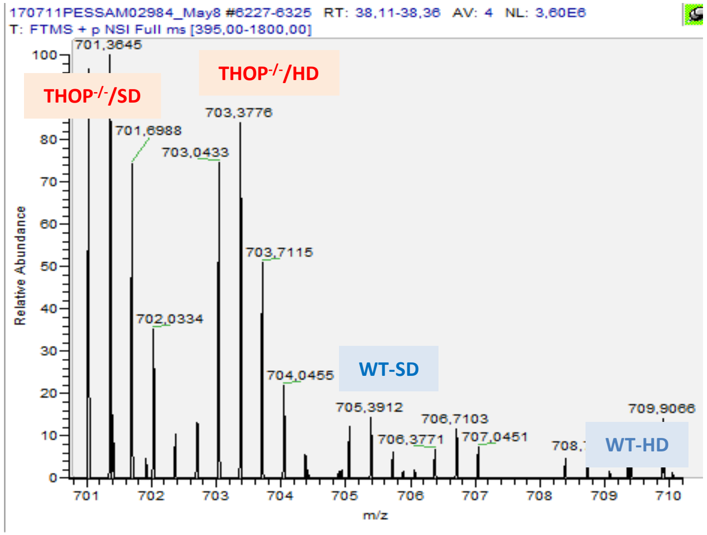

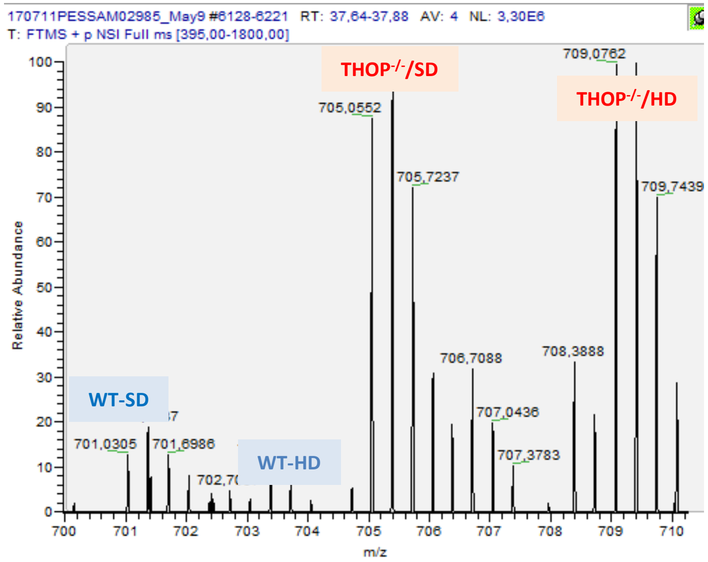

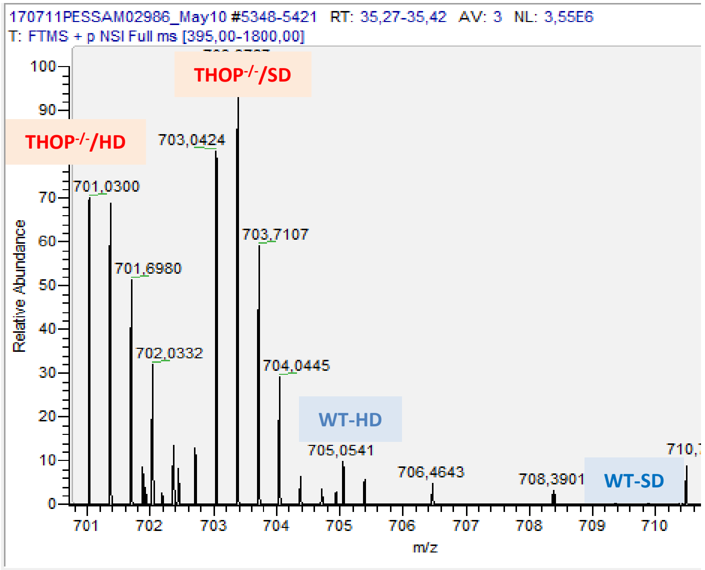

Mascot Search Results

Peptide View

MS/MS Fragmentation of **ASVLTAQPRLMEPI**
Found in **EF2_MOUSE** in **SwissProt**, Elongation factor 2 OS=Mus musculus GN=Eef2 PE=1 SV=2

Match to Query 10834: 1560.910508 from(781.462530,2+) intensity(2986679.8000) scans(18798) rawscans(sn18798) rtinseconds(4807.0934) index(12263)
Title: 12264: Scan 18798 (rt=4807.09) [D:\Users\Mayara - HMS Fusion data\170711PESSAM02977_May1.raw]
Data file 170711PESSAM02977_May1.temp.mgf

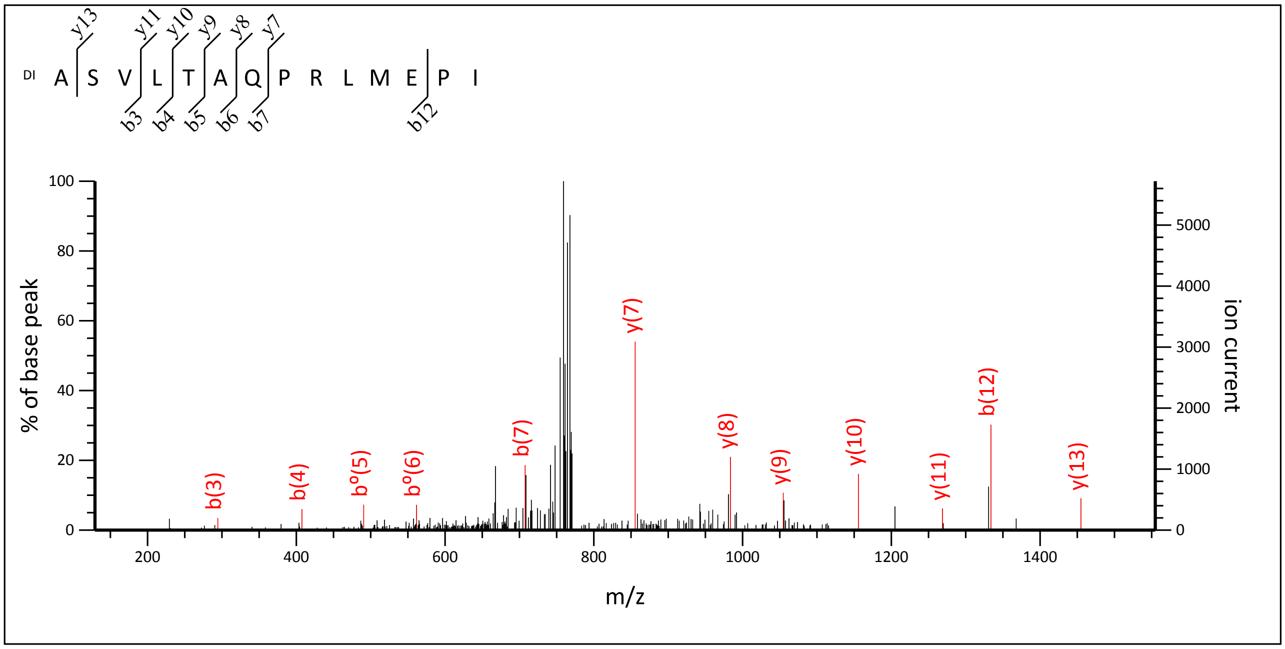

129.28 to 1554.87

Label all possible matches ○ Label matches used for scoring ◉

Monoisotopic mass of neutral peptide Mr(calc): 1560.9090
Variable modifications:
N-term : Dimethyl:2H(6)13C(2) (N-term)
Ions Score: 48 **Expect:** 0.022
Matches : 13/128 fragment ions using 13 most intense peaks (help)

#	b	b^{++}	b*	b^{*++}	b^{0}	b^{0++}	Seq.	y	y^{++}	y*	y^{*++}	y^{0}	y^{0++}	#
1	108.1201	54.5637					A							14
2	195.1521	98.0797			177.1415	89.0744	S	***1454.8036***	727.9054	1437.7770	719.3921	1436.7930	718.9001	13
3	***294.2205***	147.6139			276.2099	138.6086	V	1367.7715	684.3894	1350.7450	675.8761	1349.7610	675.3841	12
4	***407.3046***	204.1559			389.2940	195.1506	L	***1268.7031***	634.8552	1251.6766	626.3419	1250.6926	625.8499	11
5	508.3522	254.6798			490.3417	245.6745	T	***1155.6191***	578.3132	1138.5925	569.7999	1137.6085	569.3079	10
6	579.3894	290.1983			561.3788	281.1930	A	***1054.5714***	527.7893	1037.5448	519.2761	1036.5608	518.7840	9
7	***707.4479***	354.2276	690.4214	345.7143	689.4374	345.2223	Q	***983.5343***	492.2708	966.5077	483.7575	965.5237	483.2655	8
8	804.5007	402.7540	787.4742	394.2407	786.4901	393.7487	P	***855.4757***	428.2415	838.4491	419.7282	837.4651	419.2362	7
9	960.6018	480.8045	943.5753	472.2913	942.5912	471.7993	R	758.4229	379.7151	741.3964	371.2018	740.4124	370.7098	6
10	1073.6859	537.3466	1056.6593	528.8333	1055.6753	528.3413	L	602.3218	301.6645			584.3112	292.6593	5
11	1204.7264	602.8668	1187.6998	594.3535	1186.7158	593.8615	M	489.2377	245.1225			471.2272	236.1172	4
12	***1333.7690***	667.3881	1316.7424	658.8748	1315.7584	658.3828	E	358.1973	179.6023			340.1867	170.5970	3
13	1430.8217	715.9145	1413.7952	707.4012	1412.8112	706.9092	P	229.1547	115.0810					2
14							I	132.1019	66.5546					1

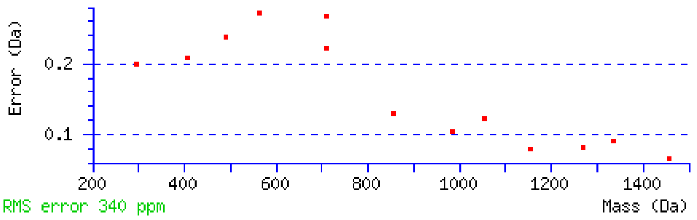

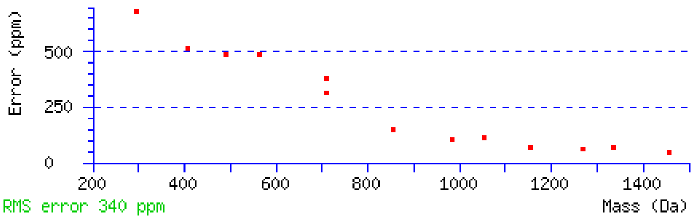

NCBI **BLAST** search of ASVLTAQPRLMEPI
(Parameters: blastp, nr protein database, expect=20000, no filter, PAM30)
Other BLAST web gateways

All matches to this query

Score	Mr(calc)	Delta	Sequence
48.5	1560.9090	0.0015	ASVLTAQPRLMEPI
29.5	1560.8850	0.0255	FLRFCKQATLQF
28.0	1560.9007	0.0098	AEGLTCLIKVGAEAD
26.9	1560.7915	0.1190	GEALTAEKEHMEE
22.4	1560.9279	-0.0174	KLPIYALAQTLGAF
20.6	1560.8868	0.0237	QELTSKQGGRCSPV
19.3	1560.8624	0.0482	RLRLNEEATLQF
18.9	1560.7781	0.1324	QELTAMRMSRDTA
18.9	1560.7781	0.1324	QELTAMRMSRDTA
17.6	1560.8483	0.0622	DRDALVGLAVLLGCD

Mascot: http://www.matrixscience.com/

ASVLTAQPRLMEPI

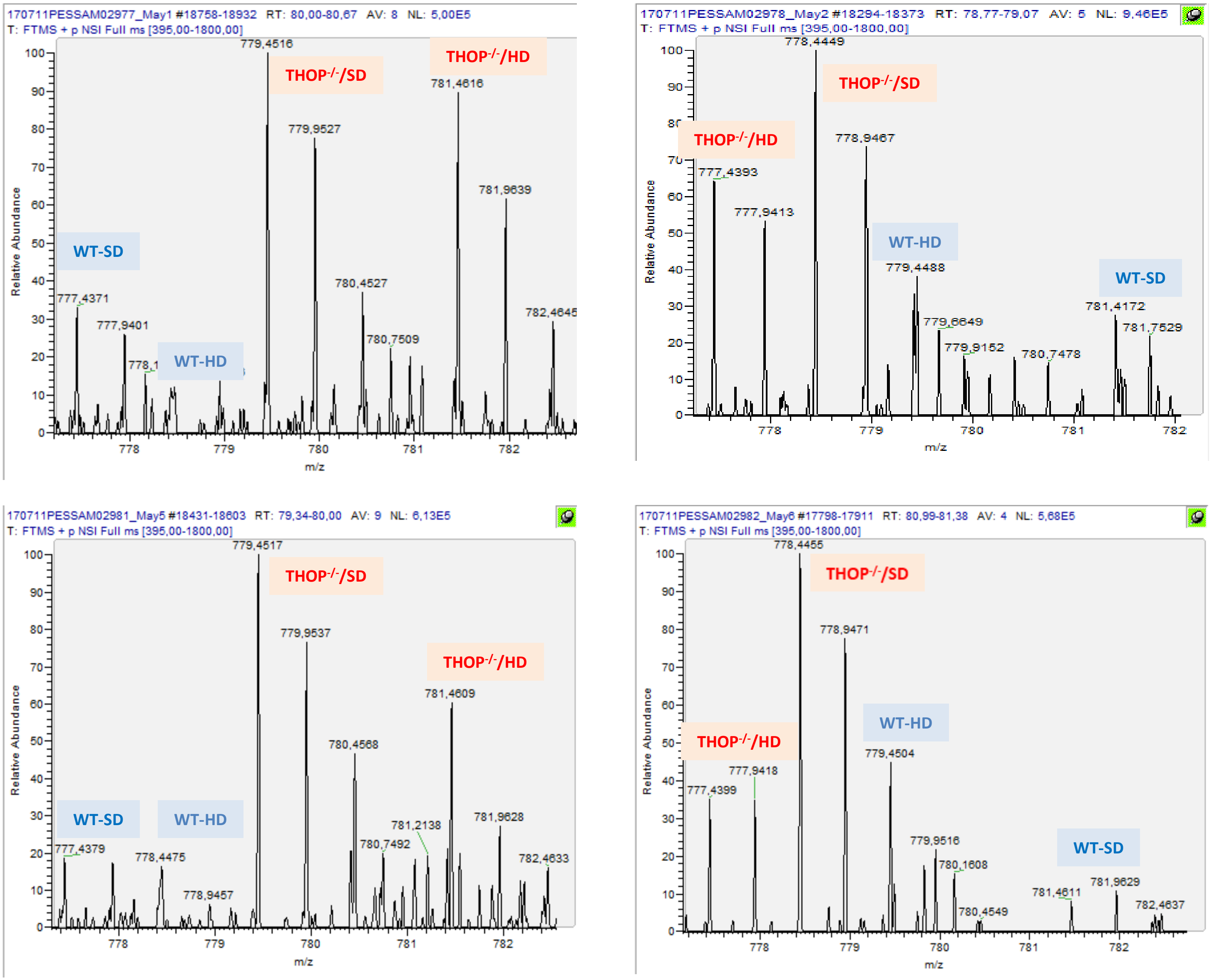

ASVLTAQPRLMEPI

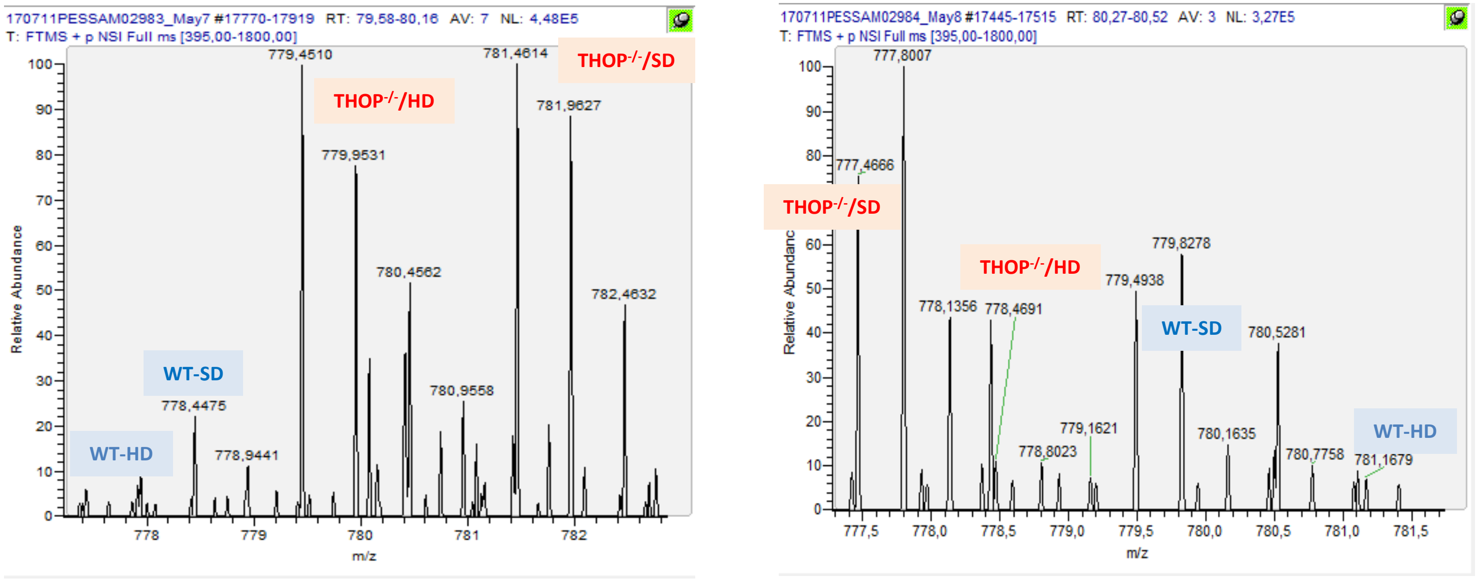

Mascot Search Results

Peptide View

MS/MS Fragmentation of **DISNADRLGSSEVEQ**
Found in **KCRM_MOUSE** in **SwissProt**, Creatine kinase M-type OS=Mus musculus GN=Ckm PE=1 SV=1

Match to Query 12401: 1650.795028 from(826.404790,2+) intensity(1203988.6000) scans(9733) rawscans(sn9733) rtinseconds(2944.0576) index(6186)
Title: 6187: Scan 9733 (rt=2944.06) [D:\Users\Mayara - HMS Fusion data\170711PESSAM02979_May3.raw]
Data file 170711PESSAM02979_May3.temp.mgf

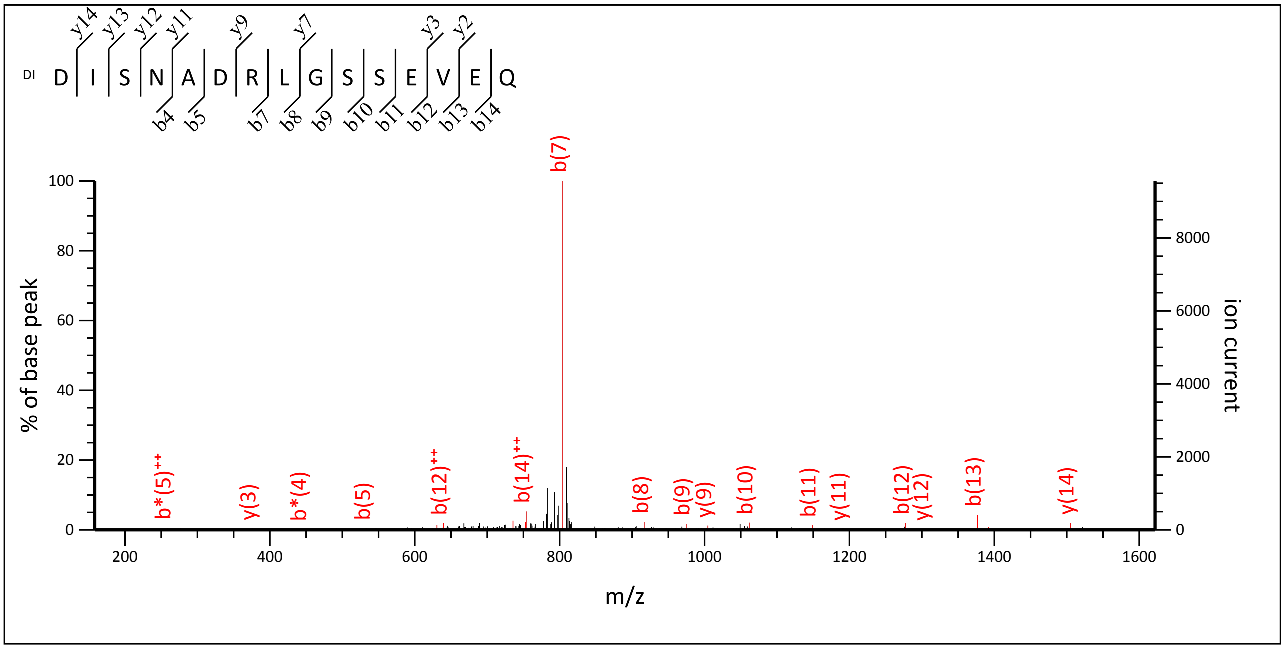

158.26 to 1621.86

Label all possible matches ○ Label matches used for scoring ◉

```
Monoisotopic mass of neutral peptide Mr(calc): 1650.7999
Variable modifications:
N-term : Dimethyl:2H(4) (N-term)
Ions Score: 75  Expect: 8.1e-005
Matches : 28/160 fragment ions using 37 most intense peaks   (help)
```

#	b	b^{++}	b*	b^{*++}	b^0	b^{0++}	Seq.	y	y^{++}	y*	y^{*++}	y^0	y^{0++}	#
1	148.0906	74.5490			130.0801	65.5437	D							15
2	261.1747	131.0910			243.1641	122.0857	I	***1504.7238***	752.8655	1487.6972	744.3523	1486.7132	743.8603	14
3	348.2067	174.6070			330.1962	165.6017	S	***1391.6397***	696.3235	1374.6132	687.8102	1373.6292	687.3182	13
4	462.2496	231.6285	445.2231	223.1152	444.2391	222.6232	N	***1304.6077***	652.8075	1287.5811	644.2942	1286.5971	643.8022	12
5	***533.2868***	267.1470	516.2602	258.6337	515.2762	258.1417	A	***1190.5648***	595.7860	1173.5382	587.2727	1172.5542	586.7807	11
6	648.3137	324.6605	631.2872	316.1472	630.3031	315.6552	D	1119.5277	560.2675	1102.5011	551.7542	1101.5171	551.2622	10
7	***804.4148***	402.7110	787.3883	394.1978	786.4042	393.7058	R	***1004.5007***	502.7540	987.4742	494.2407	986.4901	493.7487	9
8	***917.4989***	459.2531	900.4723	450.7398	899.4883	450.2478	L	848.3996	424.7034	831.3731	416.1902	830.3890	415.6982	8
9	***974.5203***	487.7638	957.4938	479.2505	956.5098	478.7585	G	***735.3155***	368.1614	718.2890	359.6481	717.3050	359.1561	7
10	***1061.5524***	531.2798	1044.5258	522.7665	1043.5418	522.2745	S	678.2941	339.6507	661.2675	331.1374	660.2835	330.6454	6
11	***1148.5844***	574.7958	1131.5578	566.2826	1130.5738	565.7906	S	591.2620	296.1347	574.2355	287.6214	573.2515	287.1294	5
12	***1277.6270***	**639.3171**	1260.6004	630.8039	1259.6164	630.3119	E	504.2300	252.6186	487.2035	244.1054	486.2195	243.6134	4
13	***1376.6954***	688.8513	1359.6689	680.3381	1358.6848	679.8461	V	***375.1874***	188.0974	358.1609	179.5841	357.1769	179.0921	3
14	1505.7380	**753.3726**	1488.7114	744.8594	1487.7274	744.3674	E	***276.1190***	138.5631	259.0925	130.0499	258.1084	129.5579	2

15							Q	147.0764	74.0418	130.0499	65.5286			1

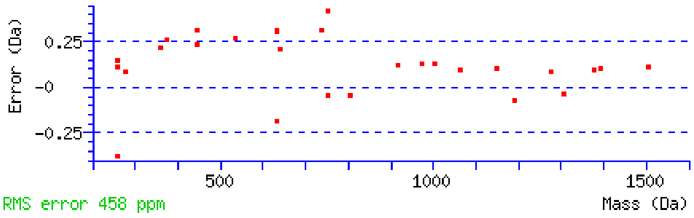

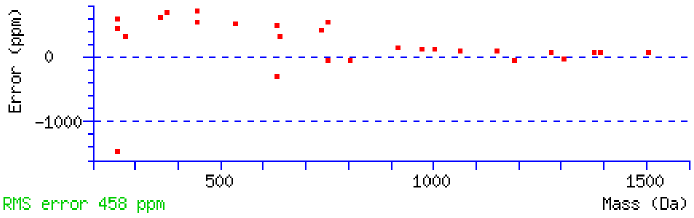

NCBI **BLAST** search of DISNADRLGSSEVEQ
(Parameters: blastp, nr protein database, expect=20000, no filter, PAM30)
Other BLAST web gateways

All matches to this query

Score	Mr(calc)	Delta	Sequence
75.0	1650.7999	-0.0048	DISNADRLGSSEVEQ
30.3	1650.9810	-0.1860	RLSNVSLTGLGTVRT
25.1	1650.7634	0.0316	RSVEEAVNGSEEENA
24.1	1651.0120	-0.2170	VSGTTLRGLIKLNDT
23.7	1650.8875	-0.0925	ARVTWCAVGSEEKR
22.9	1650.7828	0.0123	DQVSAYGFITNNYQ
22.4	1650.7407	0.0544	SEQEVNELCQSVQE
21.9	1650.9506	-0.1556	EVLCVSVLKLWQE
21.8	1650.7948	0.0002	TSSASSVTGVVNGESLE
21.6	1650.8626	-0.0676	DQVSEDTVEMPLPK

Mascot: http://www.matrixscience.com/

DISNADRLGSSEVEQ

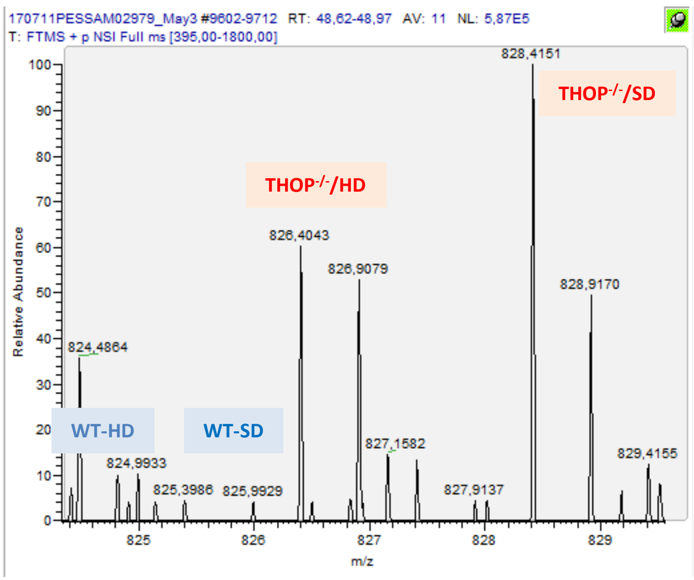

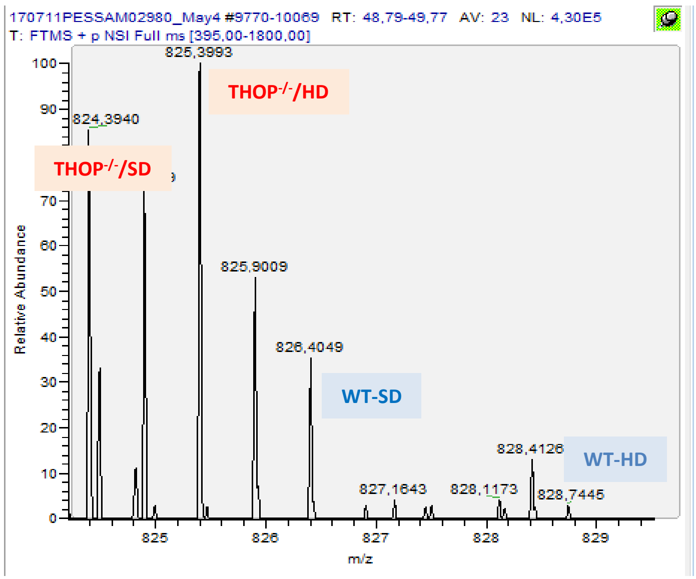

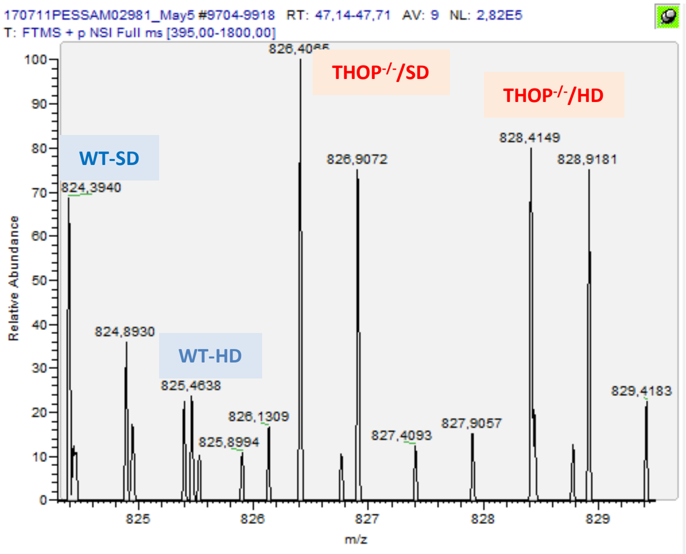

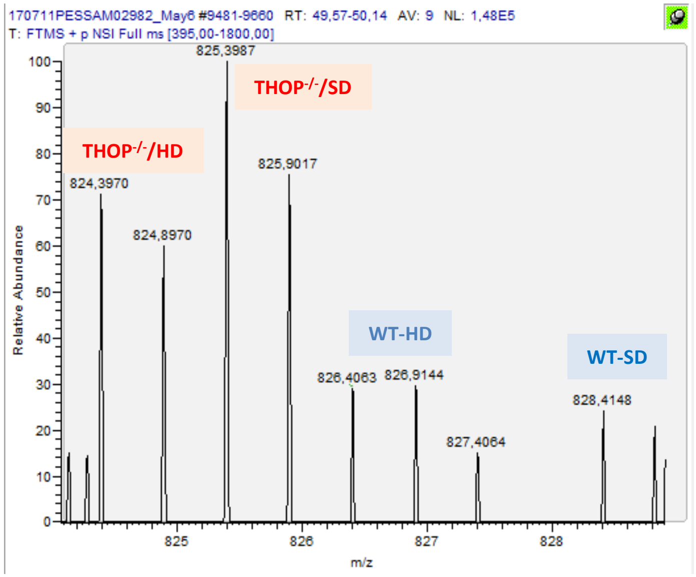

DISNADRLGSSEVEQ

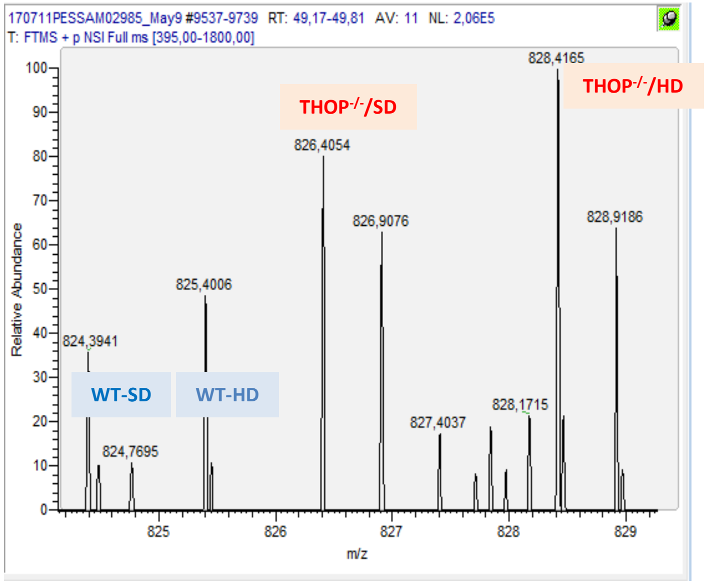

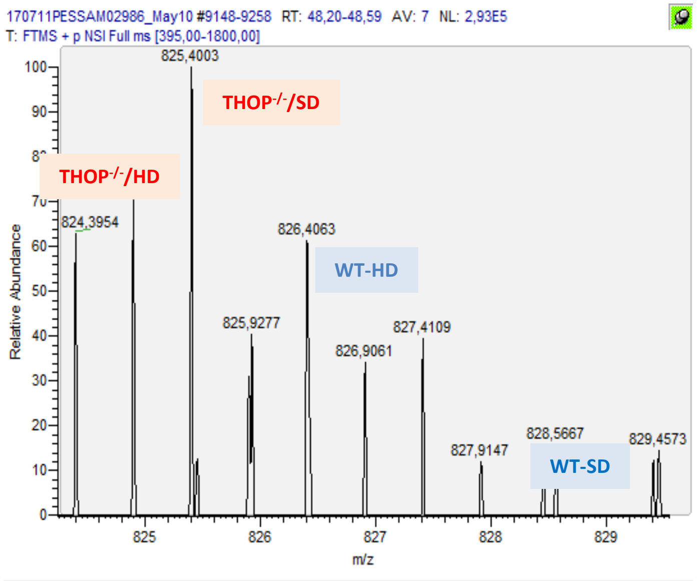

Mascot Search Results

Peptide View

MS/MS Fragmentation of **DISNADRLGSSEVEQV**
Found in **KCRM_MOUSE** in **SwissProt**, Creatine kinase M-type OS=Mus musculus GN=Ckm PE=1 SV=1

Match to Query 13809: 1753.890468 from(877.952510,2+) intensity(1823285.3000) scans(13413) rawscans(sn13413) rtinseconds(3585.2932) index(8910)
Title: 8911: Scan 13413 (rt=3585.29) [D:\Users\Mayara - HMS Fusion data\170711PESSAM02981_May5.raw]
Data file 170711PESSAM02981_May5.temp.mgf

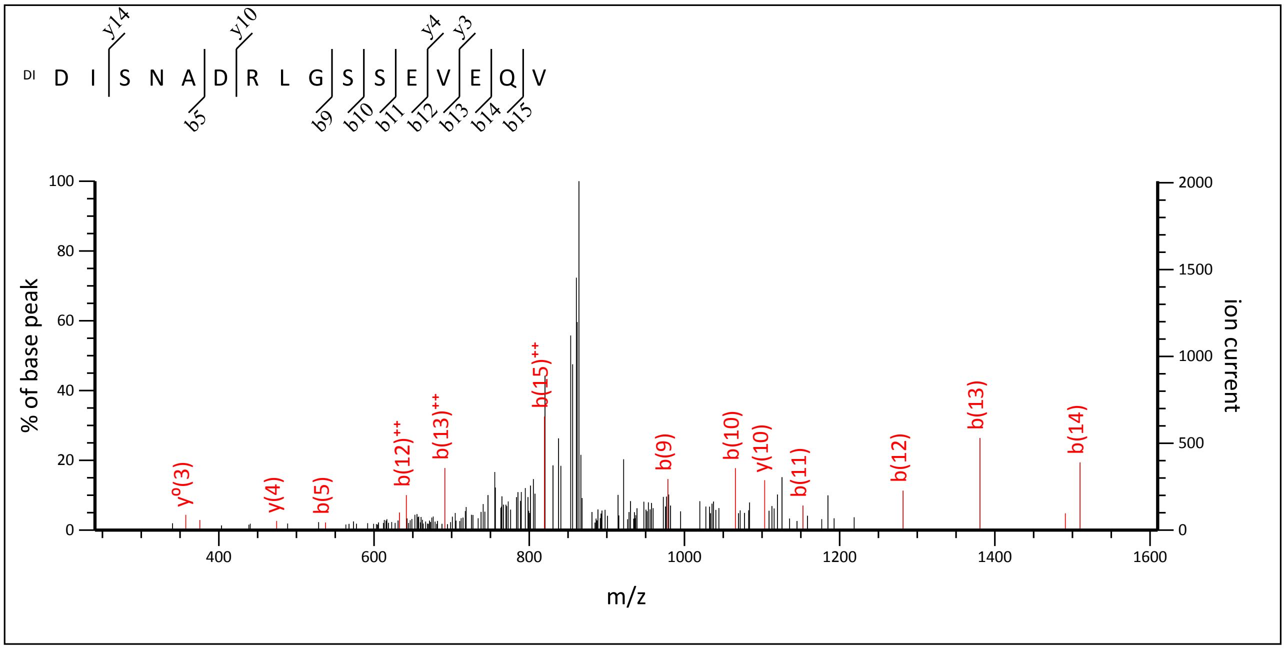

240.37 to 1609.83

Label all possible matches ○ Label matches used for scoring ◉

Monoisotopic mass of neutral peptide Mr(calc): 1753.8875
Variable modifications:
N-term : Dimethyl:2H(6)13C(2) (N-term)
Ions Score: 34 **Expect:** 0.46
Matches : 18/168 fragment ions using 31 most intense peaks (help)

#	b	b⁺⁺	b*	b*⁺⁺	b⁰	b⁰⁺⁺	Seq.	y	y⁺⁺	y*	y*⁺⁺	y⁰	y⁰⁺⁺	#
1	152.1099	76.5586			134.0993	67.5533	D							16
2	265.1940	133.1006			247.1834	124.0953	I	1603.7922	802.3997	1586.7657	793.8865	1585.7816	793.3945	15
3	352.2260	176.6166			334.2154	167.6113	S	***1490.7081***	745.8577	1473.6816	737.3444	1472.6976	736.8524	14
4	466.2689	233.6381	449.2424	225.1248	448.2583	224.6328	N	1403.6761	702.3417	1386.6496	693.8284	1385.6655	693.3364	13
5	***537.3060***	269.1566	520.2795	260.6434	519.2955	260.1514	A	1289.6332	645.3202	1272.6066	636.8070	1271.6226	636.3149	12
6	652.3330	326.6701	635.3064	318.1568	634.3224	317.6648	D	1218.5961	609.8017	1201.5695	601.2884	1200.5855	600.7964	11
7	808.4341	404.7207	791.4075	396.2074	790.4235	395.7154	R	***1103.5691***	552.2882	1086.5426	543.7749	1085.5586	543.2829	10
8	921.5181	461.2627	904.4916	452.7494	903.5076	452.2574	L	947.4680	474.2376	930.4415	465.7244	929.4575	465.2324	9
9	***978.5396***	489.7734	961.5131	481.2602	960.5290	480.7682	G	834.3840	417.6956	817.3574	409.1823	816.3734	408.6903	8
10	***1065.5716***	533.2895	1048.5451	524.7762	1047.5611	524.2842	S	777.3625	389.1849	760.3359	380.6716	759.3519	380.1796	7
11	***1152.6037***	576.8055	1135.5771	568.2922	1134.5931	567.8002	S	690.3305	345.6689	673.3039	337.1556	672.3199	336.6636	6
12	***1281.6463***	**641.3268**	1264.6197	632.8135	1263.6357	632.3215	E	603.2984	302.1529	586.2719	293.6396	585.2879	293.1476	5
13	***1380.7147***	**690.8610**	1363.6881	682.3477	1362.7041	681.8557	V	***474.2558***	237.6316	457.2293	229.1183	456.2453	228.6263	4
14	***1509.7573***	755.3823	1492.7307	746.8690	1491.7467	746.3770	E	***375.1874***	188.0974	358.1609	179.5841	357.1769	179.0921	3

**15**	1637.8158	**819.4116**	1620.7893	810.8983	1619.8053	810.4063	**Q**	246.1448	123.5761	229.1183	115.0628			**2**
**16**							**V**	118.0863	59.5468					**1**

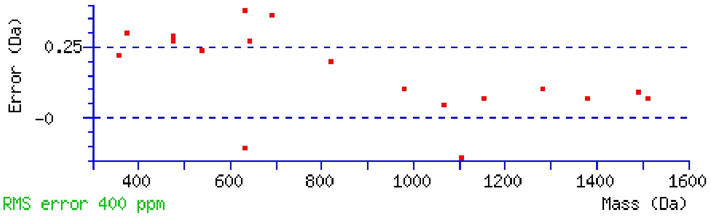

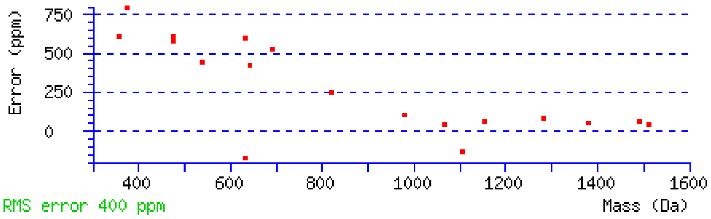

NCBI **BLAST** search of DISNADRLGSSEVEQV
(Parameters: blastp, nr protein database, expect=20000, no filter, PAM30)
Other BLAST web gateways

All matches to this query

Score	Mr(calc)	Delta	Sequence
33.7	1753.8875	0.0029	DISNADRLGSSEVEQV
28.9	1753.8808	0.0097	EDTFKKCSSEVEAK
22.5	1754.0066	-0.1161	ILTFGQVKNSEVEQV
21.3	1753.9308	-0.0404	KGGPGAAVEPSEGEEVQ
20.4	1753.9284	-0.0379	FRDEELKCTVVELN
19.9	1754.0025	-0.1121	NVTAEKEVSTGTVLRS
19.9	1753.7428	0.1477	ENGSDISSAENEQTEA
19.8	1753.9873	-0.0968	GPPGHKGEKGDMVISR
19.5	1753.9826	-0.0921	GLLTTTVTLQQPVELN
19.1	1753.9195	-0.0291	GNEVDGMSRPLRPRP

Mascot: http://www.matrixscience.com/

DISNADRLGSSEVEQV

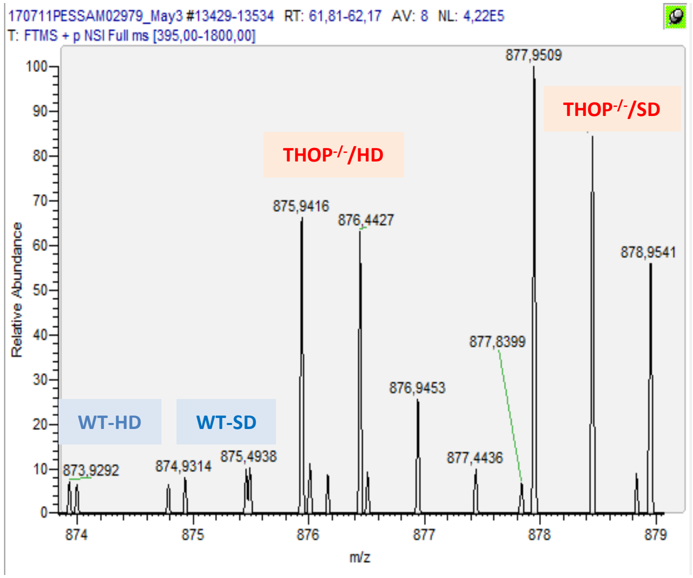

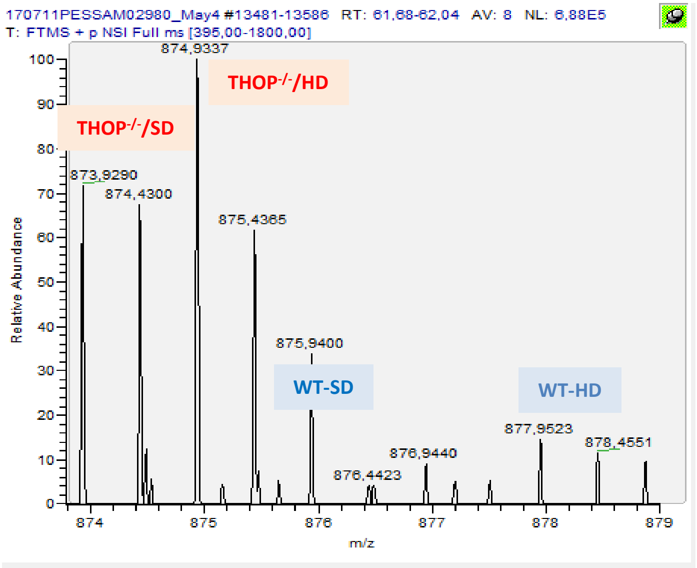

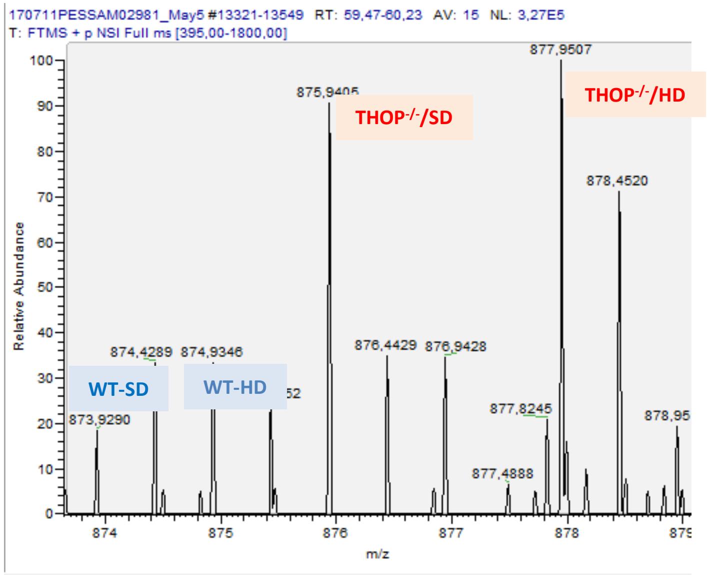

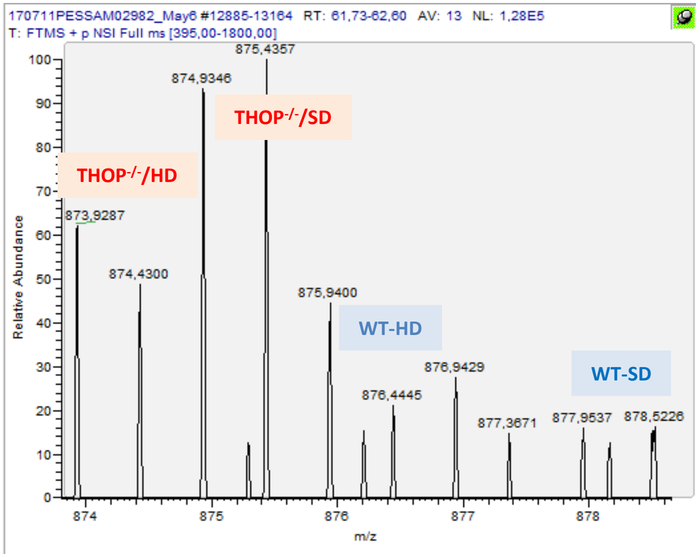

DISNADRLGSSEVEQV

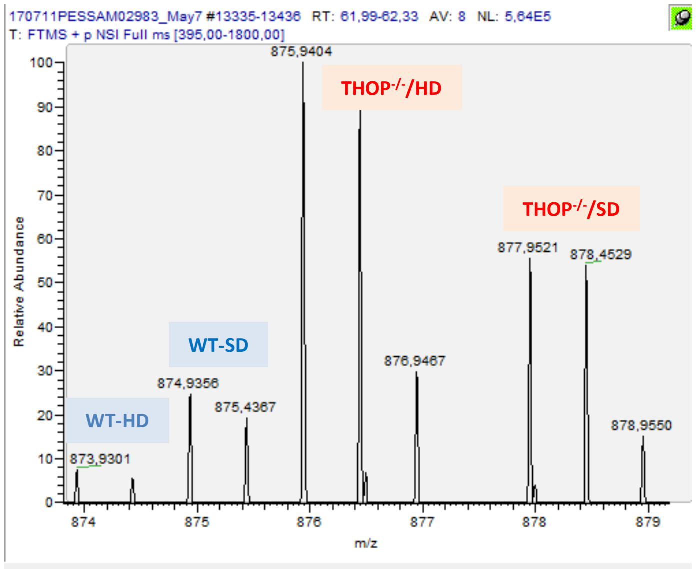

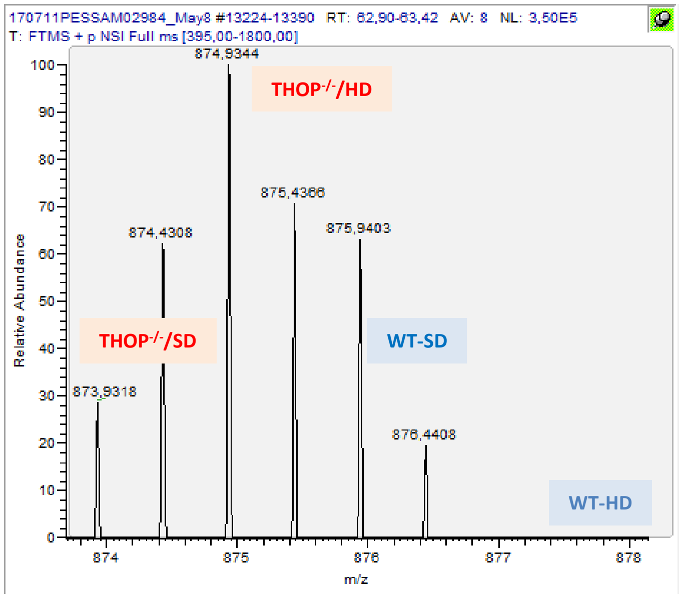

Mascot Search Results

Peptide View

MS/MS Fragmentation of **IDDHFLFDKPVSPLL**
Found in **KCRM_MOUSE** in **SwissProt**, Creatine kinase M-type OS=Mus musculus GN=Ckm PE=1 SV=1

Match to Query 14384: 1827.078528 from(914.546540,2+) intensity(746218.2800) scans(23165-23196) rawscans(sn23165:sn23196) rtinseconds(5986.6983-5996.3487) index(15276)
Title: 15277: Sum of 2 scans in range 23165 (rt=5986.7) to 23196 (rt=5996.35) [D:\Users\Mayara - HMS Fusion data\170711PESSAM02977_May1.raw]
Data file 170711PESSAM02977_May1.temp.mgf

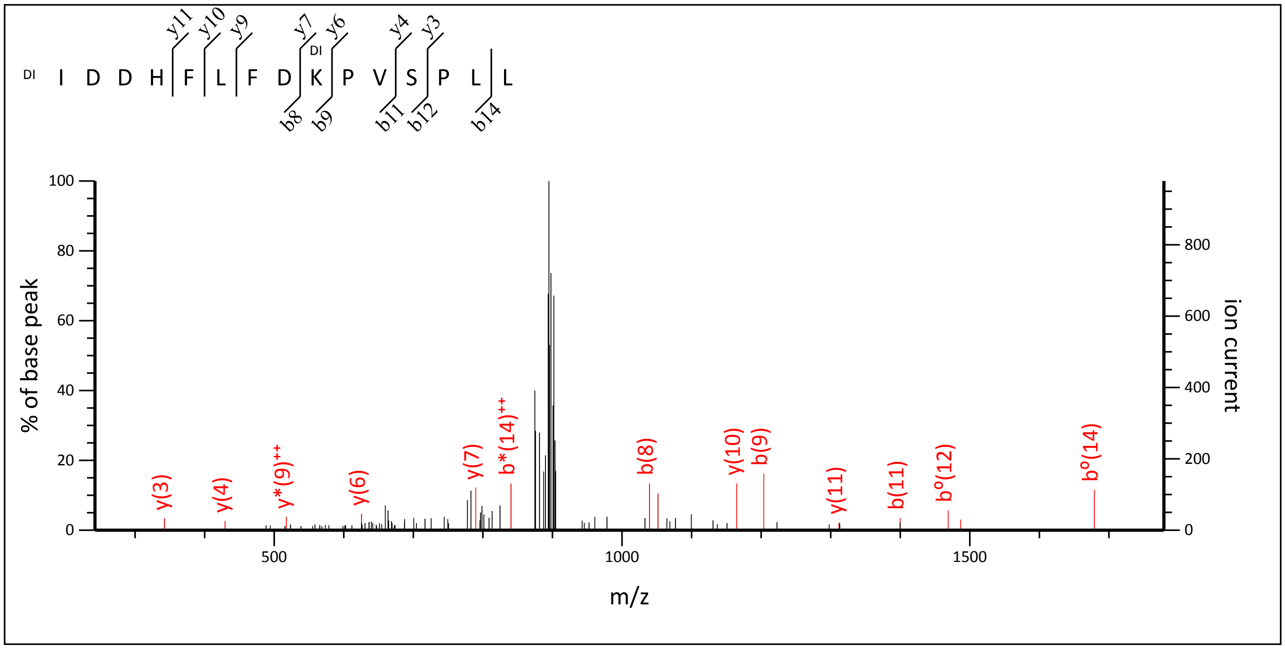

242.33 to 1779

Label all possible matches ○ Label matches used for scoring ◉

Monoisotopic mass of neutral peptide Mr(calc): 1827.0757
Variable modifications:
N-term : Dimethyl:2H(6)13C(2) (N-term)
K9 : Dimethyl:2H(6)13C(2) (K)
Ions Score: 46 **Expect:** 0.0018
Matches : 20/132 fragment ions using 25 most intense peaks (help)

#	b	b^{++}	b*	b^{*++}	b^0	b^{0++}	Seq.	y	y^{++}	y*	y^{*++}	y^0	y^{0++}	#
1	150.1670	75.5871					I							15
2	265.1940	133.1006			247.1834	124.0953	D	***1678.9232***	839.9652	1661.8967	831.4520	1660.9126	830.9600	14
3	380.2209	190.6141			362.2103	181.6088	D	1563.8963	782.4518	1546.8697	773.9385	1545.8857	773.4465	13
4	**517.2798**	259.1435			499.2692	250.1383	H	1448.8693	724.9383	1431.8428	716.4250	1430.8588	715.9330	12
5	664.3482	332.6777			646.3377	323.6725	F	***1311.8104***	656.4088	1294.7839	647.8956	1293.7998	647.4036	11
6	777.4323	389.2198			759.4217	380.2145	L	***1164.7420***	582.8746	1147.7154	574.3614	1146.7314	573.8694	10
7	924.5007	462.7540			906.4901	453.7487	F	***1051.6579***	526.3326	1034.6314	517.8193	1033.6474	517.3273	9
8	**1039.5276**	520.2675			1021.5171	511.2622	D	904.5895	452.7984	887.5630	444.2851	886.5789	443.7931	8
9	**1203.6983**	602.3528	1186.6717	593.8395	1185.6877	593.3475	K	***789.5626***	395.2849	772.5360	386.7716	771.5520	386.2796	7
10	1300.7510	650.8792	1283.7245	642.3659	1282.7405	641.8739	P	***625.3919***	313.1996			607.3814	304.1943	6
11	**1399.8195**	700.4134	1382.7929	691.9001	1381.8089	691.4081	V	528.3392	264.6732			510.3286	255.6679	5
12	**1486.8515**	743.9294	1469.8249	735.4161	1468.8409	734.9241	S	***429.2708***	215.1390			411.2602	206.1337	4
13	1583.9042	792.4558	1566.8777	783.9425	1565.8937	783.4505	P	***342.2387***	171.6230					3

14	1696.9883	848.9978	1679.9618	840.4845	1678.9777	839.9925	L	245.1860	123.0966					2
15							L	132.1019	66.5546					1

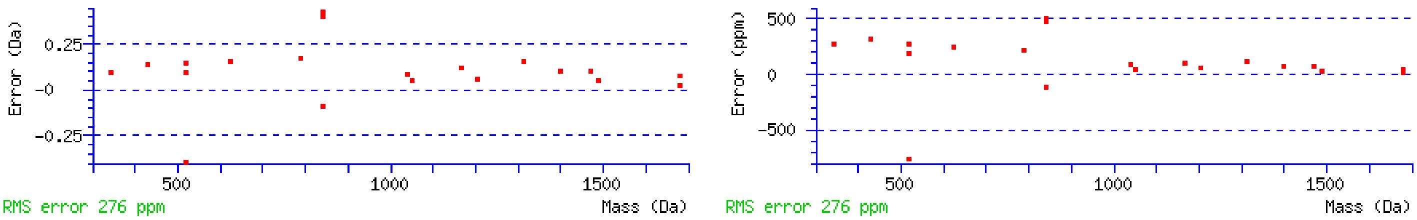

NCBI **BLAST** search of IDDHFLFDKPVSPLL
(Parameters: blastp, nr protein database, expect=20000, no filter, PAM30)
Other BLAST web gateways

All matches to this query

Score	Mr(calc)	Delta	Sequence
46.4	1827.0757	0.0028	IDDHFLFDKPVSPLL
16.2	1827.1069	-0.0284	QLLSKTPGSRSQPLPL
15.8	1827.0717	0.0068	PVDAKVGLGISQSSSLLP
15.1	1826.9560	0.1225	LCPSVPSNSVSQLQKAN
12.6	1826.8972	0.1813	PASSASFVASTESMPVTL
11.2	1827.0618	0.0167	VTASPVIKAVTNFRQV
11.1	1826.9384	0.1401	IMSIFWGMDMVILAI
11.0	1826.8886	0.1899	MARSRGHLWAYSPEP
10.9	1827.0135	0.0651	ASMPDNTAAQKVSHLLG
10.9	1826.9978	0.0807	LITEAQTQASGAGNKFQ

Mascot: http://www.matrixscience.com/

IDDHFLFDKPVSPLL

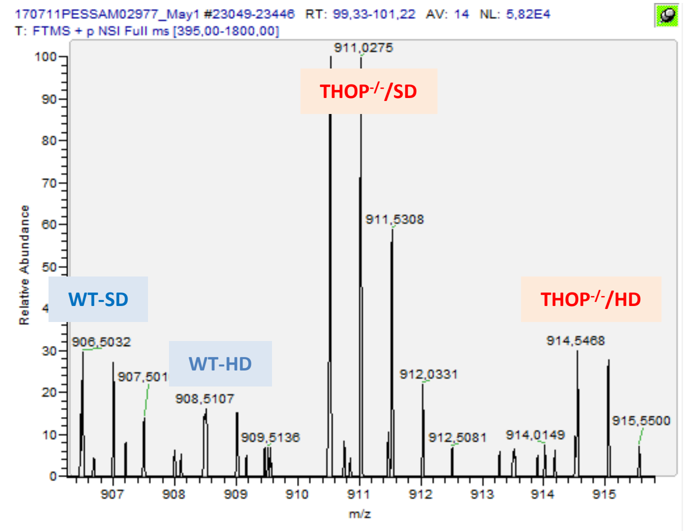

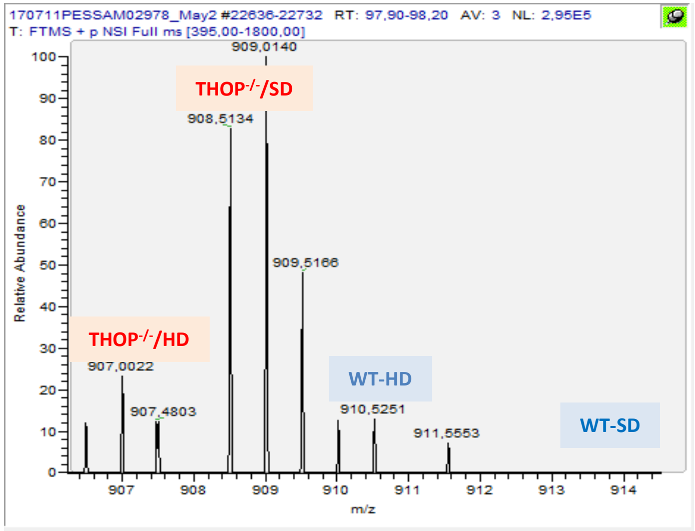

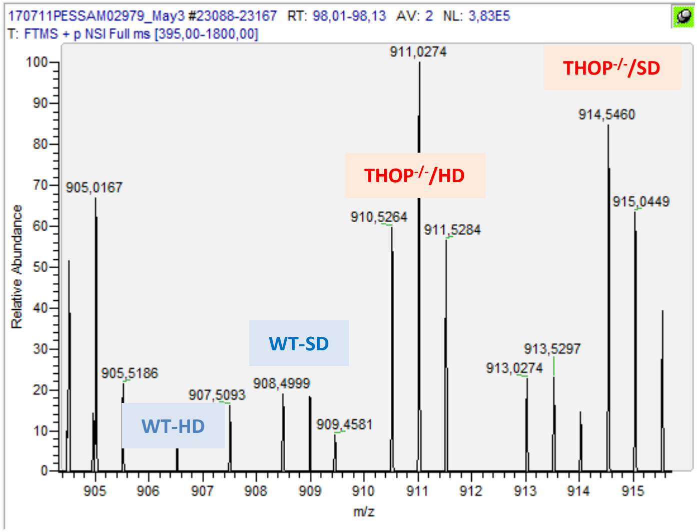

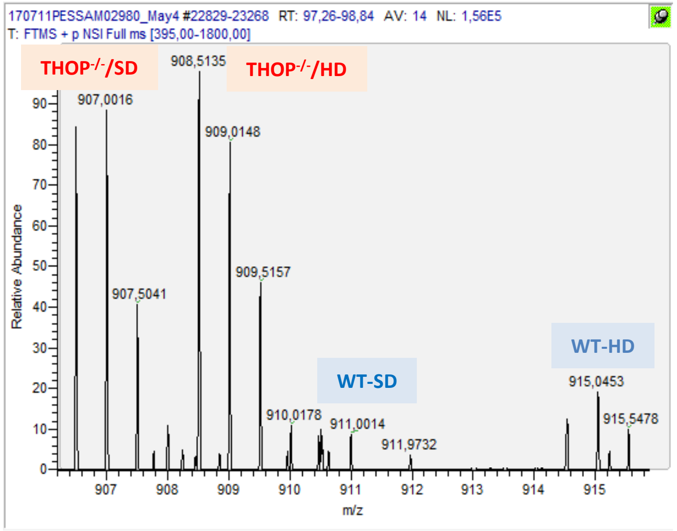

IDDHFLFDKPVSPLL

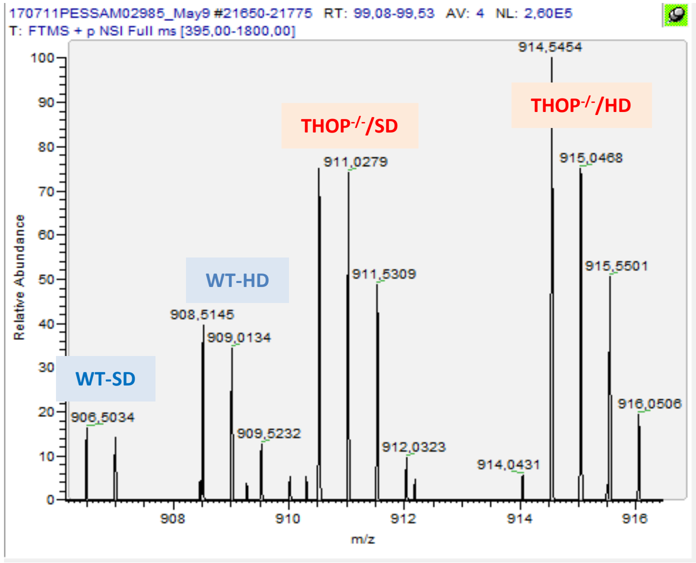

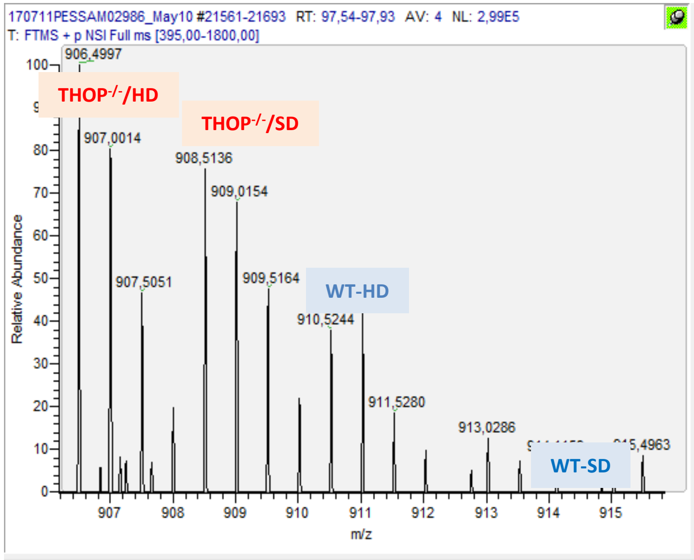

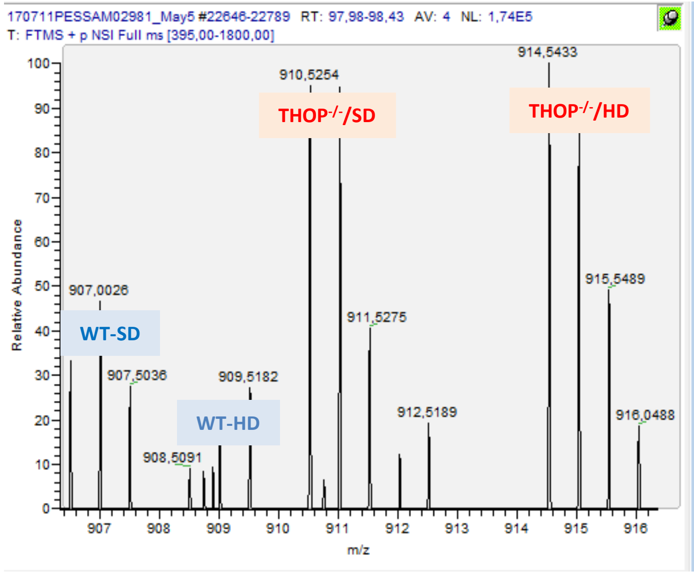

May6 –not found

IDDHFLFDKPVSPLL

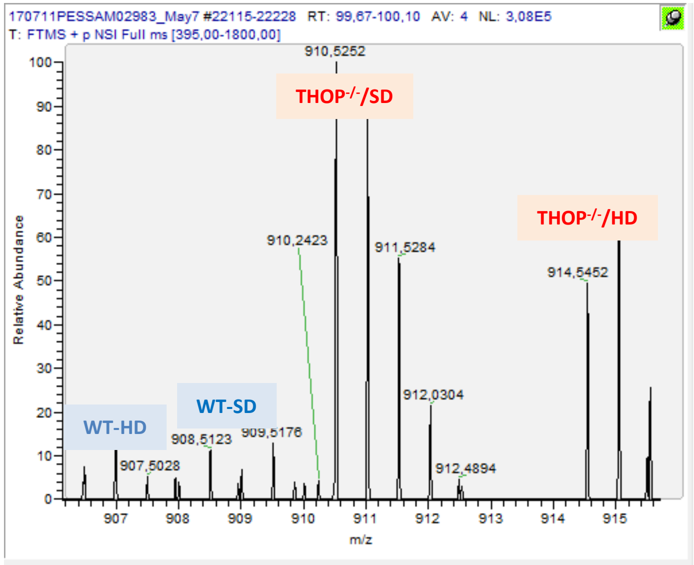

May8 –not found

Mascot Search Results

Peptide View

MS/MS Fragmentation of **LETLKTQVQSVIDKA**
Found in **APOA1_MOUSE** in **SwissProt**, Apolipoprotein A-I OS=Mus musculus GN=Apoa1 PE=1 SV=2

Match to Query 13752: 1762.070802 from(588.364210,3+) intensity(4227358.0000) scans(16013) rawscans(sn16013) rtinseconds(4398.351) index(10848)
Title: 10849: Scan 16013 (rt=4398.35) [D:\Users\Mayara - HMS Fusion data\170711PESSAM02985_May9.raw]
Data file 170711PESSAM02985_May9.temp.mgf

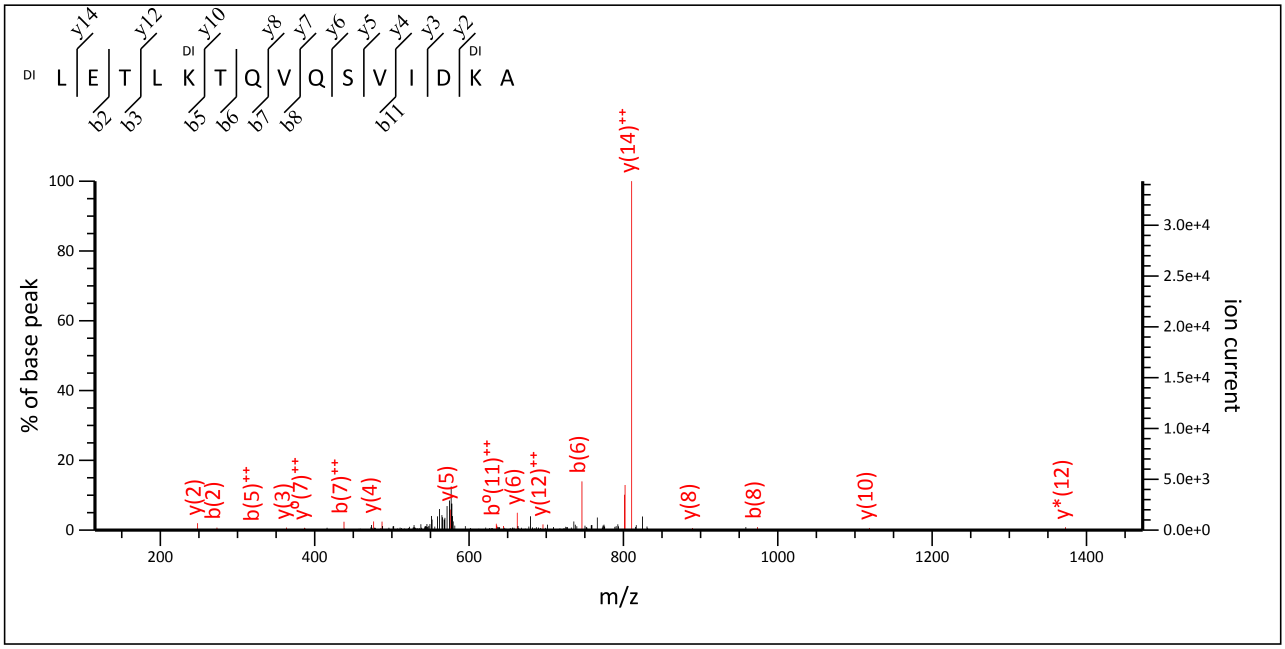

115.3 to 1472.81

Label all possible matches Label matches used for scoring

Monoisotopic mass of neutral peptide Mr(calc): 1762.0815
Variable modifications:
N-term : Dimethyl:2H(2) (N-term)
K5 : Dimethyl:2H(2) (K)
K14 : Dimethyl:2H(2) (K)
Ions Score: 38 **Expect:** 0.015
Matches : 30/152 fragment ions using 56 most intense peaks (help)

#	b	b^{++}	b*	b^{*++}	b^{0}	b^{0++}	Seq.	y	y^{++}	y*	y^{*++}	y^{0}	y^{0++}	#
1	144.1383	72.5728					L							15
2	273.1809	137.0941			255.1703	128.0888	E	1619.9578	810.4825	1602.9313	801.9693	1601.9473	801.4773	14
3	374.2286	187.6179			356.2180	178.6126	T	1490.9152	745.9612	1473.8887	737.4480	1472.9047	736.9560	13
4	487.3126	244.1600			469.3021	235.1547	L	1389.8675	695.4374	1372.8410	686.9241	1371.8570	686.4321	12
5	645.4545	323.2309	628.4280	314.7176	627.4440	314.2256	K	1276.7835	638.8954	1259.7569	630.3821	1258.7729	629.8901	11
6	746.5022	373.7547	729.4757	365.2415	728.4917	364.7495	T	1118.6416	559.8244	1101.6150	551.3111	1100.6310	550.8191	10
7	874.5608	437.7840	857.5342	429.2708	856.5502	428.7788	Q	1017.5939	509.3006	1000.5673	500.7873	999.5833	500.2953	9
8	973.6292	487.3182	956.6027	478.8050	955.6186	478.3130	V	889.5353	445.2713	872.5088	436.7580	871.5247	436.2660	8
9	1101.6878	551.3475	1084.6612	542.8343	1083.6772	542.3422	Q	790.4669	395.7371	773.4403	387.2238	772.4563	386.7318	7
10	1188.7198	594.8635	1171.6933	586.3503	1170.7093	585.8583	S	662.4083	331.7078	645.3818	323.1945	644.3978	322.7025	6
11	1287.7882	644.3978	1270.7617	635.8845	1269.7777	635.3925	V	575.3763	288.1918	558.3497	279.6785	557.3657	279.1865	5
12	1400.8723	700.9398	1383.8457	692.4265	1382.8617	691.9345	I	476.3079	238.6576	459.2813	230.1443	458.2973	229.6523	4
13	1515.8992	758.4533	1498.8727	749.9400	1497.8887	749.4480	D	363.2238	182.1155	346.1973	173.6023	345.2132	173.1103	3

14	1674.0412	837.5242	1657.0146	829.0109	1656.0306	828.5189	K	*248.1969*	124.6021	231.1703	116.0888			2
15							A	90.0550	45.5311					1

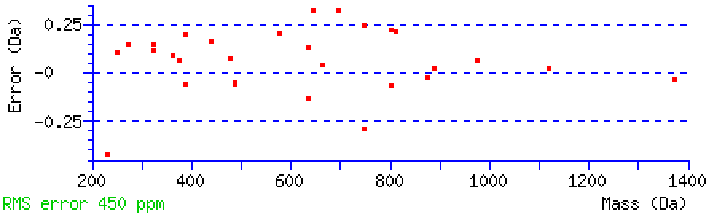

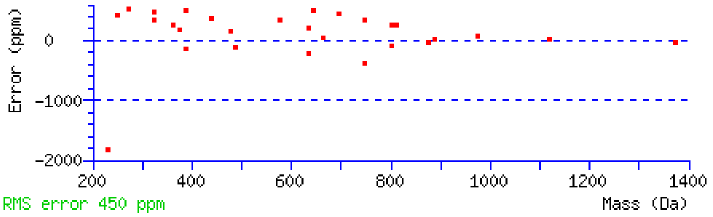

NCBI **BLAST** search of LETLKTQVQSVIDKA
(Parameters: blastp, nr protein database, expect=20000, no filter, PAM30)
Other BLAST web gateways

All matches to this query

Score	Mr(calc)	Delta	Sequence
38.2	1762.0815	-0.0107	LETLKTQVQSVIDKA
18.3	1761.9625	0.1083	LEITQDSHKGVPLEH
17.4	1762.1008	-0.0300	LAGLGLGLSLIFIAVYL
16.6	1762.0829	-0.0121	NNLQLLSQVLPRLKA
16.5	1762.0136	0.0572	LAIVALISIIYMMLH
16.3	1762.0829	-0.0121	IHKAASVGDVAKVQHI
16.2	1761.9036	0.1672	ASGLPLVTEETPPPPGE
16.2	1761.9108	0.1600	AEPAGLSLRADSDSGSK
16.1	1761.8818	0.1890	GGSMPSSAAISQSLSELL
16.0	1762.0262	0.0446	SAGAVTVLKAPNRELM

Mascot: http://www.matrixscience.com/

LETLKTQVQSVIDKA

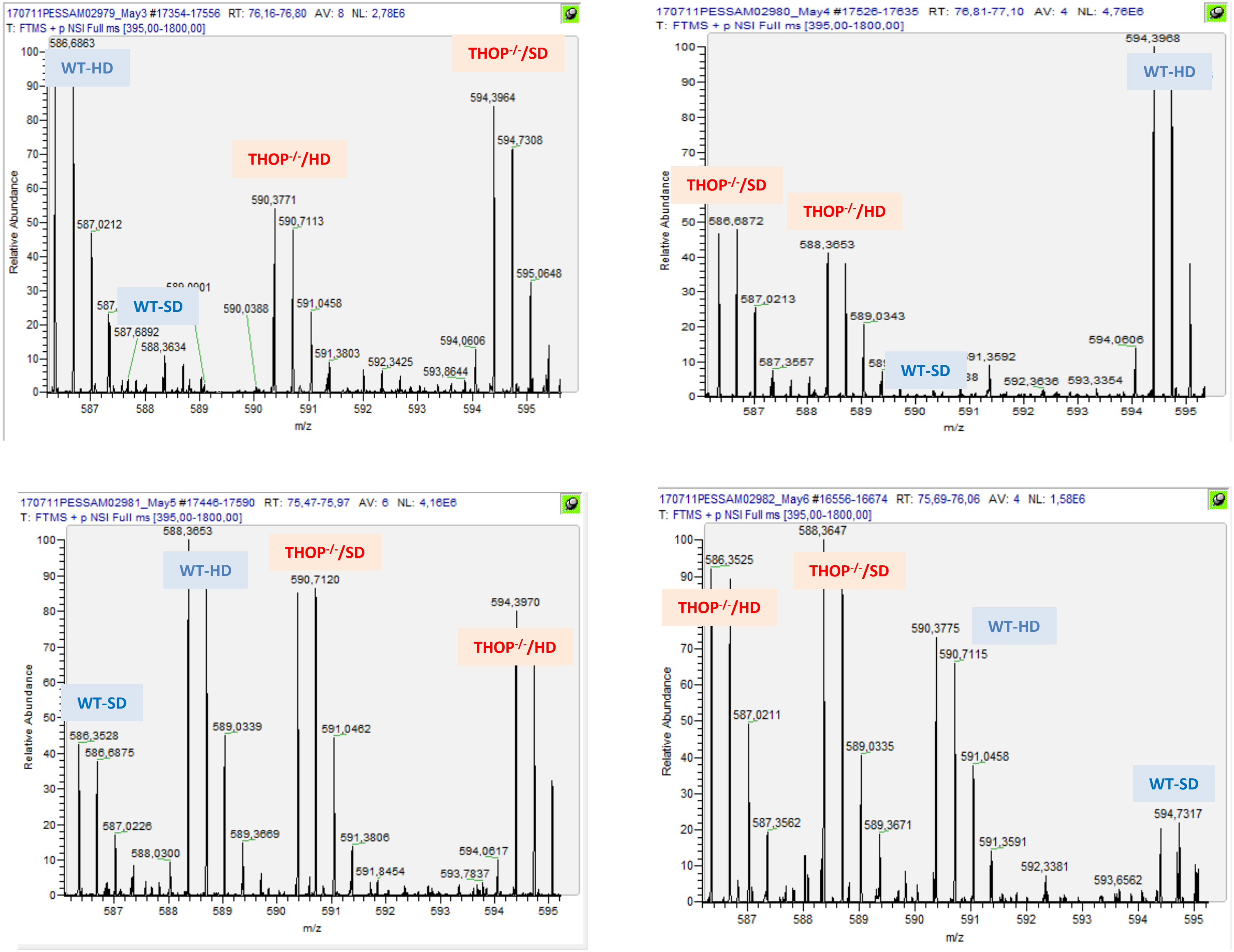

LETLKTQVQSVIDKA

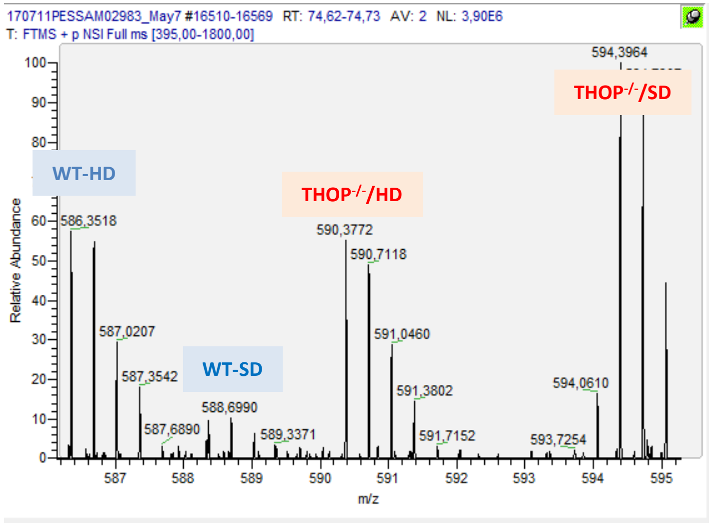

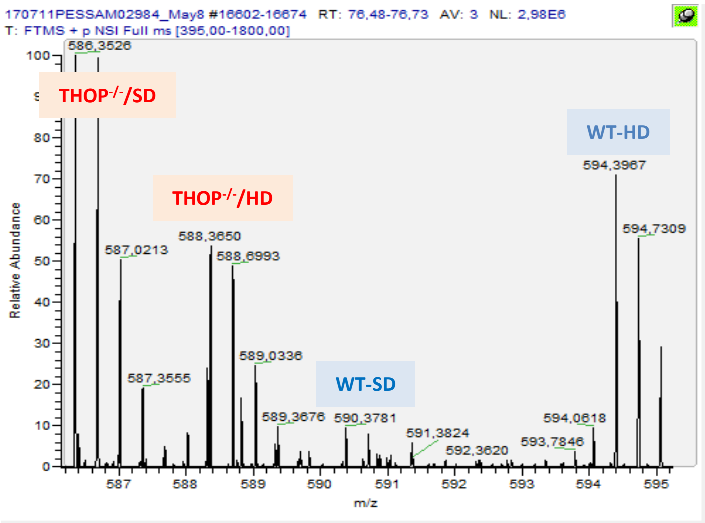

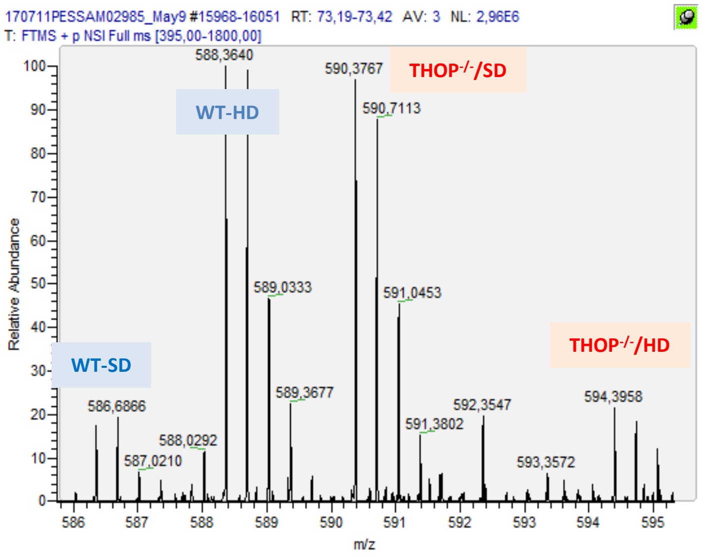

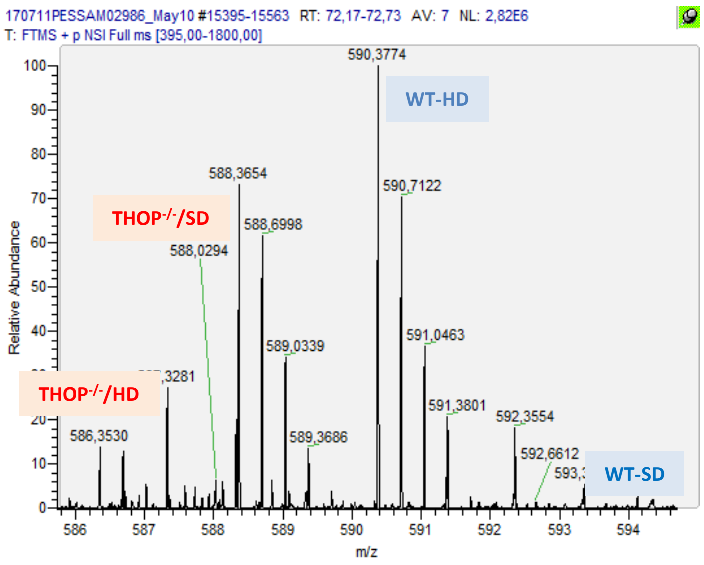

Mascot Search Results

Peptide View

MS/MS Fragmentation of **FSSLMNLEEKPAPAA**
Found in **APOA2_MOUSE** in **SwissProt**, Apolipoprotein A-II OS=Mus musculus GN=Apoa2 PE=1 SV=2

Match to Query 12466: 1675.941268 from(838.977910,2+) intensity(1987756.5000) scans(16579) rawscans(sn16579) rtinseconds(4288.0265) index(10657)
Title: 10658: Scan 16579 (rt=4288.03) [D:\Users\Mayara - HMS Fusion data\170711PESSAM02977_May1.raw]
Data file 170711PESSAM02977_May1.temp.mgf

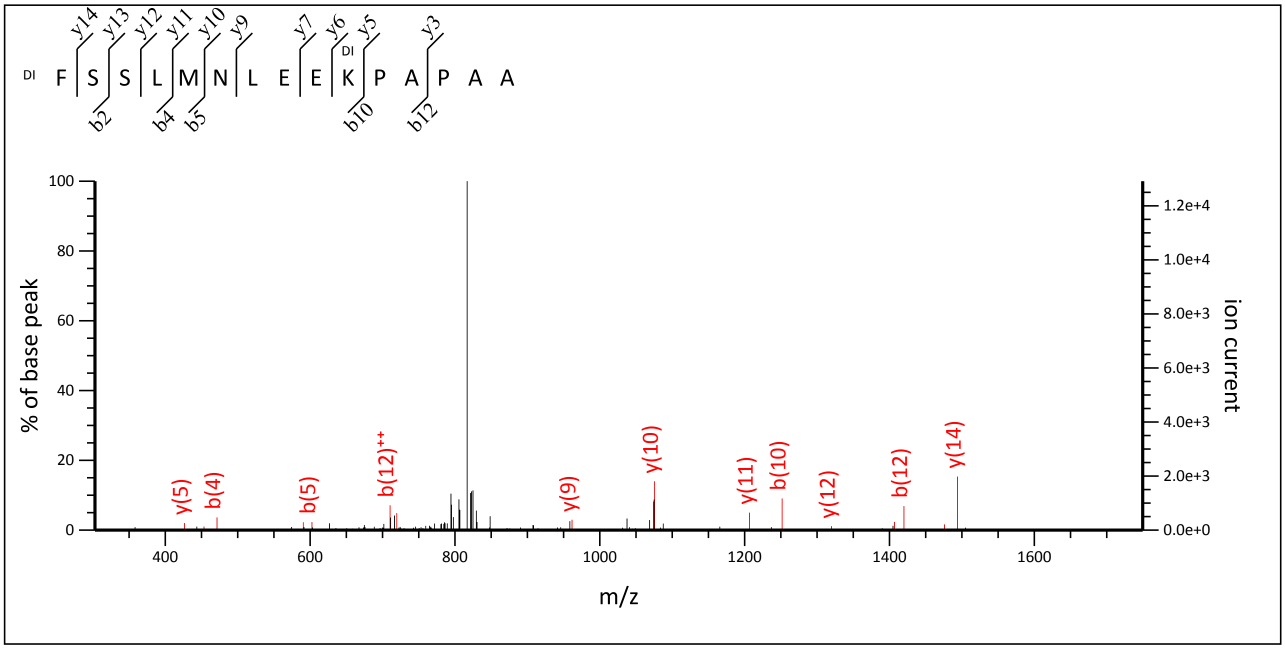

302.9 to 1749.64

Label all possible matches ○ Label matches used for scoring ◉

Monoisotopic mass of neutral peptide Mr(calc): 1675.9429
Variable modifications:
N-term : Dimethyl:2H(6)13C(2) (N-term)
K10 : Dimethyl:2H(6)13C(2) (K)
Ions Score: 74 **Expect:** 6.1e-006
Matches : 19/134 fragment ions using 25 most intense peaks (help)

#	b	b^{++}	b*	b*$^{++}$	b^{0}	b^{0++}	Seq.	y	y^{++}	y*	y*$^{++}$	y^{0}	y^{0++}	#
1	184.1514	92.5793					F							15
2	**271.1834**	136.0953			253.1728	127.0900	S	***1493.8061***	747.4067	1476.7796	738.8934	1475.7956	738.4014	14
3	358.2154	179.6113			340.2049	170.6061	S	***1406.7741***	703.8907	1389.7475	695.3774	1388.7635	694.8854	13
4	**471.2995**	236.1534			453.2889	227.1481	L	***1319.7421***	660.3747	1302.7155	651.8614	1301.7315	651.3694	12
5	**602.3400**	301.6736			584.3294	292.6683	M	***1206.6580***	603.8326	1189.6315	595.3194	1188.6474	594.8274	11
6	716.3829	358.6951	699.3563	350.1818	698.3723	349.6898	N	***1075.6175***	538.3124	1058.5910	529.7991	1057.6070	529.3071	10
7	829.4670	415.2371	812.4404	406.7238	811.4564	406.2318	L	***961.5746***	481.2909	944.5480	472.7777	943.5640	472.2857	9
8	958.5095	479.7584	941.4830	471.2451	940.4990	470.7531	E	848.4905	424.7489	831.4640	416.2356	830.4800	415.7436	8
9	1087.5521	544.2797	1070.5256	535.7664	1069.5416	535.2744	E	***719.4479***	360.2276	702.4214	351.7143	701.4374	351.2223	7
10	**1251.7228**	626.3650	1234.6962	617.8518	1233.7122	617.3597	K	***590.4053***	295.7063	573.3788	287.1930			6
11	1348.7755	674.8914	1331.7490	666.3781	1330.7650	665.8861	P	***426.2347***	213.6210					5
12	**1419.8127**	710.4100	1402.7861	701.8967	1401.8021	701.4047	A	329.1819	165.0946					4
13	1516.8654	758.9363	1499.8389	750.4231	1498.8549	749.9311	P	***258.1448***	129.5761					3

14	1587.9025	794.4549	1570.8760	785.9416	1569.8920	785.4496	A	161.0921	81.0497					2
15							A	90.0550	45.5311					1

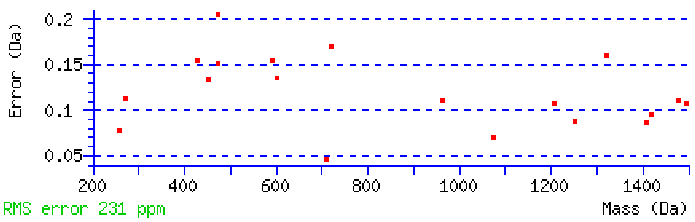

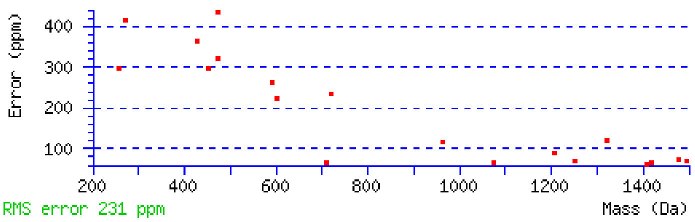

NCBI **BLAST** search of FSSLMNLEEKPAPAA
(Parameters: blastp, nr protein database, expect=20000, no filter, PAM30)
Other BLAST web gateways

All matches to this query

Score	Mr(calc)	Delta	Sequence
74.2	1675.9429	-0.0016	FSSLMNLEEKPAPAA
18.0	1675.6775	0.2638	FSSMIADDMSNYDE
15.8	1675.8095	0.1317	ELSLCHITGMREML
15.3	1675.7402	0.2011	HGGGTGGGGGAGGQAMDPAA
15.3	1675.8516	0.0897	EISLPTTSTIIDGSQS
14.4	1675.9007	0.0405	ILCLIYTLNFAIDH
14.4	1675.9912	-0.0500	LKHLIIENFIPLE
14.1	1675.9468	-0.0056	ASASLSGTSALTGLTKR
13.9	1675.8095	0.1317	ELSLCHITGMREML
13.2	1675.8473	0.0940	RKNMPRCIYCIHA

Mascot: http://www.matrixscience.com/

FSSLMNLEEKPAPAA

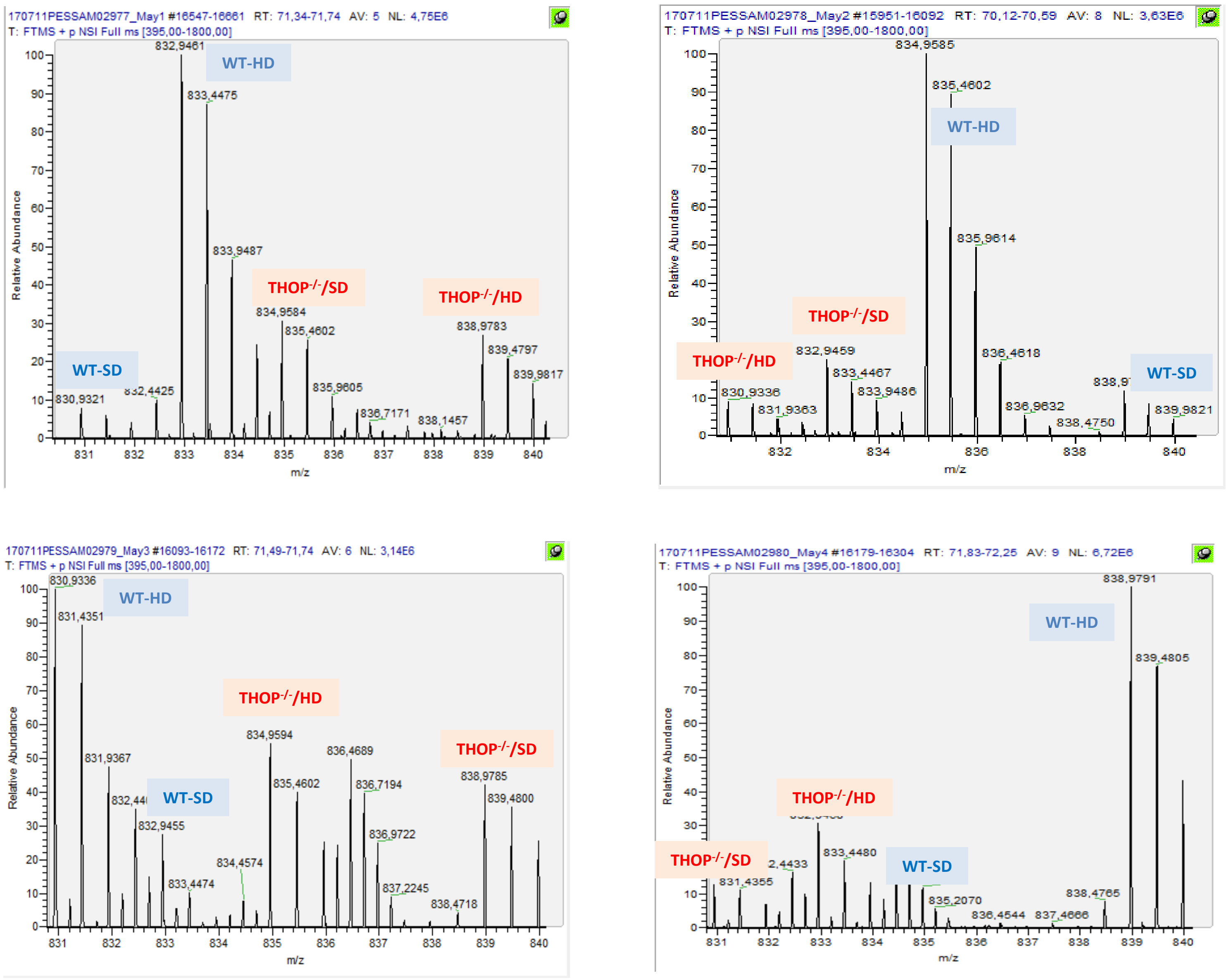

FSSLMNLEEKPAPAA

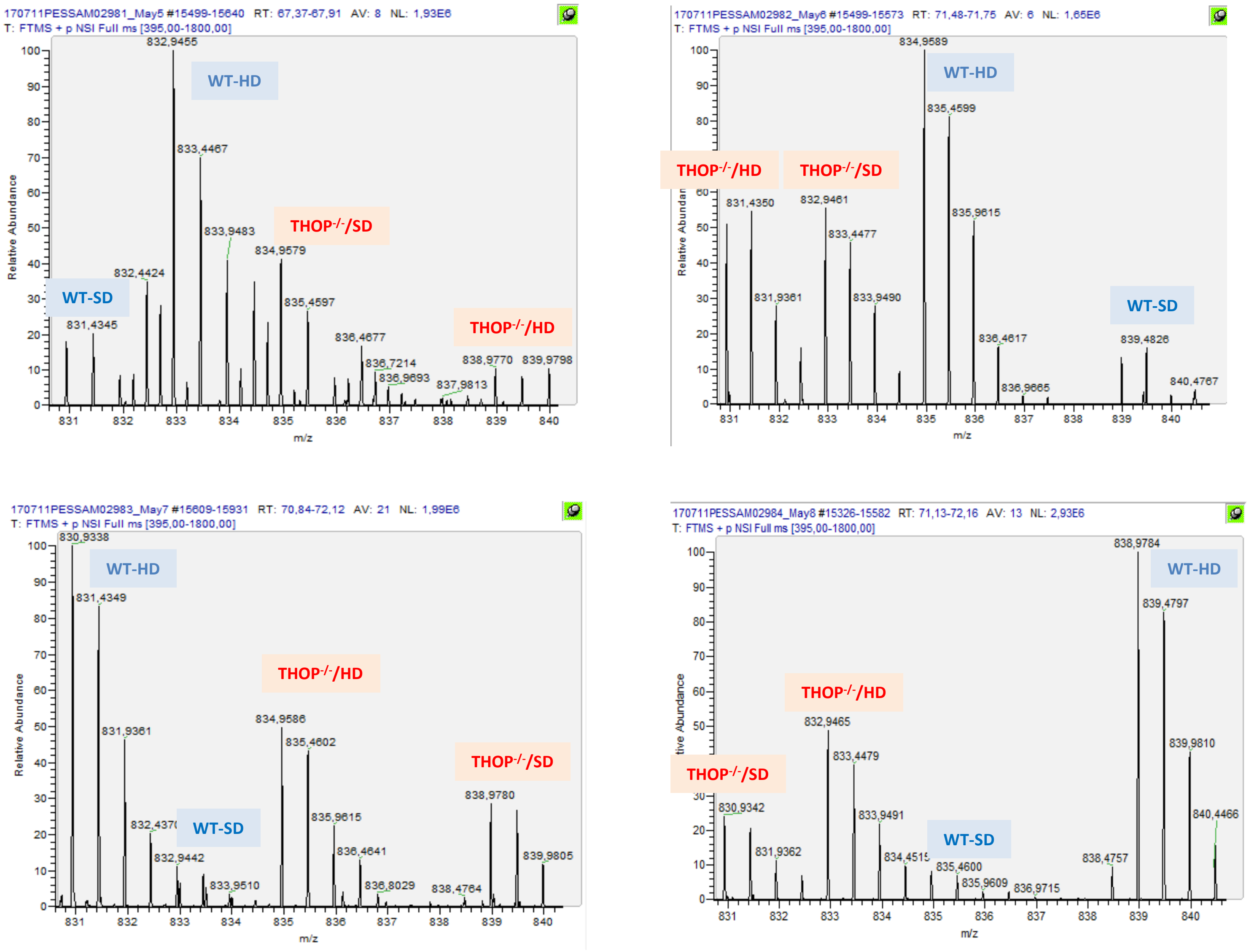

FSSLMNLEEKPAPAA

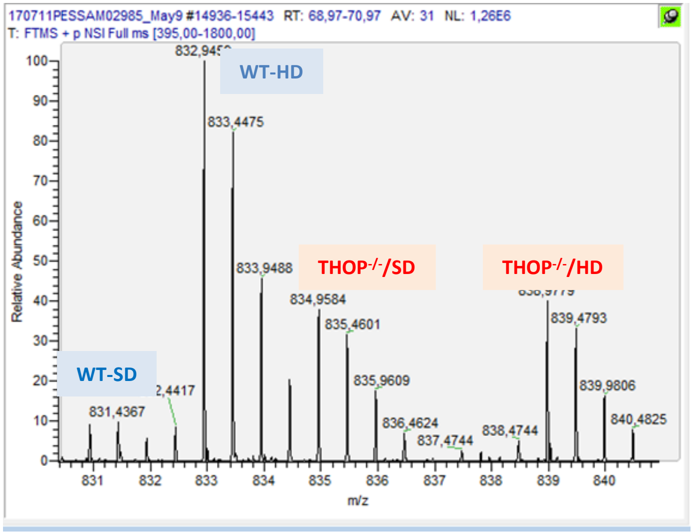

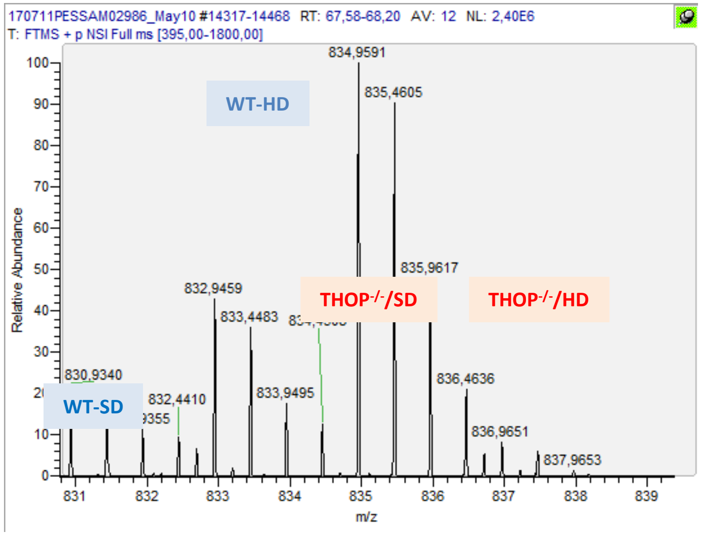

Mascot Search Results

Peptide View

MS/MS Fragmentation of **HEQLTPLVRSAGTSLVN**
Found in **APOA2_MOUSE** in **SwissProt**, Apolipoprotein A-II OS=Mus musculus GN=Apoa2 PE=1 SV=2

Match to Query 14687: 1849.006168 from(925.510360,2+) intensity(1484091.9000) scans(12825) rawscans(sn12825) rtinseconds(3595.332) index(8313)
Title: 8314: Scan 12825 (rt=3595.33) [D:\Users\Mayara - HMS Fusion data\170711PESSAM02983_May7.raw]
Data file 170711PESSAM02983_May7.temp.mgf

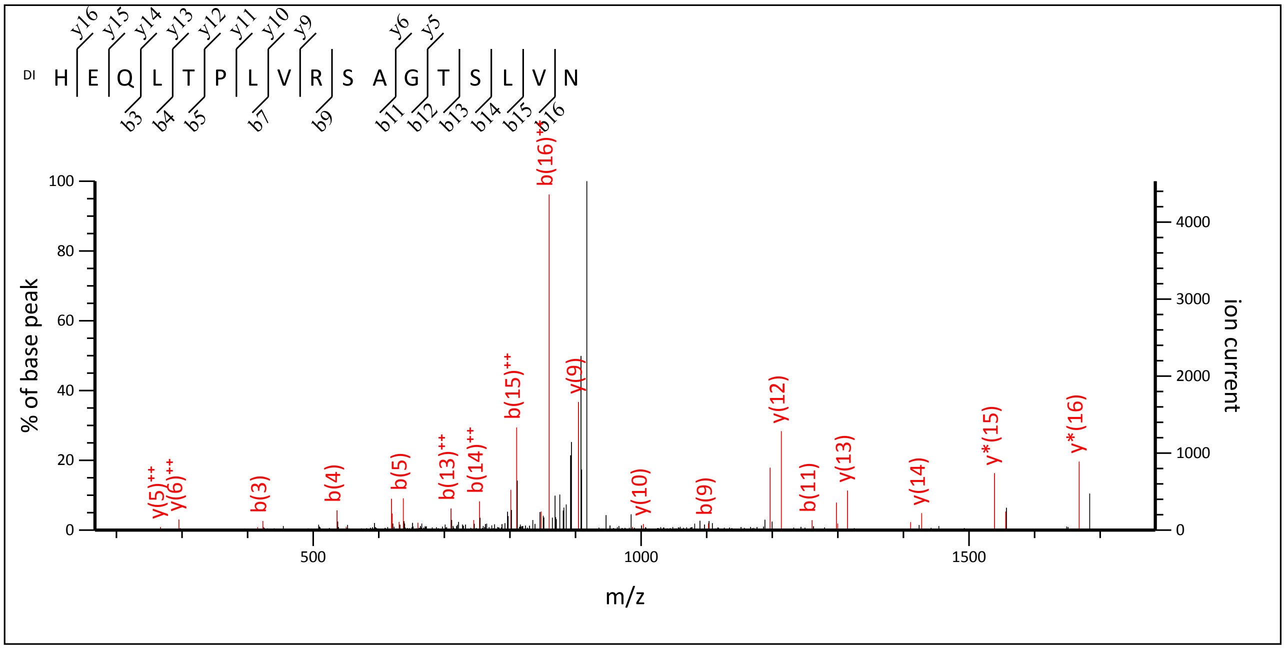

167.25 to 1784.03

Label all possible matches ○ Label matches used for scoring ◉

Monoisotopic mass of neutral peptide Mr(calc): 1849.0058
Variable modifications:
N-term : Dimethyl (N-term)
Ions Score: 39 **Expect:** 0.0088
Matches : 41/180 fragment ions using 77 most intense peaks (help)

#	b	b++	b*	b*++	b0	b0++	Seq.	y	y++	y*	y*++	y0	y0++	#
1	166.0975	83.5524					H							17
2	***295.1401***	148.0737			277.1295	139.0684	E	1684.9228	842.9651	1667.8963	834.4518	1666.9123	833.9598	16
3	***423.1987***	212.1030	406.1721	203.5897	405.1881	203.0977	Q	**1555.8802**	778.4438	1538.8537	769.9305	1537.8697	769.4385	15
4	***536.2827***	***268.6450***	519.2562	260.1317	518.2722	259.6397	L	**1427.8217**	714.4145	1410.7951	705.9012	1409.8111	705.4092	14
5	***637.3304***	319.1688	620.3039	310.6556	619.3198	310.1636	T	**1314.7376**	657.8724	1297.7110	649.3592	1296.7270	648.8672	13
6	734.3832	367.6952	717.3566	359.1819	716.3726	358.6899	P	**1213.6899**	607.3486	1196.6634	598.8353	1195.6793	598.3433	12
7	***847.4672***	***424.2373***	830.4407	415.7240	829.4567	415.2320	L	1116.6371	558.8222	1099.6106	550.3089	1098.6266	549.8169	11
8	946.5356	473.7715	929.5091	465.2582	928.5251	464.7662	V	**1003.5531**	502.2802	986.5265	493.7669	985.5425	493.2749	10
9	***1102.6368***	551.8220	1085.6102	543.3087	1084.6262	542.8167	R	**904.4847**	452.7460	887.4581	444.2327	886.4741	443.7407	9
10	1189.6688	595.3380	1172.6422	586.8248	1171.6582	586.3327	S	748.3836	374.6954	731.3570	366.1821	730.3730	365.6901	8
11	***1260.7059***	***630.8566***	1243.6793	622.3433	1242.6953	621.8513	A	661.3515	331.1794	644.3250	322.6661	643.3410	322.1741	7
12	1317.7274	***659.3673***	1300.7008	650.8540	1299.7168	650.3620	G	590.3144	295.6608	573.2879	287.1476	572.3039	286.6556	6
13	1418.7750	***709.8912***	1401.7485	701.3779	1400.7645	700.8859	T	533.2930	267.1501	516.2664	258.6368	515.2824	258.1448	5
14	1505.8071	***753.4072***	1488.7805	744.8939	1487.7965	744.4019	S	432.2453	216.6263	415.2187	208.1130	414.2347	207.6210	4

**15**	1618.8911	***809.9492***	1601.8646	801.4359	1600.8806	800.9439	**L**	345.2132	173.1103	328.1867	164.5970			**3**
**16**	1717.9595	***859.4834***	1700.9330	850.9701	1699.9490	850.4781	**V**	232.1292	116.5682	215.1026	108.0550			**2**
**17**							**N**	133.0608	67.0340	116.0342	58.5207			**1**

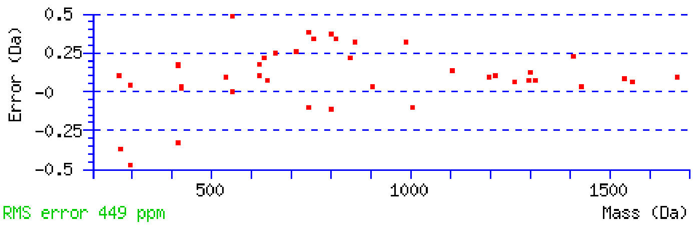

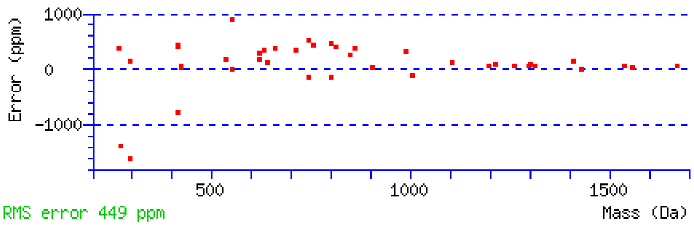

NCBI **BLAST** search of HEQLTPLVRSAGTSLVN
(Parameters: blastp, nr protein database, expect=20000, no filter, PAM30)
Other BLAST web gateways

All matches to this query

Score	Mr(calc)	Delta	Sequence
39.2	1849.0058	0.0004	HEQLTPLVRSAGTSLVN
16.9	1848.9159	0.0903	FGFEQAARDYTLRTF
16.5	1848.9059	0.1002	AISDIMSSDQETYSCK
16.3	1849.0713	-0.0651	PALLLTALEQHVLYLQ
15.4	1848.9846	0.0216	IQQALHRPPSSAAQYL
15.2	1849.0273	-0.0211	EEQLTLVRLSRPCAQA
14.6	1849.0098	-0.0036	VTPATFPRNAQTLQLY
14.5	1849.0814	-0.0752	RDKEFQSHLTSHEK
14.5	1848.8418	0.1644	FSPEDYQSTAGTLEFQ
14.4	1849.1164	-0.1102	LAQALNEALKPTLQYL

Mascot: http://www.matrixscience.com/

HEQLTPLVRSAGTSLVN

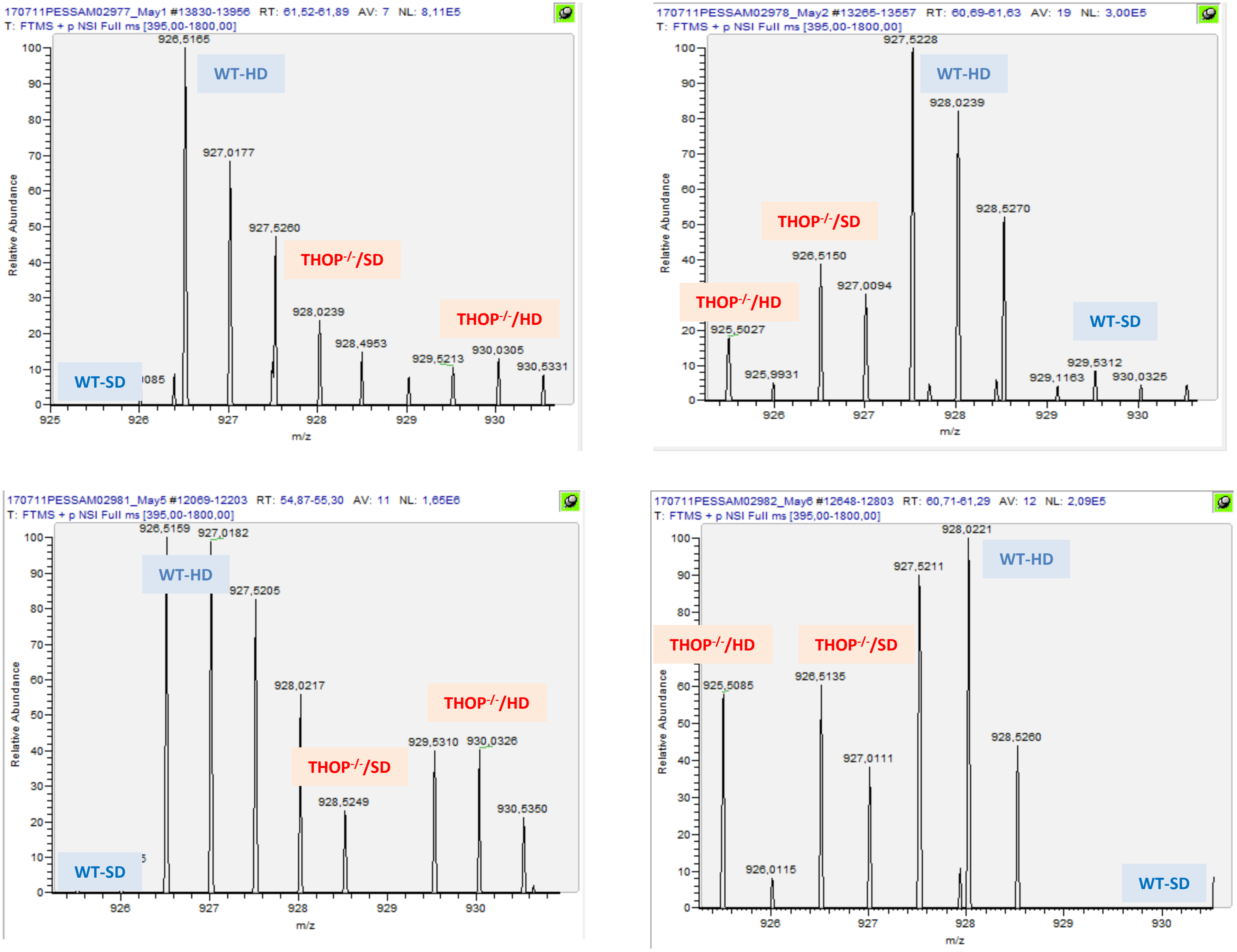

HEQLTPLVRSAGTSLVN

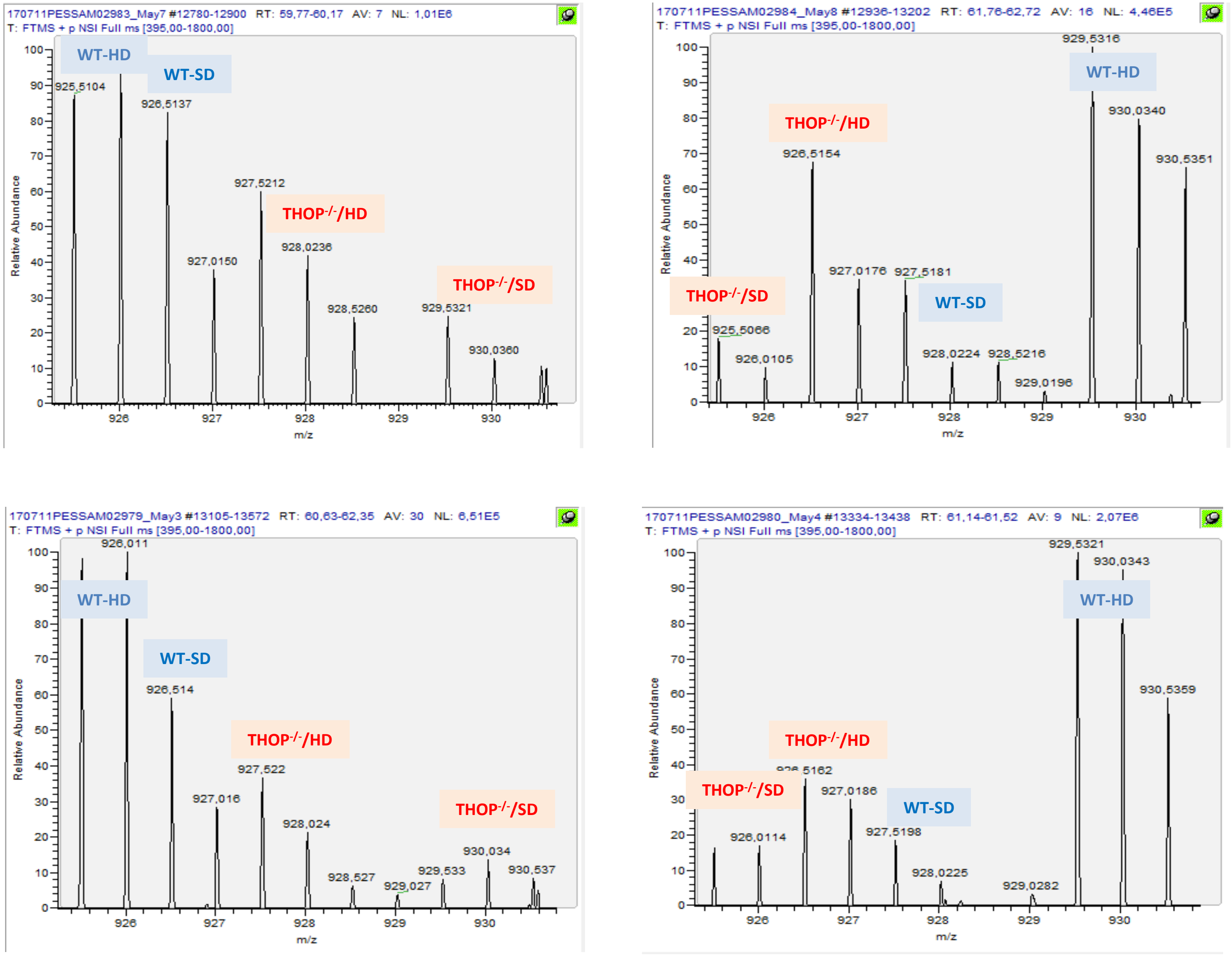

Mascot Search Results

Peptide View

MS/MS Fragmentation of **SQTFPNADFAEITKL**
Found in **ALBU_MOUSE** in **SwissProt**, Serum albumin OS=Mus musculus GN=Alb PE=1 SV=3

Match to Query 13506: 1752.982588 from(877.498570,2+) intensity(2773684.7000) scans(21840-21901) rawscans(sn21840:sn21901) rtinseconds(5595.796-5612.719) index(14323)
Title: 14324: Sum of 3 scans in range 21840 (rt=5595.8) to 21901 (rt=5612.72) [D:\Users\Mayara - HMS Fusion data\170711PESSAM02977_May1.raw]
Data file 170711PESSAM02977_May1.temp.mgf

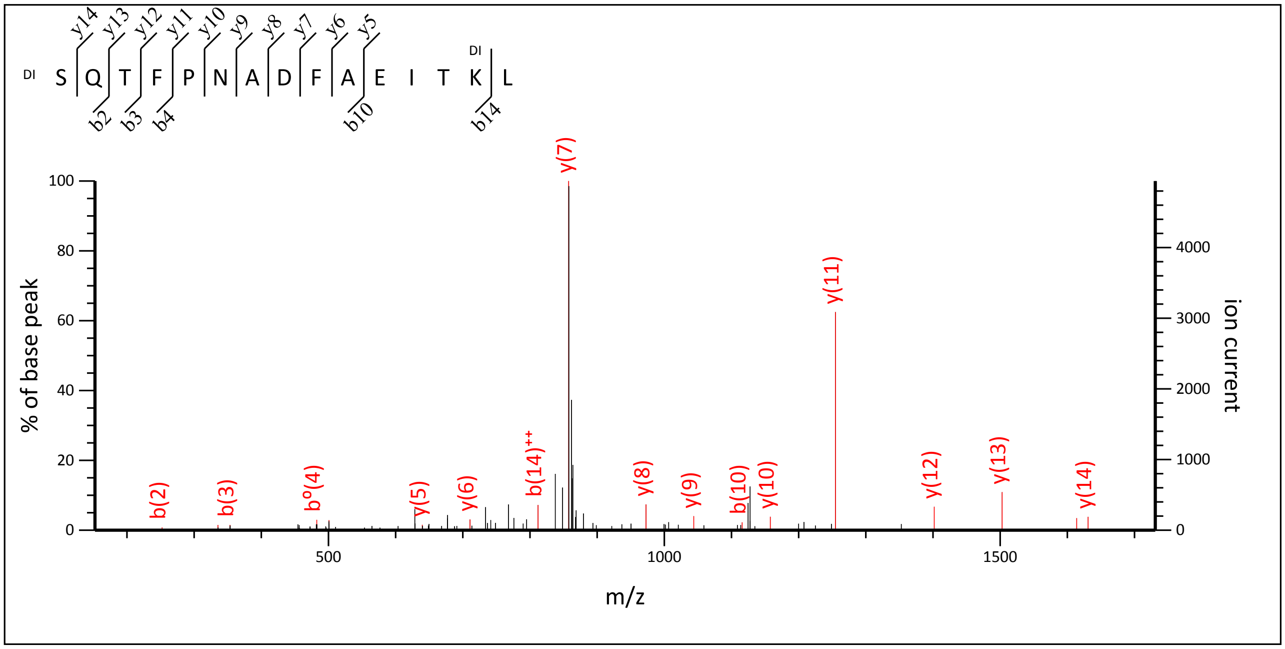

152.37 to 1730.94

Label all possible matches ○ Label matches used for scoring ◉

```
Monoisotopic mass of neutral peptide Mr(calc): 1752.9872
Variable modifications:
N-term : Dimethyl:2H(6)13C(2) (N-term)
K14    : Dimethyl:2H(6)13C(2) (K)
Ions Score: 59  Expect: 9.9e-005
Matches : 18/160 fragment ions using 44 most intense peaks   (help)
```

#	b	b^{++}	b*	b^{*++}	b^{0}	b^{0++}	Seq.	y	y^{++}	y*	y^{*++}	y^{0}	y^{0++}	#
1	124.1150	62.5611			106.1044	53.5558	S							15
2	**252.1736**	126.5904	235.1470	118.0771	234.1630	117.5851	Q	***1630.8868***	815.9470	1613.8603	807.4338	1612.8763	806.9418	14
3	**353.2212**	177.1143	336.1947	168.6010	335.2107	168.1090	T	***1502.8282***	751.9178	1485.8017	743.4045	1484.8177	742.9125	13
4	**500.2896**	250.6485	483.2631	242.1352	482.2791	241.6432	F	***1401.7806***	701.3939	1384.7540	692.8806	1383.7700	692.3886	12
5	597.3424	299.1748	580.3159	290.6616	579.3318	290.1696	P	***1254.7122***	627.8597	1237.6856	619.3464	1236.7016	618.8544	11
6	711.3853	356.1963	694.3588	347.6830	693.3748	347.1910	N	***1157.6594***	579.3333	1140.6328	570.8201	1139.6488	570.3280	10
7	782.4224	391.7149	765.3959	383.2016	764.4119	382.7096	A	***1043.6165***	522.3119	1026.5899	513.7986	1025.6059	513.3066	9
8	897.4494	449.2283	880.4228	440.7151	879.4388	440.2231	D	***972.5793***	486.7933	955.5528	478.2800	954.5688	477.7880	8
9	1044.5178	522.7625	1027.4913	514.2493	1026.5072	513.7573	F	***857.5524***	429.2798	840.5259	420.7666	839.5418	420.2746	7
10	1115.5549	558.2811	1098.5284	549.7678	1097.5444	549.2758	A	***710.4840***	355.7456	693.4574	347.2324	692.4734	346.7403	6
11	1244.5975	622.8024	1227.5710	614.2891	1226.5869	613.7971	E	***639.4469***	320.2271	622.4203	311.7138	621.4363	311.2218	5
12	1357.6816	679.3444	1340.6550	670.8312	1339.6710	670.3391	I	510.4043	255.7058	493.3777	247.1925	492.3937	246.7005	4
13	1458.7293	729.8683	1441.7027	721.3550	1440.7187	720.8630	T	397.3202	199.1637	380.2937	190.6505	379.3097	190.1585	3

14	1622.8999	811.9536	1605.8733	803.4403	1604.8893	802.9483	K	296.2725	148.6399	279.2460	140.1266			2
15							L	132.1019	66.5546					1

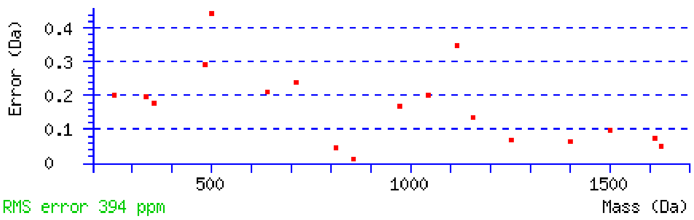

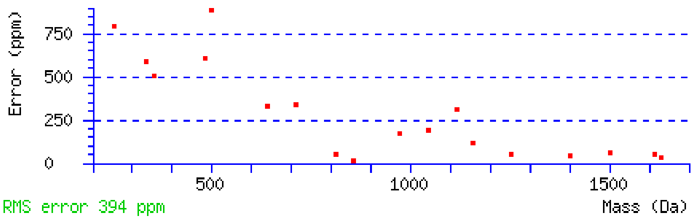

NCBI **BLAST** search of SQTFPNADFAEITKL
(Parameters: blastp, nr protein database, expect=20000, no filter, PAM30)
Other BLAST web gateways

All matches to this query

Score	Mr(calc)	Delta	Sequence
59.0	1752.9872	-0.0047	SQTFPNADFAEITKL
15.6	1752.9895	-0.0070	GILSPGVALGTAGGAMSSK
12.3	1753.0552	-0.0726	HLPPTLAAPGQPLRVL
10.9	1752.9762	0.0063	SQTMAPLKTVGTLGMM
10.8	1752.9633	0.0192	LFTPGSRIESILMSL
10.5	1752.9183	0.0643	SIMQSQSVMLPLVPGD
10.1	1752.9035	0.0790	TNTTNGTNGNLVPVTTN
10.1	1752.9048	0.0777	GSAPRHGSARSGPAPEPS
9.9	1753.0387	-0.0561	KKEDIFASSPMSGTK
9.9	1752.7168	0.2658	NCCEDTQASMQQVHQ

Mascot: http://www.matrixscience.com/

SQTFPNADFAEITKL

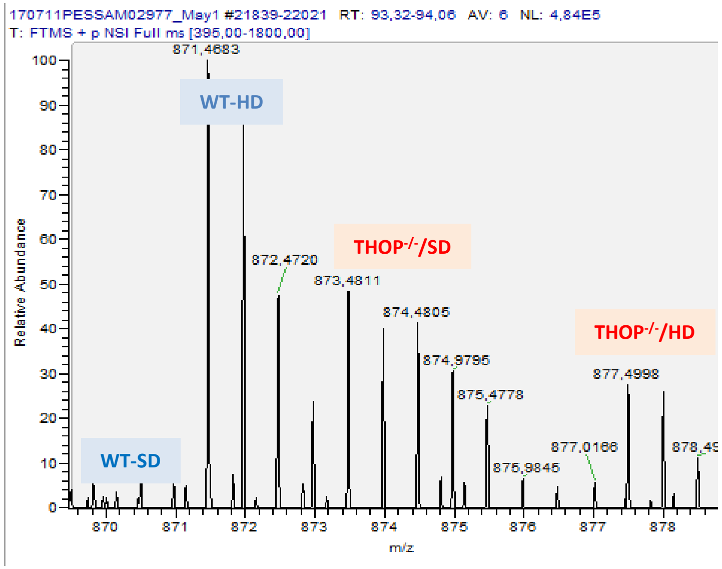

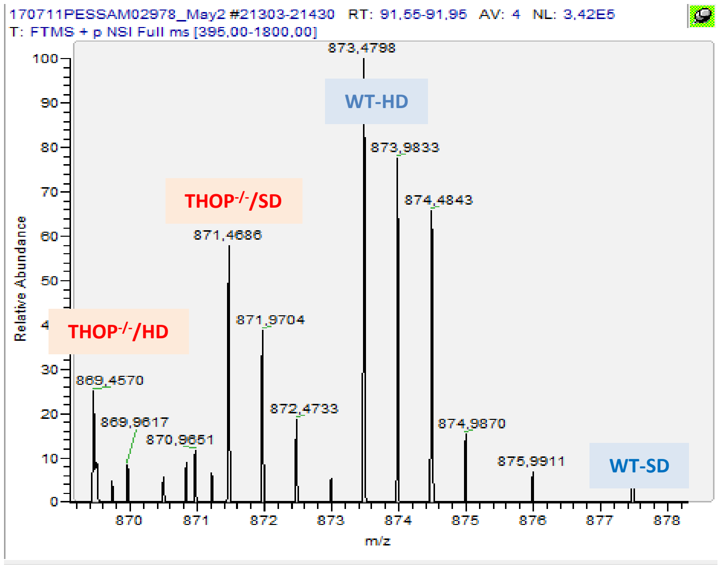

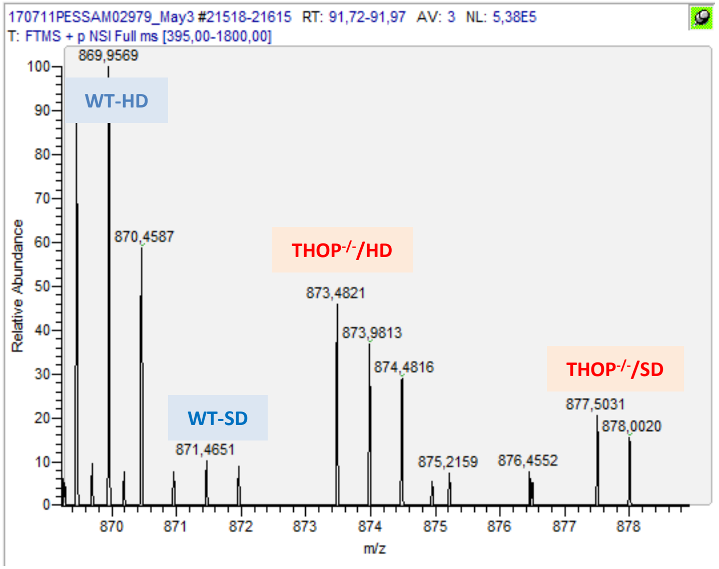

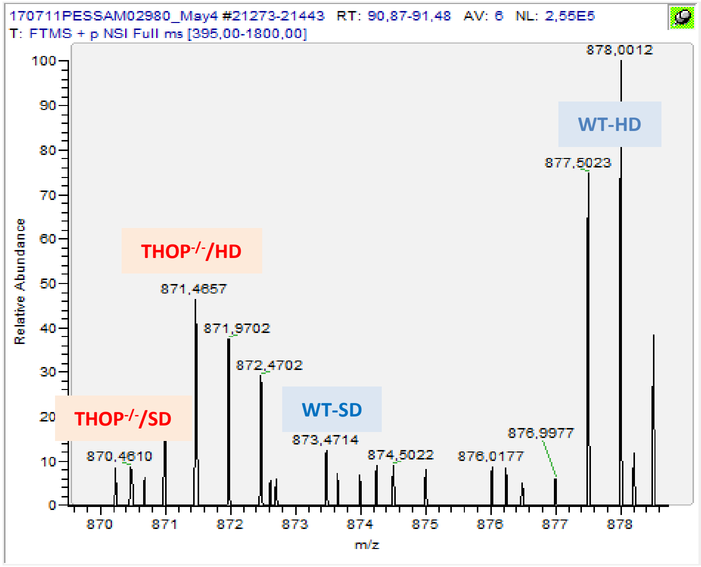

SQTFPNADFAEITKL

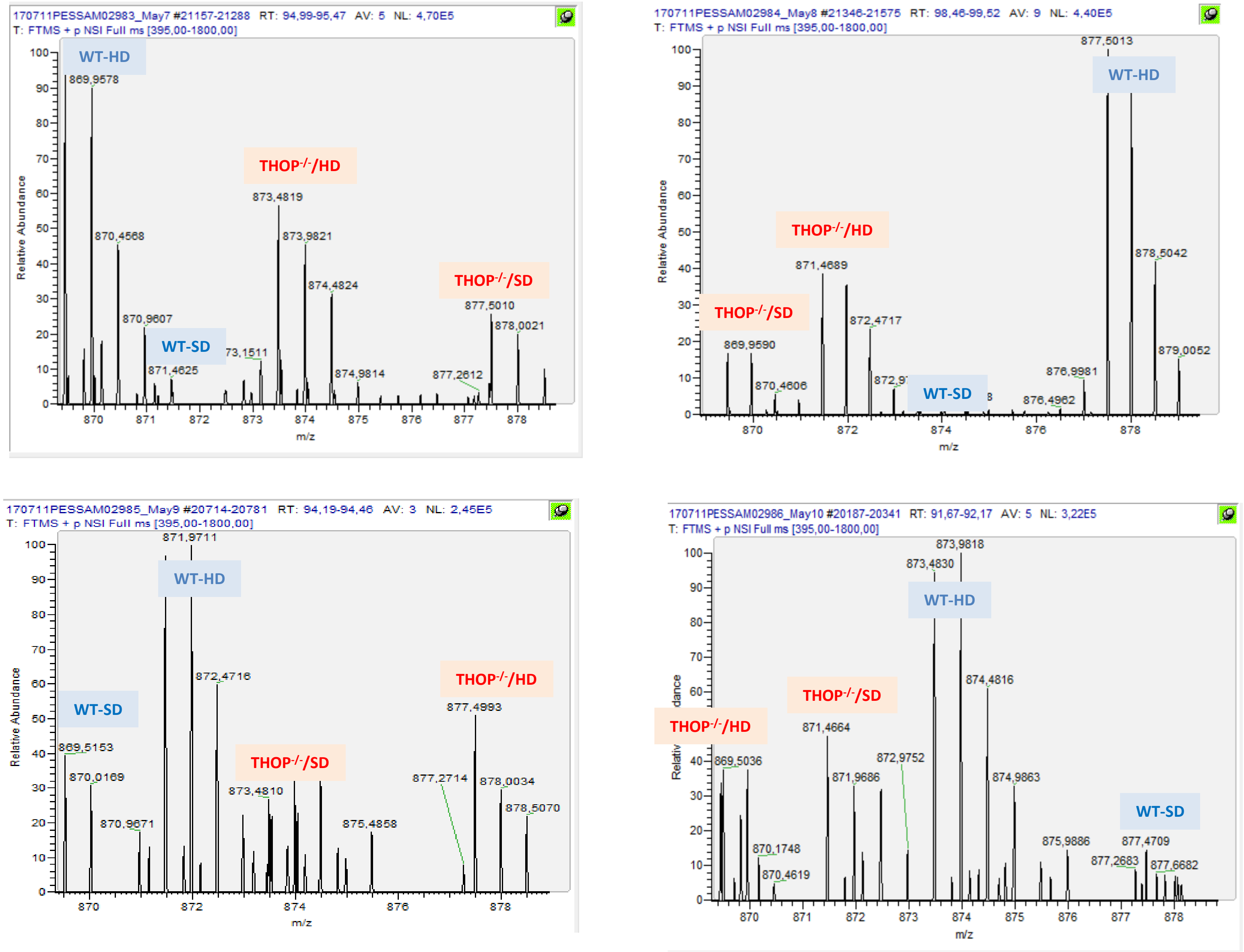

Mascot Search Results

Peptide View

MS/MS Fragmentation of **ADSDLLALMTGKMNPQSA**
Found in **NLTP_MOUSE** in **SwissProt**, Non-specific lipid-transfer protein OS=Mus musculus GN=Scp2 PE=1 SV=3

Match to Query 15454: 1917.951368 from(959.982960,2+) intensity(389329.7200) scans(21991) rawscans(sn21991) rtinseconds(5940.3122) index(14787)
Title: 14788: Scan 21991 (rt=5940.31) [D:\Users\Mayara - HMS Fusion data\170711PESSAM02983_May7.raw]
Data file 170711PESSAM02983_May7.temp.mgf

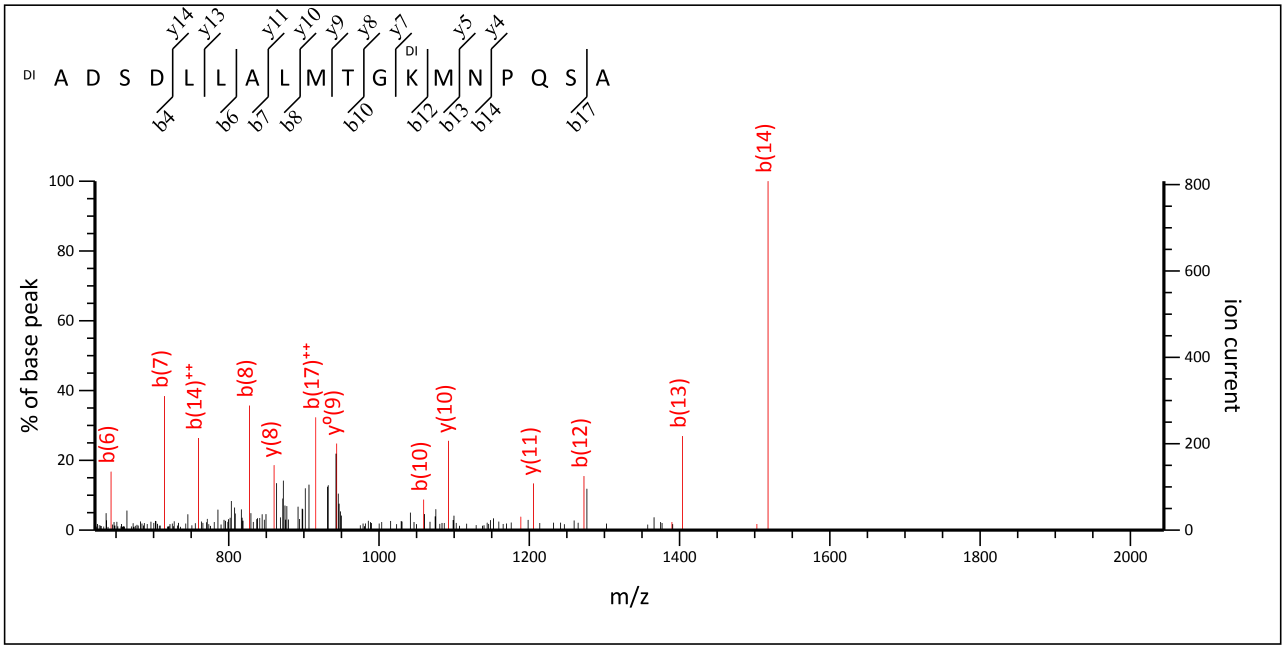

621.98 to 2044.58

Label all possible matches ○ Label matches used for scoring ◉

Monoisotopic mass of neutral peptide Mr(calc): 1917.9540
Variable modifications:
N-term : Dimethyl (N-term)
K12 : Dimethyl (K)
Ions Score: 78 **Expect:** 1.5e-005
Matches : 24/174 fragment ions using 25 most intense peaks (help)

#	b	b^{++}	b*	b*$^{++}$	b^{0}	b^{0++}	Seq.	y	y^{++}	y*	y*$^{++}$	y^{0}	y^{0++}	#
1	100.0757	50.5415					A							18
2	215.1026	108.0550			197.0921	99.0497	D	1819.8928	910.4501	1802.8663	901.9368	1801.8823	901.4448	17
3	302.1347	151.5710			284.1241	142.5657	S	1704.8659	852.9366	1687.8394	844.4233	1686.8553	843.9313	16
4	***417.1616***	209.0844			399.1510	200.0792	D	1617.8339	809.4206	1600.8073	800.9073	1599.8233	800.4153	15
5	***530.2457***	265.6265			512.2351	256.6212	L	**1502.8069**	751.9071	1485.7804	743.3938	1484.7964	742.9018	14
6	***643.3297***	322.1685			625.3192	313.1632	L	**1389.7229**	695.3651	1372.6963	686.8518	1371.7123	686.3598	13
7	***714.3668***	357.6871			696.3563	348.6818	A	1276.6388	638.8230	1259.6123	630.3098	1258.6282	629.8178	12
8	***827.4509***	414.2291			809.4403	405.2238	L	**1205.6017**	603.3045	1188.5751	594.7912	1187.5911	594.2992	11
9	958.4914	479.7493			940.4808	470.7441	M	**1092.5176**	546.7625	1075.4911	538.2492	1074.5071	537.7572	10
10	***1059.5391***	**530.2732**			1041.5285	521.2679	T	961.4771	481.2422	944.4506	472.7289	943.4666	472.2369	9
11	1116.5605	558.7839			1098.5500	549.7786	G	**860.4295**	430.7184	843.4029	422.2051	842.4189	421.7131	8
12	***1272.6868***	636.8470	1255.6603	628.3338	1254.6762	627.8418	K	803.4080	402.2076	786.3815	393.6944	785.3974	393.2024	7
13	***1403.7273***	702.3673	1386.7007	693.8540	1385.7167	693.3620	M	647.2817	324.1445	630.2552	315.6312	629.2712	315.1392	6

**14**	***1517.7702***	**759.3887**	1500.7437	750.8755	1499.7596	750.3835	**N**	**516.2413**	258.6243	499.2147	250.1110	498.2307	249.6190	**5**
**15**	1614.8230	807.9151	1597.7964	799.4019	1596.8124	798.9098	**P**	**402.1983**	201.6028	385.1718	193.0895	384.1878	192.5975	**4**
**16**	1742.8816	871.9444	1725.8550	863.4311	1724.8710	862.9391	**Q**	305.1456	153.0764	288.1190	144.5631	287.1350	144.0711	**3**
**17**	1829.9136	**915.4604**	1812.8870	906.9472	1811.9030	906.4551	**S**	177.0870	89.0471			159.0764	80.0418	**2**
**18**							**A**	90.0550	45.5311					**1**

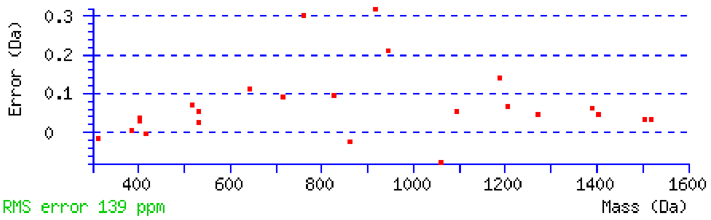

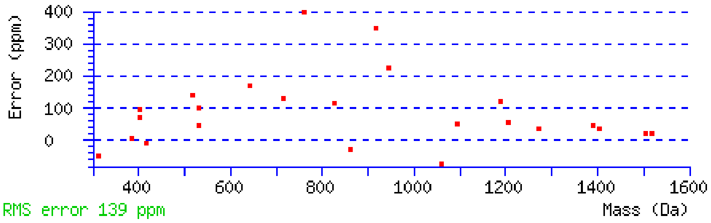

NCBI **BLAST** search of ADSDLLALMTGKMNPQSA
(Parameters: blastp, nr protein database, expect=20000, no filter, PAM30)
Other BLAST web gateways

All matches to this query

Score	Mr(calc)	Delta	Sequence
77.8	1917.9540	-0.0026	ADSDLLALMTGKMNPQSA
25.6	1918.0485	-0.0971	MQLSIALDINFSIIDK
23.2	1918.0303	-0.0789	PYSYISLITMAIQQAPS
22.2	1918.0138	-0.0624	AGGAAAGLSPPAPAPFPLEPG
21.6	1917.9694	-0.0180	PAGGSGGGLSDSGRNSLTSLP
21.6	1918.1900	-0.2386	KDLLLAAELGKMLLER
20.0	1917.9578	-0.0064	MEAQLASQSTQRTGKGQ
19.9	1918.0921	-0.1407	EAENEKMKAIGARNLL
19.5	1918.1342	-0.1828	WRIYRPTTGALLLLTA
19.3	1918.1007	-0.1494	VPLVIGCAVSCMALLTLLA

Mascot: http://www.matrixscience.com/

ADSDLLALMTGKMNPQSA

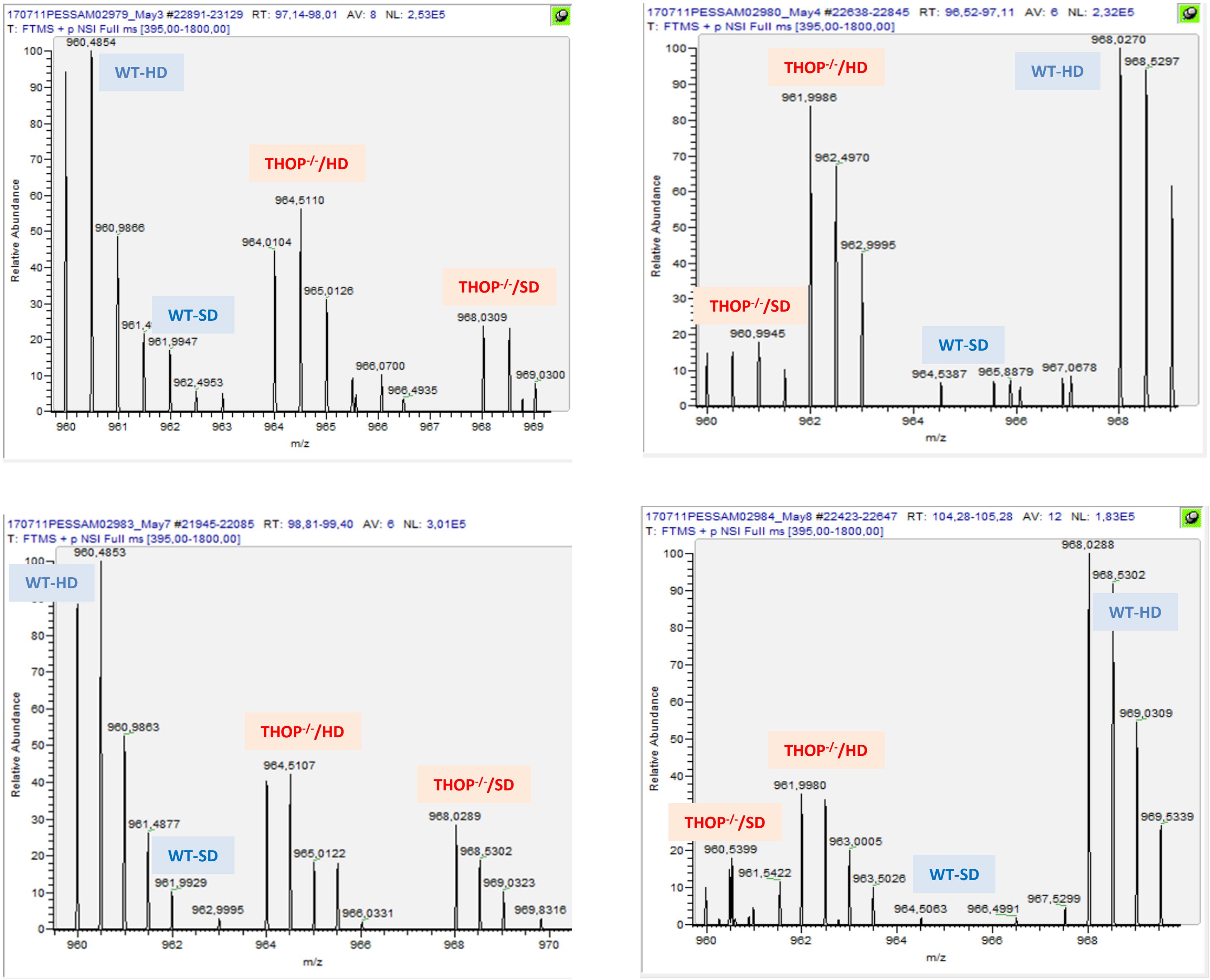

ADSDLLALMTGKMNPQSA

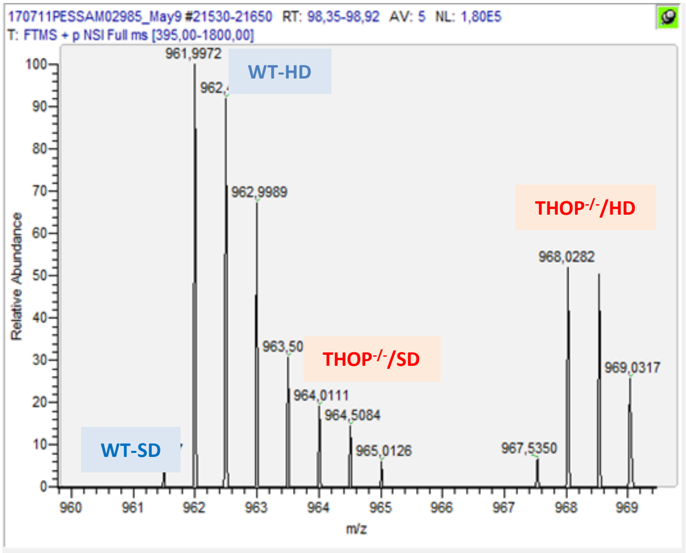

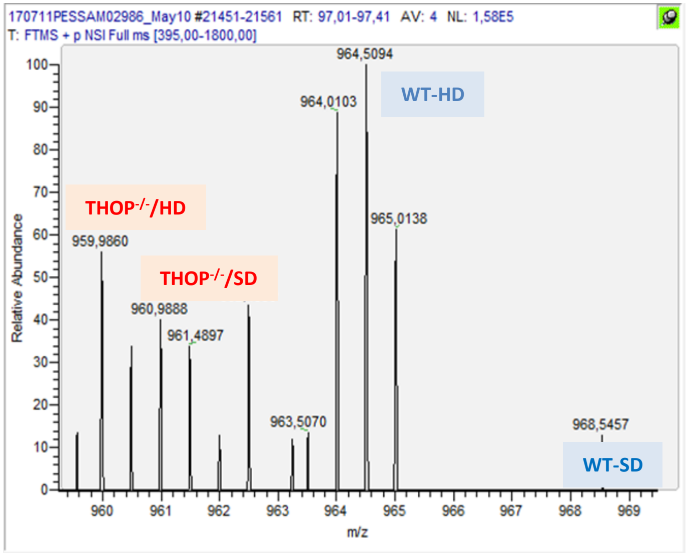

Mascot Search Results

Peptide View

MS/MS Fragmentation of **GAEALERMFASFPTTK**
Found in **HBA_MOUSE** in **SwissProt**, Hemoglobin subunit alpha OS=Mus musculus GN=Hba PE=1 SV=2

Match to Query 14265: 1814.951108 from(908.482830,2+) intensity(4558077.2000) scans(20700-20805) rawscans(sn20700:sn20805) rtinseconds(5278.4324-5307.2462) index(13611)
Title: 13612: Sum of 4 scans in range 20700 (rt=5278.43) to 20805 (rt=5307.25) [D:\Users\Mayara - HMS Fusion data\170711PESSAM02977_May1.raw]
Data file 170711PESSAM02977_May1.temp.mgf

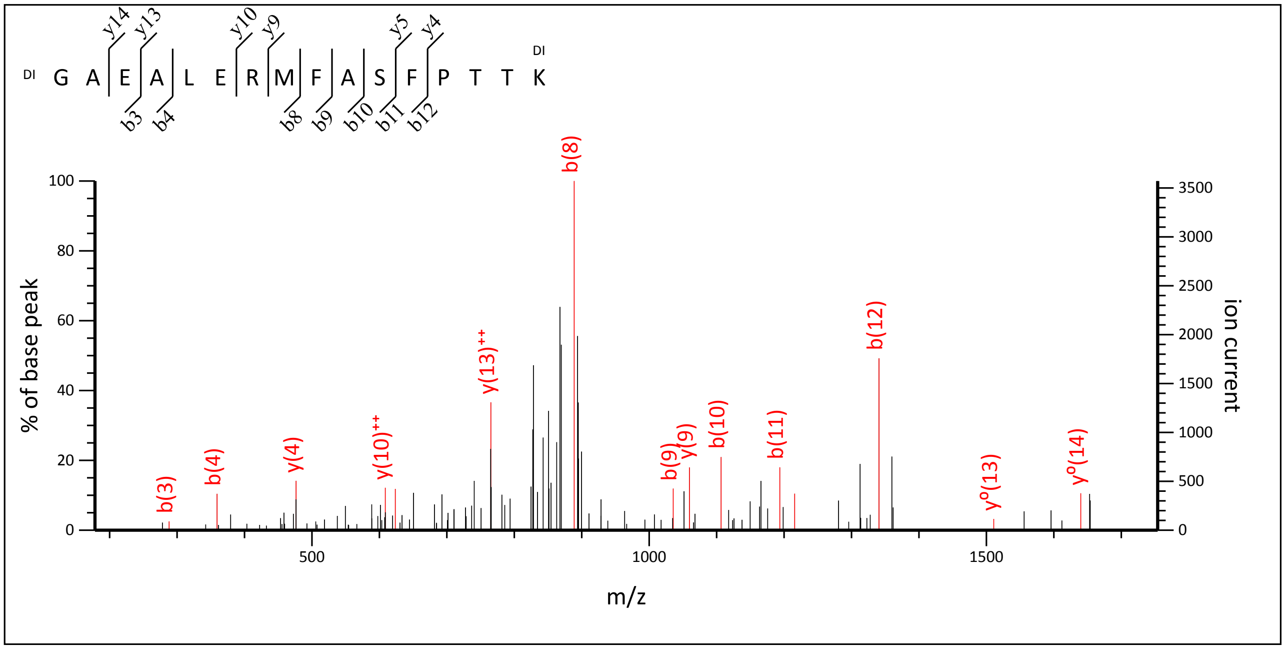

178.27 to 1753.95

Label all possible matches ○ Label matches used for scoring ◉

Monoisotopic mass of neutral peptide Mr(calc): 1814.9600
Variable modifications:
N-term : Dimethyl:2H(2) (N-term)
K16 : Dimethyl:2H(2) (K)
Ions Score: 52 **Expect:** 0.00084
Matches : 16/162 fragment ions using 26 most intense peaks (help)

#	b	b^{++}	b*	b^{*++}	b^0	b^{0++}	Seq.	y	y^{++}	y*	y^{*++}	y^0	y^{0++}	#
1	88.0757	44.5415					G							16
2	159.1128	80.0600					A	1728.8989	864.9531	1711.8724	856.4398	1710.8884	855.9478	15
3	***288.1554***	144.5813			270.1448	135.5761	E	1657.8618	829.4345	1640.8353	820.9213	1639.8512	820.4293	14
4	***359.1925***	180.0999			341.1819	171.0946	A	1528.8192	**764.9132**	1511.7927	756.4000	1510.8086	755.9080	13
5	472.2766	236.6419			454.2660	227.6366	L	1457.7821	729.3947	1440.7556	720.8814	1439.7715	720.3894	12
6	601.3192	301.1632			583.3086	292.1579	E	1344.6980	672.8527	1327.6715	664.3394	1326.6875	663.8474	11
7	757.4203	379.2138	740.3937	370.7005	739.4097	370.2085	R	**1215.6554**	**608.3314**	1198.6289	599.8181	1197.6449	599.3261	10
8	***888.4608***	444.7340	871.4342	436.2207	870.4502	435.7287	M	**1059.5543**	530.2808	1042.5278	521.7675	1041.5438	521.2755	9
9	***1035.5292***	518.2682	1018.5026	509.7550	1017.5186	509.2629	F	928.5138	464.7606	911.4873	456.2473	910.5033	455.7553	8
10	***1106.5663***	553.7868	1089.5397	545.2735	1088.5557	544.7815	A	781.4454	391.2264	764.4189	382.7131	763.4349	382.2211	7
11	***1193.5983***	597.3028	1176.5718	588.7895	1175.5878	588.2975	S	710.4083	355.7078	693.3818	347.1945	692.3978	346.7025	6
12	***1340.6667***	670.8370	1323.6402	662.3237	1322.6562	661.8317	F	**623.3763**	312.1918	606.3497	303.6785	605.3657	303.1865	5
13	1437.7195	719.3634	1420.6929	710.8501	1419.7089	710.3581	P	**476.3079**	238.6576	459.2813	230.1443	458.2973	229.6523	4

14	1538.7672	769.8872	1521.7406	761.3740	1520.7566	760.8819	T	379.2551	190.1312	362.2286	181.6179	361.2445	181.1259	3
15	*1639.8149*	820.4111	1622.7883	811.8978	1621.8043	811.4058	T	278.2074	139.6074	261.1809	131.0941	260.1969	130.6021	2
16							K	177.1598	89.0835	160.1332	80.5702			1

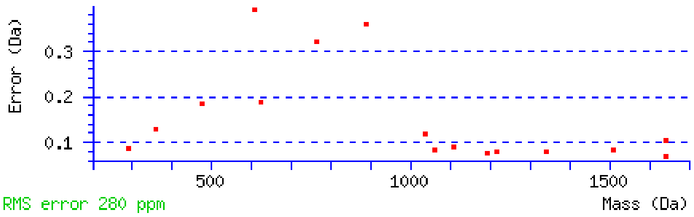

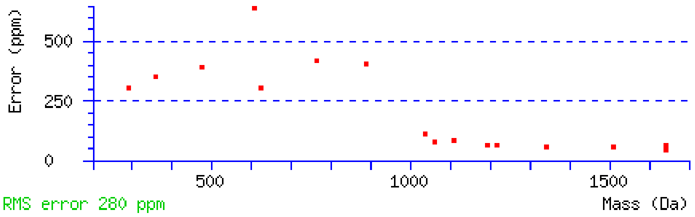

NCBI **BLAST** search of GAEALERMFASFPTTK
(Parameters: blastp, nr protein database, expect=20000, no filter, PAM30)
Other BLAST web gateways

All matches to this query

Score	Mr(calc)	Delta	Sequence
52.5	1814.9600	-0.0089	GAEALERMFASFPTTK
18.8	1814.9854	-0.0343	SVPRFSAAASCSNITRL
17.2	1815.1671	-0.2160	VKASTCKPFLFLLTK
14.0	1814.8331	0.1180	GMSLGMSATTHYHTYL
13.1	1814.7787	0.1724	NNSAGMAASPEMMGLGDV
13.1	1815.0001	-0.0490	LIGIYATALSASMSSLIG
12.7	1814.8506	0.1005	NVATSEDSDLSMRTLS
12.4	1815.0029	-0.0518	KSADFFPGLSAFLSQE
12.0	1815.0103	-0.0592	EEFMLLPLKQVLEL
11.4	1814.7822	0.1690	AVGYSWPGAFSMADSSP

Mascot: http://www.matrixscience.com/

GAEALERMFASFPTTK

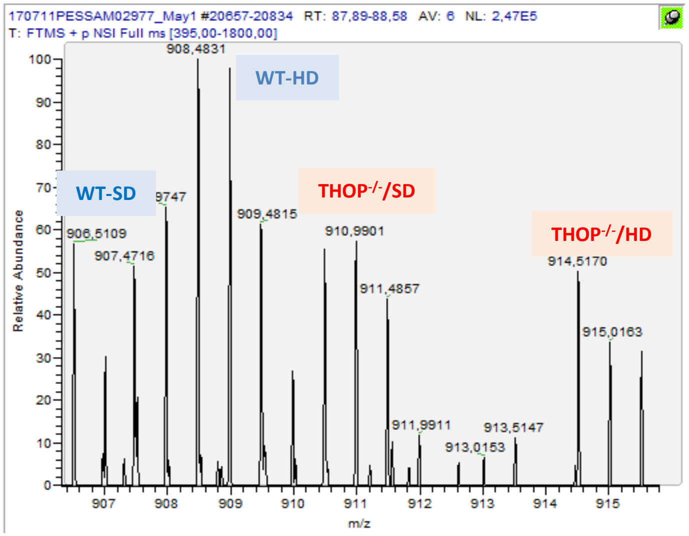

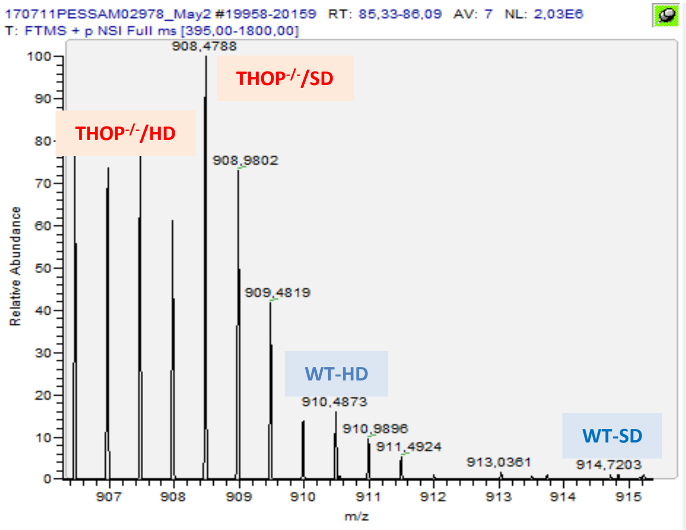

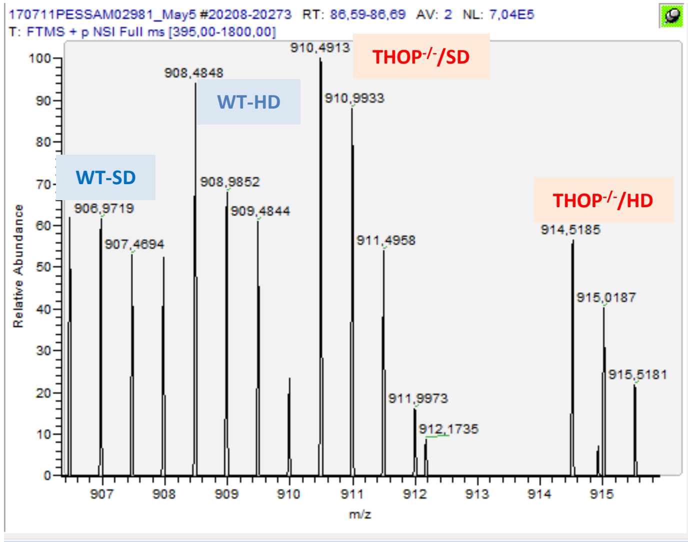

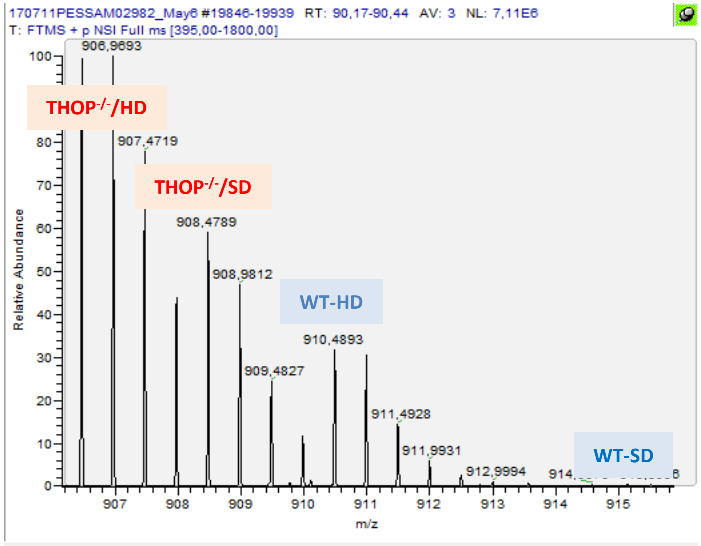

GAEALERMFASFPTTK

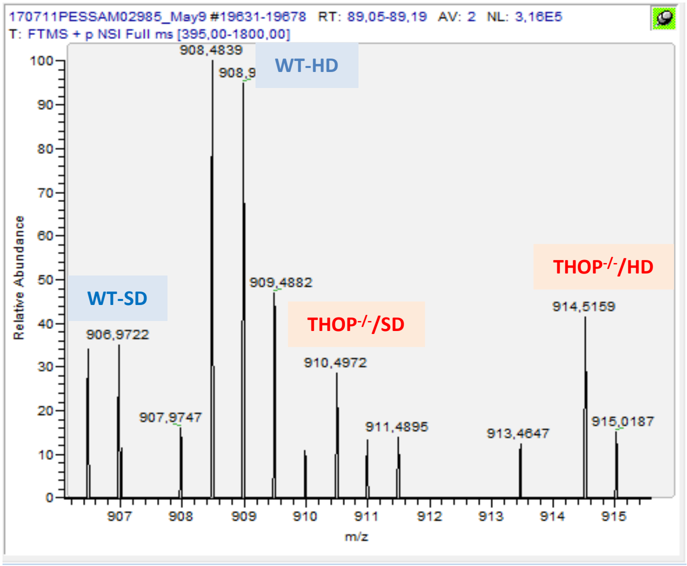

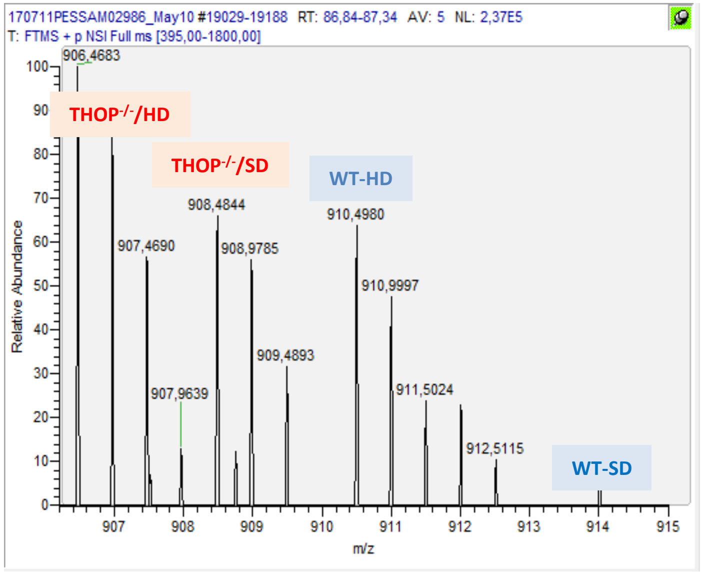

Mascot Search Results

Peptide View

MS/MS Fragmentation of **FDVSHGSAQVK**
Found in **HBA_MOUSE** in **SwissProt**, Hemoglobin subunit alpha OS=Mus musculus GN=Hba PE=1 SV=2

Match to Query 5649: 1233.664668 from(617.839610,2+) intensity(5933215.5000) scans(2251) rawscans(sn2251) rtinseconds(1457.7588) index(1428)
Title: 1429: Scan 2251 (rt=1457.76) [D:\Users\Mayara - HMS Fusion data\170711PESSAM02981_May5.raw]
Data file 170711PESSAM02981_May5.temp.mgf

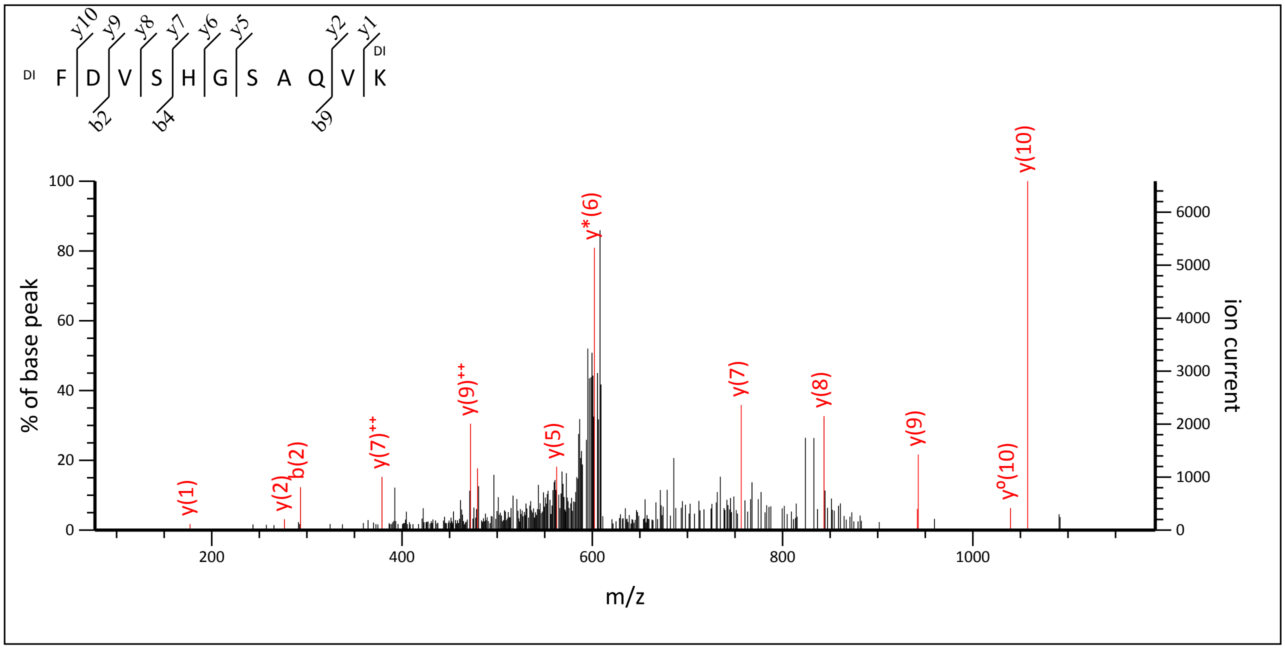

77.26 to 1191.75

Label all possible matches Label matches used for scoring

Monoisotopic mass of neutral peptide Mr(calc): 1233.6718
Variable modifications:
N-term : Dimethyl:2H(2) (N-term)
K11 : Dimethyl:2H(2) (K)
Ions Score: 51 **Expect:** 0.011
Matches : 16/94 fragment ions using 20 most intense peaks (help)

#	b	b^{++}	b*	b^{*++}	b^{0}	b^{0++}	Seq.	y	y^{++}	y*	y^{*++}	y^{0}	y^{0++}	#
1	178.1226	89.5650					F							11
2	**293.1496**	147.0784			275.1390	138.0731	D	***1057.5636***	529.2855	1040.5371	520.7722	1039.5531	520.2802	10
3	392.2180	196.6126			374.2074	187.6074	V	***942.5367***	**471.7720**	925.5102	463.2587	924.5261	462.7667	9
4	**479.2500**	240.1287			461.2395	231.1234	S	***843.4683***	422.2378	826.4417	413.7245	825.4577	413.2325	8
5	616.3089	308.6581			598.2984	299.6528	H	***756.4363***	**378.7218**	739.4097	370.2085	738.4257	369.7165	7
6	673.3304	337.1688			655.3198	328.1636	G	619.3774	310.1923	602.3508	301.6790	601.3668	301.1870	6
7	760.3624	380.6849			742.3519	371.6796	S	***562.3559***	281.6816	545.3293	273.1683	544.3453	272.6763	5
8	831.3995	416.2034			813.3890	407.1981	A	475.3239	238.1656	458.2973	229.6523			4
9	959.4581	480.2327	942.4316	471.7194	941.4476	471.2274	Q	404.2867	202.6470	387.2602	194.1337			3
10	1058.5265	529.7669	1041.5000	521.2536	1040.5160	520.7616	V	***276.2282***	138.6177	259.2016	130.1044			2
11							K	***177.1598***	89.0835	160.1332	80.5702			1

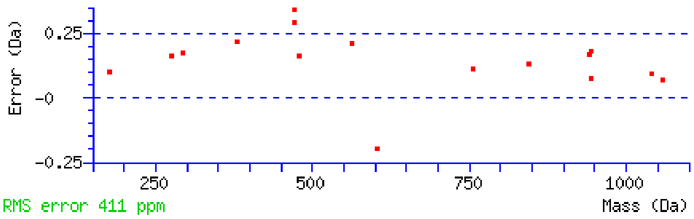

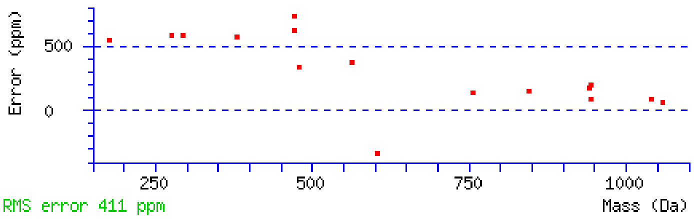

NCBI **BLAST** search of FDVSHGSAQVK
(Parameters: blastp, nr protein database, expect=20000, no filter, PAM30)
Other BLAST web gateways

All matches to this query

Score	Mr(calc)	Delta	Sequence
50.8	1233.6718	-0.0071	FDVSHGSAQVK
28.7	1233.6176	0.0471	MDVSFHLPSR
20.9	1233.6241	0.0405	FDVSAPGSGGLTP
17.2	1233.6639	0.0008	TPVKMPPSNTS
16.7	1233.7043	-0.0396	IGVIFMASPVL
16.6	1233.6791	-0.0145	MITSHFPPKT
16.6	1233.5105	0.1542	MTLSTEMSDAS
15.5	1233.6577	0.0070	IMVSAIGNDGPL
14.7	1233.5980	0.0667	VDVSTEAMYAA
14.4	1233.7243	-0.0596	CDVSKTLLHL

Mascot: http://www.matrixscience.com/

FDVSHGSAQVK

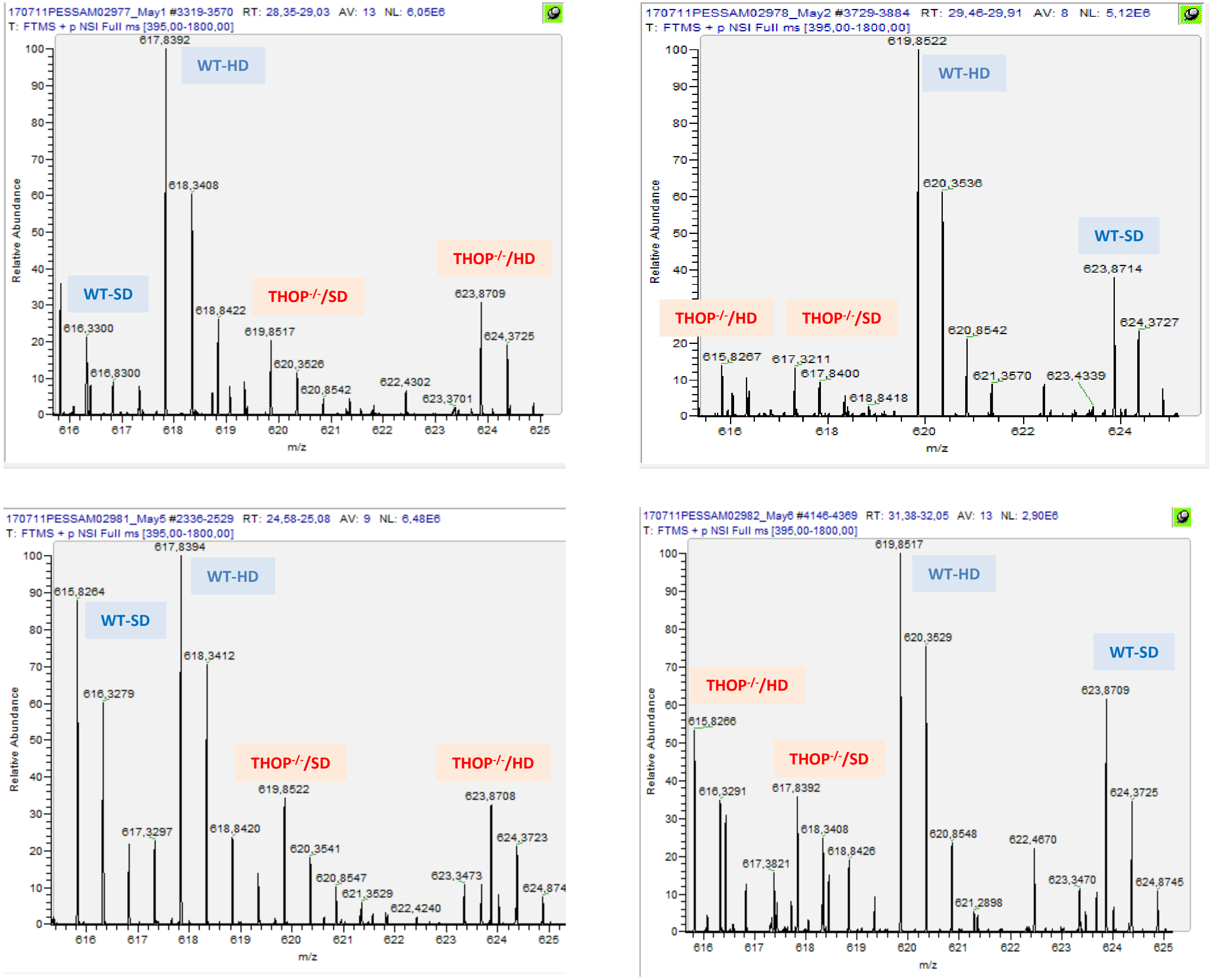

FDVSHGSAQVK

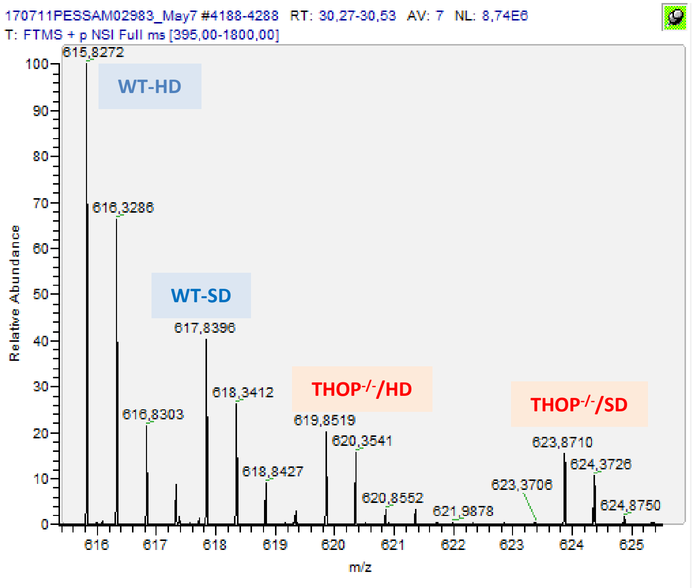

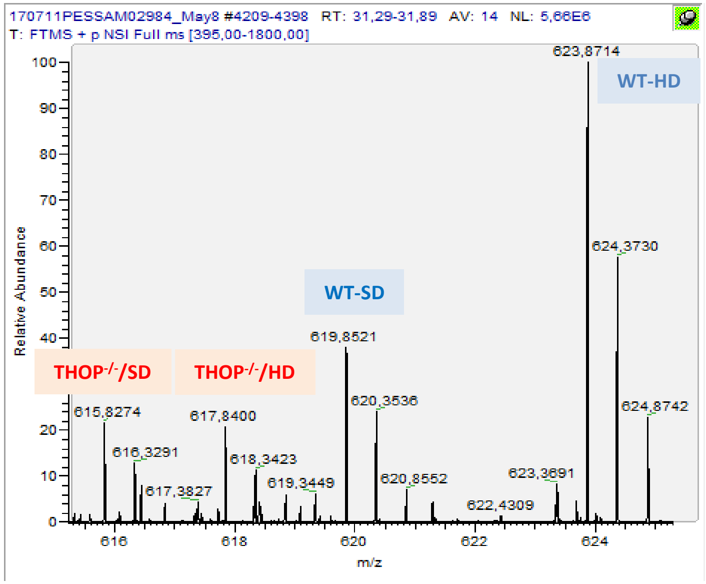

Mascot Search Results

Peptide View

MS/MS Fragmentation of **IGGHGAEYGAEALER**
Found in **HBA_MOUSE** in **SwissProt**, Hemoglobin subunit alpha OS=Mus musculus GN=Hba PE=1 SV=2

Match to Query 10548: 1556.759292 from(519.927040,3+) intensity(6059307.0000) scans(7099) rawscans(sn7099) rtinseconds(2381.2278) index(4520)
Title: 4521: Scan 7099 (rt=2381.23) [D:\Users\Mayara - HMS Fusion data\170711PESSAM02983_May7.raw]
Data file 170711PESSAM02983_May7.temp.mgf

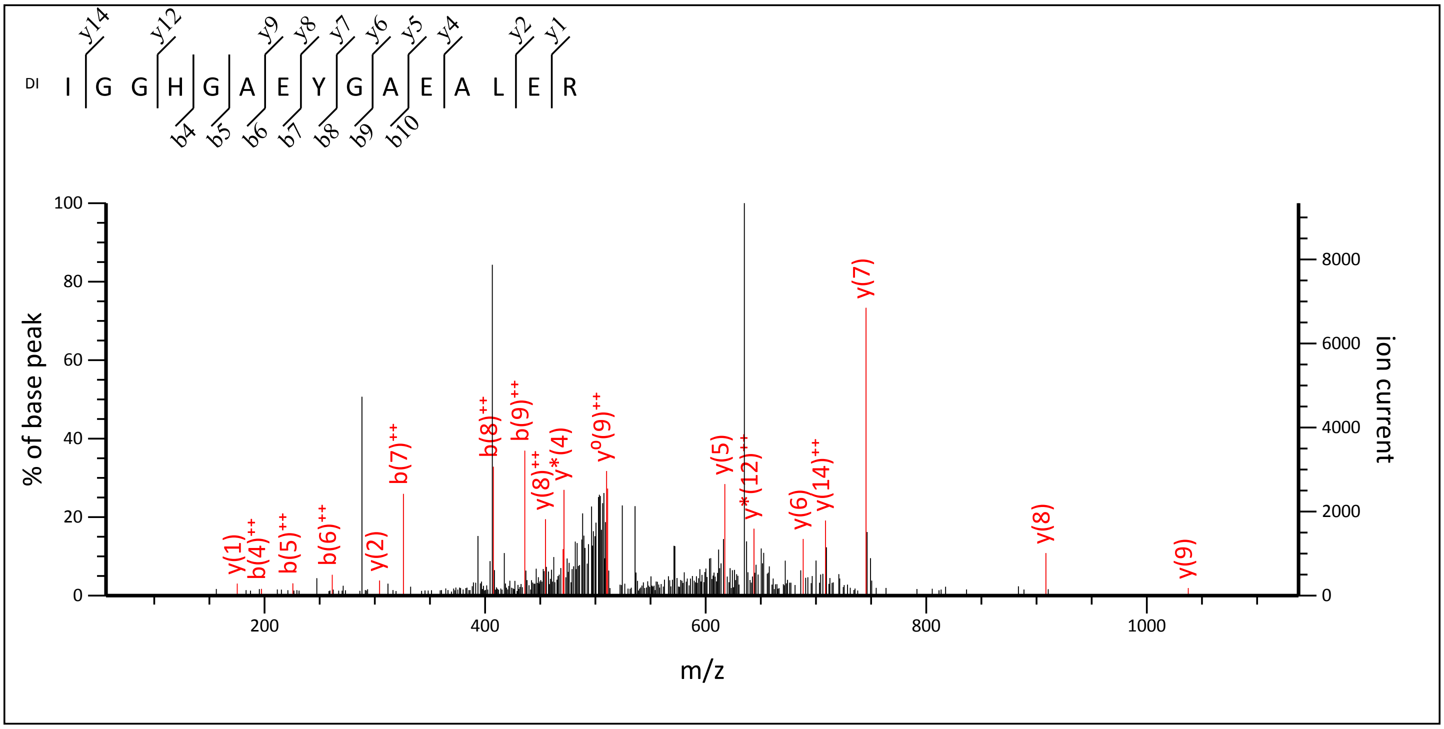

56.27 to 1137.57

Label all possible matches ○ Label matches used for scoring ●

Monoisotopic mass of neutral peptide Mr(calc): 1556.7583
Variable modifications:
N-term : Dimethyl (N-term)
Ions Score: 44 **Expect:** 0.0065
Matches : 20/126 fragment ions using 33 most intense peaks (help)

#	b	b^{++}	b^{0}	b^{0++}	Seq.	y	y^{++}	y*	y^{*++}	y^{0}	y^{0++}	#
1	142.1226	71.5650			I							15
2	199.1441	100.0757			G	1416.6502	**708.8288**	1399.6237	700.3155	1398.6397	699.8235	14
3	256.1656	128.5864			G	1359.6288	680.3180	1342.6022	671.8047	1341.6182	671.3127	13
4	393.2245	***197.1159***			H	1302.6073	651.8073	1285.5808	643.2940	1284.5967	642.8020	12
5	450.2459	***225.6266***			G	1165.5484	583.2778	1148.5218	574.7646	1147.5378	574.2726	11
6	521.2831	***261.1452***			A	1108.5269	554.7671	1091.5004	546.2538	1090.5164	545.7618	10
7	650.3257	***325.6665***	632.3151	316.6612	E	***1037.4898***	519.2485	1020.4633	510.7353	1019.4793	510.2433	9
8	813.3890	***407.1981***	795.3784	398.1928	Y	***908.4472***	**454.7272**	891.4207	446.2140	890.4367	445.7220	8

**9**	870.4104	***435.7089***	852.3999	426.7036	**G**	***745.3839***	373.1956	728.3573	364.6823	727.3733	364.1903	**7**
**10**	941.4476	***471.2274***	923.4370	462.2221	**A**	***688.3624***	344.6849	671.3359	336.1716	670.3519	335.6796	**6**
**11**	1070.4902	535.7487	1052.4796	526.7434	**E**	***617.3253***	309.1663	600.2988	300.6530	599.3148	300.1610	**5**
**12**	1141.5273	571.2673	1123.5167	562.2620	**A**	488.2827	244.6450	471.2562	236.1317	470.2722	235.6397	**4**
**13**	1254.6113	627.8093	1236.6008	618.8040	**L**	417.2456	209.1264	400.2191	200.6132	399.2350	200.1212	**3**
**14**	1383.6539	692.3306	1365.6434	683.3253	**E**	***304.1615***	152.5844	287.1350	144.0711	286.1510	143.5791	**2**
**15**					**R**	***175.1190***	88.0631	158.0924	79.5498			**1**

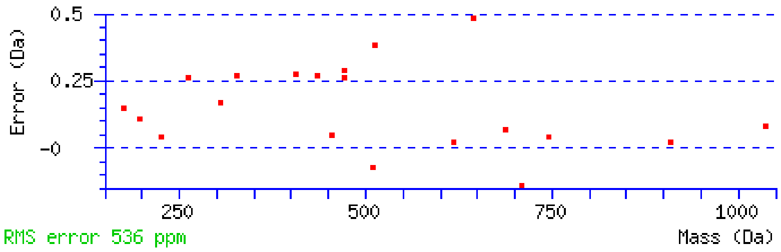

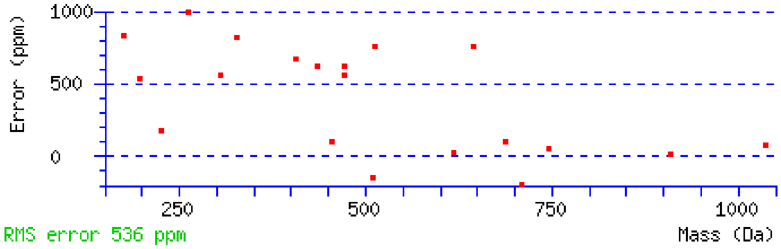

NCBI **BLAST** search of IGGHGAEYGAEALER
(Parameters: blastp, nr protein database, expect=20000, no filter, PAM30)
Other BLAST web gateways

All matches to this query

Score	Mr(calc)	Delta	Sequence
44.5	1556.7583	0.0010	IGGHGAEYGAEALER
20.4	1556.8708	-0.1115	AYIHEKGMIHRD
18.9	1556.8218	-0.0625	ELEAEKAGTVSMY
17.4	1556.7028	0.0565	EMGDEYAVERAVT
17.4	1556.8546	-0.0953	LPMMEYIPEKRA
17.4	1556.8546	-0.0953	LPMMEYIPEKRA
16.8	1556.9713	-0.2120	SAKGAERVITLKM
16.6	1556.9054	-0.1461	DLPYEYLKPSRI
15.3	1556.8675	-0.1082	KGNKGEHGPPGPPGP
14.6	1556.8596	-0.1003	IAGLNMLSAISIER

Mascot: http://www.matrixscience.com/

IGGHGAEYGAEALER

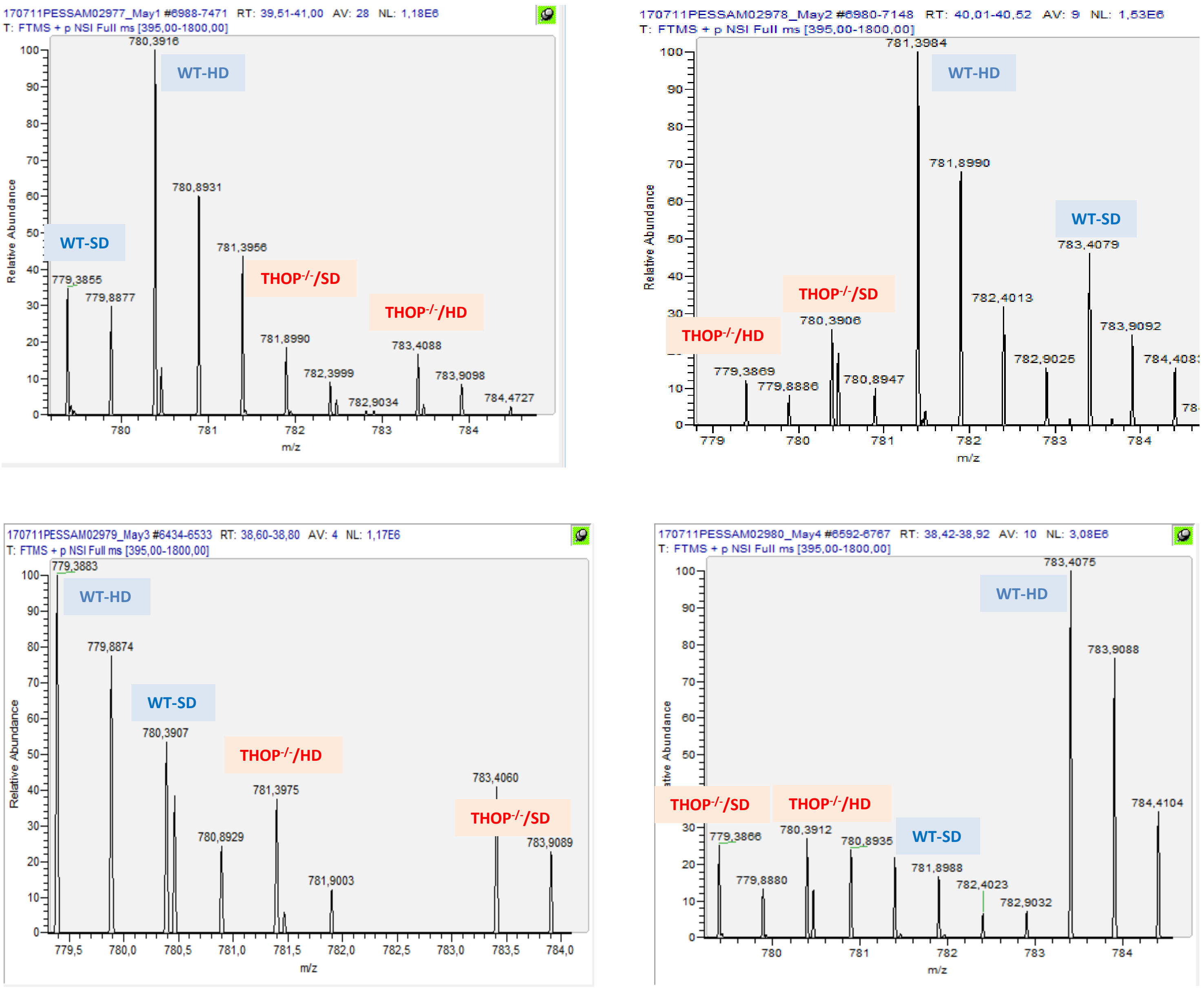

IGGHGAEYGAEALER

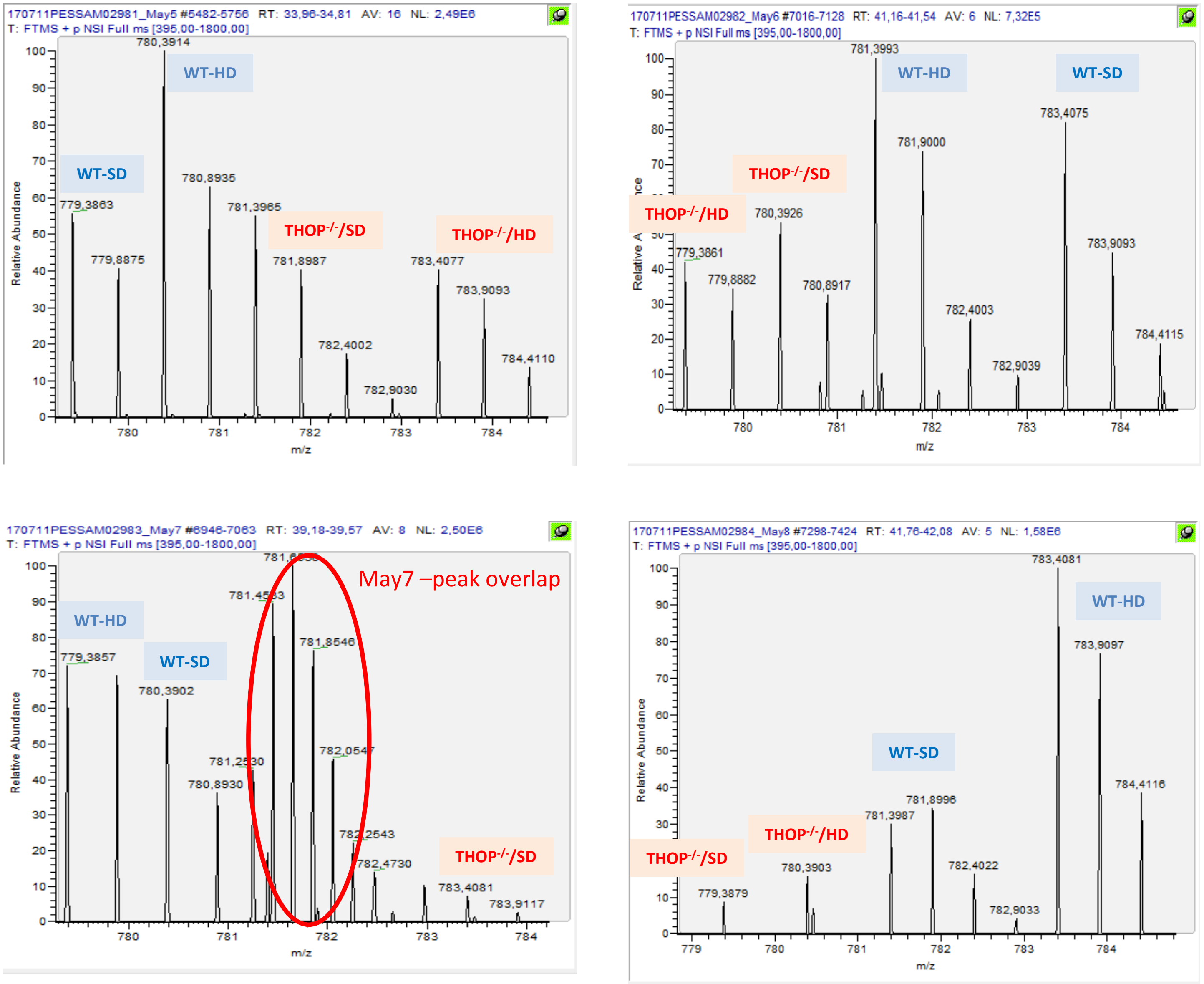

Mascot Search Results

Peptide View

MS/MS Fragmentation of **SVSTVLTSK**
Found in **HBA_MOUSE** in **SwissProt**, Hemoglobin subunit alpha OS=Mus musculus GN=Hba PE=1 SV=2

Match to Query 2839: 992.668188 from(497.341370,2+) intensity(14296911.0000) scans(5675) rawscans(sn5675) rtinseconds(2123.5117) index(3301)
Title: 3302: Scan 5675 (rt=2123.51) [D:\Users\Mayara - HMS Fusion data\170711PESSAM02977_May1.raw]
Data file 170711PESSAM02977_May1.temp.mgf

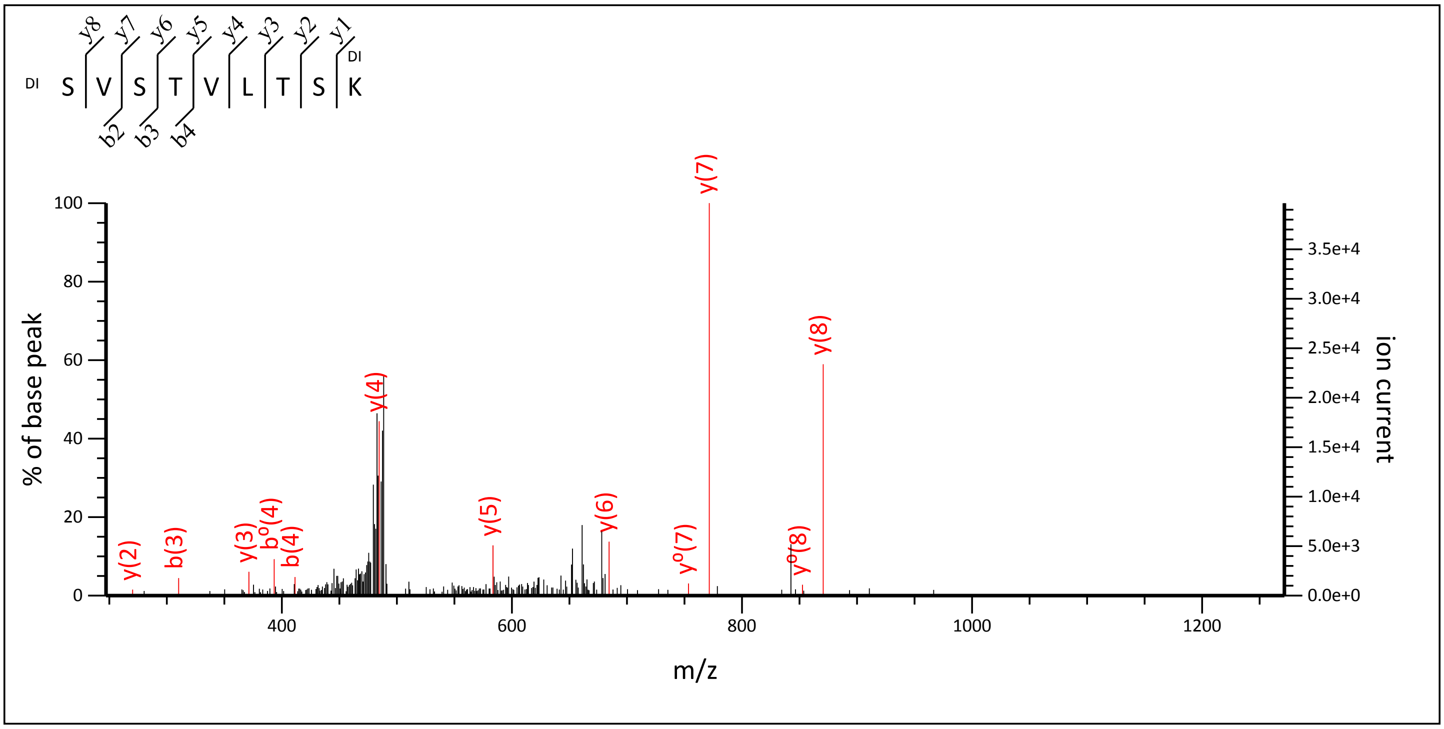

247.13 to 1271.51

Label all possible matches ○ Label matches used for scoring ◉

Monoisotopic mass of neutral peptide Mr(calc): 992.6692
Variable modifications:
N-term : Dimethyl:2H(6)13C(2) (N-term)
K9 : Dimethyl:2H(6)13C(2) (K)
Ions Score: 62 **Expect:** 0.023
Matches : 14/78 fragment ions using 25 most intense peaks (help)

#	b	b^{++}	b^{0}	b^{0++}	Seq.	y	y^{++}	y^{*}	y^{*++}	y^{0}	y^{0++}	#
1	124.1150	62.5611	106.1044	53.5558	S							9
2	**223.1834**	112.0953	205.1728	103.0900	V	***870.5688***	435.7880	853.5422	427.2748	852.5582	426.7827	8
3	**310.2154**	155.6113	292.2049	146.6061	S	***771.5004***	386.2538	754.4738	377.7405	753.4898	377.2485	7
4	**411.2631**	206.1352	393.2525	197.1299	T	***684.4683***	342.7378	667.4418	334.2245	666.4578	333.7325	6
5	510.3315	255.6694	492.3209	246.6641	V	***583.4207***	292.2140	566.3941	283.7007	565.4101	283.2087	5
6	623.4156	312.2114	605.4050	303.2061	L	***484.3522***	242.6798	467.3257	234.1665	466.3417	233.6745	4
7	724.4633	362.7353	706.4527	353.7300	T	***371.2682***	186.1377	354.2416	177.6245	353.2576	177.1324	3
8	811.4953	406.2513	793.4847	397.2460	S	***270.2205***	135.6139	253.1940	127.1006	252.2099	126.6086	2

**9**					**K**	***183.1885***	92.0979	166.1619	83.5846			**1**

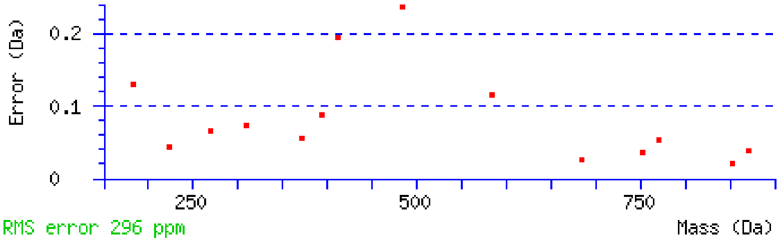

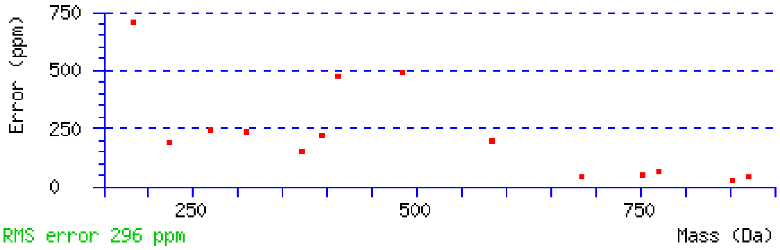

NCBI **BLAST** search of SVSTVLTSK
(Parameters: blastp, nr protein database, expect=20000, no filter, PAM30)
Other BLAST web gateways

All matches to this query

Score	Mr(calc)	Delta	Sequence
62.2	992.6692	-0.0010	SVSTVLTSK
44.8	992.6328	0.0354	GSSTVITSK
26.9	992.5030	0.1652	SVSSFAVGGM
26.2	992.5179	0.1503	INDLLSSFG
25.4	992.5787	0.0895	SVSSPMADK
24.6	992.5360	0.1321	SVVAGFSSGF
23.6	992.4485	0.2197	PGDLSVSSTC
23.2	992.4848	0.1833	DNLLVTSMG
22.8	992.5212	0.1469	VAKSVTSGM
21.7	992.5787	0.0895	SVGMGDVEK

Mascot: http://www.matrixscience.com/

SVSTVLTSK

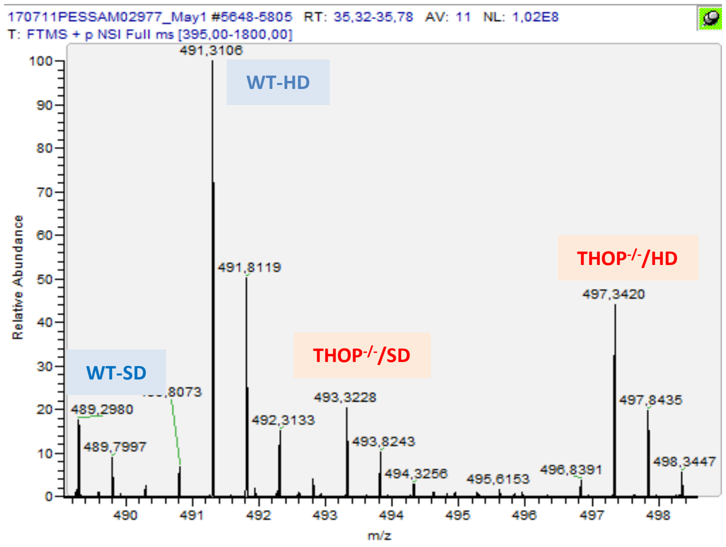
170711PESSAM02977_May1 #5648-5805 RT: 35,32-35,78 AV: 11 NL: 1,02E8
T: FTMS + p NSI Full ms [395,00-1800,00]
WT-HD
WT-SD
THOP$^{-/-}$/HD
THOP$^{-/-}$/SD
Relative Abundance
m/z

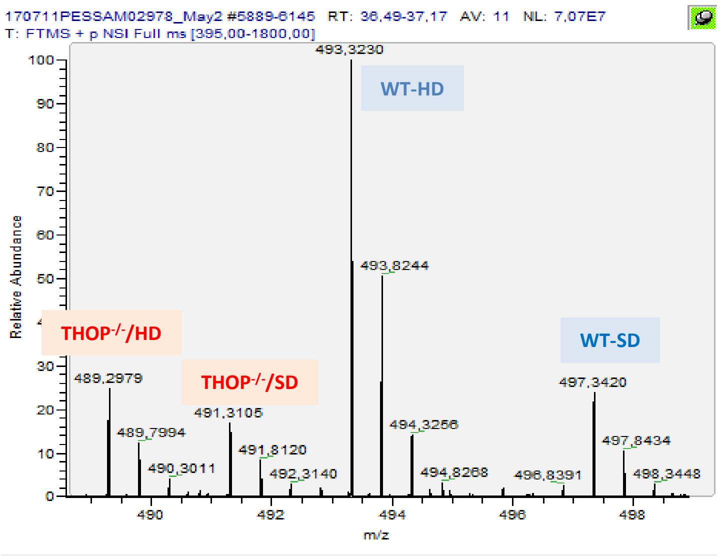
170711PESSAM02978_May2 #5889-6145 RT: 36,49-37,17 AV: 11 NL: 7,07E7
T: FTMS + p NSI Full ms [395,00-1800,00]
WT-HD
WT-SD
THOP$^{-/-}$/HD
THOP$^{-/-}$/SD
Relative Abundance
m/z

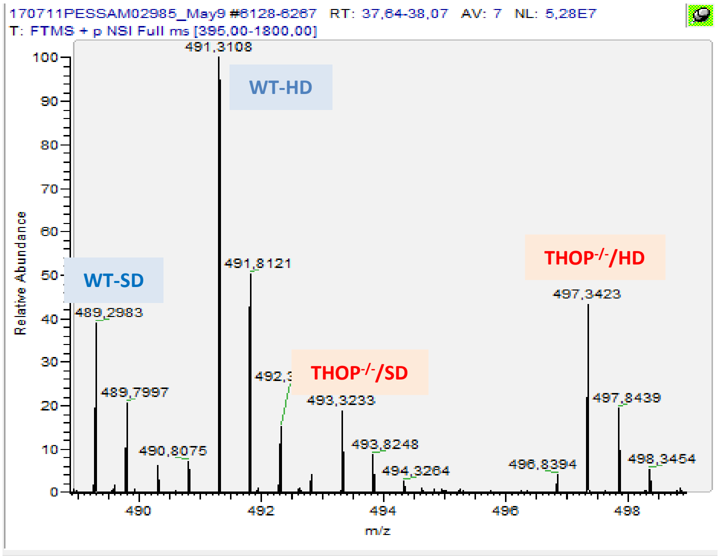
170711PESSAM02985_May9 #6128-6267 RT: 37,64-38,07 AV: 7 NL: 5,28E7
T: FTMS + p NSI Full ms [395,00-1800,00]
WT-HD
WT-SD
THOP$^{-/-}$/HD
THOP$^{-/-}$/SD
Relative Abundance
m/z

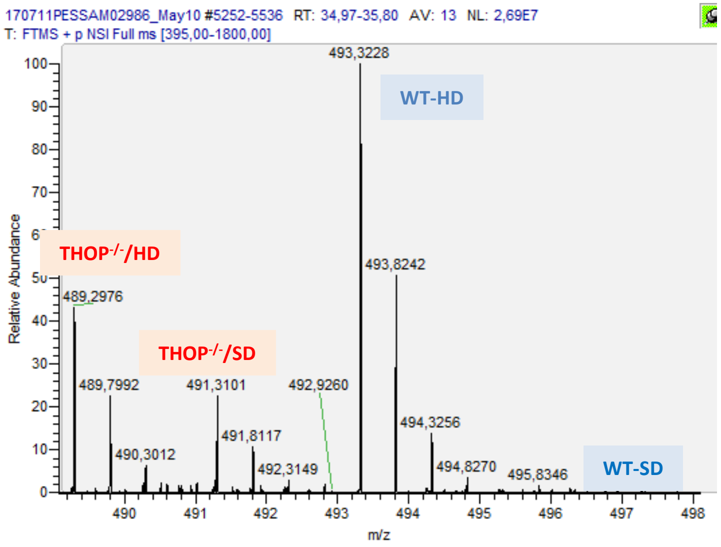
170711PESSAM02986_May10 #5252-5536 RT: 34,97-35,80 AV: 13 NL: 2,69E7
T: FTMS + p NSI Full ms [395,00-1800,00]
WT-HD
WT-SD
THOP$^{-/-}$/HD
THOP$^{-/-}$/SD
Relative Abundance
m/z

SVSTVLTSK

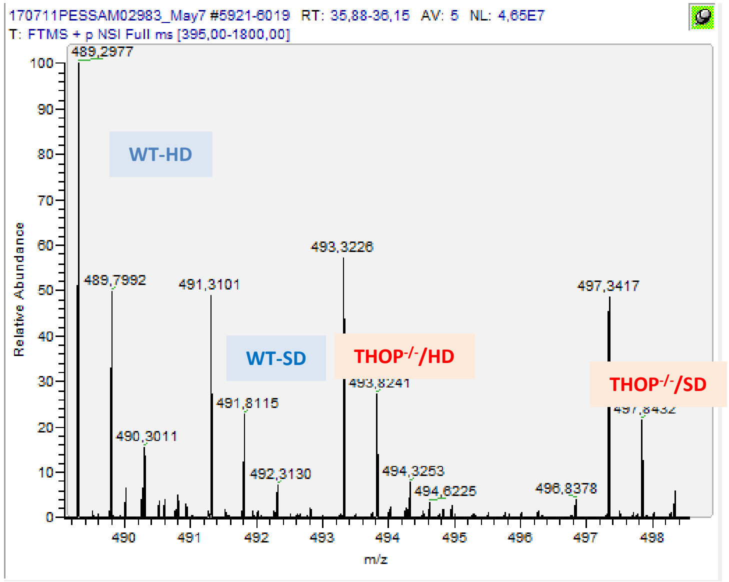

170711PESSAM02983_May7 #5921-6019 RT: 35,88-36,15 AV: 5 NL: 4,65E7
T: FTMS + p NSI Full ms [395,00-1800,00]
WT-HD
WT-SD
THOP-/-/HD
THOP-/-/SD
489,2977
489,7992
490,3011
491,3101
491,8115
492,3130
493,3226
493,8241
494,3253
494,6225
496,8378
497,3417
497,8432
Relative Abundance
m/z

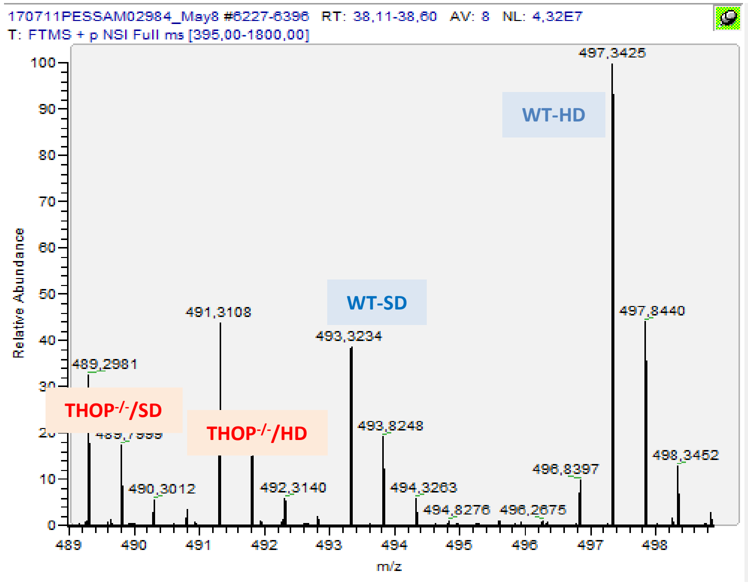

170711PESSAM02984_May8 #6227-6396 RT: 38,11-38,60 AV: 8 NL: 4,32E7
T: FTMS + p NSI Full ms [395,00-1800,00]
WT-HD
WT-SD
THOP-/-/SD
THOP-/-/HD
497,3425
497,8440
498,3452
496,8397
491,3108
493,3234
493,8248
494,3263
494,8276
496,2675
489,2981
489,7999
490,3012
492,3140
Relative Abundance
m/z

Mascot Search Results

Peptide View

MS/MS Fragmentation of **QATVGDVNTDRPGLLDLKGK**
Found in **ACBP_MOUSE** in **SwissProt**, Acyl-CoA-binding protein OS=Mus musculus GN=Dbi PE=1 SV=2

Match to Query 17147: 2186.250912 from(729.757580,3+) intensity(10054624.0000) scans(12874) rawscans(sn12874) rtinseconds(3491.9313) index(8013)
Title: 8014: Scan 12874 (rt=3491.93) [D:\Users\Mayara - HMS Fusion data\170711PESSAM02977_May1.raw]
Data file 170711PESSAM02977_May1.temp.mgf

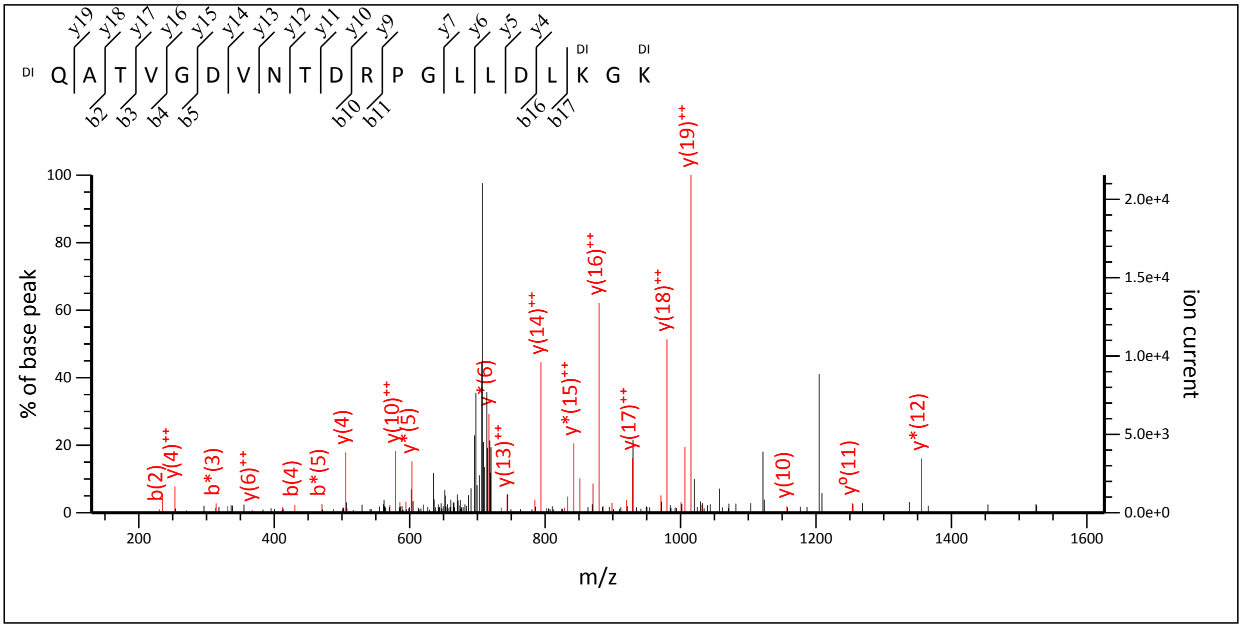

130.26 to 1625.91

Label all possible matches ○ Label matches used for scoring ◉

Monoisotopic mass of neutral peptide Mr(calc): 2186.2634
Variable modifications:
N-term : Dimethyl:2H(2) (N-term)
K18 : Dimethyl:2H(2) (K)
K20 : Dimethyl:2H(2) (K)
Ions Score: 41 **Expect:** 0.0066
Matches : 63/216 fragment ions using 104 most intense peaks (help)

#	b	b++	b*	b*++	b0	b0++	Seq.	y	y++	y*	y*++	y0	y0++	#
1	159.1128	80.0600	142.0863	71.5468			Q							20
2	**230.1499**	115.5786	213.1234	107.0653			A	2029.1652	***1015.0862***	2012.1386	1006.5730	2011.1546	1006.0809	19
3	**331.1976**	166.1024	314.1710	157.5892	313.1870	157.0972	T	1958.1281	***979.5677***	1941.1015	971.0544	1940.1175	970.5624	18
4	**430.2660**	215.6366	413.2395	207.1234	412.2554	206.6314	V	1857.0804	***929.0438***	1840.0538	920.5306	1839.0698	920.0385	17
5	**487.2875**	244.1474	470.2609	235.6341	469.2769	235.1421	G	1758.0120	***879.5096***	1740.9854	870.9964	1740.0014	870.5043	16
6	**602.3144**	301.6608	585.2879	293.1476	584.3039	292.6556	D	1700.9905	***850.9989***	1683.9640	842.4856	1682.9799	841.9936	15
7	701.3828	351.1951	684.3563	342.6818	683.3723	342.1898	V	1585.9636	***793.4854***	1568.9370	784.9721	1567.9530	784.4801	14
8	815.4258	408.2165	798.3992	399.7032	797.4152	399.2112	N	1486.8952	***743.9512***	1469.8686	735.4379	1468.8846	734.9459	13
9	916.4734	458.7404	899.4469	450.2271	898.4629	449.7351	T	1372.8522	686.9298	1355.8257	678.4165	1354.8417	677.9245	12
10	**1031.5004**	516.2538	1014.4738	507.7406	1013.4898	507.2485	D	1271.8045	636.4059	1254.7780	627.8926	1253.7940	627.4006	11
11	1187.6015	**594.3044**	1170.5749	585.7911	1169.5909	585.2991	R	**1156.7776**	***578.8924***	1139.7511	570.3792	1138.7670	569.8872	10
12	1284.6543	642.8308	1267.6277	634.3175	1266.6437	633.8255	P	**1000.6765**	***500.8419***	983.6499	492.3286	982.6659	491.8366	9
13	1341.6757	671.3415	1324.6492	662.8282	1323.6652	662.3362	G	903.6237	452.3155	886.5972	443.8022	885.6132	443.3102	8
14	1454.7598	727.8835	1437.7332	719.3703	1436.7492	718.8782	L	846.6023	423.8048	829.5757	415.2915	828.5917	414.7995	7
15	1567.8438	**784.4256**	1550.8173	775.9123	1549.8333	775.4203	L	733.5182	***367.2627***	716.4917	358.7495	715.5076	358.2575	6

16	1682.8708	**841.9390**	1665.8442	833.4258	1664.8602	832.9338	D	**620.4341**	310.7207	603.4076	302.2074	602.4236	301.7154	5
17	1795.9549	**898.4811**	1778.9283	889.9678	1777.9443	889.4758	L	**505.4072**	***253.2072***	488.3806	244.6940			4
18	1954.0968	977.5520	1937.0702	969.0387	1936.0862	968.5467	K	392.3231	196.6652	375.2966	188.1519			3
19	2011.1182	**1006.0628**	1994.0917	997.5495	1993.1077	997.0575	G	234.1812	117.5942	217.1547	109.0810			2
20							K	177.1598	89.0835	160.1332	80.5702			1

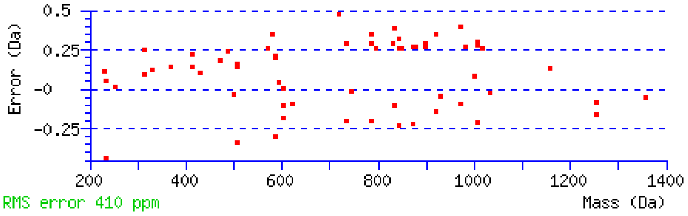

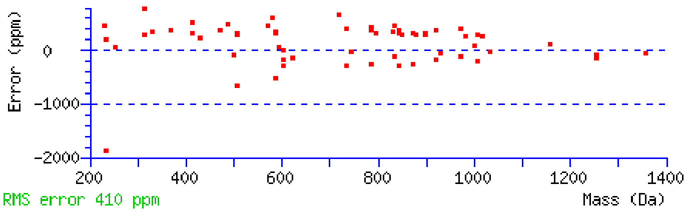

NCBI **BLAST** search of QATVGDVNTDRPGLLDLKGK
(Parameters: blastp, nr protein database, expect=20000, no filter, PAM30)
Other BLAST web gateways

All matches to this query

Score	Mr(calc)	Delta	Sequence
41.4	2186.2634	-0.0125	QATVGDVNTDRPGLLDLKGK
17.4	2186.1636	0.0873	APRKPPSSFLLFSRDHYA
15.9	2186.0460	0.2049	ANDVGMIQTAHVGVGISGNEGM
15.7	2186.1623	0.0886	SATDRKIIFQVIDYVSAY
14.2	2186.0416	0.2093	YSTPSPATIETQSSSSEEIV
13.0	2186.0678	0.1831	QATVGNSYKCNTEEHIFVS
12.9	2186.1486	0.1023	YVVFLKLFLETAEQHFM
12.5	2186.1261	0.1248	PPAGDDGSGSRPQRLLWQTA
11.7	2186.0060	0.2449	SNGNRRHESDEDSLGSSGRV
11.3	2186.1253	0.1256	VTPVPGQTLVTMATATVTANNG

Mascot: http://www.matrixscience.com/

QATVGDVNTDRPGLLDLKGK

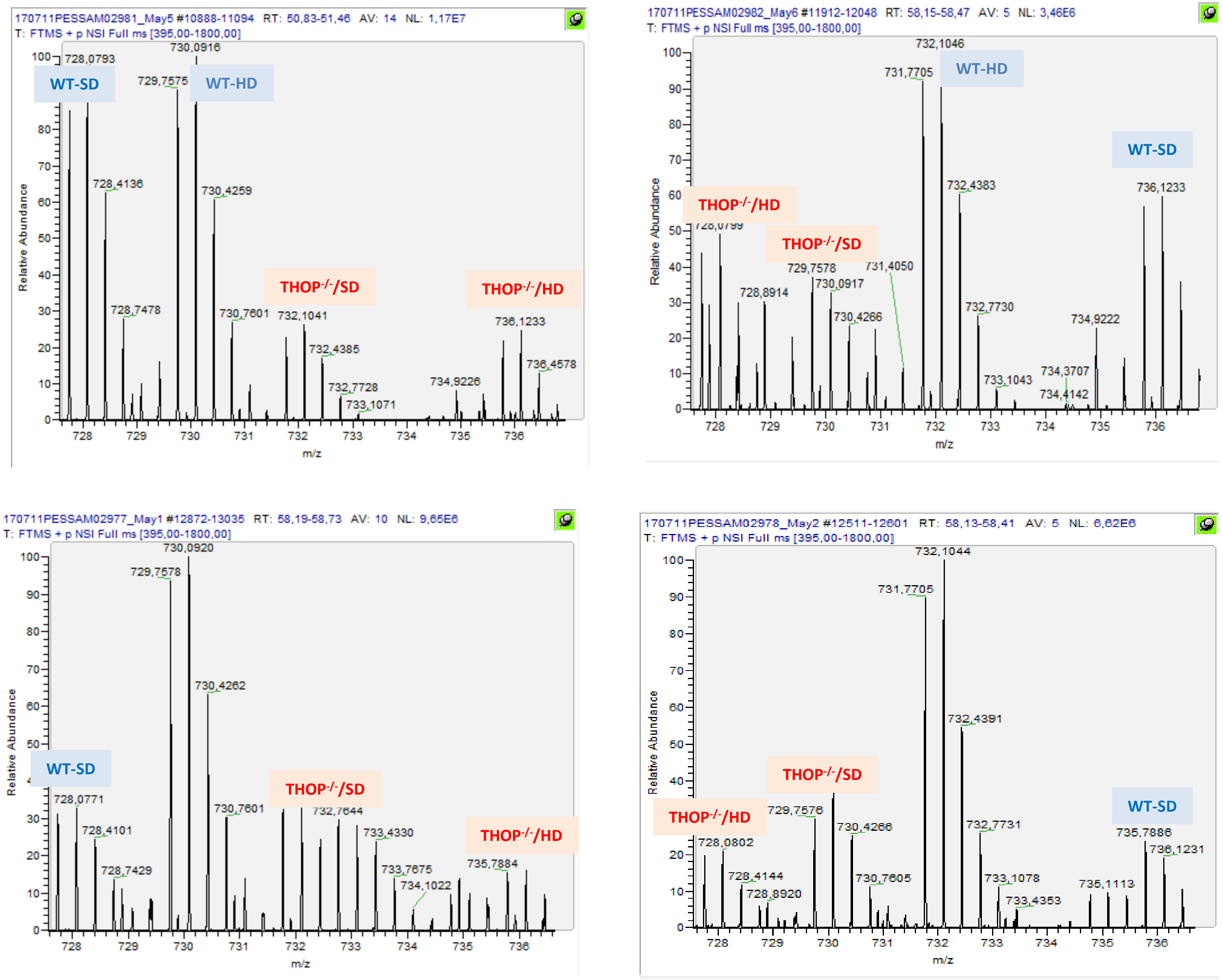

QATVGDVNTDRPGLLDLKGK

May7 –not found

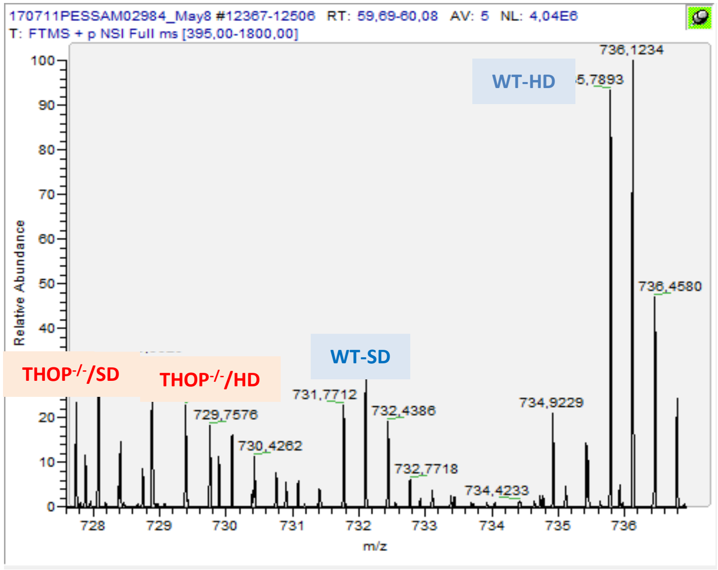

Supplement: Supplementary file 1 [file biomolecules-10-00321-s001.zip › biomolecules-700233-Supporting Information/Additional Mass Spectrometry Supporting Information.pdf]
