# Supplementary material for: The Relevance of Thimet Oligopeptidase in the Regulation of Energy Metabolism and Diet-Induced Obesity
Source: Biomolecules. 2020 Feb 17;10(2):321. doi: 10.3390/biom10020321 (PMC7072564; doi:10.3390/biom10020321)
Supplement: Supplementary file 1 [file biomolecules-10-00321-s001.zip › biomolecules-700233-Supporting Information/Returning Proof readings - Gewehr et al - Biomolecules - Supplemental.pdf]

Supplemental Table S1. Primer sequences.

Gene	Sequence	Amplicon (bp)	Accession number
**Peroxisome proliferator activated receptor gamma (PPAR-γ)**	Fwd: ATCTTAACTGCCGGATCCAC<br>Rev: CAAACCTGATGGCATTGTGAG	102	NM_001127330.2
**Peroxisome proliferator activated receptor alpha (PPAR-α)**	Fwd: TGCAATTCGCTTTGGAAGAA<br>Rev: CTTGCCCAGAGATTTGAGGT	118	NM_011144.6
**Fat acid synthase (FAS)**	Fwd: GATTCGGTGTATCCTGCTGTC<br>Rev: CATGCTTTAGCACCTGCTGT	95	NM_007988.3
**Lipoprotein lipase (LPL)**	Fwd: GTCTGGCTGACACTGGACAAA<br>Rev: CCCACTTTCAAACACCCAAA	122	NM_008509.2
**CD36 molecule (CD36)**	Fwd: GATGAATGGTTGAGACCCCG<br>Rev: GCTCCACACATTTCAGAAGGC	174	NM_001159558.1
**Mannose receptor (CD206)**	Fwd: CTGTGTTCAGCTATTGGACGC<br>Rev: CGGAATTTCTGGGATTCAGCTTC	133	NM_008625.2
**Integrin alpha X (CD11C)**	Fwd: CTGGATAGCCTTTCTTCTGCTG<br>Rev: GCACACTGTGTCCGAACTCA	113	NM_021334.3
**Adhesion G protein-coupled receptor E1 (F4/80)**	Fwd: AACATGCAACCTGCCACAAC<br>Rev: TTCACAGGATTCGTCCAGGC	110	NM_010130.4
**Fatty acid-binding protein 4 (FAPB4)**	Fwd: CGCAGACGACAGGAAGGT<br>Rev: TTCCATCCCACTTCTGCAC	77	NM_024406.3
**Ribosomal protein L19 (RPL19)**	Fwd: CAATGCCAACTCCCGTCA<br>Rev: GTGTTTTTCCGGCAACGAG	102	NM_009078.2
**Adrenergic receptor, beta 1 (β1AR)**	Fwd: CATCATGGGTGTGTTCACG<br>Rev: GAAGACGAAGAGGCGATCC	100	NM_007419.3
**Adrenergic receptor, beta 2 (β2AR)**	Fwd: AGGCATGGAAGGCTTTGTGA<br>Rev: TATTACAGTGGCGAGTCATT	99	NM_007420.3
**Adrenergic receptor, beta 3 (β3AR)**	Fwd: ACCCTGATGATCGACATGTTCC<br>Rev: GCCATAGTGAGGAGACAGGG	129	NM_013462.3
**Neprilysin (NEP)**	Fwd: CCTGAACTTTGCCCAGGTGT<br>Rev: GCGGCAATGAAAGGCATCTG	148	NM_001289462.1
**Angiotensin I converting enzyme (ACE1)**	Fwd: ACCCTAGGACCTGCCAATCT<br>Rev: CGTGAGGAAGCCAGGATGTT	164	NM_207624.5
**Prolyl oligopeptidase (POP)**	Fwd: GGGTGCTCCGACACTAAACA<br>Rev: GACGGGTACTGGATGTCGTC	98	NM_011156.3
**Insulin degrading enzyme (IDE)**	Fwd: GTCCATGTTCTTGCCAGGGA<br>Rev: TTCACGAGGGGAAACAGTGG	161	NM_031156.3
**Dipeptidylpeptidase 4 (DPP4)**	Fwd: GACGGCAGAGGAAGTGGTT<br>Rev: CGCTTGCTATCCACAAATCCC	134	NM_010074.3
**Proteasome subunit beta 5 (Protβ5)**	Fwd: CCAAACTGCTCGCTAACATGG<br>Rev: GTTCCCCTCGCTGTCTACG	119	NM_011186.1

Supplemental Table S2. Schematic distribution of "May" peptidomic samples.

Samples	Light 1	Light 2	Intermediary	Heavy
May 1	WT/SD	WT/HD	$THOP1^{-/-}$/SD	$THOP1^{-/-}$/HD
May 2	$THOP1^{-/-}$/HD	$THOP1^{-/-}$/SD	WT/HD	WT/SD
May 3	WT/HD	WT/SD	$THOP1^{-/-}$/HD	$THOP1^{-/-}$/SD
May 4	$THOP1^{-/-}$/SD	$THOP1^{-/-}$/HD	WT/SD	WT/HD
May 5	WT/SD	WT/HD	$THOP1^{-/-}$/SD	$THOP1^{-/-}$/HD
May 6	$THOP1^{-/-}$/HD	$THOP1^{-/-}$/SD	WT/HD	WT/SD
May 7	WT/HD	WT/SD	$THOP1^{-/-}$/HD	$THOP1^{-/-}$/SD
May 8	$THOP1^{-/-}$/SD	$THOP1^{-/-}$/HD	WT/SD	WT/HD
May 9	WT/SD	WT/HD	$THOP1^{-/-}$/SD	$THOP1^{-/-}$/HD
May 10	$THOP1^{-/-}$/HD	$THOP1^{-/-}$/SD	WT/HD	WT/SD

Footnotes: A total of 20 µg of peptides were extracted from inguinal adipose tissue (*n* = 5 for each group); 5 µg of peptides from each group were labeled with isotope, as follows:

- Light 1: Add 28 Da of mass per each amino group (N-terminal) and/or Lys residue(s): chemical formula of isotope = $(CH_3)_2$–N–R
- Light 2: Add 30 Da of mass per each amino group (N-terminal) and/or Lys residue(s): chemical formula of isotope = $(CDH_2)_2$–N–R
- Intermediary: Add 32 Da of mass per each amino group (N-terminal) and/or Lys residue(s): chemical formula of isotope = $(CHD_2)_2$–N–R
- Heavy: Add 36 Da of mass per each amino group (N-terminal) and/or Lys residue(s): chemical formula of isotope = $(^{13}CD_3)_2$–N–R

Samples are from male WT or $THOP1^{-/-}$ mice (PROTX gene knockout animals on C57BL6 genetic background).

For each experimental group, there were 5 animals, and each group was treated according to 4 different protocols (SD or HD, WT or $THOP1^{-/-}$), and all were labeled in duplicates (labeling was done in "forward" and "reverse" for each group):

- Group WT/SD, n = 5
- Group WT/HD, n = 5
- Group $THOP1^{-/-}$/SD, *n* = 5
- Group $THOP1^{-/-}$/HD, *n* = 5

Supplemental Table S3. Complete data analyses for the identified intracellular peptides

Precursor Protein name	Peptide sequence	RT	L1_m/z	L2_m/z	I_m/z	H_m/z	z	T	Avg. mass	SD	Obs. mass	Theor. mass	ppm
Macrophage migration inhib. factor	LSELTQQLAQATGKPAQ	64.7	920.5638	922.5580	924.5377	928.55860	2	2	1783.0083	0.04	1783.0083	1782.9549	29.9
Histone H2A type 1-B	AQGGVLPNIQAVLLPK	96.9	837.5191	839.5303	841.5442	845.5635	2	2	1616.9778	0.02	1616.9778	1616.9687	5.6
Histone H2B type 2-B	KQVHPDTGISSKAMGI**m**NS	37.6	701.0305	703.0540	705.0552	709.0762	3	3	2016.0107	0.04	2016.0107	2015.9792	15.6
Elongation factor 2	ASVLTAQPRLMEPI	80.1	777.4376	778.4376	779.4517	781.4618	2	1	1524.8360	0.01	1524.8360	1524.8407	-3.1
Creatine kinase M-type	DISNADRLGSSEVEQ	49.1	824.4038	825.4003	826.4043	828.4151	2	1	1618.7534	0.01	1618.7534	1618.66	57.7
Creatine kinase M-type	DISNADRLGSSEVEQV	59.5	873.929	874.9346	875.9405	877.9507	2	1	1717.8190	0.01	1717.8190	1717.8191	-0.1
Creatine kinase M-type	IDDHFLFDKPVSPLL	99.3	906.50320	908.51070	910.52580	914.5468	2	2	1754.9425	0.02	1754.9425	1754.9316	6.2
Apolipoprotein A-I	LETLKTQVQSVIDKA	73.2	586.3524	588.3640	590.3767	594.3958	3	3	1671.9655	0.04	1671.9655	1671.948	10.5
Apolipoprotein A-II	FSSLMNLEEKPAPAA	71.5	830.9321	832.9461	834.9584	838.9783	2	2	1603.8067	0.02	1603.8067	1603.7989	4.8
Apolipoprotein A-II	HEQLTPLVRSAGTSLVN	60.0	925.5099	926.5137	927.5212	929.5321	2	1	1820.9801	0.01	1820.9801	1820.9817	-0.9
Serum albumin	SQTFPNADFAEITKL	93.2	869.46	871.4683	873.4811	877.4998	2	2	1680.8538	0.02	1680.8538	1680.8432	6.3
Non-specific lipid-transfer protein	ADSDLLALMTGKMNPQSA	99.0	959.9837	961.9929	964.0899	968.0289	2	2	1861.9469	0.10	1861.9469	1861.8987	25.9
Hemoglobin subunit alpha	GAEALERMFASFPTTK	87.8	906.5109	908.4832	910.4923	914.5168	2	2	1754.9008	0.03	1754.9008	1754.834	38.1
Hemoglobin subunit alpha	FDVSHGSAQVK	24.6	615.8264	617.839	619.8522	623.8708	2	2	1173.5936	0.02	1173.5936	1173.5851	7.2
Hemoglobin subunit alpha	IGGHGAEYGAEALER	39.5	779.3855	780.3916	781.3956	783.4088	2	1	1528.7324	0.01	1528.7324	1528.7343	-1.3
Hemoglobin subunit alpha	SVSTVLTSK	35.3	489.298	491.3106	493.3228	497.342	2	2	920.5359	0.02	920.5359	920.5251	11.7
Acyl-CoA-binding protein	QATVGDVNTDRPGLLDLKGK	58.5	727.7425	729.7576	731.7711	735.7884	3	3	2096.1435	0.04	2096.1435	2096.1299	6.5

RT= retention time; m= metionine oxidized; L1 or2 =Light; I=Intermediary; H=Heavy; z=charge; T= number of tags; SD= standard deviation;

Supplemental Fig. S1. Gewehr et al.

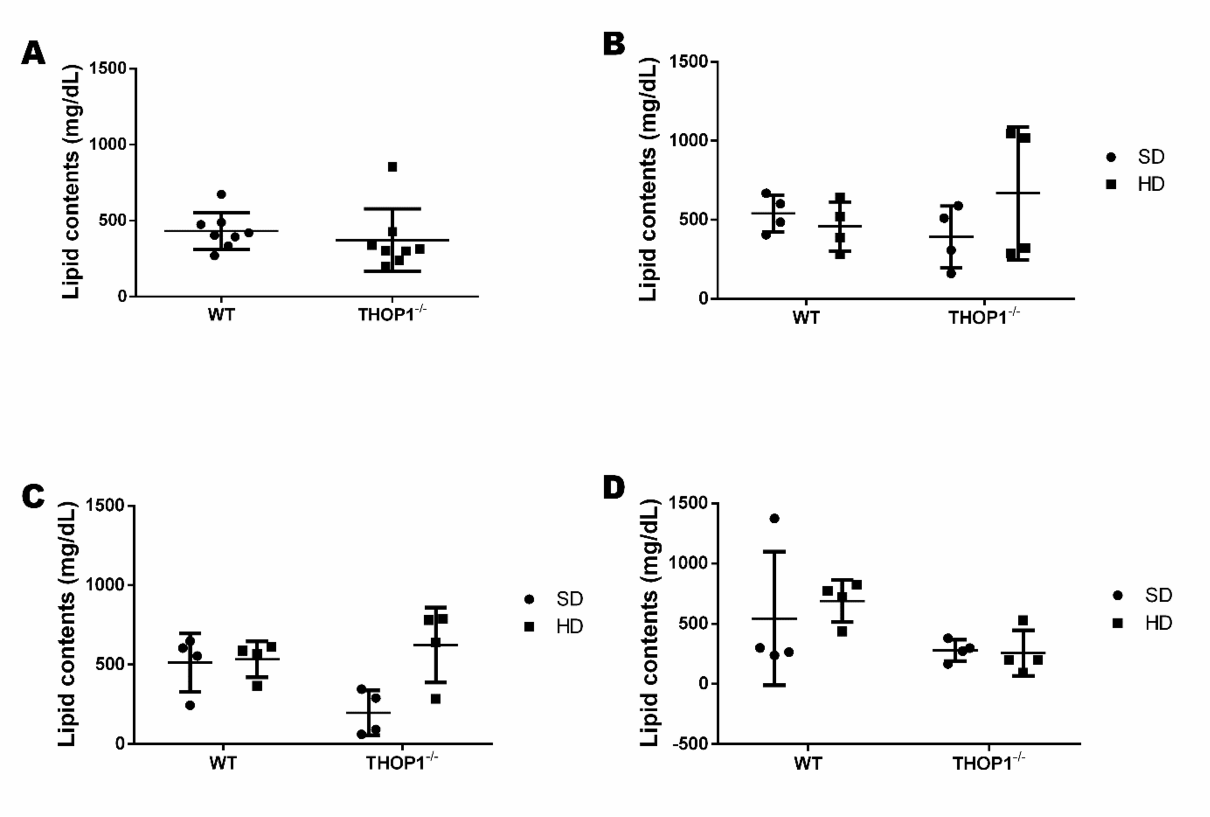

Supplemental Figure S1. Quantification of lipid content in animal feces: (A) before diet started, (B) after 6 weeks of the diet, (C) after 12 weeks of the diet, and (D) after 24 weeks of the diet. Determination of the lipid content in animal feces was performed by extraction using an organic solvent (n = 4). Results are expressed as mean ± SEM. Statistical analyses were performed using Student's unpaired t-test (n = 4).

Supplemental Fig S2. Gewehr et al.

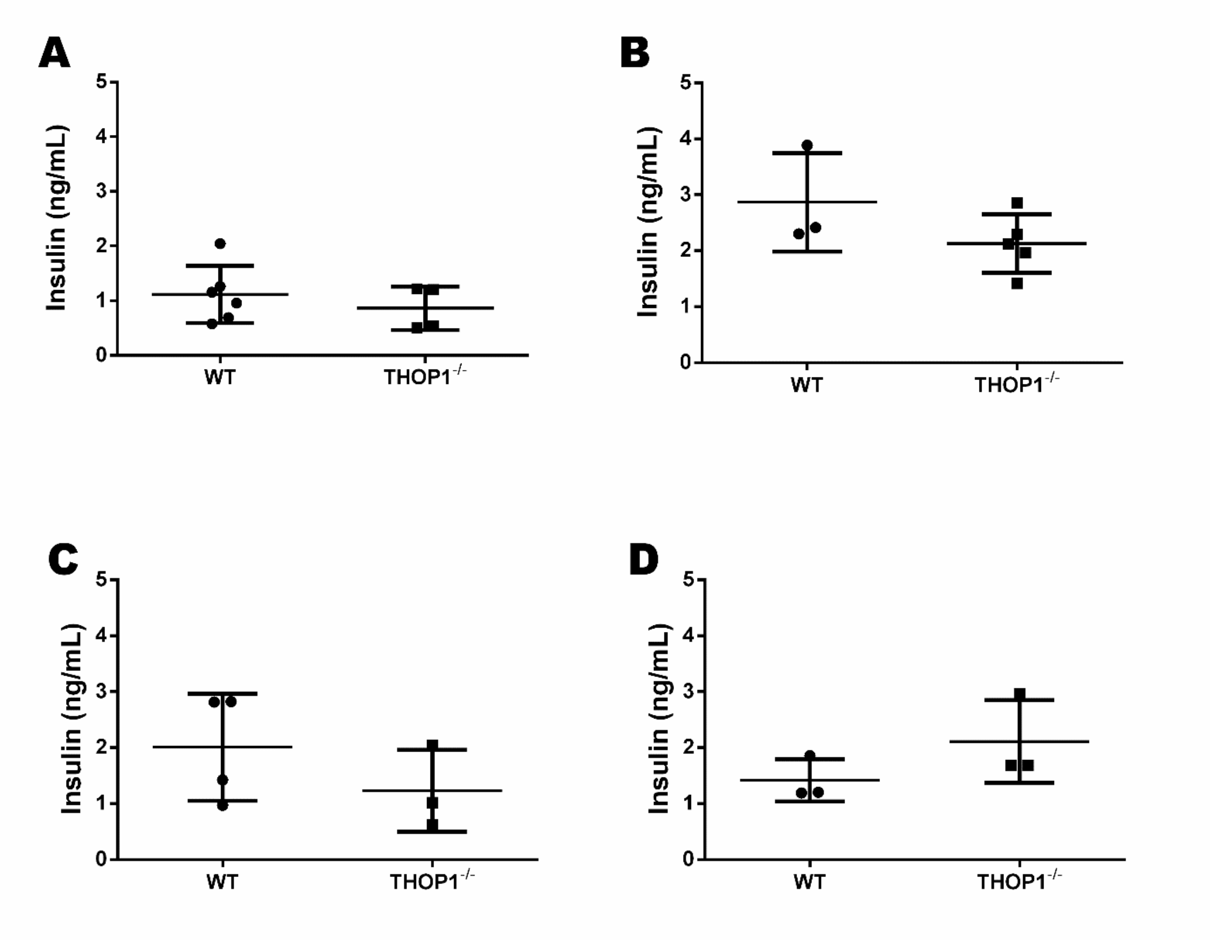

Supplemental Figure S2. Pre-prandial insulin levels of female (A,C) and male (B,D) mice, WT or THOP1$^{-/-}$, were evaluated in animals fasted for 10 h (A,B) or 4 h (C,D). Experimental details are shown in the Methods section. Note that no differences were observed in the insulin levels among WT or THOP1$^{-/-}$ mice (n = 3–4).

Supplemental Figure S3. Gewehr et al.

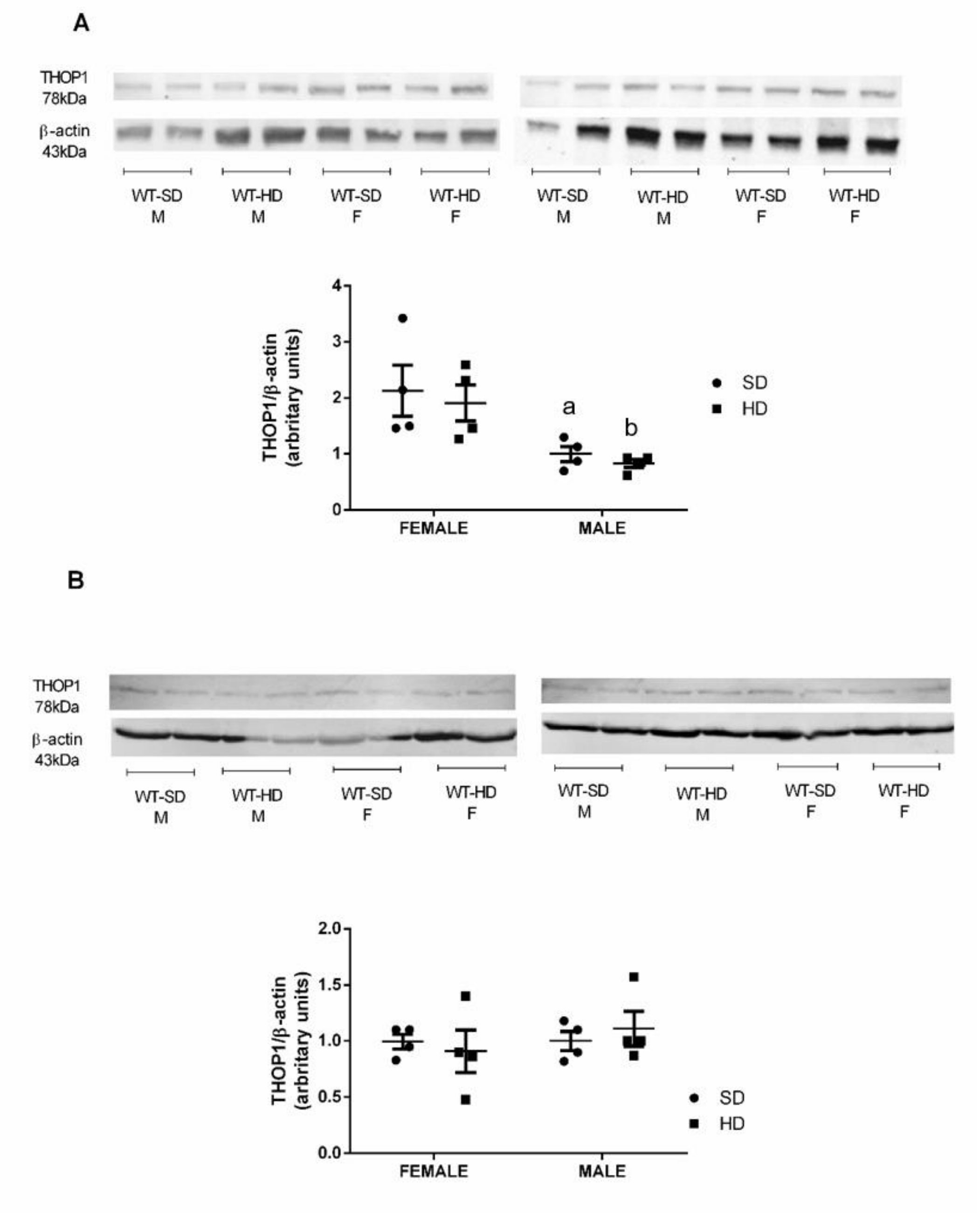

Supplemental Figure S3. Western blots show immunoreactivity for THOP1 and beta-actin on adipose (A) and liver (B) tissue homogenates from WT animals. Note, no differences were observed in THOP1 expression levels among the groups ($n = 4$). M, male; F, female. Results are expressed as mean ± SEM. Statistical analyses were conducted using Student's unpaired *t*-test. One letter, $p \leq 0.05$; a, female WT/SD *vs*. male WT/SD; b, female WT/HD *vs*. male WT/HD ($n = 4$).

Supplemental Figure S4. Gewehr et al.

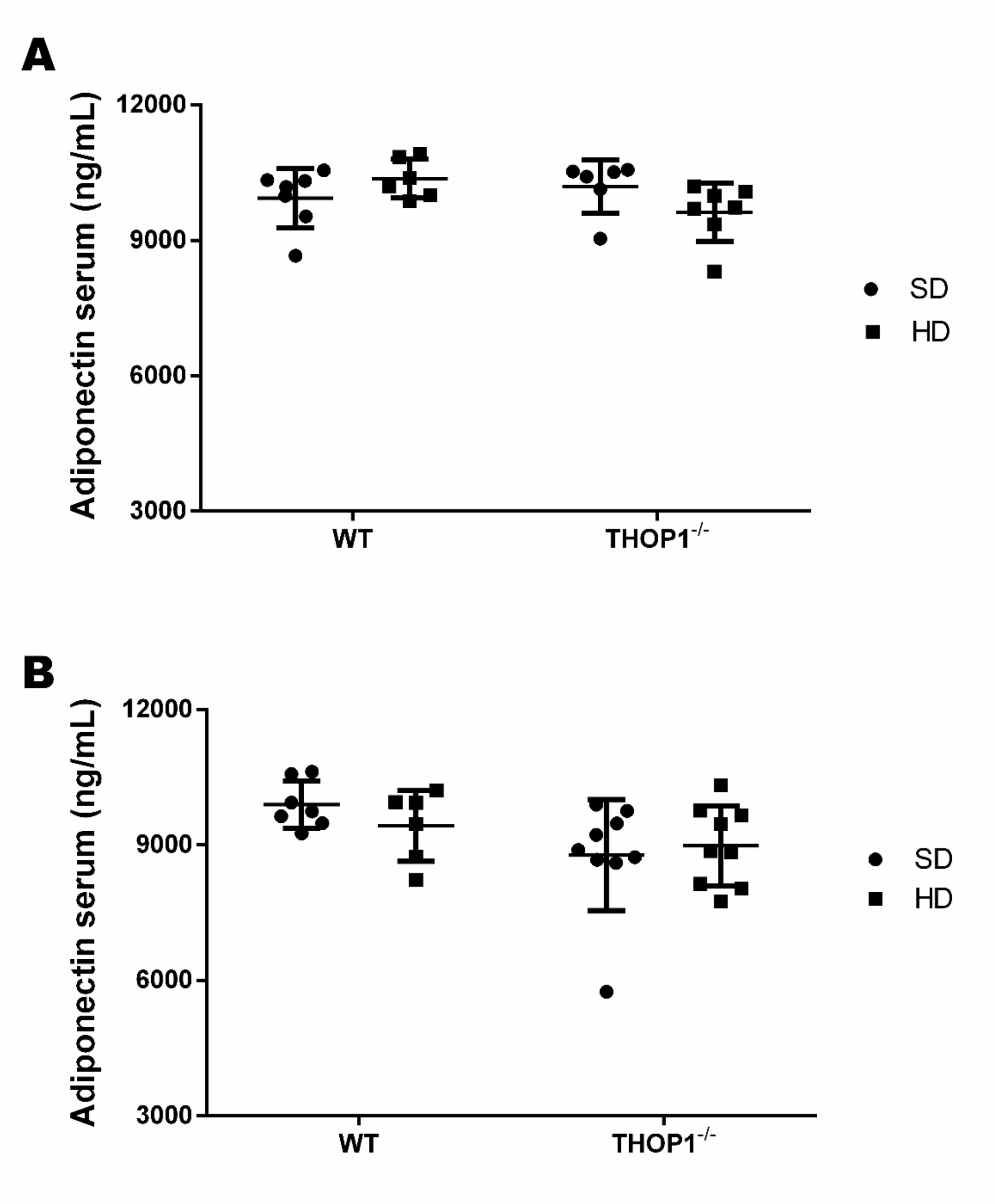

Supplemental Figure S4. Adiponectin levels of female (A) and male (B) mice were evaluated after 10 h of food restriction (pre-prandial). Experimental details are shown in the Methods section. Note that no differences were observed in adiponectin levels among the WT or THOP1$^{-/-}$ groups (n = 6–9).

Supplemental Fig S5. Gewehr et al.

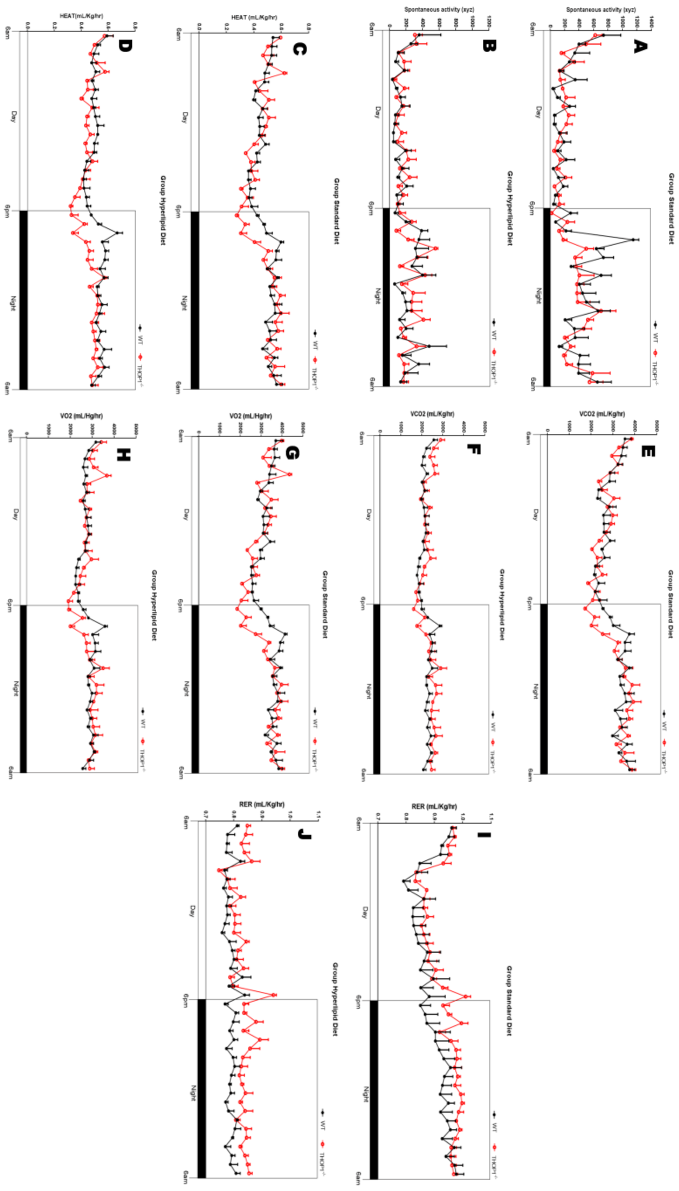

Supplemental Figure S5. Resting energy metabolism of WT (black lanes) or THOP1$^{-/-}$ (red lanes) male mice across 24 h. Panels: (A,B) spontaneous locomotor activity; (C,D) heat production; (E,F) VCO_2 (mL/kg/h) production; (G,H) VO_2 (mL/kg/h) consumption; (I,J) respiratory exchange ratios (RER). Panels: (A,C,E,G,I) mice were fed an SD; (B,D,F,H,J) mice were fed a HD. Statistical analyses are shown in Fig 5 ($n = 4$). Note that an RER of 0.7 indicates that fat is the predominant fuel source, a value of 1.0 is indicative of carbohydrate being the predominant fuel source, and a value between 0.7 and 1.0 suggests a mix of both fat and carbohydrate (Kenney, W. Larry. (2012). Physiology of sport and exercise. Wilmore, Jack H., 1938–2014., Costill, David L., Wilmore, Jack H., 1938–2014. 5th ed., Champaign, IL: Human Kinetics. pp. 117–118. ISBN 9780736094092).

Supplemental Fig S6. Gewehr et al.

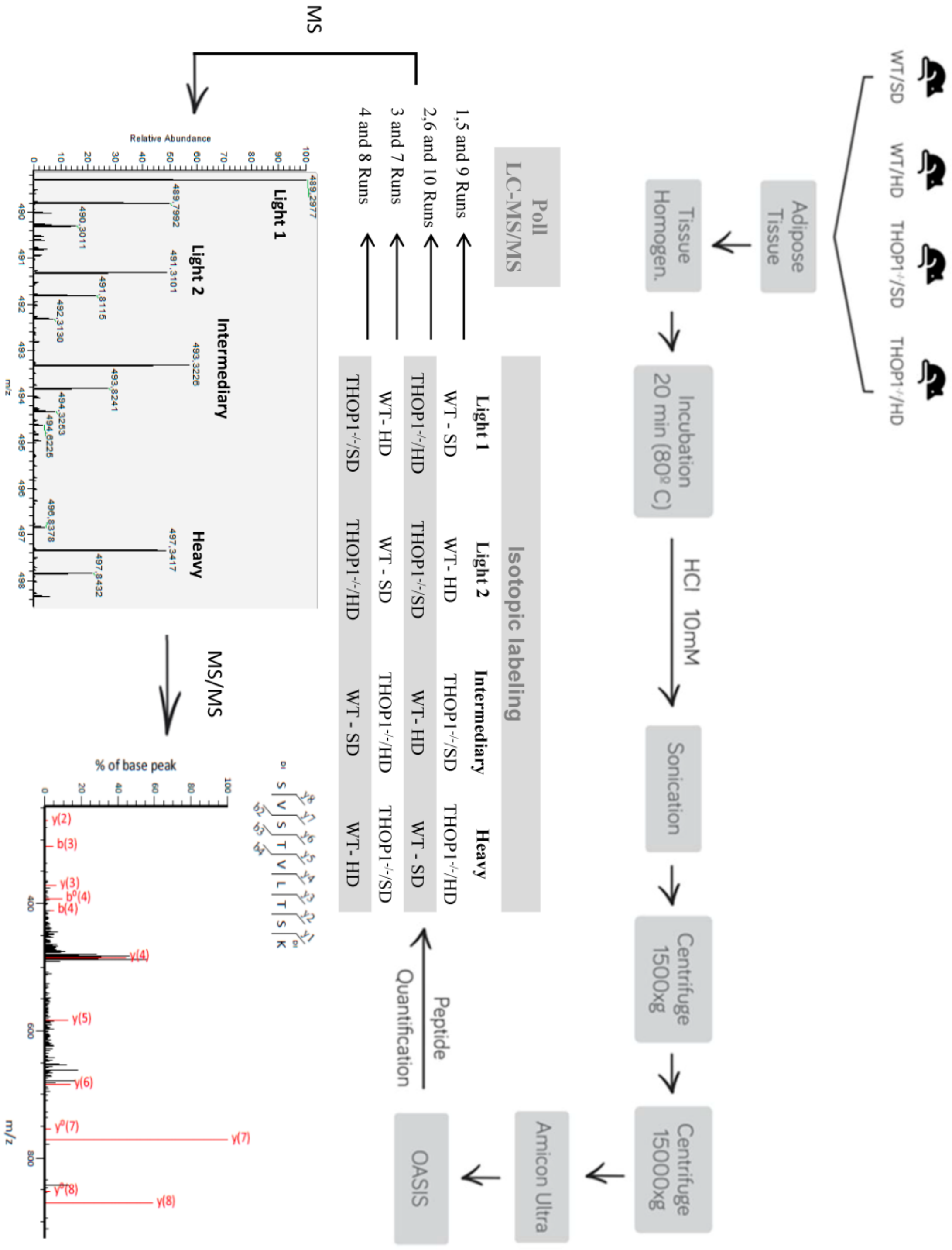

Supplemental Figure S6. Schematic representation to illustrate the semi-quantitative peptidomic analyses. Experimental details are presented in the Methods section.
